# Insights Into the Origin and Local Adaptation Evolution of the Cultivated Sesame With Telomere‐to‐Telomere High‐Quality Genome

**DOI:** 10.1111/pbi.70714

**Published:** 2026-07-04

**Authors:** Weifei Yang, Hengchun Cao, Hui Guo, Hongmei Miao, Guiting Li, Yinghui Duan, Ming Ju, Qiuzhen Tian, Qin Ma, Pengjie Chang, Zhanyou Zhang, Cong Mu, Xiaoxu Feng, Libin Wei, Weixiu Hou, Andrew H. Paterson, Haiyang Zhang

**Affiliations:** ^1^ Henan Sesame Research Center Henan Academy of Agricultural Sciences Zhengzhou Henan China; ^2^ Henan Key Laboratory of Specific Oilseed Crops Genomics (Henan Sesame Research Center Henan Academy of Agricultural Sciences) Zhengzhou Henan China; ^3^ Henan International Joint Key Laboratory of Special Oilseed Crops Improvement Zhengzhou Henan China; ^4^ The Shennong Laboratory Zhengzhou Henan China; ^5^ Plant Genome Mapping Laboratory University of Georgia Athens Georgia USA; ^6^ College of Agronomy Henan Agricultural University Zhengzhou Henan China

**Keywords:** days from sowing to flowering, GWAS, predictive modelling, *sesamum*, *SiUBP16*

## Abstract

Sesame ( *Sesamum indicum* L., 2*n* = 26) is one of the oldest oilseed crops and is often called the ‘queen of oilseeds’ due to its high content of unsaturated fatty acids and natural antioxidants. Despite its long history, the origin and global spread of cultivated sesame remain unresolved. We assembled a telomere‐to‐telomere (T2T), high‐quality reference genome of sesame (cv. Yuzhi11) to investigate sequence differences between genomes and its origin and the local adaptation evolution of flowering time (DF). We generated a 305 Mb T2T sesame reference genome (cv. Yuzhi11) with > 99.99% base‐level accuracy, identifying 31 063 protein‐coding genes. Repetitive elements accounted for 52.03% of the genome. Population genomic analysis of 927 accessions from 14 regions identified four major groups. Integrative analyses of linkage disequilibrium decay (LD), nucleotide diversity (π), and fixation index (*F*_ST_) support East Africa as the center of origin, with subsequent migration through the Middle East, to South Asia, South‐East Asia, East Asia and ultimately to other parts of the world. Genome‐wide association studies (GWAS) and selection scans identified 30 genes associated with flowering time. *SiUBP16* is a candidate associated with 7.6% of DF variation. Early‐flowering accessions carried up to 225 favourable alleles. A flowering time prediction model for high‐latitude regions achieved 96% accuracy. We present a high‐quality T2T reference genome for cultivated sesame, shedding light on its origin, evolutionary history, and regional flowering time adaptation. This genome insights valuable tools for breeding programs aimed at improving yield and environmental adaptation in sesame and related crops.

## 1 Introduction

Sesame ( *Sesamum indicum* L. 2*n* = 2*x* = 26), the only cultivated species of the *Sesamum* genus in the Pedaliaceae family, is one of the oldest known oilseed crops, with evidence of its cultivation dating back to 3000–3050 bc (Bedigian and Harlan 1986). Today, sesame is primarily cultivated in tropical and subtropical regions (Zhang et al. 2013; Anastasi et al. 2017), with Africa and Asia contributing 96.3% of global sesame seed production in FAOSTAT database at 2021. Major producing countries include Sudan, India, Tanzania, Myanmar, and China. Sesame seeds contain approximately 55% oil, 20% protein, and 0.8% lignans, and are widely recognised for their high nutritional and antioxidant properties, earning the title ‘the queen of oilseeds’ (Bedigian and Harlan 1986; Zhang et al. 2019). Owing to its nutritional value and medicinal uses, sesame plays an important role in human diets and health care. Global sesame production and cultivated area have increased steadily over recent decades (Alipoor et al. 2012; Bedigian and Harlan 1986; Sukumar et al. 2008; Zhang et al. 2019). Additionally, sesame's short life cycle (70–90 days), prolific seed production (~6000 seeds per plant), and small genome size (estimated at 312.95 Mb) make it an attractive model for studying angiosperm evolution and biological traits (Miao et al. 2024; Zhang et al. 2019).

The *Sesamum* genus comprises 36 species with three chromosome numbers (*n* = 13, 16, and 32) (Kobayashi 1991; Zhang et al. 2019, 2021). Despite extensive research, the geographic origin and domestication pathway of cultivated sesame remain unresolved, as its wild progenitor has not been definitively identified (Ashri 1998; Bedigian and Harlan 1986; Bedigian 2003, 2004, 2010; De Candolle 1885; Hiltebrandt 1932; Miao et al. 2021; Nayar 1995; Nayar and Mehra 1970; Nanthakumar et al. 2000; Vavilov and Dorofeev 1992; Weiss 1983). The abundance of *Sesamum* species in Africa has led many scholars to propose an African origin (Joshi et al. 1961; Kobayashi 1991; Ekta Sharma et al. 2014; Weiss 1983). In contrast, the rich diversity of cultivated sesame in Asia has prompted others to argue for domestication in the Indian subcontinent (Bedigian 1984, 1988; Bedigian et al. 1985; Bedigian and Harlan 1986; Bhat et al. 1999; D. Fuller 2002; Fuller and Madella 2001; Powell 1991; Zohary and Hopf 2000). Archaeological discoveries of charred seed have further supported the hypothesis that sesame was domesticated in northwestern South Asia during the Harappan civilisation (D. Q. Fuller 2003).

In recent years, genomic approaches have been increasingly employed to explore sesame's origins and domestication history (Dossa et al. 2016; Miao et al. 2024; Wei et al. 2015). Wei et al. (2015) analysed the population structure of 705 diverse sesame varieties and conducted genome‐wide association studies (GWAS) for 56 agronomic traits, identifying two major genetic groups and evidence of long‐term selection in the northern‐area group. Dossa et al. (2016), using 33 highly polymorphic SSR markers to genotype 96 accessions, reported great genetic diversity, highlighting a strong correlation between genetic diversity and geographic origin.

To further elucidate the evolutionary history of the *Sesamum* genus and accelerate molecular breeding, a Sesame Genome Project was initiated in 2010 by the Sesame Genome Working Group (Miao et al. 2024; Zhang, Miao, et al. 2013). In 2024, chromosome‐scale genome assemblies were published for cultivated sesame (var. Yuzhi11, *n* = 13) and six wild *Sesamum* species, i.e., *S. alatum* (*n* = 13), *S. latifolium* (*n* = 16), *S. angolense* (*n* = 16), *S. calycinum* (*n* = 16), *S. angustifolium* (*n* = 16), and *S. radiatum* (*n* = 2*x* = 32), representing all three known karyotypes within the genus (Cao et al. 2024; Miao et al. 2024). Phylogenomic analysis indicated an evolutionary trajectory from *n* = 13 to *n* = 16, followed by allotetraploidisation in *S. radiatum* . Karyotyping and genome‐based phylogenetic analysis suggest that cultivated sesame (*n* = 13) diverged prior to 34.2 million years ago (MYA) during the early Tertiary period (65.5–23.3 MYA). In addition, seven additional sesame genomes have been released, comprising two cultivated varieties, four landraces, and a wild allotetraploid sesame (Song et al. 2023; Wang et al. 2023). These genomic resources have been instrumental in elucidating the pivotal role of structural variations (SVs) in shaping sesame's yield, quality, and environmental adaptability. Notably, Wang et al. (2023) demonstrated that subfunctionalisation of duplicated genes is a key driver in the formation and evolutionary success of allotetraploid sesame. While previous studies have laid a solid foundation, they largely rely on assemblies with unresolved gaps and incomplete telomeric and centromeric regions. In contrast, our telomere‐to‐telomere (T2T) assembly eliminates these gaps and fully resolves both telomeric and centromeric sequences, thereby enabling an unprecedented resolution of SVs and centromere‐specific gene content. Nonetheless, additional genomic evidence is needed to fully resolve the origin and domestication history of *S. indicum* .

Here, we assembled a high‐quality telomere‐to‐telomere (T2T) reference genome for sesame and systematically exploited natural variations in 927 accessions collected from 42 countries. We aimed to clarify the geographic origin and global dissemination of cultivated sesame, and to dissect the genomic basis underlying key adaptive traits, including flowering time (days from sowing to flowering, DF). The findings from this study provide new insights into the evolutionary and migration history of sesame and establish a valuable resource for breeding programs targeting enhanced yield and environmental adaptation in sesame and other crops.

## 2 Results

### 2.1 T2T Genome Sequencing and Assembly of Cultivated Sesame

We assembled a T2T reference genome for cultivated sesame ( *Sesamum indicum* var. Yuzhi 11) using an integrated sequencing strategy combining PacBio HiFi, Oxford Nanopore ultra‐long reads, DNBSEQ Hi‐C and BioNano optical mapping (Figure S1; Table S1). In total, we generated 40.5 Gb of clean DNBSEQ paired‐end reads (~131.9× genome coverage), 30.3 Gb of HiFi reads (~98.7×), 25.6 Gb Nanopore ultra‐long reads (~83.4×), 38.4 Gb Hi‐C reads (~125.2× depth), and 123.9 Gb BioNano molecules (~406 ×) to support genome assembly (Table S1). K‐mer (*k* = 19) analysis estimated the sesame genome (var. Yuzhi 11) size is 307.07 Mb with a heterozygosity rate of 0.43%, and repetitive sequences accounting for 39.51% of the genome (Figure S2; Table S2).

**FIGURE 1:**
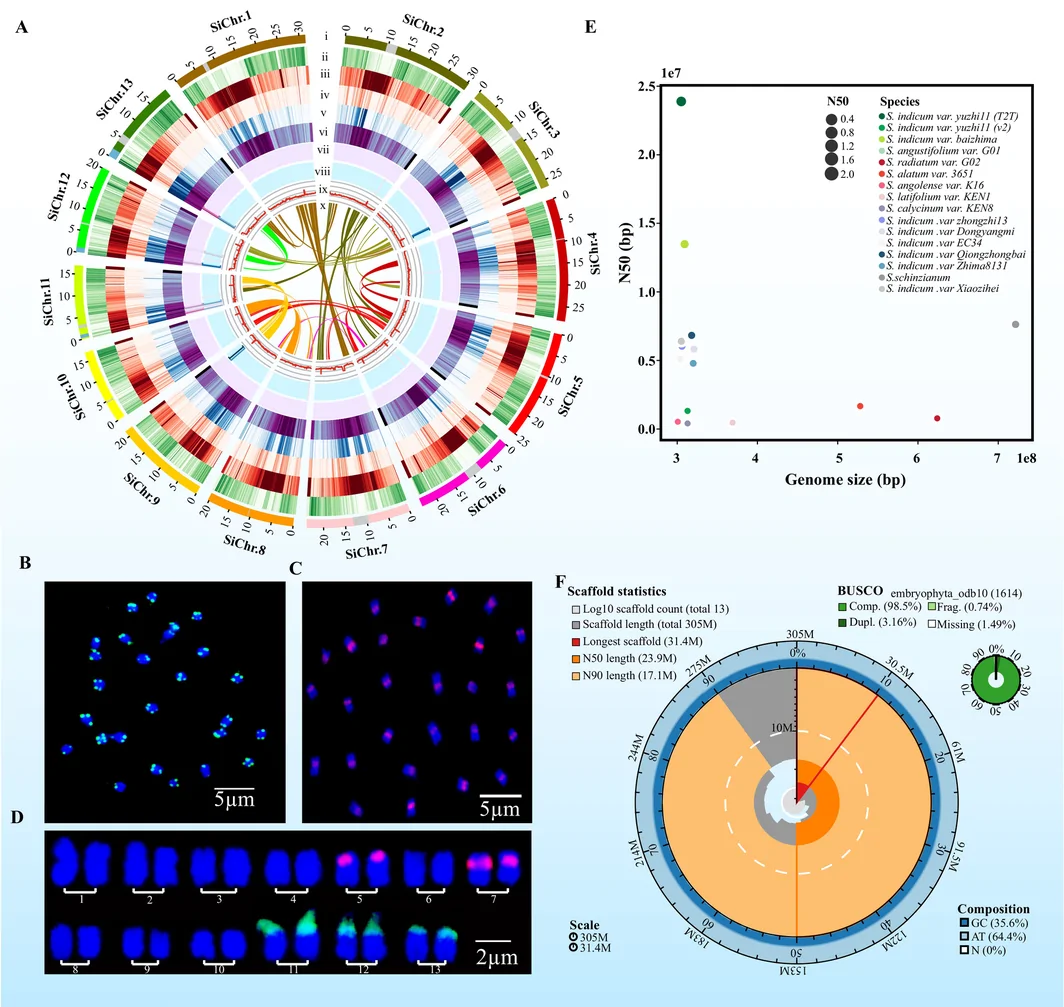
Summary of the T2T sesame genome assembly. (A) Landscape view of the T2T sesame genome. From outer to inner tracks: (i) Chromosome number and length; (ii) Gene density; (iii) Repeat density; (iv) LTR‐Copia element density; (v) LTR‐Gypsy element density; (vi) DNA methylation density; (vii) SNP density; (viii) Indel density; (ix) GC content, and (x) Syntenic blocks. (B) Fluorescence in situ hybridisation (FISH) of sesame chromosomes with telomere probe (in green). (C) Chromosome FISH with a centromere repeat probe (in red). (D) Chromosome FISH with 45S (green) and 5S rDNA (red) probes. (E) Comparative statistics of genome size and contig N50 across 16 published sesame genomes. Circle size represents contig N50. (F) Snail plot of the T2T sesame genome, showing genomic features and completeness assessment. The inset pie chart (green) presents BUSCO evaluation results.

Primary genome assembly with hifiasm (v0.18.2‐r467) produced 92 contigs with N50 of 20.09 Mb (Table S2). Hi‐C interaction data identified nine assembly gaps, which were subsequently closed using Nanopore ultra‐long reads. The final corrected T2T genome assembly (Figures 1A and S3) spans 305 Mb, achieving a contig N50 of 23.89 Mb (Table S3). Chromosome IDs and orientations were adjusted according to the sesame genome v3.0 (var. Yuzhi 11) (Miao et al. 2024). Fluorescence in situ hybridisation (FISH) confirmed that sesame contains 13 centromeres and 26 telomeres, with three chromosomes featuring a 45S rDNA cluster at one terminus (Figure 1B–D) and without three telomeres in assembled T2T genome (Table S4). The genome quality value (QV) ranged from 44.9 to 47.9. The assembly achieved 98.8% k‐mer completeness and a Long Terminal Repeat (LTR) assembly index (LAI) of 24.92 (Tables S3 and S5). Benchmarking Universal Single‐Copy Orthologs (BUSCO) analysis recovered 98.6% complete orthologs (Figure 1F; Table S5), supporting high completeness of the T2T genome. In addition, mapping coverage of HiFi and Nanopore ultra‐long reads was consistent across the genome (Figure S4). The comparison with published genome quality demonstrated that this T2T assembly represents the most accurate and complete sesame genome reported to date (Table S3).

### 2.2 Genome Annotation

A total of 31 063 protein‐coding genes were predicted in the T2T sesame genome, producing 42 781 transcripts (Figure 1A; Table S3). Notably, over 93% of annotated gene models are functionally supported (Table S6). The genome comprises 162 Mb repeat (53.11% of the total) (Table S7), with Transposable elements (TEs) constituting 52.5% (DNA transposons: 27.21; retrotransposons: 21.48%). Long terminal repeat (LTR) elements were dominated by LTR‐Gypsy (17.23%) and LTR‐Copia (7.4%) elements. Additionally, 5 637 noncoding RNAs (ncRNAs) were identified, including 120 miRNAs, 1 072 transfer RNAs (tRNAs), 4 089 ribosomal RNAs (rRNAs), and 356 small nuclear RNAs (snRNA) (Table S8).

The telomeric repeat motif (TTTAGGG/CCCTAAA) was conserved, with copy numbers ranging from 270 to 12 548 (Table S4). Due to extended long repetitive satellite DNA regions at the ends of the three satellite chromosomes (i.e., *Si*Chr. 11, 12 and 13), we identified 23 telomeres at the both ends of the 10 regular chromosomes and the opposing ends of the three satellite chromosomes (Figure S5). Remarkably, six telomeres exceeded 10 Kb in length, surpassing reported maximum telomere length in *Arabidopsis thaliana* (9 197) (Wang, Yang, et al. 2022) and *Ziziphus jujuba* (2 807) (Li et al. 2024). Thirteen centromeric regions per chromosome were defined by the 153 bp conserved centromere‐specific monomer *SiCEN1* (Miao et al. 2024) (Figures 1A and S5, Table S9), ranging from 42 651 bp to 3 511 084 bp, with a mean length of 1 352 928 bp, which is smaller than that of maize (2 216 500 bp) (Chen et al. 2023). Notably, 234 genes reside within centromeric regions and Gene Ontology (GO) enrichment analysis showed significant enrichment in ‘regulation of early endosome to late endosome transport’, ‘endosome organisation’ and ‘cytokinin biosynthetic process’ (Figure S6), consistent with findings in rice (Song et al. 2021). Collectively, these findings underscore the high completeness and annotation quality of the T2T sesame genome.

### 2.3 Genome Comparison Between the T2T Genome and *Sesamum indicum* Var. Baizhima

**FIGURE 2:**
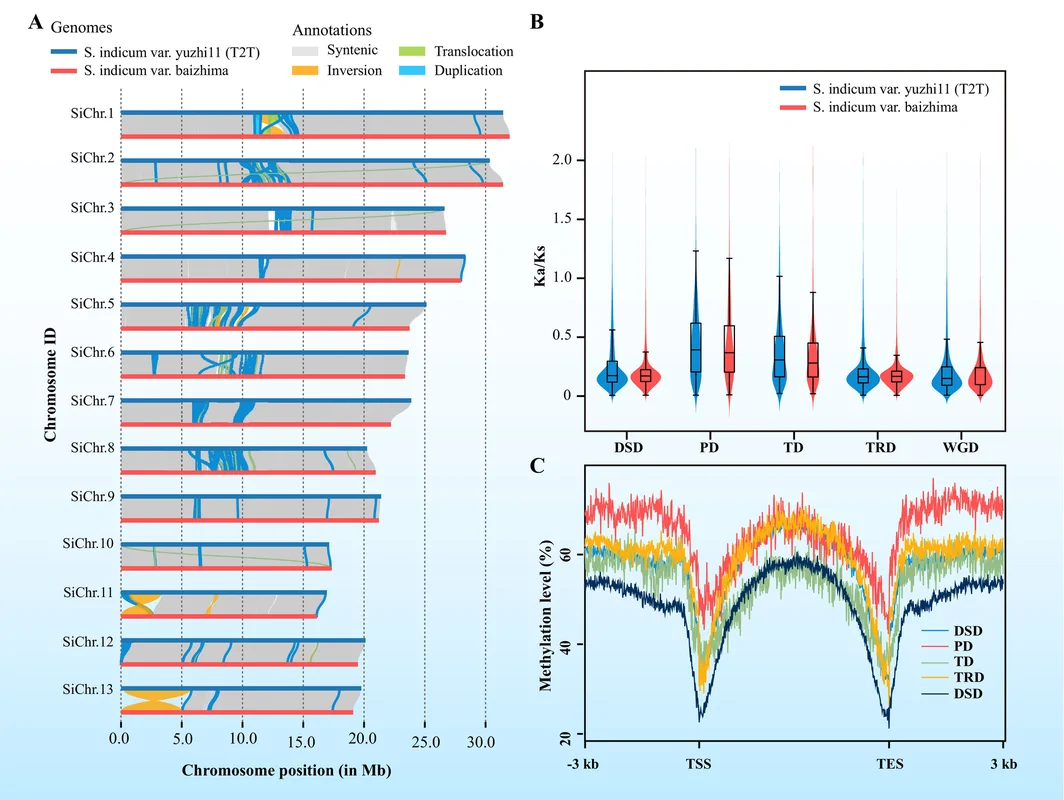
Comparative analysis of the T2T sesame genome and the reference genome of var. Baizhima. (A) Syntenic analysis of T2T genome (blue) and that of var. Baizhima (red). (B) Analysis of selection pressure (Ka/Ks ratio) among duplicated gene pairs in the T2T and Baizhima genomes. (C) Methylation levels of genes duplicated by different modes in T2T sesame genome. TD: Tandem duplication; PD: Proximal duplication; TRD: Transposed duplication; DSD: Dispersed duplication; WGD: Whole‐genome duplication.

To assess the improvements of the T2T genome, we performed a pairwise alignment against the published and available highest‐quality reference, *S. indicum* var. Baizhima genome (https://github.com/mummer4/mummer) (Figure 1E; Table S3). Whole‐genome comparison identified 2 765 structural variations (SVs), comprising 1 359 insertions, 1 251 deletions, 8 inversions, and 147 translocations (Figures 2A and S7). These SVs were primarily distributed on chromosomes SiChr.5 and SiChr.8 (Figure 2A). Most SVs (50 to 400 bp) reside in intergenic regions (Figure S7) and affect approximately 419 genes (Table S10). Notably, *SiIAA19*, a negative regulator of the auxin signalling and plant branching (Krishna Reddy and Finlayson 2014), is significantly upregulated in the single‐stem Yuzhi11 relative to the branched Baizhima. Gene Ontology (GO) enrichment of SV‐affected genes underscores roles in shoot and root development, and DNA integration‐key functions for plant growth and morphological differentiation. KEGG pathway analysis indicated significant enrichment in SNARE interactions in vesicular transport and pentose and glucuronate interconversions (Figure S8).

Whole‐genome duplication (WGD) and other duplication events are major drivers of genome evolution (Paterson et al. 2010; Soltis et al. 2009). We systematically investigated gene duplication patterns in both the T2T genome and the Baizhima genome (Table S11; Figure S9).

In the T2T genome of var. Yuzhi 11, we detected a total of 7 467 WGD, 1 074 tandem duplication (TD), 1 135 proximal duplication (PD), 6 706 transposed duplication (TRD), and 19 152 dispersed duplication (DSD) gene pairs. Ka, Ks, and Ka/Ks ratios were calculated for these pairs (Figure 2B; Table S12). Notably, PD and TD genes exhibited the highest positive selection rates (4.7% and 4.47%, respectively) (Figure 2B), and PD genes had the highest levels of methylation (Figure 2C). GO enrichment analysis of PD genes showed strong associations with defence response, response to other organisms, carbohydrate metabolic process (Figure S9), indicating adaptive evolution for environmental resilience.

### 2.4 Population Structure Analysis of a Core Sesame Population

**FIGURE 3:**
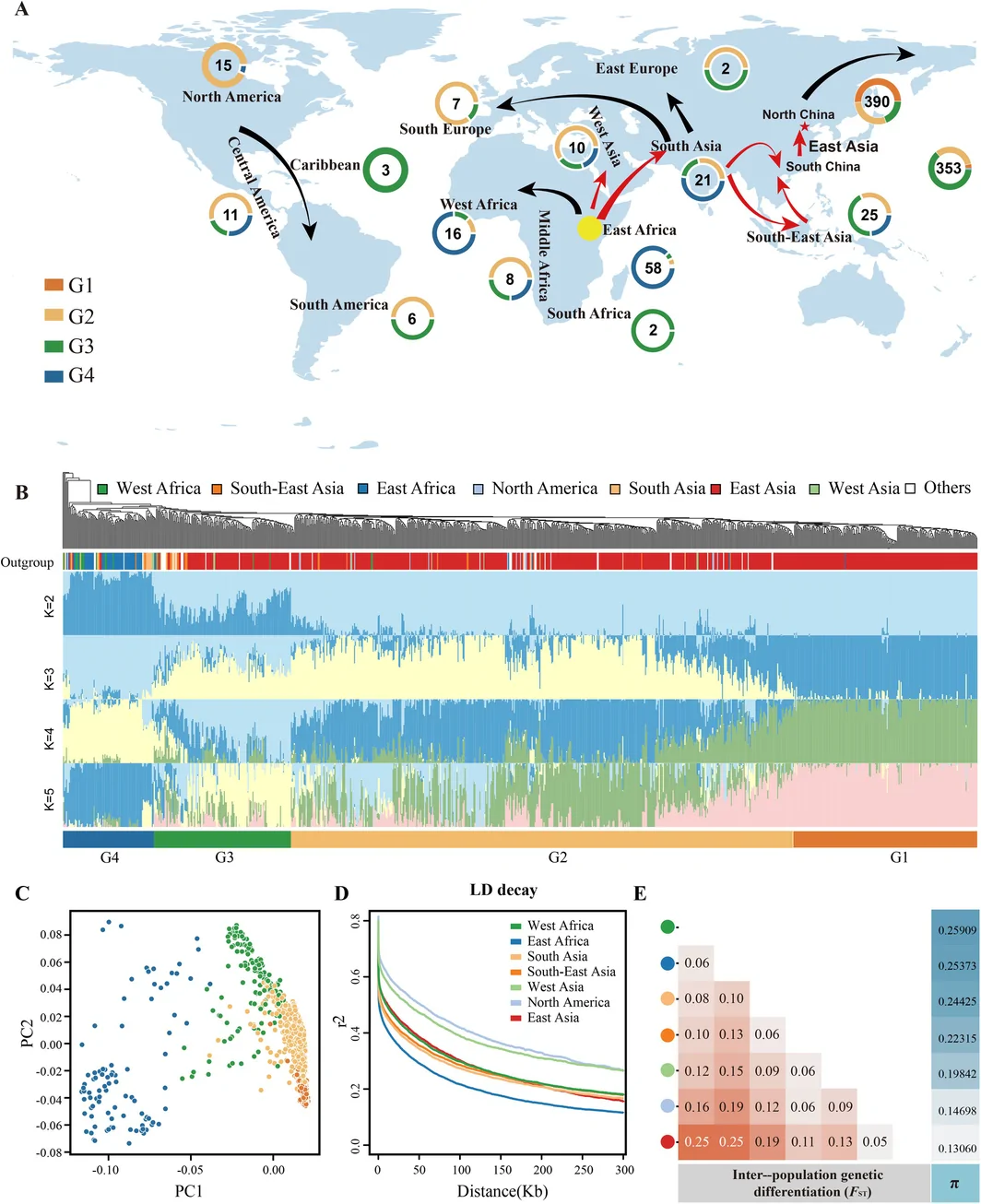
Phylogenic relationships and genetic structure of the core sesame population. (A) Geographic distribution of 927 sesame accessions and inferred dissemination routes. Fourteen pie charts represent geographic groups, with segment colours corresponding to population structure groups. Red arrows indicate the eastward spread of sesame from East Africa to Asia based on molecular evidence; black arrows represent hypothesised global dissemination routes. (B) Phylogenetic tree and population structure analysis with different cluster levels (*K* = 2, 3, 4, and 5, respectively). (C) Principal component analysis (PCA) of 927 sesame accessions showing the first two components. Dot colours correspond to the four groups identified in population structure analysis. (D) Linkage disequilibrium (LD) decay pattern for each of the seven geographic groups. (E) Inter‐population genetic differentiation (*F*_ST_) and average nucleotide diversity (π) of the seven geographic groups. Population group colours are consistent across all panels.

Sesame is one of the oldest cultivated oilseed crops, yet its origin and domestication remain subjects of ongoing debate. Complementing fossil records, analyses of genetic diversity and linkage disequilibrium (LD) decay may clarify sesame's evolutionary history. Genome re‐sequencing of 927 sesame accessions (Table S13) including 802 landraces and 125 cultivars, collected from 42 countries (Figure 3A), generated a total of 5.28 Tb clean reads, with an average genome coverage of 18.7× per accession. Using the T2T reference genome, we identified 2 732 061 high‐quality single‐nucleotide polymorphisms (SNPs). These SNPs were unevenly distributed across chromosomes (Figure S10A), with 42.31% located in intergenic regions and 6.65% within exons. A total of 18 314 genes were affected by non‐synonymous SNPs (Figure S10B).

We investigated geographic patterns of diversity across 927 accessions, which we assigned to 14 regions (Figure 3A). Phylogenetic, population structure, and PCA analyses (Figure 3B,C) consistently identified four main clades: G1 (predominantly North China), G2 (mixed North–South China), G3 (mixed South China and non‐China), and G4 (primarily African and South Asia). Model‐based clustering further supported these subdivisions, with *k* = 2 separating Chinese from non‐Chinese accessions and *k* = 4 delineating the four clades. Gene flow between South China and non‐Chinese populations appears limited, as only a small proportion of G1 accessions exhibit G2 ancestry. Collectively, these results suggest a gradual northward expansion of cultivated sesame within China, with South China acting as a key genetic bridge to non‐Chinese populations.

### 2.5 Geographic Origin and Transmission Routes of the Cultivated Sesame

To investigate the potential origin and migration routes of cultivated sesame, we analysed population genetics across seven major geographic groups (Figure 3E; Table S14). Pairwise *F*_ST_ values exceeded 0.05, indicating moderate differentiation among groups. Notably, with notably low *F*_ST_ values between West Africa and East Africa (*F*_ST_ = 0.06), South‐East Asia and South Asia (*F*_ST_ = 0.05), North America and South‐East Asia (*F*_ST_ = 0.06), West Asia and South‐East Asia (*F*_ST_ = 0.06), and East Asia and North America (*F*_ST_ = 0.05), implying close genetic relationships. Conversely, pronounced differentiation was observed between East Asia and both East and West Africa, with *F*_ST_ values reaching 0.25 (Figure 3E). This suggests significant genetic divergence, likely driven by strong selection pressures in East Asia related to environmental adaptation, such as differences in latitude, moisture, and temperature. Nucleotide diversity (π) was highest in West Africa, East Africa, and South Asia (greater than 0.2), indicating that these regions harbour rich genetic diversity and may have been key centres for sesame domestication and evolution (Figure 3E).

To further examine population structure, we assessed linkage disequilibrium (LD) decay and half‐decay distances across the genome in each population (Figure 3D). The most rapid LD decay was observed in the East Africa group, with a half‐decay distance of approximately 27 Kb, comparable to wild soybean ( *Glycine soja* Sieb. & Zucc.) (Zhou et al. 2015). Interestingly, the decline in LD of sesame was faster than that reported for common bean ( *Phaseolus vulgaris* L., 107 Kb) and foxtail millet ( *Setaria italica* , 100 Kb) (Jia et al. 2013), but slower than wild rice ( *Oryza rufipogon* , 20 Kb) (Huang et al. 2012) and wild maize ( *Zea mays ssp. parviglumis* , 22 Kb) (Hufford et al. 2012). Among the seven groups, East Africa, South Asia and South‐East Asia exhibited the most rapid LD decay, suggesting higher historical recombination rates and larger effective population sizes in these regions—further supporting their likely roles in sesame domestication and diffusion (Figure 3D).

**FIGURE 4:**
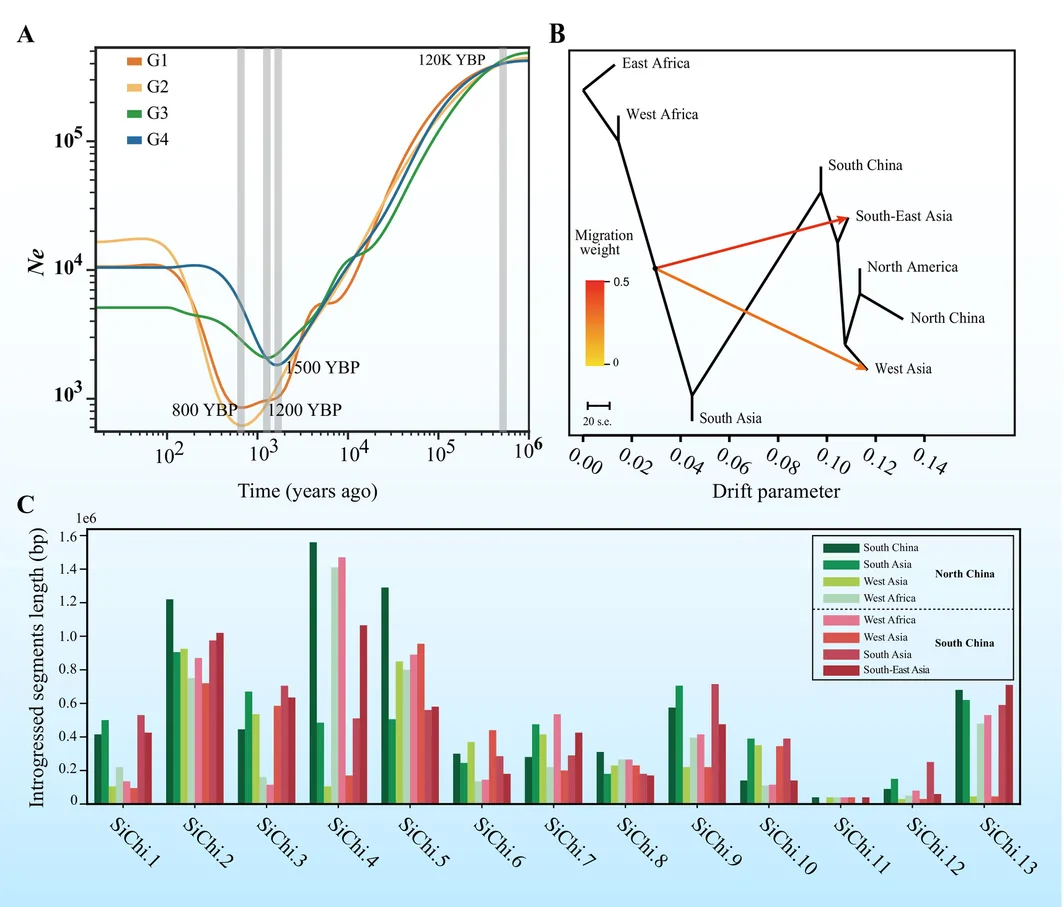
Historical demography and dissemination patterns of sesame. (A) Dynamics of effective population sizes (Ne) over time using SMC++. A generation time (g) of 1 year and a mutation rate (μ) of 6.95 × 10^−9^ per site per generation were applied. (B) Patterns of historical gene flow among seven sesame lineages, reconstructed using TreeMix. The maximum likelihood (ML) tree shows population relationships among the seven sesame lineages. Two inferred migration events are indicated by coloured arrows, with arrow weight reflecting migration intensity. The scale bar represents 10 times the average standard error. (C) Estimated proportions of introgressed genomic segments in North and South China sesame groups originating from South Asia, West Africa, South‐East Asia and West Asia.

To reconstruct the demographic history of cultivated sesame, we applied SMC++ to estimate the effective population size (Ne) and the divergence time among the four core population groups (G1—G4) (Figure 4A). Demographic modelling (SMC++) identified a severe bottleneck 120 K years before present (YBP) across four core groups (G1—G4), with subsequent recoveries occurring earlier in Asia populations (800–1500 YBP) compared to African groups (Figure 4A). Divergence timing suggested an initial split between African and South Asia populations, followed by more recent separations among Asia groups, with the North China and North America split being the most recent (Figure 4B).

To examine migration patterns, we analysed the gene flow among the core sesame populations using TreeMix (v1.15.3). The relationship between the number of migration edges (m) and model fit (log likelihood) was evaluated to determine the optimal level of gene flow events in the evolutionary history of sesame. The model selection curve (Figure S11) exhibits a characteristic saturation pattern that supports *m* = 2 as the most appropriate number of migration edges for subsequent analysis. The analysis revealed two significant gene flow events from African populations into South‐East and West Asia populations (Figure 4B). Additional evidence supported strong gene flow between West and East African groups (Figure S12).

Based on the f‐statistics analysis of admixtools tool (Table S15), Treemix result (Figure S12) and appeal results, we elucidated the evolutionary history and dispersal routes of sesame ( *Sesamum indicum* L.) (Figure 3A). Our results support an East Africa origin of cultivated sesame, with subsequent westward gene flow to West Africa. From Africa, sesame dispersed eastward across the Indian Ocean to South Asia, which represents the basal population of Asian sesame. We identified two independent centers of diversity in Asia: (i) a western center in West Asia that originated directly from East Africa via the Red Sea/Arabian Peninsula route, and (ii) an eastern center involving South Asia, South‐East Asia, and East Asia. Notably, the South China population exhibits a dual genetic composition, deriving primarily from South Asia and South‐East Asia. In contrast, the North China population originated unambiguously from South China without significant admixture, indicating a northward expansion of sesame cultivation. These findings reveal a complex domestication and dispersal history of sesame, characterised by parallel transcontinental expansions and region‐specific admixture events.

To quantify genome‐wide introgression, we performed *f*d‐statistic analysis using the ABBABABAwindows tool, and calculated the proportion of genome introgression among the North and South China populations, as well as from South Asia, South‐East Asia, West Asia, and West Africa (Figure 4C). Notable introgression was observed from West Asia, South Asia, South‐East Asia, and West Africa into Chinese populations (Figure S13). In South China, the largest proportion of genome introgression originated from South Asia (5.98 Mb, 1.96%), followed by South‐East Asia (5.93 Mb, 1.94%) and West Africa (5.61 Mb, 1.84%) and West Asia (4.08 Mb, 1.34%) (Figure 4C). North China harboured 7.35 Mb (2.41%) of introgressed segments from South China, supporting the hypothesis that northern populations are derived from southern sources.

Chromosomal‐level analysis showed the most extensive introgression into North China originated from South China and South Asia, particularly on *Si*Chi4, where a segment from South China exceeded 1.56 Mb (Figure 4C). Additional introgressed segments from South Asia were located on chromosomes 1, 3, 9, 10, 12, and 13, whereas introgression from West Asia and West Africa was less frequent (Figures 4C and S13). Interestingly, introgressed fragments from West Africa were predominantly observed on chromosome 4 (Figures 4C and S13).

We performed GO and KEGG enrichment analyses to elucidate the functional significance of introgressed genes. In North China, West Africa‐derived genes were enriched in defence response, response to other organism, and immune‐related processes (Figures S14A and S16A) and pathways involved in nucleotide metabolism and sesquiterpenoid and triterpenoid biosynthesis (Figures S14B and S16B), indicating early selection for stress resistance. In contrast, genes introgressed into South China from South Asia were enriched in carbohydrate metabolic process, nucleobase‐containing compound metabolic process, glutathione metabolic process, and ubiquinone biosynthetic process (Figures S15 and S17). These pathways point to the potential medicinal value of sesame as a significant factor in the selection of traits in the South China population. Collectively, these findings suggest that gene flow from neighbouring regions contributed to the formation of genetically diverse sesame populations with enhanced adaptability, and that regional selection pressures shaped specific traits related to defence and metabolism during sesame's global dissemination.

### 2.6 Evolution Dynamics of Flowering Time and Regulatory Genes in Sesame

**FIGURE 5:**
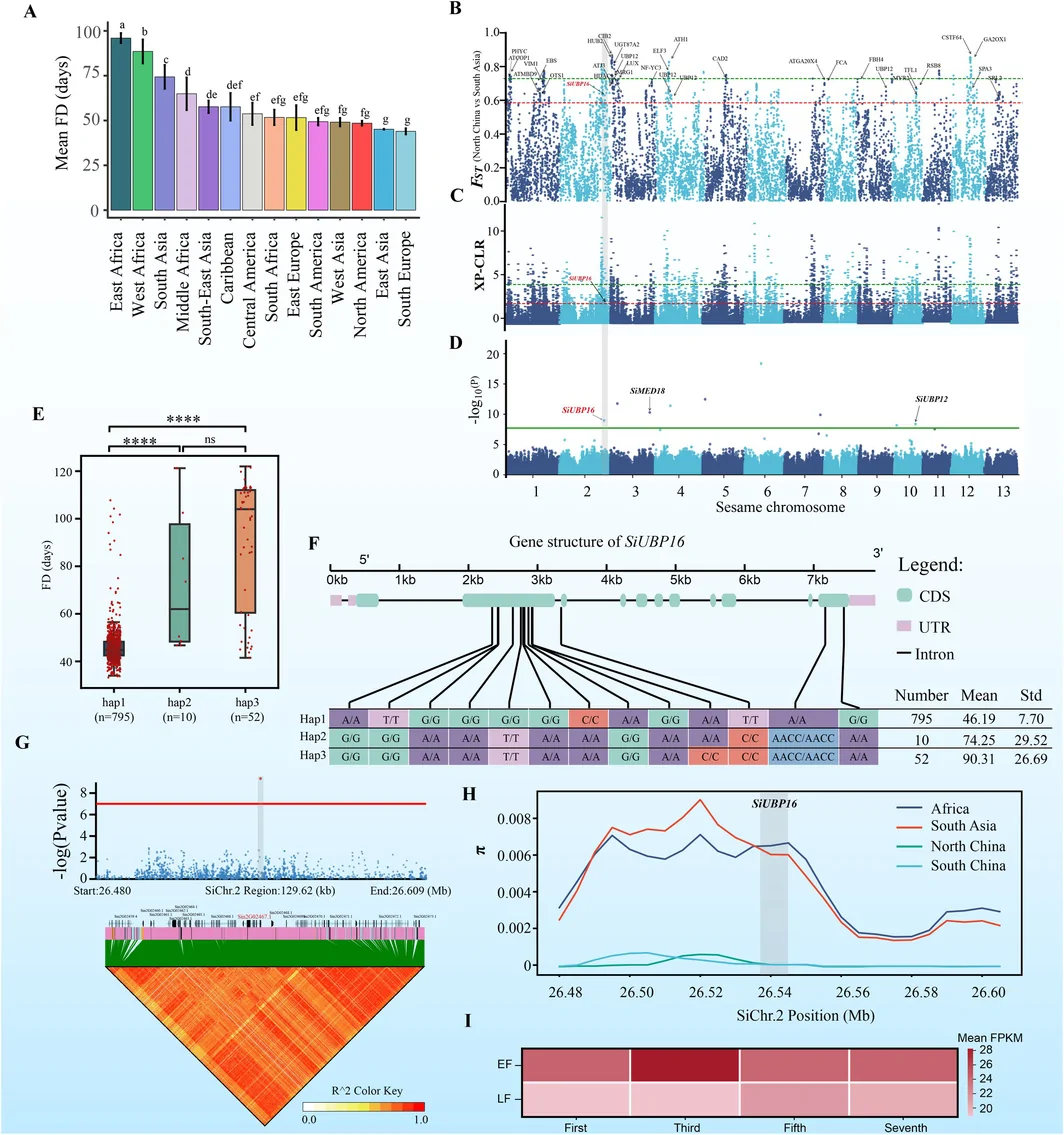
Identification of flowering time‐associated genes in sesame. (A) Comparison of mean flowering time among sesame accessions from 14 different geographic regions. (B and C) *F*_ST_ and XP‐CLR distribution between 90 sesame accessions from North China and South Asia groups, estimated using 20 Kb sliding windows with 5 Kb steps. (D) Manhattan plot for GWAS results for DF trait in Yuanyang (2022). The green line indicates the significance threshold (‐log_10_Pvalue = 7.7; Bonferroni correction). Three candidate genes, *SiUBP16* (Sin2G02467), *SiUBP12* (Sin10G01117) and *SiMED18* (Sin3G02274), were identified. (E) Boxplots of DF values for three *SiUBP16* haplotypes. Statistical significance was evaluated using a two‐tailed Student's *t*‐test. * indicates *p* < 0.01, ** for < 0.001, *** for < 0.0001, and **** for < 0.00001. (F) Gene structure and mutation sites of *SiUBP16*. (G) Linkage disequilibrium (LD) heatmap for a 120 Kb genomic region surrounding lead SNPs on SiChr.2. (H) Sliding‐window plot of nucleotide diversity (π) among population groups. (I) The expression of *SiUBP16* in the shoot apex was analysed at distinct developmental stages: The emergence of one, three, five, and seven pairs of true leaves, respectively. EF: Early flower sesame accession; LF: Late flower sesame accession.

Flowering time is a key agronomic trait in sesame, influencing environmental adaptation and yield potential. As a short‐day crop native to tropical regions, sesame's flowering behaviour is sensitive to photoperiod and temperature signals (Andrés and Coupland 2012; Khan et al. 2014; Leijten et al. 2018). In our study, the DF of the 927 sesame accessions showed wide variation and was strongly correlated with geographic origin (Figure 5A; Table S16). Notably, many accessions from South Asia and Africa displayed significantly delayed flowering or vegetative growth when cultivated in high‐latitude environments like Yuanyang, North China. For instance, accession S93 from East Africa flowered at 34–35 days in Sanya (latitude = 18°25′ N) but required 91–120 days in Yuanyang (latitude = 35°07′ N: Table S16). Heritability analysis among the 927 accessions showed that the flowering time trait was controlled by genotype, as the ratio of V(g) and V(e) varied from 0.93 to 0.99 under the four environments (Table S17).

Thus, to investigate the evolution of flowering time and the potential dissemination routes of Chinese sesame from South Asia, we compared 69 accessions from North China with 21 accessions from South Asia using *F*_ST_ and XP‐CLR analyses (Figure 5B,C, Table S18). We identified 200 genomic common regions (top 5% *F*_ST_ and XP‐CLR) harbouring 1 825 genes that showed strong divergence between the two groups (Table S18). Of these, 27 genes were homologues of flowering‐related genes in Arabidopsis, based on the Flowering Interactive Database (Table S19). This finding suggests that during the gradual northward dispersal of sesame from South Asia through South China to higher latitudes, genes regulating flowering time underwent reshaping to adapt to the prolonged daylight conditions at elevated altitudes. GO and KEGG enrichment analysis indicated that these candidate genes were under selection and enriched in biological pathways such as response to salt stress, cellulose biosynthetic process, plant organ development, pigment biosynthetic process, and Isoquinoline Alkaloid Biosynthesis (Figure S18).

Additionally, we conducted genome‐wide association study (GWAS) for DF across 4 years in 235 sesame accessions grown at the Yuanyang experimental station (Figures 5C and S19, Table S20). In the 2022 dataset, 9 genomic regions were significantly associated with DF (−log_10_Pvalue≥ 7.72), encompassing 146 genes (Figures 5C and S19). Notably, the candidate gene *SiUBP16* (*Sin2G02467*) was significantly associated with DF across two environments, explaining up to 7.6% of phenotypic variance. This gene also showed a strong positive selection signal (Figure 5D,G, Tables S18 and S20). Two additional flowering‐related candidate genes *SiMED18* (*Sin3G02274*) and *SiUBP12* (*Sin10G01117*) were also identified, which were both homologous to Arabidopsis genes involved in flowering regulation (Table S20).

To elucidate the functional impact of allelic variation in *SiUBP16* (*Sin2G02467*) on flowering time, we performed genotype–phenotype association analysis across 927 sesame accessions (Figure 5E). A total of 13 non‐synonymous mutations (SNPs) and a non‐frameshift insertion (InDels) were identified in the coding regions of *SiUBP16* (*Sin2G02467*), resulting in three distinct haplotypes. Under the high‐latitude Yuanyang environment, accessions carrying hap1 genotype (*n* = 795) exhibited an early flowering phenotype (EDF, mean DF = 46d), whereas accessions carrying hap3 (*n* = 52), mainly of South Asian origin, showed significantly delayed flowering (mean DF = 90d) (Figure 5E). *SiUBP16* resides in a region with strong linkage disequilibrium (Figure 5F) and high selection pressure (Figure 5G) on chromosome *Si*Chr. 2, indicating a genomic region with low recombination frequency and evolutionary conservation. Gene flow analysis suggested that *SiUBP16* was introduced from South Asia (Table S21). Additionally, we examined transcript accumulation in shoot apex throughout pre‐flowering developmental stages. Our analysis revealed that *SiUBP16* expression was markedly higher in early‐flowering lines than in late‐flowering lines (Figure 5I).

Using a hidden Markov model (HMM) search of the *S. indicum* var. Yuzhi11 T2T genome, we identified six full‐length UBP genes that retain the zinc‐finger ubiquitin binding domain (ZnF UBP) (Table S22). Phylogenetic analysis of these enzymes with 27 UBP genes from *A. thaliana* determined that *Sin2G02467* might be a possible homologue of UBP16 (Figure S20A). To further classify genes containing the ZnF UBP domain, we screened UBP genes among the 26 other species (Table S22). Phylogenetic analysis showed that the ZnF UBP gene family was divided into four subfamilies (Figure S20B).

Protein domain annotation using InterProScan (https://www.ebi.ac.uk/jdispatcher/pfa/iprscan5) identified a UCH (ubiquitin C‐terminal hydrolase) and a zf‐MYND (zinc finger MYND‐type) domain in SiUBP16 (Figure S21). The UCH domain classifies SiUBP16 as a deubiquitination enzyme (DUB), which functions by releasing covalently linked ubiquitin (Ub) and played an essential role in recycling Ub and reversing the action of Ub conjugation (Yang et al. 2007; Mevissen and Komander 2017). Given the presence of the zf‐MYND domain, known for recognising PXLXP motifs, we hypothesised that SiUBP16 may regulate flowering by targeting proteins involved in floral timing. Orthology analysis identified 40 sesame genes with PXLXP motifs that are homologous to flowering‐time regulatory genes in *Arabidopsis* (Table S23).

**FIGURE 6:**
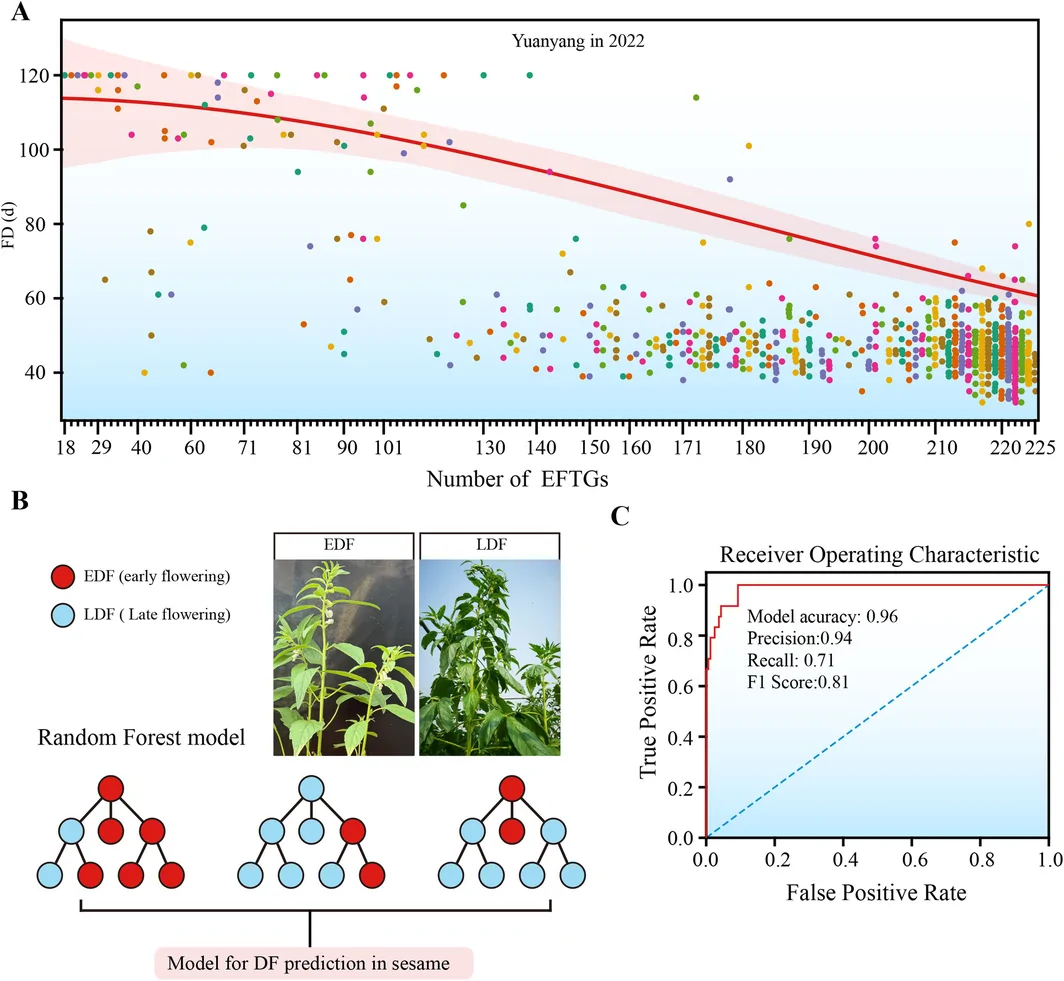
Characterisation of early flowering genes and construction of a flowering time prediction model in sesame. (A) Relationship between the number of early flowering‐related gene alleles and flowering time under Yuanyang environment in 2022. (B) Schematic workflow for constructing genotype‐based prediction models for DF phenotype at high latitude. (C) Performance evaluation of the random forest‐based flowering time prediction model. Metrics include model accuracy, receiver operating characteristic (ROC) curve, and F1 score.

To explore broader genotype–phenotype associations, we examined 175 non‐synonymous SNPs across the 40 PXLXP‐related genes and three GWAS‐identified candidate genes (including *SiUBP16*, *SiMED18* and *SiUBP12*) (Figure 6). Transcriptome data revealed that most of these genes are highly expressed in root, with *SiUBP16* exhibiting minimal expression in pod and leaf (Figure S22). Across the 927 sesame accessions, we identified seven genotypes harbouring 225 allelic sites associated with early flowering (mean DF = 48 days in Yuanyang). These were designated as early DF genotype alleles (EDGs). A negative correlation was observed between the number of EDGs and flowering time: the greater the number of polymerised EDGs, the earlier the flowering (Figures 6A and S23). To translate these insights into predictive tools, we constructed a random forest model to predict DF type in high‐latitude environments based on genotypic data (Figure 6B). The model achieved an overall accuracy of 0.96, with an ROC value of 0.99 and an F1‐score of 0.81, indicating high predictive power (Figure 6C).

## 3 Discussion

High‐quality reference genomes serve as invaluable genomic resources, enabling deep insights into population evolution and domestication, as well as gene function and molecular breeding studies. In this study, we report the first T2T reference genome of sesame (var. Yuzhi 11) and provide comprehensive population genomic analysis that clarify its evolutionary history and support East Africa as the center of origin. This genomic resource lays the foundation for future gene discovery and breeding programs focused on improving sesame varieties to meet the demands of climate changes and diverse market demands. The inclusion of telomere and centromere sequences in our assembly revealed a set of 234 centromere‐specific genes that were completely absent or fragmented in the Baizhima and other 2023 assemblies. Functional enrichment analysis of these genes uncovered pathways related to regulation of early endosome to late endosome transport, endosome organisation and cytokinin biosynthetic process—insights that were unattainable in previous studies due to missing centromeric data. Future work will focus on integrating population variation data to elucidate the functional importance of centromeric variations, thereby extending the insights provided by Song et al. (2023) and Wang et al. (2023).

Although fifteen genome assemblies of cultivated and wild sesame species have been published (Miao et al. 2024; Song et al. 2023; Wang et al. 2014, 2023; Wang, Huang, et al. 2022; Zhang et al. 2021), the precise origin of cultivated sesame has remained unresolved. Archaeological and historical records have traditionally supported South Asia as the domestication center, primarily due to the earliest carbonised seeds discovered at the PKT site in eastern India and Harappan sites in Pakistan (Bedigian 2004; Pokharia et al. 2011). Additionally, the wild subspecies *Sesamum indicum* subsp. *Malabaricum*, which closely resembles modern cultivars both morphologically and chemically (sesamin and sesamolin content), is native to South Asia (Bedigian 2015). While previous SSR‐based studies suggested higher nucleotide diversity in South Asia sesame (Dossa et al. 2016), our comprehensive genomic analysis reveals a contrasting pattern: East African accessions exhibit significantly higher genetic diversity and occupy basal positions in phylogenetic trees, indicating that Africa harbours a richer reservoir of wild genetic resources (Bedigian 2003; Dossa et al. 2016). This is further supported by the greater number of unique wild Sesamum species in Africa (18 species) compared to South Asia (8 species, with only 5 endemics) (Kafiriti and Mponda 2010). Consequently, our data suggest that Africa may have been a critical center of diversification, if not the primary domestication site. The genetic structure revealed by our dataset mirrors earlier SSR and isozyme studies, highlighting pronounced differentiation between African and Asia germplasm (Dossa et al. 2016). Our findings reveal sesame's domestication as a dynamic process intertwined with early *Homo sapiens* migration. The genetic signatures support a southward diffusion hypothesis, where early humans, traversing emerging ecological corridors, facilitated sesame's spread from Africa to South Asia. This movement likely coincided with climatic shifts and population bottlenecks around 120 000 years ago, aligning with the Southern Dispersal Route model (Ao et al. 2024; Reyes‐Centeno et al. 2014, 2015). Archaeological evidence from Harappan sites further confirms sesame's early integration into South Asia agriculture, highlighting the co‐evolution of human migration and crop dispersal. However, the exact timing and location of domestication events remain ambiguous without ancient DNA evidence. Future research should integrate broader sampling of wild relatives and advanced population genomic methods to disentangle these complex evolutionary histories.

In this study, to determine the sesame origin position, we extended population genomic analysis with a large germplasm population. Consistent with these previous reports, our analysis supports that Africa is a basal position in the sesame phylogeny and there is a northward dispersal pattern from low‐ to high‐latitude regions within China (Song et al. 2022, 2023). Moreover, the expanded global germplasm collection (927 accessions) used in this study enabled a finer‐scale resolution of population structure and admixture patterns that were not apparent in the previously published datasets. Especially, we detected ancestral components from South Asia and South‐East Asia as the main components in South China germplasm. The results suggested there is a more complex historical gene flow between these regions than previously recognised. We thus interpret these patterns as population genomic inferences rather than definitive historical conclusions.

In addition, our broader continental‐scale sampling revealed a substantial genetic differentiation among major geographic groups, with *F*_ST_ values of 0.25 between East Asia and Africa and 0.05 between East Asia and North America, which would likely be a result of long‐term geographic isolation following initial dispersal events. In our study, the denser African sampling supported that ‘African origin’ hypothesis proposed by some early studies (Bedigian 2013; Kobayashi 1991; Ekta Sharma et al. 2014; Weiss 1983). The results suggested that East Africa is a major center of early sesame diversification, which thereby refines the region of the ‘African origin’ hypothesis. We believe that expanding African accessions sampling eliminates some limits from the sampling framework and resolves fine‐scale evolutionary patterns with comprehensive international germplasm collections.

Furthermore, East Africa is widely recognised as a major domestication and diversification hotspot for several other staple and economically important crops. This region is the cradle of *Coffea arabica* domestication (Salojärvi et al. 2024; Vavilov and Dorofeev 1992), the primary center of origin for *Sorghum bicolor* (De Wet and Harlan 1971), and the sole origin of *Eragrostis tef* (Vavilov and Dorofeev 1992). Additional crops such as *Guizotia abyssinica* (Vavilov and Dorofeev 1992) and *Eleusine coracana* (Vavilov and Dorofeev 1992) also trace their origins to this region. The concentration of these diverse origin events underscores the unique ecological and evolutionary conditions that have historically made East Africa a fertile ground for crop evolution, suggesting the possibility of sesame's origin.

Gene flow plays a critical role in plant adaptation and diversification, enabling populations to incorporate beneficial alleles from divergent lineages (Diop et al. 2022; Tigano and Friesen 2016). Our genome‐wide introgression analysis revealed that modern Chinese sesame varieties are mosaics of multiple genetic origins, with the largest contributions from South Asia (1.96%), followed by South‐East Asia (1.94%), West Africa (1.84%) and West Asia (1.34%). These results suggest historical migration and secondary domestication events, with local adaptation shaping population‐specific traits. Interestingly, varieties closer to the center of origin harboured lower levels of external introgression, consistent with the model of progressive selection during dissemination.

The genetic evidence strongly supports South Asia, particularly India, as the primary source of sesame introduced into China, a conclusion consistent with prior morphological and historical analyses (Nayar and Mehra 1970). Population demographic modelling revealed a major bottleneck in African, Indian, and South China populations, with recovery beginning approximately 1500 years ago. The Chinese accessions (G3) diverged from the Africa sesame accessions (G4) around 1200 years ago, followed by expansion in the Chinese groups (G1 and G2) ~800 years ago (Figure 4A). Supporting this timeline, sesame remains have been excavated from several archaeological sites in the Mediterranean, including Clonia, Pompeii, and Thessaloniki, dating back to the late 1st century BC. However, these finds suggest sesame was an exotic or luxury commodity, evidenced by the small quantities recovered at harbour trade sites (Margaritis 2014; Zech‐Matterne et al. 2015). These findings align with the global maritime exchange surge known as the ‘Age of Sail’, highlighting the role of ancient trade in the crop's global distribution.

Archaeological and historical evidence suggests that the global spread of sesame was significantly influenced by early trade networks, especially the Maritime Silk Road, which enabled its exchange across continents. Remnants of sesame has been recovered from coastal China dating back more than 2800 years, possibly to Neolithic contexts (Qiu et al. 2012). Our findings suggest that sesame may have accompanied the migration of *H. sapiens* out of Africa approximately 120 000 years ago, although its population size likely remained limited due to subsistence‐level agriculture and low dietary prioritisation. By the late Middle Ages around 1200 years ago, improvements in agricultural practices and the flourishing of maritime trade during the late Middle Ages likely catalysed the expansion and cultivation of sesame. Increased crop demand and trade routes enhanced both the population size and geographic spread of sesame, leading to adaptive selection and diversification. Sesame varieties from North China likely descended from those in South China, gradually adapting to different photoperiod and temperature, consistent with earlier hypothesis (Dossou et al. 2023). This trajectory illustrates the tight interdependence between agriculture, trade, and societal progress.

Early flowering time is a critical trait for crop adaptation, essential for high yield and adaptability, particularly in regions with short growing seasons (Mühleisen et al. 2014). Numerous environmental factors, including photoperiod (Song et al. 2015), light intensity (Yang et al. 2020), temperature changes (Jagadish et al. 2016), vernalisation (Amasino 2005), drought (Riboni et al. 2013), and salinity (Li et al. 2007), regulate flowering time, often through conserved regulatory networks. The mechanisms involved in flowering regulation have been extensively studied, particularly in *Arabidopsis thaliana* , where many regulatory genes have been identified (Maple et al. 2024; Mouradov et al. 2002). Specifically, the Nup96‐HOS1 complex in *A. thaliana* , located in the nuclear membrane, acts as a repressive module that regulates the levels of CONSTANS (CO) proteins and prevents precocious flowering under long‐day conditions. Mutations in any of these components contribute to increased accumulation of CO proteins (Cheng et al. 2020). Moreover, GIGANTEA (GI) and the FLAVINBINDING, KELCH REPEAT, F BOX protein 1 (FKF1) form a complex that mediates the degradation of CYCLING DOF FACTOR 1 (CDF1), a key repressor of CO. Increased CO protein levels subsequently elevate the expression of the FT gene and promote earlier flowering.

Adaptation to photoperiod and latitude has played a major role in shaping sesame flowering behaviour, but the mechanisms of flowering time regulation remain poorly understood. Early‐flowering cultivars initiate reproductive growth 30–40 days after sowing, whereas late‐flowering varieties may flower 70 to 80 days after sowing (Gadri et al. 2020; Langham 2007). When grown at high latitudes, near‐equatorial varieties tend to flower late (Table S16), while high‐latitude‐adapted varieties flower early in lower latitude regions. Previous studies indicated that sesame flowering is controlled by photoperiod sensitivity and multiple genetic loci (Kotecha et al. 1975), but the precise regulatory pathways remain unresolved.

Multi‐year GWAS analyses with *F*_ST_‐based divergence scans between South Asian and North Chinese populations (Figure 5) identified a novel flowering time regulator, *SiUBP16*. *SiUBP16* encodes a ubiquitin‐specific protease harbouring a zf‐MYND domain, which is known to recognise the PXLXP motif and mediate protein–protein interactions (Ansieau and Leutz 2002; Wu et al. 2022). While the molecular mechanism underlying *SiUBP16* function requires further investigation, the observed expression pattern suggests potential involvement in flowering time regulation. Protein–protein interactions and downstream signalling pathways involving SiUBP16 will be investigated in future studies.

## 4 Conclusion

In conclusion, we report the first high‐quality telomere‐to‐telomere (T2T) assembly of the cultivated sesame genome (var. Yuzhi 11). Population genomic analyses trace sesame's origin to East Africa and map its dispersal route through South and Southeast Asia to South China, followed by subsequent northward expansion. Additionally, we acknowledge that modern population structure inevitably carries signals of both ancient and more recent (e.g., breeding‐mediated) gene flow, which cannot be fully disentangled without ancient DNA evidence. Therefore, the proposed dissemination map should be regarded as a genome‐inferred hypothesis, and future studies incorporating ancient sesame DNA will be necessary to more precisely distinguish ancient admixture events from recent introgression. Notably, we identified a suite of flowering‐time genes, with *SiUBP16* emerging as a prime candidate that underwent strong selective sweeps during the migration from South Asia to North China. However, our findings are constrained by a sampling bias towards Chinese germplasm and limited representation from East Africa and South Asia, as well as the lack of functional validation for the candidate genes. Future work should prioritise the collection of ancient and wild sesame germplasm, particularly from East Africa, and employ CRISPR‐Cas9 to functionally validate *SiUBP16* and other flowering‐time regulators in vivo. By integrating multi‐omics data with functional genomics, we aim to unravel the genetic architecture underlying flowering time and accelerate the breeding of climate‐resilient sesame varieties.

## 5 Methods

### 5.1 Plant Materials

A well‐known Chinese sesame cultivar, Yuzhi 11 ( *S. indicum* , 2*n* = 26), was selected for T2T genome assembly. A core collection of 927 diverse sesame germplasm accessions, sourced from 42 countries, containing 802 landraces and 125 cultivars (Table S13), was used for genome resequencing, population structure, genetic diversity, and evolutionary analyses. Genome data for five wild sesame accessions, obtained from previous studies (Miao et al. 2024), were included as outgroups. All germplasm materials are publicly available from the sesame germplasm collection at Henan Sesame Research Center, Henan Academy of Agricultural Sciences, China. In the construction of the phylogenetic tree and genetic structure analysis, we focus on macro‐level geographic distribution patterns; therefore, China (730), North Korea (2), Korea (3), and Japan (9) materials are grouped as the ‘East Asia.’ In contrast, when investigating gene flow and genetic diversity, we focus on intra‐country geographic differences and subdivide Chinese materials into ‘South China’ and ‘North China’ to elucidate the genetic exchange between these regions. Geographic groups containing fewer than 10 accessions were excluded from quantitative population genetic analysis, including *F*_ST_, nucleotide diversity, and gene flow analyses.

All accessions were cultivated at Yuanyang (113°97′ E, 35°05′ N: in 2019, 2020, 2021 and 2022) and Sanya (109°50′ E, 18°25′ N: in 2018, 2019, 2021 and 2022) experimental stations for phenotypic evaluation of flowering time, measured as the number of days from sowing to flowering (DF).

### 5.2 DNA and RNA Extraction and Whole Genome Sequencing

High‐quality genomic DNA (gDNA) was extracted from fresh leaves of Yuzhi11 using the cetyltrimethylammonium bromide (CTAB) method (Stefanova et al. 2013). DNA quality was assessed using a NanoDrop One spectrophotometer (NanoDrop Technologies, Wilmington, DE, USA) and a Qubit 3.0 Fluorometer (Life Technologies, Carlsbad, CA, USA). Total RNA was isolated from root, stem, leaf, flower, and capsule tissues of Yuzhi11 following the TRIzol protocol, and RNA integrity was evaluated with an Agilent 2100 Bioanalyzer (Agilent Technologies, Palo Alto, California, USA).

We employed a multi‐platform strategy to generate a high‐quality genome assembly for Yuzhi11 (Figure S1). Briefly, short‐insert (~300 bp) paired‐end (PE) libraries were constructed from young leaf DNA and sequenced on the DNBSEQ‐T7 platform. For long‐read sequencing, we prepared a 15 Kb HiFi SMRTbell library (Saga Science, MA, USA), and sequenced it on the PacBio Revio platform (Pacific Biosciences, USA). Ultra‐long Nanopore libraries (≥ 100 kb inserts) were generated using the SageHLS HMW library system (Sage Science, USA) and sequenced on a PromethION device (ONT, R9.4 flow cells). Additionally, high‐throughput Hi‐C libraries were prepared and sequenced on the DNBSEQ‐T7 platform to capture chromatin spatial organisation (Figure S1 and Table S1).

To examine *SiUBP16* expression patterns during the transition to flowering, transcriptome sequencing was performed on shoot apical meristems (SAMs) from the early‐flowering line S142 and the late‐flowering line S792. Plants were grown in controlled growth chambers at 29°C temperature, 65% humidity, 15 h/9 h light/dark cycle and a light intensity of 120 μmol m^−2^ s^−1^. To account for developmental asynchrony between genotypes, SAMs were collected based on the number of expanded true leaves (1, 3, 5, and 7 pairs) rather than physiological age. Three biological replicates were prepared for each different stage of development of SAMs. Samples were flash‐frozen in liquid nitrogen immediately after collection and used to perform RNA_seq.

### 5.3 Genome Assembly and Quality Assessment

For T2T sesame genome assembly, pbccs read consensus tools (v6.0.0, Smartlink v10.1.0.119588) were utilised with parameters (−min‐passes 3 –min‐snr 2.5 –top‐passes 60). Approximately 30.3 Gb high‐quality consensus HiFi reads (~98.7 × genome coverage) were produced for assembly. Primary genome assembly was performed with hifiasm (v0.18.2‐r467, https://github.com/chhylp123/hifiasm) using default settings. To remove non‐nuclear genomic sequences, the assembled contigs were aligned against known mitochondrion, chloroplast, and contaminant genome sequences using minimap2 (v2.17‐r941, https://github.com/lh3/minimap2) with the ‐x asm5 parameter. Contigs with < 50% alignment coverage were retained as the core nuclear genome.

Approximately 38 Gb of Hi‐C reads, representing ~125.2 × genome coverage, were generated using the DNBSEQ‐T7 platform and employed to assemble a chromosome‐scale sesame genome. The ALLHIC (v0.9.8, https://github.com/tanghaibao/allhic) pipeline was used to cluster and anchor contigs with default parameters. Manual correction of contig orientation and positioning was performed using Juicebox (v1.11.08, https://github.com/aidenlab/Juicebox). Subsequently, genome alignment was conducted with Winnowmap (v1.11, https://github.com/marbl/Winnowmap). Gap filling and telomere extension were performed with TGS‐GapCloser (v1.2.1, https://github.com/BGI‐Qingdao/TGS‐GapCloser) using ~25.6 Gb (~83.4× coverage) ultra‐long Nanopore reads and an in‐house python script. To further enhance assembly quality, HiFi reads were used to polish the genome using Inspector software (v1.0.1, https://github.com/Maggi‐Chen/Inspector) at default settings. Additionally, 123.9 Gb of BioNano optical map data (~406× coverage) were integrated to improve genome assembly (Figure S1).

The quality of the assembled T2T sesame genome was assessed using three approaches: (1) Benchmarking Universal Single‐Copy Orthologs (BUSCO) (https://gitlab.com/ezlab/busco/‐/releases#5.7.0, Simao et al. 2015), to evaluate genome completeness using embryophyte_odb10 database. (2) Merqury analysis (v1.3, https://github.com/marbl/merqury), to assess genome accuracy by mapping short reads to the assembled genome; (3) LTR Assembly Index (LAI) was calculated using LTR_retriever (v3.0.1, https://github.com/oushujun/LTR_retriever), to evaluate genome continuity based on LTR retrotransposon content. Additionally, sequencing depth across genomic regions was determined separately for HiFi and Nanopore reads.

### 5.4 Telomere and Centromere Identification

Telomeric repeats were identified by screening the assembled genome against known plant telomere sequences using the ‘Telomere Database’ (http://telomerase.asu.edu/sequences_telomere.html). The tidk tool (v0.2.41, https://github.com/tolkit/telomeric‐identifier) was used to detect telomeric repeat units with its ‘explore’ module and to map telomeric repeat positions on each chromosome with the ‘search and plot’ command. Centromeric regions were identified using Centromics (v0.3, https://github.com/ShuaiNIEgithub/Centromics) with default parameters, based on HiFi read alignments and the genome assembly. Candidate centromeres were defined as regions with a high density of short tandem repeats, low gene density, and other centromere‐associated features. Known ribosomal DNA repeats (45S and 5S rDNAs) and the previously identified centromere repeat *SiCen1* (Miao et al. 2024) were also employed to validate assembly integrity of 13 chromosomes and centromere regions.

### 5.5 Genome Annotation

Genome annotation was preceded by repeat sequence identification using both *de novo* and homology‐based methods. RepeatModeler (v1.0.11, parameters: ‐pa 10, https://github.com/Dfam‐consortium/RepeatModeler) was used to identify *de* novo transposable elements and to construct a repeat model library. Long terminal repeats (LTR) were detected using LTR_FINDER_parallel (https://github.com/oushujun/LTR_FINDER_parallel; parameters: ‐threads 16 ‐harvest_out ‐size 1 000 000 ‐time 300). Non‐redundant LTR sequences were curated using LTR_retriever (v2.9.0, https://github.com/oushujun/LTR_retriever) with default parameters. The LTR sequences from both tools were merged to construct a *de novo* repeat sequence library. RepeatProteinMask (v4.0.9, https://www.repeatmasker.org/) was used to identify TE protein sequences. This *de novo* repeat library, together with homologous repeat sequences from RepBase (repbase20.05, https://www.girinst.org/downloads/), was applied as the database for RepeatMasker (v4.0.9, parameters: ‐nolow ‐no_is ‐norna ‐parallel 2).

Protein‐coding genes were predicted using three complementary approaches: homologue‐based, transcriptome‐based, and *ab* initio prediction. For homologue‐based mapping, protein sequences from five reference species— *S. indicum* (v3.0), *Arabidopsis thaliana* , *Mimulus guttatus* , *Solanum lycopersicum* , and *Vitis vinifera* (https://jgi.doe.gov/) – along with the UniProt plant protein dataset were aligned to the assembled genome using the MAKER pipeline (v3.01.03, https://github.com/Yandell‐Lab/maker).

For transcriptome‐based prediction, RNA‐seq reads and full‐length transcripts collected from Nanopore sequencing were mapped to the assembled genome using HISAT2 (v2.1.0, default parameters, http://daehwankimlab.github.io/hisat2/) and minimap2 (v2.17‐r941, parameters: ‐axe map‐ont ‐xsplice ‐G 1000000, https://github.com/lh3/minimap2). Stringtie (v2.1.4, https://github.com/gpertea/stringtie) was used to reconstruct transcripts from various sesame tissues. Gene models were predicted using SNAP (v2006‐07‐28, https://github.com/KorfLab/SNAP) and Augustus (v3.4.0, https://github.com/Gaius‐Augustus/Augustus). EST sequences generated by Stringtie and full‐length transcripts were used as cDNA evidence to validate the predicted genes with the MAKER pipeline (v3.101.03, https://github.com/Yandell‐Lab/maker).

For repeat masking, the assembled genome was screened using the RepBase repeat library (repbase20.05) and RepeatMasker (v4.1.2‐p1, https://www.repeatmasker.org/) within the MAKER pipeline. Gene models were finalised in MAKER using combined evidence from all three approaches, applying the following parameters: organism_type = eukaryotic, alt_peptide = C, max_dna_len = 100 000, min_contig = 1, pred_flank = 200, pred_stats = 0, AED_threshold = 1, min_protein = 20, alt_splice = 1, map_forward = 0, keep_preds = 0, split_hit = 10 000, single_exon = 1, single_length = 250, correct_est_fusion = 0, and always_complete = 0.

Functional annotation of predicted proteins was performed using DIAMOND (v2.1.8.162, https://github.com/bbuchfink/diamond); parameters (−e 1e‐5 ‐‐more‐sensitive ‐‐max‐target‐seqs 1) by querying the NR (https://www.ncbi.nlm.nih.gov/), Pfam (http://ftp.ebi.ac.uk/pub/databases/Pfam/releases/), SwissProt (https://www.uniprot.org/downloads), and Gene Ontology (GO) (http://geneontology.org/) databases. NOG and KEGG annotations were obtained using Emapper (v2.1.12, https://github.com/eggnogdb/eggnog‐mapper). All annotation outputs were integrated using Trinotate (v4.0.2, https://github.com/Trinotate/Trinotate).

Transfer RNA (tRNA) genes were predicted with tRNAscan‐SE (v2.0.12, https://github.com/UCSC‐LoweLab/tRNAscan‐SE) under default parameters. Ribosomal RNA (rRNA) genes were identified using barrnap (v0.9, https://github.com/tseemann/barrnap); parameters: (‐‐incseq ‐‐kingdom euk). MicroRNA (miRNA) and small nuclear RNA (snRNA) genes were annotated using cmscan (v1.1.4, https://github.com/EddyRivasLab/infernal) and the Rfam14.10 database (https://ftp.ebi.ac.uk/pub/databases/Rfam/) with default parameters.

### 5.6 Chromosome Synteny and Structural Variation Analysis

Genome synteny and assembly quality were assessed by aligning the assembled genome to the *S. indicum* var. Baizhima genome (Wang, Huang, et al. 2022; https://figshare.com/articles/dataset/An_improved_assembly_and_annotation_of_the_sesame_genome/21151948) using NUCmer (v3.1, https://github.com/mummer4/mummer; parameters: ‐‐mum ‐mincluster 200 –minmatch 100). Delta‐filter was applied with parameters ‐I 95 ‐l 100 ‐1 for post‐alignment filtering.

Structural variations (SVs) were detected using SYRI (v1.6.3, https://github.com/schneebergerlab/syri) and Assemblytics (v1.2.1, https://github.com/MariaNattestad/Assemblytics). Inversions (INV) and translocations (TRA) were identified by SYRI, while insertions (INS), deletions (DEL), and duplications (DUP) were detected by Assemblytics. Identified SVs were annotated using ANNOVAR (v2020‐06‐07, https://www.openbioinformatics.org/annovar/annovar_download_form.php).

### 5.7 Gene Duplication and Gene Positive Selection Analysis

Gene duplication events in sesame were identified using DupGen_finder (https://github.com/qiao‐xin/DupGen_finder), comparing the assembled T2T genome with the Baizhima genome (Wang, Huang, et al. 2022). Positive selection analysis was conducted with Calculate_Ka_Ks_pipe (https://github.com/qiao‐xin/Scripts_for_GB) and KaKs_Calculator (v2.0, https://github.com/kullrich/kakscalculator2) using the NG model to estimate Ka, Ks, and Ka/Ks ratios within key gene families.

### 5.8 Whole Genome Methylation Analysis

Genome‐wide methylation profiling was performed by detecting 5‐methylcytosine (5mC) modifications from HiFi sequencing reads based on the kinetic signatures at CpG sites. Methylation calling was conducted using Primrose (v1.3.0, smartlink12.0.0.177059), generating detailed modified tag data. Reads were aligned to the assembled genome using pbmm2 (v1.10.0, https://github.com/PacifcBiosciences/pbmm2) and CpG methylation sites were identified using pb‐CpG‐tools (v2.3.1, https://github.com/PacifcBiosciences/pb‐CpG‐tools/).

### 5.9 Genome Re‐Sequencing and Variant Calling of 927 Sesame Accessions

High‐quality DNA was extracted from young fresh leaves of 927 sesame accessions (Table S12) using a modified CTAB method (Stefanova et al. 2013). Libraries with 350 bp insert size were constructed and sequenced on an Illumina HiSeq 2500 platform (Illumina, San Diego, CA). Sequencing quality was assessed using FastQC (v0.11.9, https://github.com/s‐andrews/FastQC). Low‐quality reads were filtered based on the following criteria: (1) reads containing more than 5% N bases, (2) reads with over 50% low‐quality bases (Phred value < 19), and (3) reads with more than five adapter‐contaminated bases, screened using fqtools (v0.18, https://github.com/alastair‐droop/fqtools). Clean reads were aligned to the T2T genome using BWA (v0.7.17, https://github.com/lh3/bwa) with default parameters. Alignments were sorted with samtools (v1.3.1, https://sourceforge.net/projects/samtools/files/samtools/1.3.1/). Duplicated reads were removed using the Picard MarkDuplicates tool (v2.27.4–0, https://github.com/broadinstitute/picard).

Variant calling was performed with the Genome Analysis Toolkit (GATK, v4.4.0.0, https://github.com/broadinstitute/gatk) using the HaplotypeCaller model. GVCF files were merged with CombineGVCFs and genotyped using GenotypeGVCFs. SNPs and InDels were selected with SelectVariants and filtered with VariantFiltration using parameters (FS > 60.0, QD < 4, MQ < 40.0, GQ < 20, and ‐window 4). High‐confidence variants were annotated with ANNOVAR (v2020‐06‐07, https://www.openbioinformatics.org/annovar/annovar_download_form.php) using the T2T genome annotation.

### 5.10 Population Structure Analysis

High‐quality variants from the 927 sesame accessions were filtered based on the following thresholds: minor allele frequency (MAF) < 0.01, SNP missing rate > 0.2, max alleles = 2, and sample missing rate > 0.1. After filtering, a total of 2 732 061 SNPs were retained for population analysis. Variants were pruned for linkage disequilibrium (LD) analysis using PLINK (v1.9; parameters: ‐‐indep‐pairwise 10 000 100 0.5, https://www.cog‐genomics.org/plink/). Principal component analysis (PCA) was performed using GCTA (v1.94.1, https://github.com/jianyangqt/gcta) with default parameters. A phylogenetic tree was constructed using iqtree2 (v2.2.0.3, https://github.com/iqtree/iqtree2; parameters: ‐st DNA ‐T 2 ‐mem 8G ‐m GTR ‐redo ‐B 1000 ‐bnni) and visualised and manually modified using iTOL (https://itol.embl.de/).

Population structure was inferred using fastSTRUCTURE (v1.0, https://github.com/rajanil/fastStructure; parameters: ‐‐full ‐‐seed = 100 ‐‐cv 5). The optimal number of subgroups (*K* = 2–10) was determined by maximising the marginal likelihood using chooseK, with cross‐validation (CV) error additionally used to support optimal K selection. Linkage disequilibrium (LD) decay was evaluated using *r* ^2^ values calculated by PopLDdecay (v3.31, https://github.com/BGI‐shenzhen/PopLDdecay; parameters: ‐MaxDist 300 ‐Het 0.88). Pairwise population differentiation (*F*_ST_) and selective sweep signals (XP‐CLR) were estimated using VCFtools (v 0.1.13) and xpclr (v1.1.2), respectively, with 20‐kb sliding windows and 5‐kb step sizes. Candidate selection regions were defined as genomic regions simultaneously falling within the top 5% of both *F*_ST_ and XP‐CLR. Nucleotide diversity (π) was estimated using Pixy (v1.2.5, https://github.com/ksamuk/pixy) with 20‐kb non‐overlapping windows. To reduce the effect of extreme sample size imbalance on *F*_ST_ and π analyses, 69 landraces accessions were randomly selected from the North China group.

### 5.11 Demographic Analysis of Sesame Core Population

Demographic history and population divergence were inferred using SMC++ (v1.15.3, https://github.com/popgenmethods/smcpp) based on unphased SNPs with a minor allele frequency (MAF) > 0.01. The mutation rate of 6.95 × 10^−9^ substitutions per site per generation and a generation time of 1 year were used to scale population size and divergence time estimates. To further investigate population split and admixture, TreeMix (v1.15.3, https://github.com/carolindahms/TreeMix) was used to construct a population graph based on the core SNP set. TreeMix analyses were performed five times, allowing 1 to 9 admixture events (m). Migration edges were predicted using the Evanno model, providing insights into population dynamics and historical relationships among eight sesame populations.

Chromosome‐wide introgression was estimated using the *f*d statistic calculated in 20 Kb sliding windows with 5 Kb overlapping steps, implemented via ABBABABAwindows.py (https://github.com/simonhmartin/genomics_general). We defined putatively introgressed genomic windows using the following stringent criteria: (1) Statistical Significance: Only windows with a Z‐score greater than 3 were retained to ensure robust detection of introgression signals. (2) Signal Presence: Windows where the *D*‐statistic equaled zero (*D* = 0) were excluded, as these represent the absence of any detectable introgression. (3) SNP Density Requirement: To mitigate stochastic variation and enhance the reliability of our estimates, each window was required to contain a minimum of 100 SNPs. These filters collectively ensured that our analysis focused on high‐confidence introgression events. Pairwise population comparisons were conducted among accessions originating from West Africa, South Asia, South China, North China, and East Africa, with East Africa used as the outgroup due to its genetic diversity. Average *f*d values were computed to assess introgression patterns and gene flow directionality across populations.

### 5.12 Genome‐Wide Association Study Analysis of Flowering Time

For GWAS analysis, SNPs from 235 sesame accessions which presented the stable phenotypes with early or late flowering time trait under specific environment were filtered using thresholds: MAF < 0.01, maximum missing rate > 0.2, minimum alleles = 2, and max alleles = 2. A total of 2 625 501 SNPs were used for GWAS analysis of days from sowing to flowering (DF) using Genome Association and Prediction Integrated Tools (GAPIT, v3.5, https://github.com/jiabowang/GAPIT) with the FarmCPU model. The kinship matrix was constructed using GCTA (https://github.com/jianyangqt/gcta, v1.94.1) to account for genetic relatedness, and the covariate matrix derived from fastStructure (https://github.com/rajanil/fastStructure) was employed to correct for population structure with default parameters. A genome‐wide significance threshold was determined using Bonferroni correction (1/2 625 501 = 1.90e‐08). Manhattan and QQ plots were generated using qmplot (https://github.com/ShujiaHuang/qmplot). Haplotype analysis was performed for genomic regions surrounding significant SNPs, with 65 kb upstream and downstream flanking sequences screened.

### 5.13 Constructing a Flowering Time Classification Model

A list of 306 flowering time related genes was obtained from the Flowering Interactive Database (http://www.phytosystems.ulg.ac.be/florid/databases/gene_list/flowering). Homologues in the sesame genome were identified by BLASTP (identity > 30 and E‐value < 1e‐10). Non‐synonymous mutations within these homozygous genes were extracted to analyse the effects of genotype classification models on flowering time and phenotype prediction. Sesame accessions grown at Yuanyang were classified into early‐flowering (EDF, DF ≤ 60 days) and late‐flowering (LDF, DF > 60 days) groups. A DF classification model was constructed using a random forest algorithm, integrating genotype and phenotype data of 927 sesame accessions from multiple years and environments.

### 5.14 UBP Gene Family Evolution Analysis

Homologue(s) of target genes were identified in the T2T sesame genome, the genomes of six wild *Sesamum* species (Miao et al. 2024) and 20 homology species using BLASTP and hmmsearch. Multiple sequence alignments and phylogenetic tree were constructed using MAFFT v7.5 (Katoh and Standley 2013) and IQ‐TREE v 2.2.0.3 (Minh et al. 2020).

## Author Contributions

H.M. and H.Z. designed the experiment and edited the manuscript. A.H.P. edited the manuscript. H.M., H.Z., W.Y., Y.D. and H.C. drafted the manuscript. H.M. and W.Y. performed genome assembly and annotation. W.Y. and G.L. preformed comparative genome analysis. H.C. and H.G. and G.L. performed the GWAS and selective analysis. W.Y., H.C., L.W. and H.G. conducted phylogenetic and population genomic analysis. Q.T., P.C., and C.M. performed DNA extraction. Q.M. and M.J. established the consecutive BAC‐FISH technique in sesame. Z.Z., X.F., and W.H. performed agronomic trait investigation and analysis.

## Conflicts of Interest

The authors declare no conflicts of interest.

## Supporting information


**Figure S1:** Whole genome sequencing and telomere to telomere (T2T) genome assembly strategy for sesame.
**Figure S2:** The frequency distribution of 19‐mers.
**Figure S3:** Hi‐C heatmap of sesame chromosome interactions. The Hi‐C map is manually adjusted based on BioNano sequencing data. Bin size = 100 Kb.
**Figure S4:** The depth distribution of HiFi and ONT reads across T2T sesame genome.
**Figure S5:** Distribution of genomic features across chromosomes. Telomere repeats are shown in black triangle on the tips of 10 chromosomes, except for SiChr.11, 12 and 13. The satellite regions of SiChr.11, 12 and 13 are shown in blue balls. Centromere regions of each chromosome are shown in cross‐cutting regions.
**Figure S6:** GO enrichment for genes in centromere regions of sesame.
**Figure S7:** Statistics of SVs between the T2T sesame genome and Genome var. Baizhima. (A) Distribution of SV length between T2T sesame genome and the published genome var. Baizhima. (B) Positional annotation of SVs between T2T sesame genome and the published genome var. Baizhima. Ratios of 1359 insertions, 1251 deletions, 8 inversions locating in intergenic, upstream and downstream of genes, gene intronic or exonic positions are shown.
**Figure S8:** GO and KEGG enrichments of genes in SVs between the T2T sesame genome and Genome var. Baizhima.
**Figure S9:** GO and KEGG enrichments of proximal duplication genes.
**Figure S10:** Distribution and statistics of variants from 927 sesames accessions. (A) Density plot of high‐quality SNPs. A total of 2 732 061 high‐quality SNPs are detected in sesame genome. Window size is 50 Kb in each chromosome. (B) Pie chart of annotation of the high‐quality SNPs. All SNPs locate in intergenic, upstream and downstream of genes, gene intronic or exonic positions.
**Figure S11:** Composite likelihoods analysis of *m* = 2 simulated migration edges with various ecological threshold models fit using OptM. Each panel represents the observed composite likelihoods (black circles) for each TREEMIX run using the four models fit. The change points predicted by each model are drawn as coloured stars.
**Figure S12:** Identification of possible introgression events according to branch‐specific statistic *f*_b_. Eight geographical regions including East Africa, West Africa, West Asia, South Asia, South‐East Asia, North America, South China, North China for sesame accessions are shown in figure.
**Figure S13:** Estimated regions of introgression segments from different population around the whole genome. (A) Estimated regions introgression segments to South China from South Asia, West, Asia, and West Africa. (B) Estimated regions introgression segments to North China from South Asia, West, Asia, West Africa, and South China. Number in parentheses represents the number of introgression segments.
**Figure S14:** GO and KEGG enrichment for genes flowed from West Africa to North China. (A) GO enrichment for genes. (B) KEGG enrichment for genes.
**Figure S15:** GO and KEGG enrichment for genes flowed from West Africa to South China. (A) GO enrichment for genes. (B) KEGG enrichment for genes.
**Figure S16:** GO and KEGG enrichment for genes flowed from South Asia to North China. (A) GO enrichment for genes. (B) KEGG enrichment for genes.
**Figure S17:** GO and KEGG enrichment for genes flowed from South Asia to South China. (A) GO enrichment for genes. (B) KEGG enrichment for genes.
**Figure S18:** GO and KEGG enrichment of genes in high *F*_*ST*_ regions between South Asia and North China. (A) GO enrichment for genes. (B) KEGG enrichment for genes.
**Figure S19:** QQ plot for GWAS of flowering time trait among the 235 sesame accessions. Phenotype data in 2022 is used.
**Figure S20:** Phylogenetic tree of UBP from 27 species. (A) The phylogenetic tree of 6 *ZnF‐UBP* genes from *S. indicum* var. Yuzhi11 T2T and all *UBP* gene family of *A. thaliana* . (B) The phylogenetic tree of all *ZnF‐UBP* genes from 27 species. Neighbour‐joining (NJ) method with a bootstrap test (*n* = 1000 replications) was used to construct the phylogenetic tree. *SiUBP16* (*Sin2G02467*) is shown in red colour.
**Figure S21:** Domain composition overview for SiUBP16 protein.
**Figure S22:** Expression of homologous genes regulating flowering time in different tissues of sesame cv. Yuzhi 11. Root, stem, flower, pod and leaf tissues are assayed for sesame genes regulating flowering time.
**Figure S23:** Relationship between the number of early DF genotype alleles and DF trait in sesame population. DF indicates the days from sowing to flowering. Three group data of the 927 sesame accessions at Yuanyang experimental station in 2019, 2020, and 2021 year, respectively are shown in three figures.

**Table S1:** Summary of sequencing data used for the genome assembly of *S. indicum* var. Yuzhi11.
**Table S2:** Genome survey and assembly statistics.
**Table S3:** Comparison of the T2T sesame genome with the published SiChr.omosome‐scaled sesame genomes.
**Table S4:** Distribution of telomere region in T2T sesame genome.
**Table S5:** Statistics of BUSCO and merqury evaluation of T2T sesame genome.
**Table S6:** Statistics of annotated genes in T2T sesame genome using various protein databases.
**Table S7:** Distribution of repeat sequences of T2T sesame genome.
**Table S8:** Statistics of ncRNAs in T2T sesame genome.
**Table S9:** Distribution of centromere regions predicted in T2T sesame genome.
**Table S10:** Genes description affected by SV between Yuzhi11 and Baizhima.
**Table S11:** Statistics of different modes of gene duplication in T2T sesame genome and sesame genome var. Baizhima.
**Table S12:** Statistics of Ka/Ks for different modes of duplicated genes from T2T sesame genome and the genome var. Baizhima.
**Table S13:** Statistics of genome resequencing data of 927 worldwide sesame accessions.
**Table S14:** Summary of sample sizes by collection site and group.
**Table S15:** Statistics of gene flow among the eight populations of 927 sesame accessions.
**Table S16:** Statistics of DF trait of 927 sesame germplasm accessions in two positions for 4 years.
**Table S17:** Phenotypic characteristics for flowering time in the 245 Sesamum indicum accessions planted in Yuanyang.
**Table S18:** Genomic regions with high divergence between ‘North China’ and ‘South Asia’ group among the 90 sesame accessions.
**Table S19:** Statistics of flowering date related genes subject to selection.
**Table S20:** Candidate genes putatively associated with DF using GWAS method.
**Table S21:** Gene flow intervals from ‘South Asia’ population into ‘North China’ population.
**Table S22:** Number of genes keeping ZnF UBP domain identified in 27 species.
**Table S23:** Prediction for PXLXP motif of homologous sesame genes to *Arabidopsis thaliana* regulating flowering time.

## Data Availability

The raw sequencing data used for T2T genome assembly is available from the National Genomics Data Center (https://bigd.big.ac.cn/) (PRJCA024033).
