# Supplementary material for: Insights Into the Origin and Local Adaptation Evolution of the Cultivated Sesame With Telomere‐to‐Telomere High‐Quality Genome
Source: Plant Biotechnol J. 2026 Jul 4:10.1111/pbi.70714. Online ahead of print. doi: 10.1111/pbi.70714 (PMC13398907; doi:10.1111/pbi.70714)
Supplement: Supplementary file 1 — Figure S1: Whole genome sequencing and telomere to telomere (T2T) genome assembly strategy for sesame. Figure S2: The frequency distribution of 19‐mers. Figure S3: Hi‐C heatmap of sesame chromosome interactions. The Hi‐C map is manually adjusted based on BioNano sequencing data. Bin size = 100 Kb. Figure S4: The depth distribution of HiFi and ONT reads across T2T sesame genome. Figure S5: Distribution of genomic features across chromosomes. Telomere repeats are shown in black triangle on the tips of 10 chromosomes, except for SiChr.11, 12 and 13. The satellite regions of SiChr.11, 12 and 13 are shown in blue balls. Centromere regions of each chromosome are shown in cross‐cutting regions. Figure S6: GO enrichment for genes in centromere regions of sesame. Figure S7: Statistics of SVs between the T2T sesame genome and Genome var. Baizhima. (A) Distribution of SV length between T2T sesame genome and the published genome var. Baizhima. (B) Positional annotation of SVs between T2T sesame genome and the published genome var. Baizhima. Ratios of 1359 insertions, 1251 deletions, 8 inversions locating in intergenic, upstream and downstream of genes, gene intronic or exonic positions are shown. Figure S8: GO and KEGG enrichments of genes in SVs between the T2T sesame genome and Genome var. Baizhima. Figure S9: GO and KEGG enrichments of proximal duplication genes. Figure S10: Distribution and statistics of variants from 927 sesames accessions. (A) Density plot of high‐quality SNPs. A total of 2 732 061 high‐quality SNPs are detected in sesame genome. Window size is 50 Kb in each chromosome. (B) Pie chart of annotation of the high‐quality SNPs. All SNPs locate in intergenic, upstream and downstream of genes, gene intronic or exonic positions. Figure S11: Composite likelihoods analysis of m = 2 simulated migration edges with various ecological threshold models fit using OptM. Each panel represents the observed composite likelihoods (black circles) for each TREEMIX run using the [file PBI-9999-0-s002.docx]

**
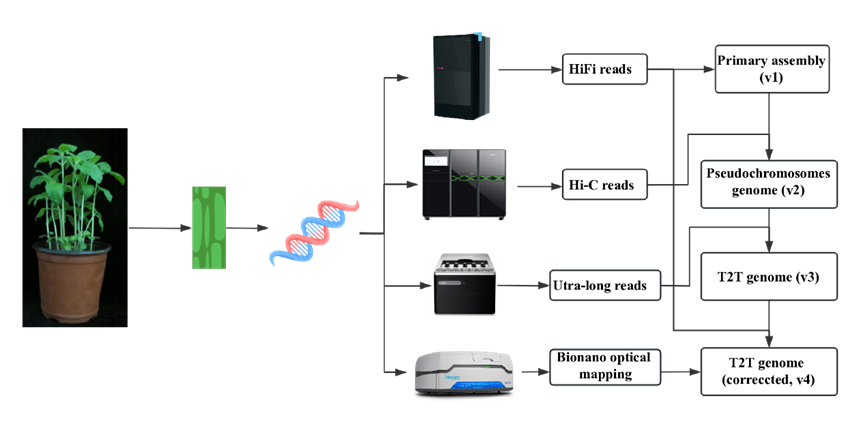
Supplementary Figures**

**Fig. S1. Whole genome sequencing and telomere to telomere (T2T) genome assembly strategy for sesame.**


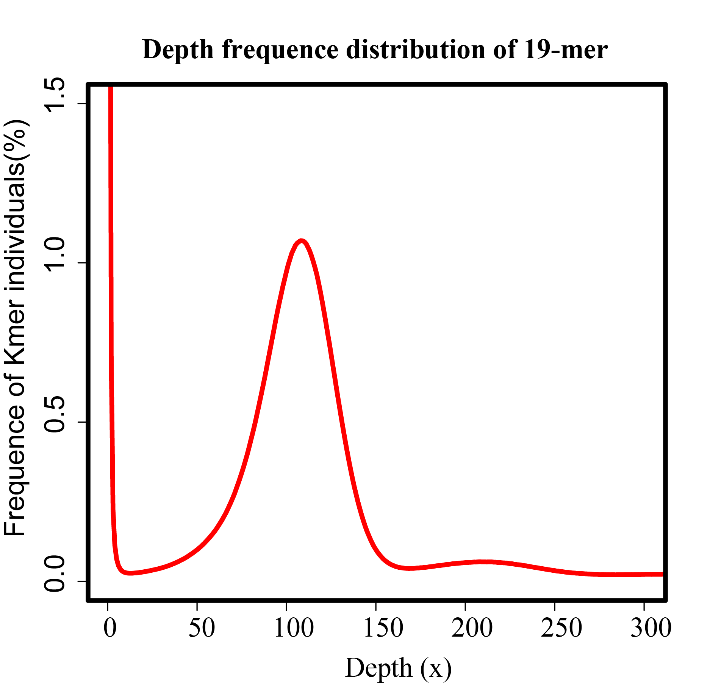


**Fig. S2. The frequency distribution of 19-mers.**


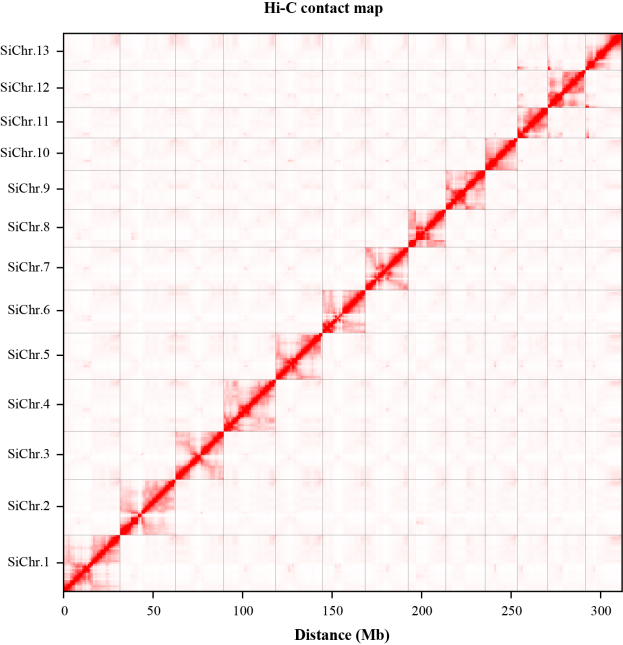
**Fig. S3. Hi-C heatmap of sesame chromosome interactions.** The Hi-C map is manually adjusted based on Bionano sequencing data. Bin size=100 Kb.


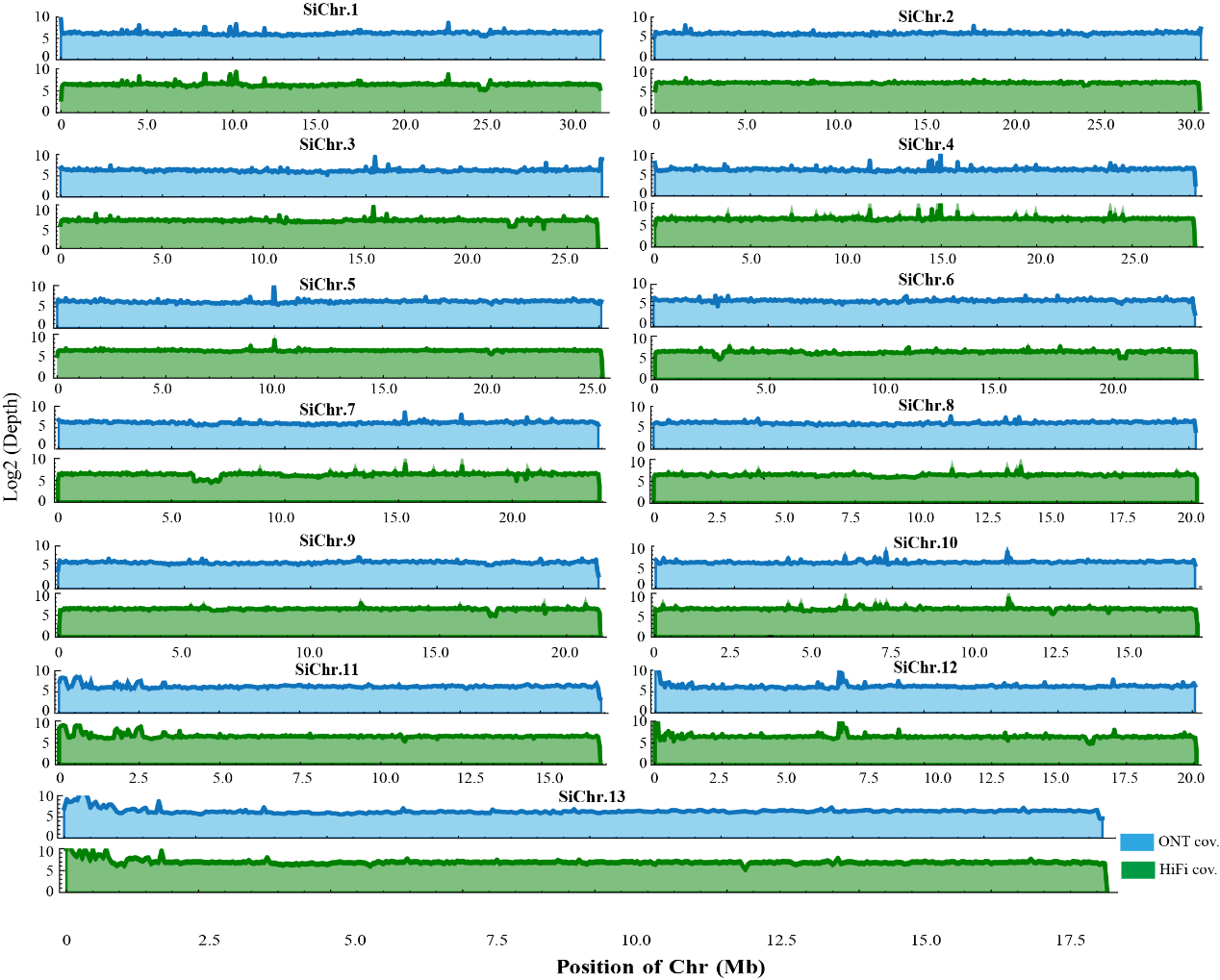


**Fig. S4. The depth distribution of HiFi and ONT reads across T2T sesame genome.**


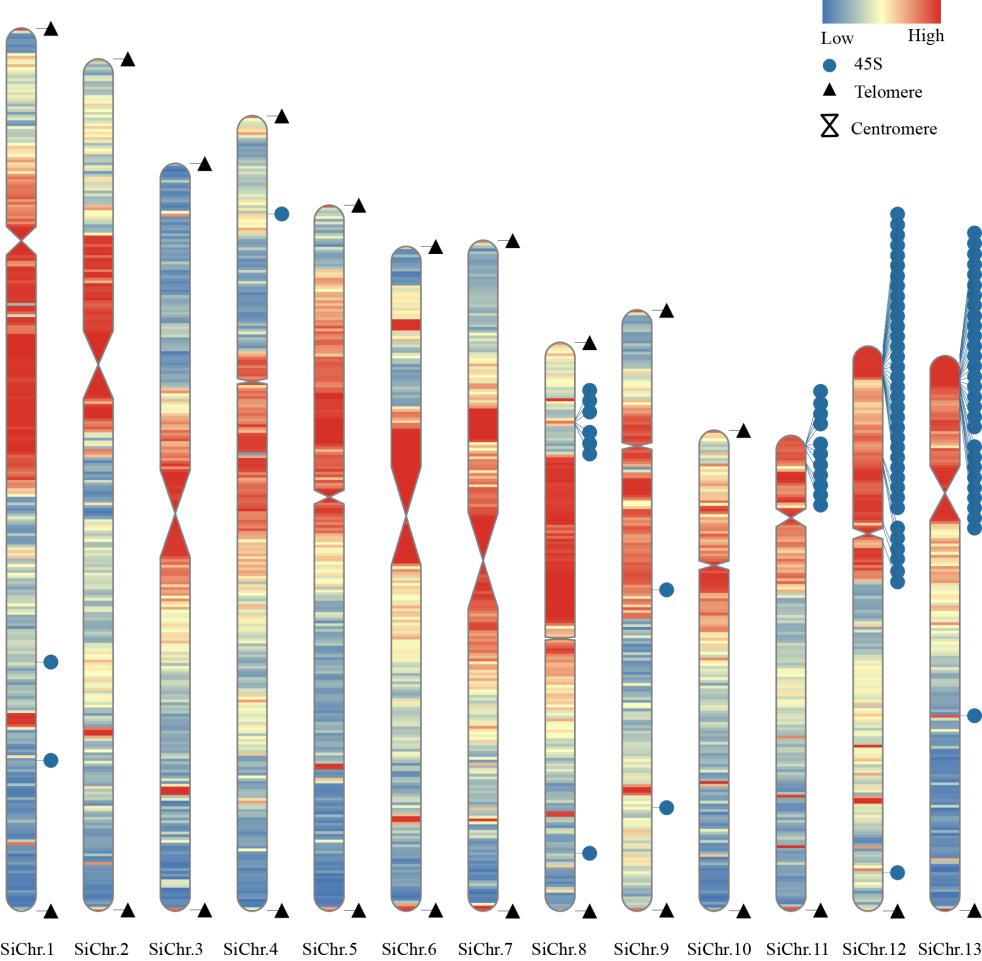


**Fig. S5. Distribution of genomic features across chromosomes.** Telomere repeats are shown in black triangle on the tips of 10 chromosomes, except for SiChr.11, 12 and 13. The satellite regions of SiChr.11, 12 and 13 are shown in blue balls. Centromere regions of each chromosome are shown in cross-cutting regions. Different color shades within the chromosomes represent the proportion of repeat content within 100kb.


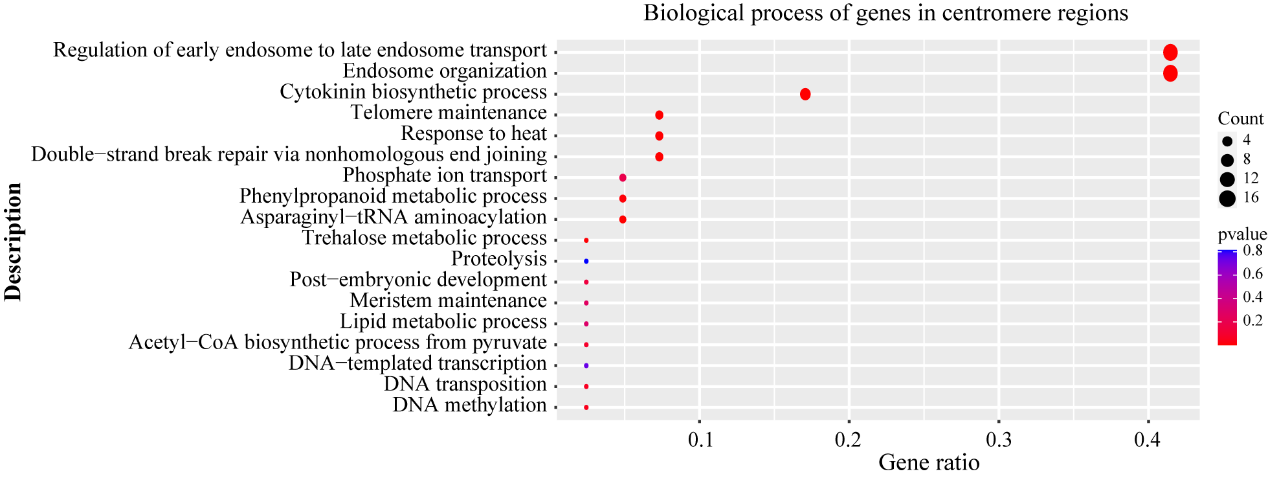


**Fig. S6. GO enrichment for genes in centromere regions of sesame.**


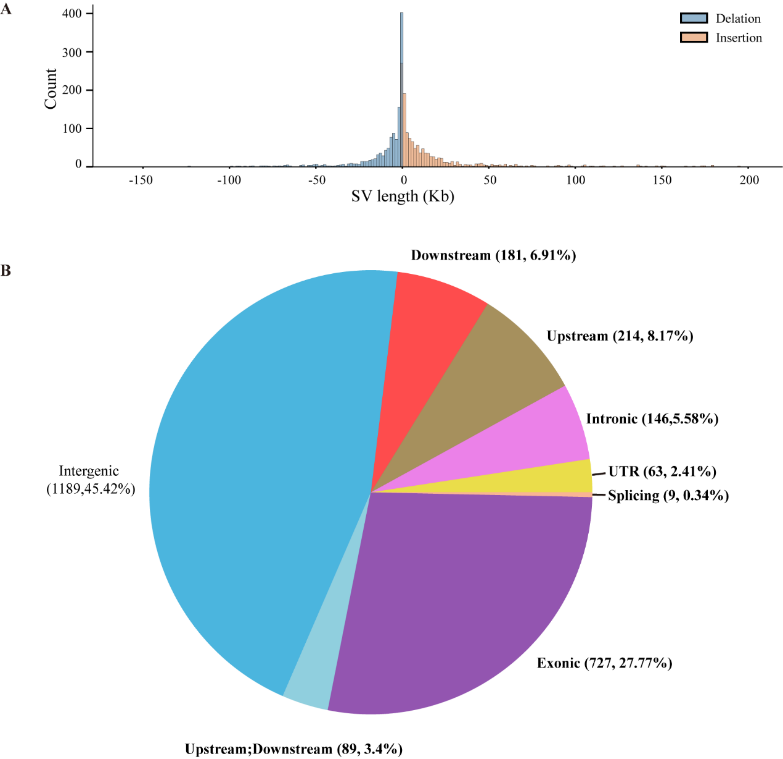


**Fig. S7. Statistics of SVs between the T2T sesame genome and Genome var. Baizhima. A:** Distribution of SV length between T2T sesame genome and the published genome var. Baizhima. **B:** Positional annotation of SVs between T2T sesame genome and the published genome var. Baizhima. Ratios of 1,359 insertions, 1,251 deletions, 8 inversions locating in intergenic, upstream and downstream of genes, gene intronic or exonic positions are shown.


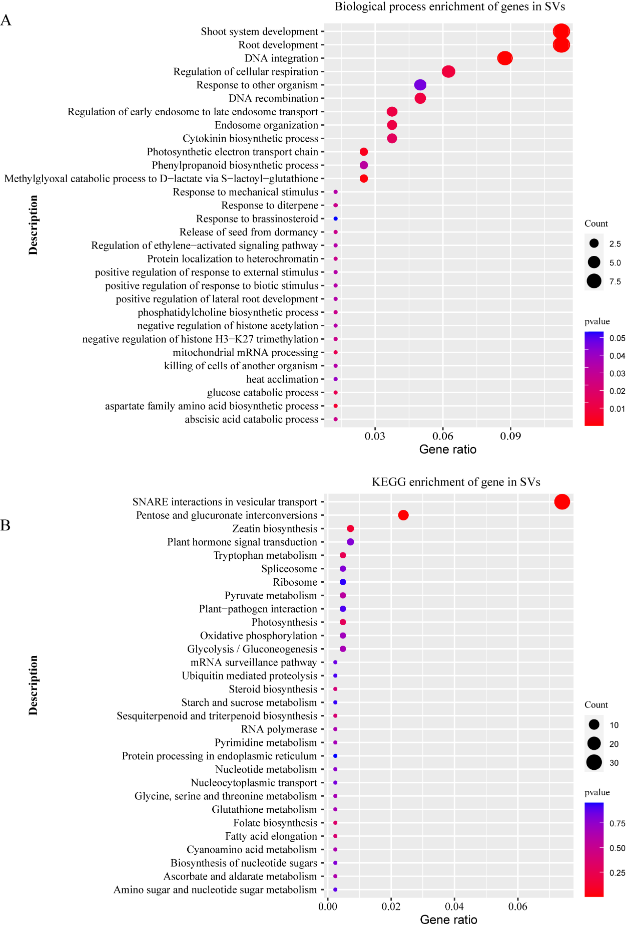


**Fig. S8. GO and KEGG enrichments of genes in SVs between the T2T sesame genome and Genome var. Baizhima.**


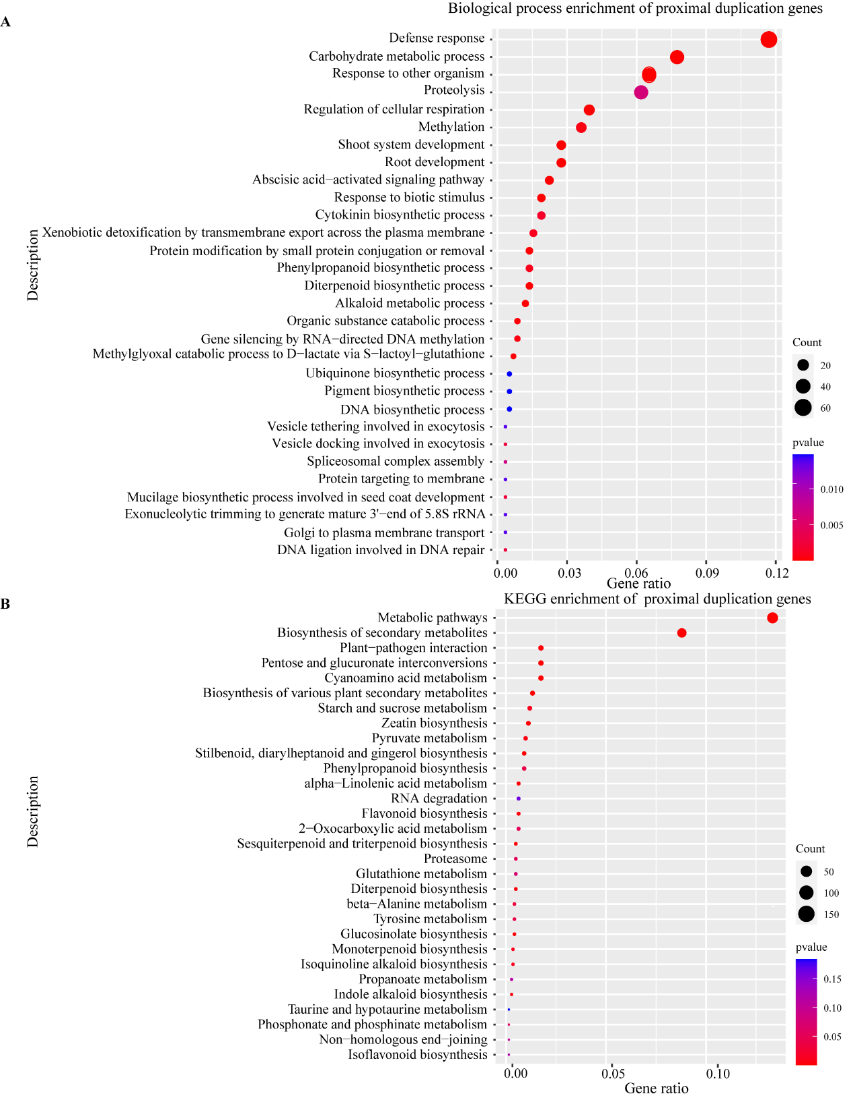


**Fig. S9. GO and KEGG enrichments of proximal duplication genes.**


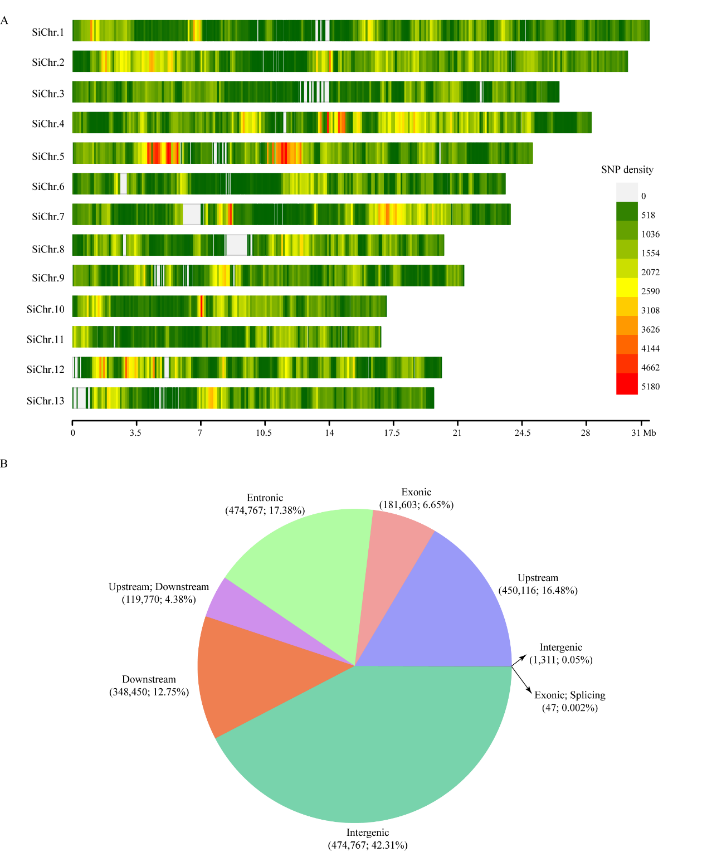


**Fig. S10. Distribution and statistics of variants from 927 sesames accessions. A:** Density plot of high-quality SNPs. A total of 2,732,061 high-quality SNPs is detected in sesame genome. Window size is 50 Kb in each chromosome. **B:** Pie chart of annotation of the high-quality SNPs. All SNPs locate in intergenic, upstream and downstream of genes, gene intronic or exonic positions.


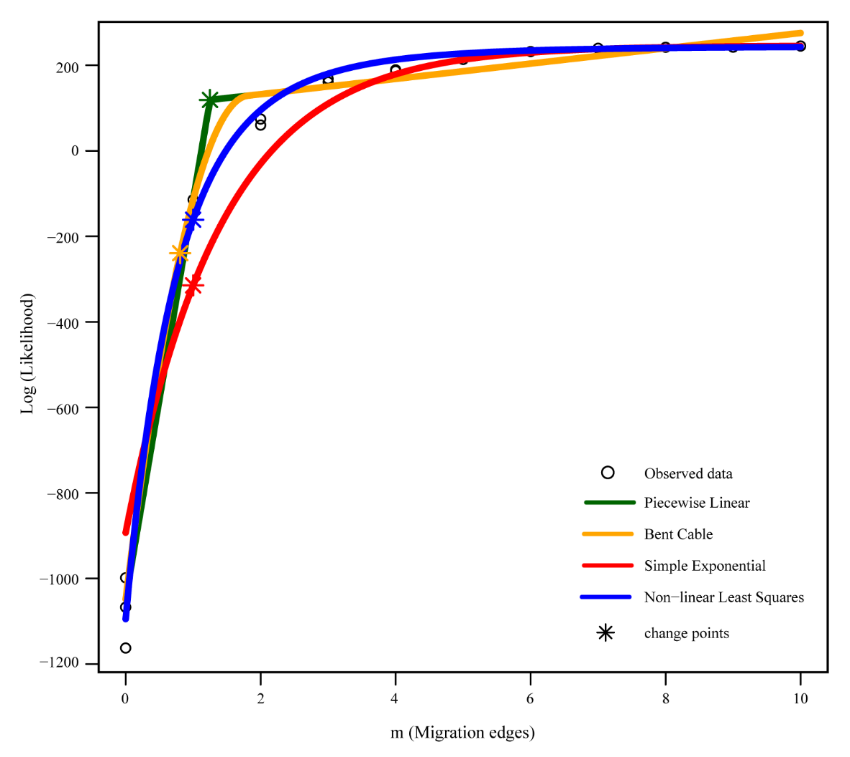


**Fig. S11. Composite likelihoods analysis of m=2 simulated migration edges with various ecological threshold models fit using OptM.** Each panel represents the observed composite likelihoods (black circles) for each TREEMIX run using the four models fit. The change points predicted by each model are drawn as colored stars.


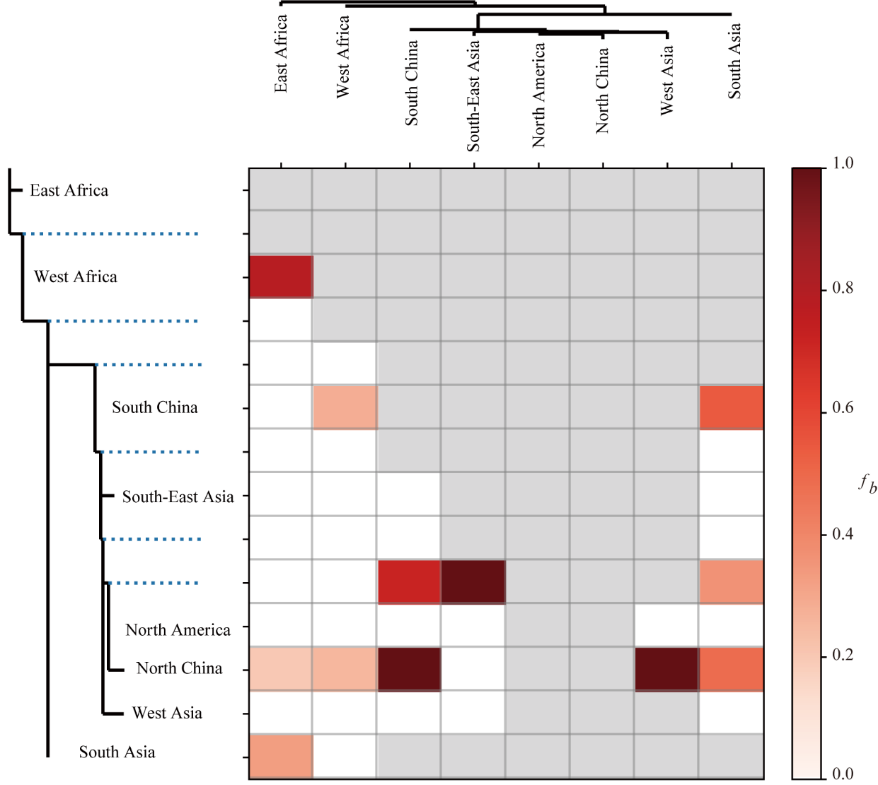


**Fig. S12. Identification of possible introgression events according to branch-specific statistic *f*_b_**. Eight geographical regions including East Africa, West Africa, West Asia, South Asia, South-East Asia, North America, South China, North China for sesame accessions are shown in figure.


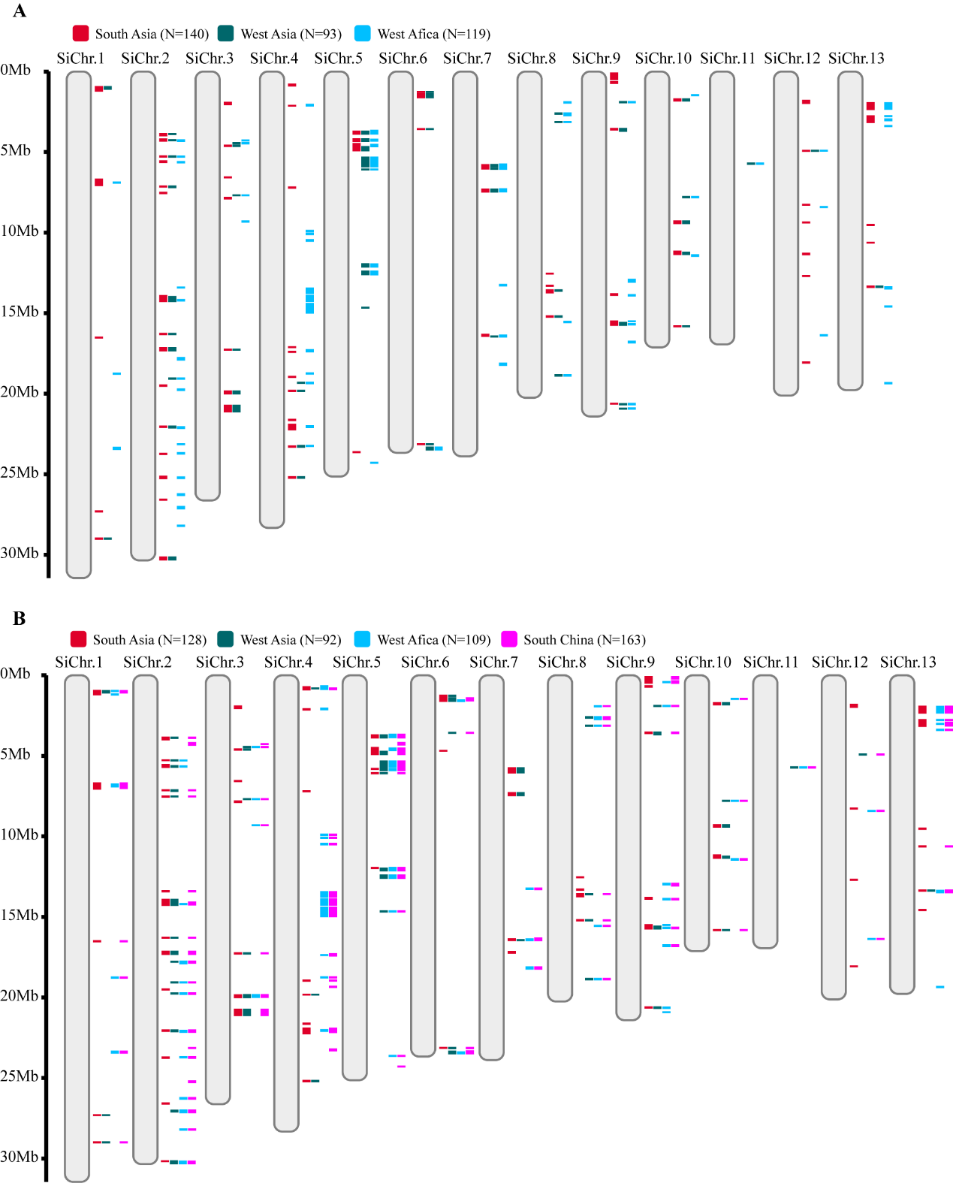


**Fig. S13. Estimated regions of introgression segments from different population around the whole genome.** **A:** Estimated regions introgression segments to Southern China from South Asia, West, Asia, and West Africa. **B:** Estimated regions introgression segments to North China from South Asia, West, Asia, West Africa, and South China. Number in parentheses represents the number of introgression segments.


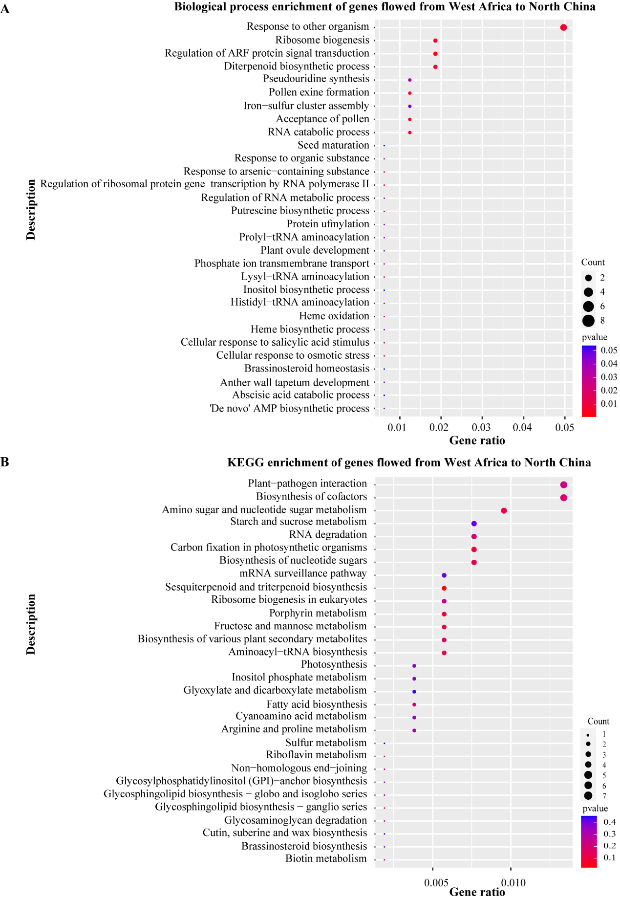


**Fig. S14.** **GO and KEGG enrichment** **for genes flowed from West Africa to North China.** **A:** GO enrichment for genes. **B:** KEGG enrichment for genes.


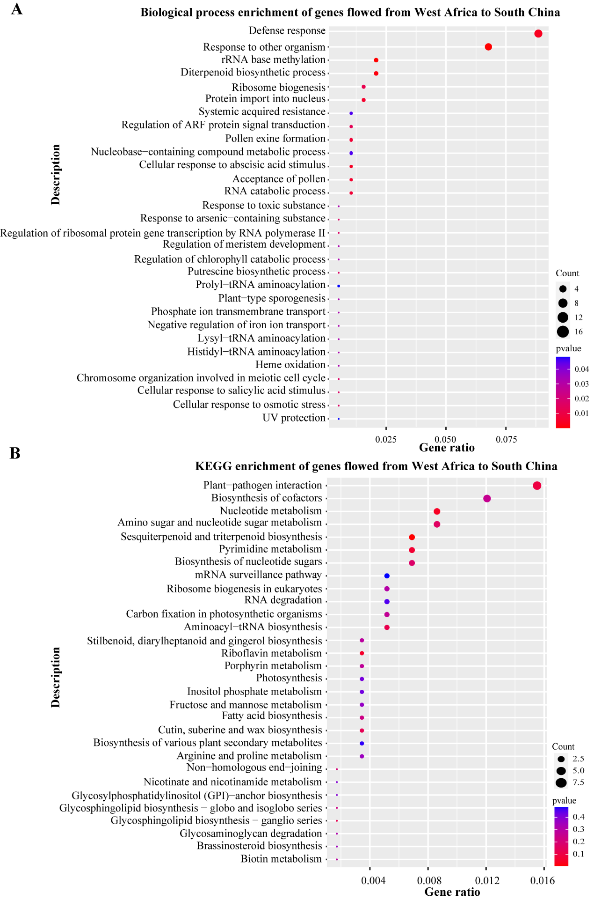


**Fig. S15. GO and KEGG enrichment for genes flowed from West Africa to South China. A:** GO enrichment for genes. **B:** KEGG enrichment for genes.


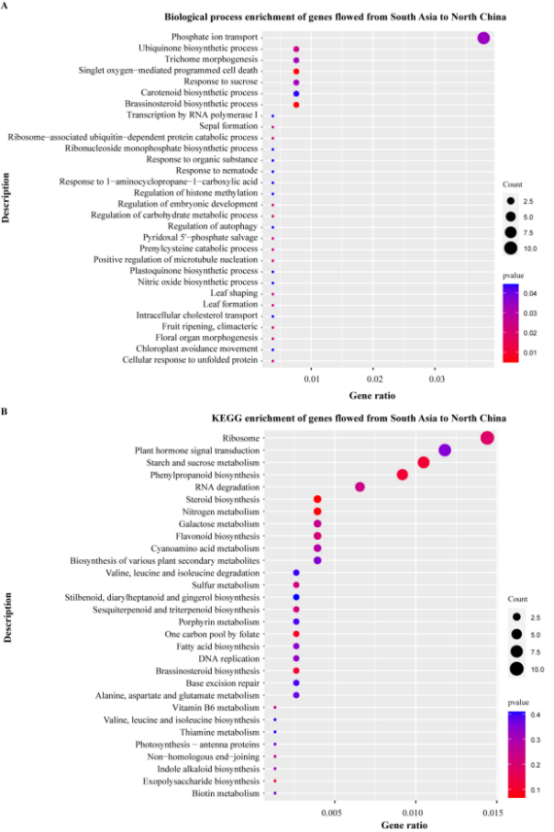


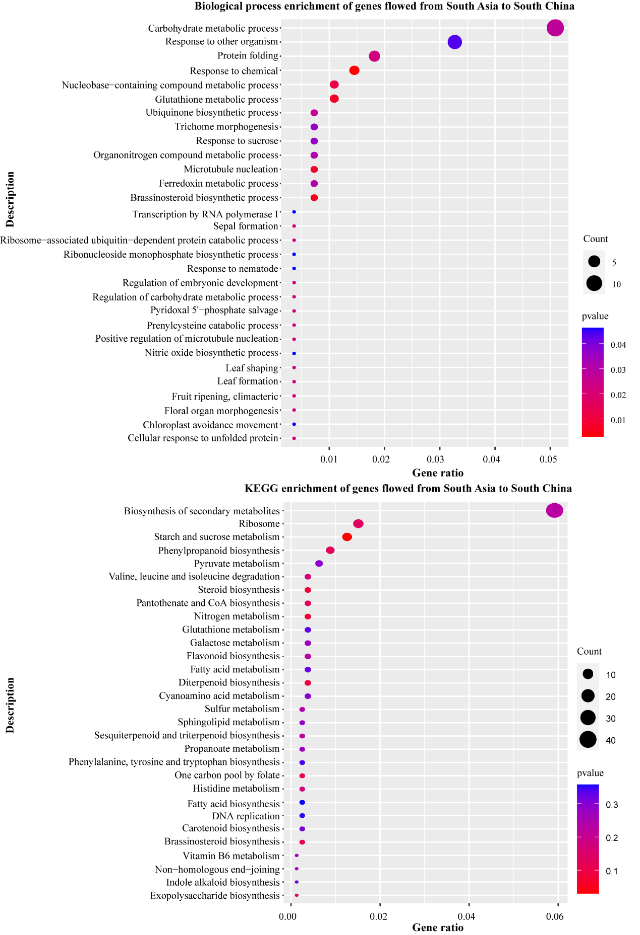
**Fig. S16. GO and KEGG enrichment for genes flowed from South Asia to North China. A:** GO enrichment for genes. **B:** KEGG enrichment for genes.

**Fig. S17. GO and KEGG enrichment for genes flowed from South Asia to South China. A:** GO enrichment for genes. **B:** KEGG enrichment for genes.

**
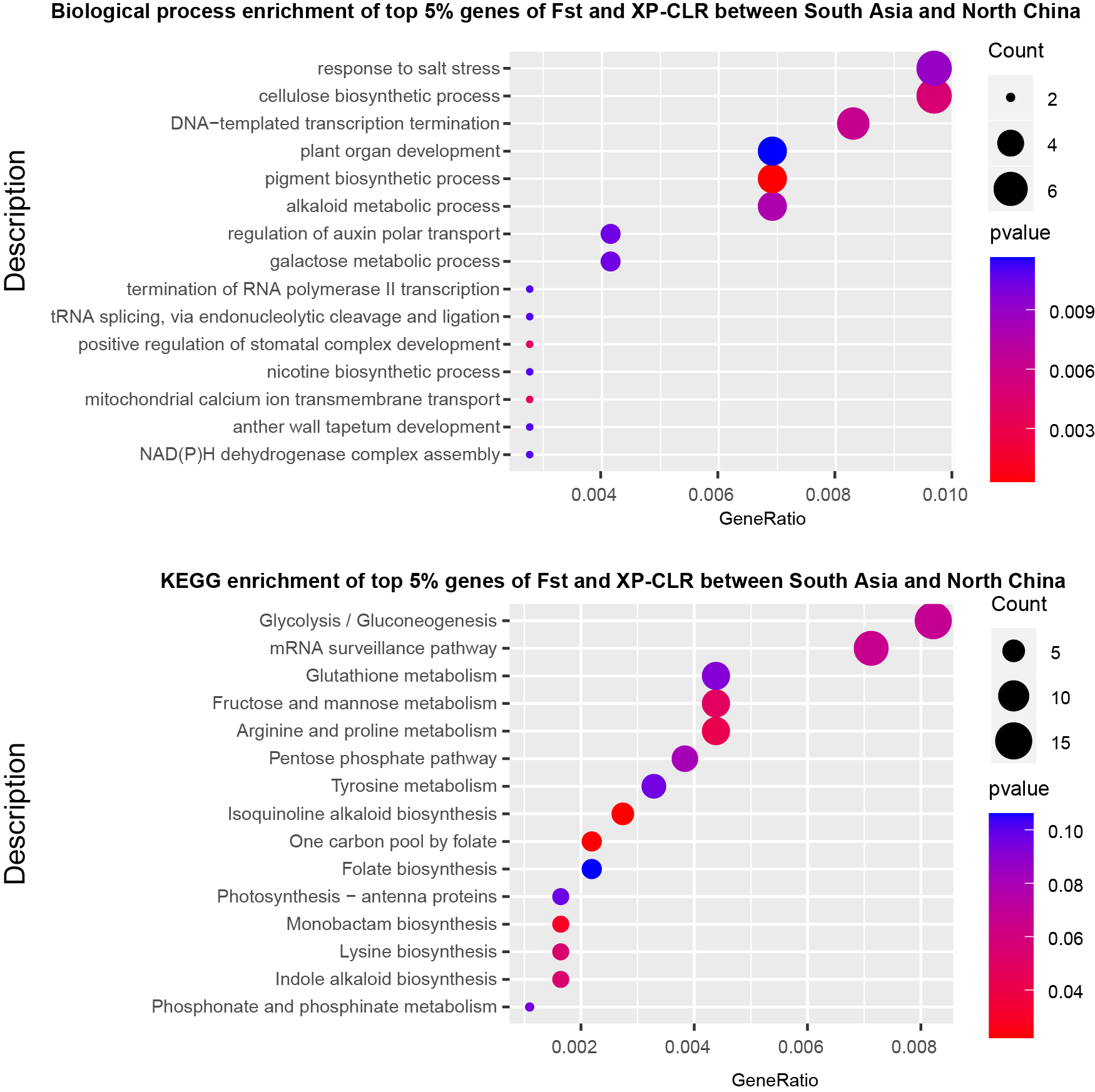
**

**Fig. S18. GO and KEGG enrichment of genes in high *F_ST_* regions between South Asia and North China. A:** GO enrichment for genes. **B:** KEGG enrichment for genes.


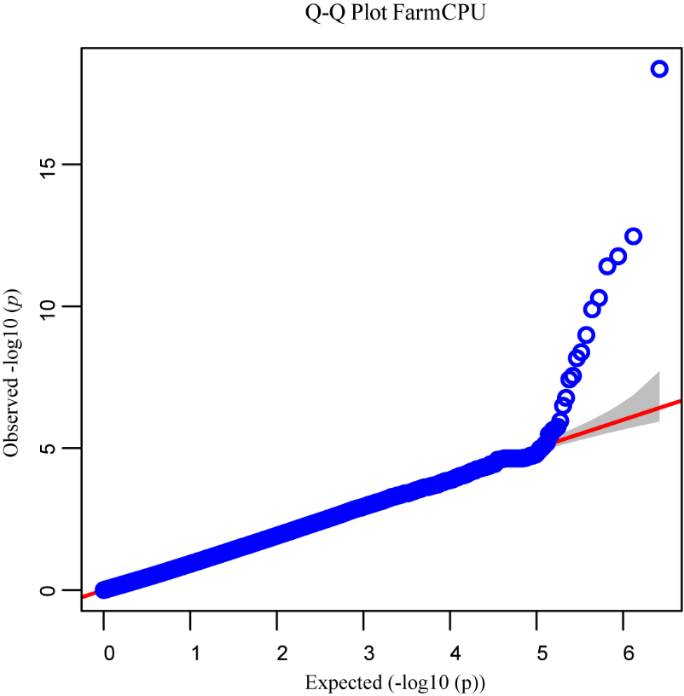


**Fig. S19. QQ plot for GWAS of flowering time trait among the 235 sesame accessions.** Phenotype data in 2022 is used.

**
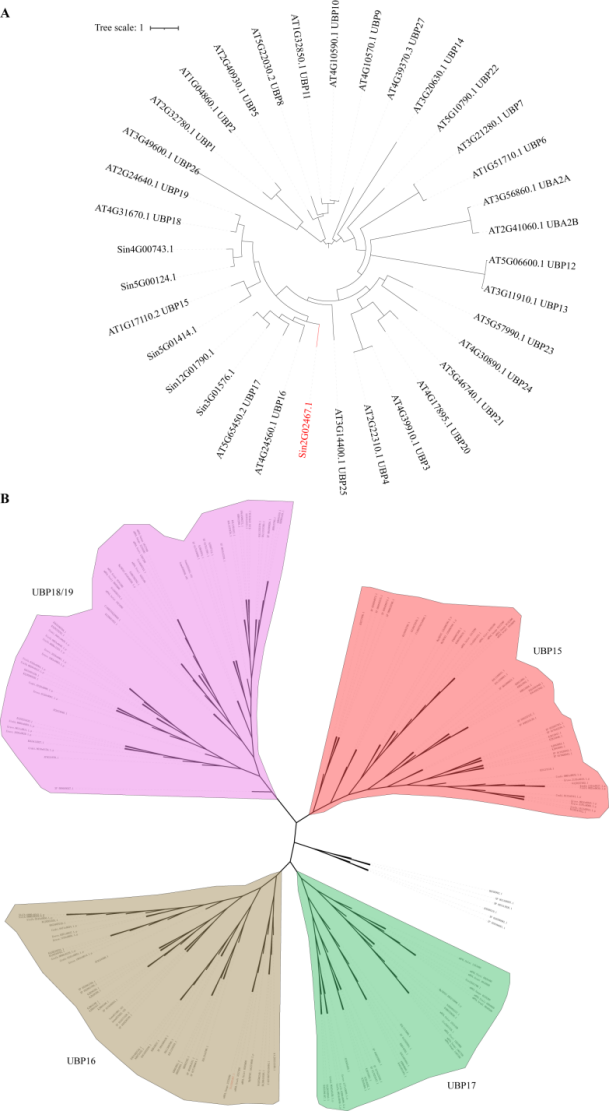
**

**Fig. S20. Phylogenetic tree of UBP from 27 species. A:** The phylogenetic tree of 6 *ZnF-UBP* genes from *S. indicum* var. Yuzhi11 T2T and all UBP gene family of *A. thaliana.* B: The phylogenetic tree of all *ZnF-UBP* genes from 27 species. Neighbor-joining (NJ) method with a bootstrap test (n=1000 replications) is used to construct the phylogenetic tree. *SiUBP16* (*Sin2G02467*) is shown in red color.


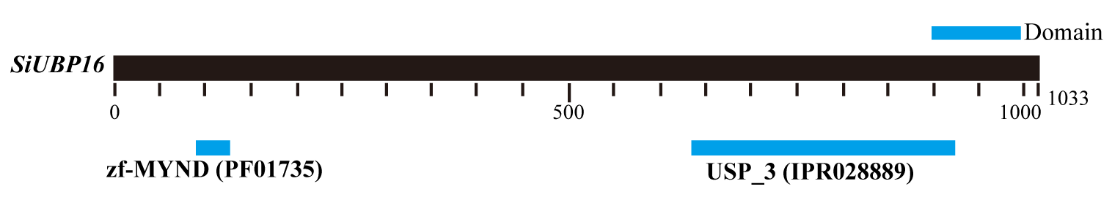


**Fig. S21. Domain composition overview for SiUBP16 protein.**


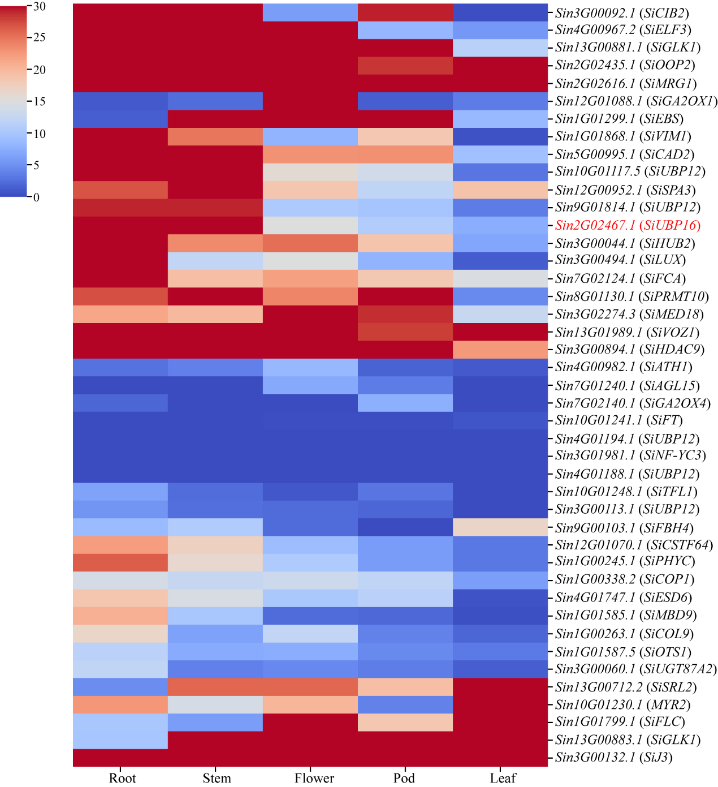


**Fig. S22. Expression of homologous genes regulating flowering time in different tissues of sesame cv. Yuzhi 11.** Root, stem, flower, pod and leaf tissues are assayed for sesame genes regulating flowering time.


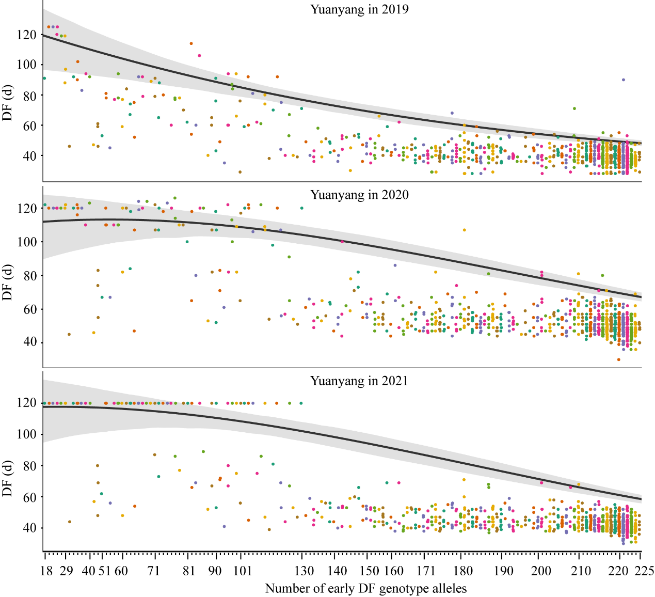


**Fig. S23. Relationship between the number of early DF genotype alleles and DF trait in sesame population.** DF indicates the days from sowing to flowering. Three group data of the 927 sesame accessions at Yuanyang experimental station in 2019, 2020, and 2021 year, respectively are shown in three figures.
