# Supplementary material for: Insights Into the Origin and Local Adaptation Evolution of the Cultivated Sesame With Telomere‐to‐Telomere High‐Quality Genome
Source: Plant Biotechnol J. 2026 Jul 4:10.1111/pbi.70714. Online ahead of print. doi: 10.1111/pbi.70714 (PMC13398907; doi:10.1111/pbi.70714)
Supplement: Supplementary file 2 — Table S1: Summary of sequencing data used for the genome assembly of S. indicum var. Yuzhi11. Table S2: Genome survey and assembly statistics. Table S3: Comparison of the T2T sesame genome with the published SiChr.omosome‐scaled sesame genomes. Table S4: Distribution of telomere region in T2T sesame genome. Table S5: Statistics of BUSCO and merqury evaluation of T2T sesame genome. Table S6: Statistics of annotated genes in T2T sesame genome using various protein databases. Table S7: Distribution of repeat sequences of T2T sesame genome. Table S8: Statistics of ncRNAs in T2T sesame genome. Table S9: Distribution of centromere regions predicted in T2T sesame genome. Table S10: Genes description affected by SV between Yuzhi11 and Baizhima. Table S11: Statistics of different modes of gene duplication in T2T sesame genome and sesame genome var. Baizhima. Table S12: Statistics of Ka/Ks for different modes of duplicated genes from T2T sesame genome and the genome var. Baizhima. Table S13: Statistics of genome resequencing data of 927 worldwide sesame accessions. Table S14: Summary of sample sizes by collection site and group. Table S15: Statistics of gene flow among the eight populations of 927 sesame accessions. Table S16: Statistics of DF trait of 927 sesame germplasm accessions in two positions for 4 years. Table S17: Phenotypic characteristics for flowering time in the 245 Sesamum indicum accessions planted in Yuanyang. Table S18: Genomic regions with high divergence between ‘North China’ and ‘South Asia’ group among the 90 sesame accessions. Table S19: Statistics of flowering date related genes subject to selection. Table S20: Candidate genes putatively associated with DF using GWAS method. Table S21: Gene flow intervals from ‘South Asia’ population into ‘North China’ population. Table S22: Number of genes keeping ZnF UBP domain identified in 27 species. Table S23: Prediction for PXLXP motif of homologous sesame genes to Arabidopsis thaliana regulating flowering time. [file PBI-9999-0-s001.doc]

| Table S1 Summary of sequencing data used for the genome assembly of S. indicum var. Yuzhi11. | |
| --- | --- |
| **1. Statistics of DNBseq data** | **clean data** |
| Sequence number | 275,480,956 |
| Total bases (bp) | 40,493,647,099 |
| GC content (%) | 37.45 |
| Q20 (%) | 97.74 |
| Q30 (%) | 94.62 |
| **2. Statistics of HiFi sequencing data** |  |
| Reads number | 1,782,480 |
| Total Bases (Gb) | 30.3 |
| HIFI read length (bp) | 16,999 |
| HIFI Read length N50 (bp) | 17,497 |
| **3. Statistics of Nanopore sequencing data** |  |
| Total base (bp) | 25,591,959,815 |
| Total reads number | 254,805 |
| Maximum length (bp) | 720,222 |
| Average length (bp) | 100,437 |
| N50 (bp) | 100,000 |
| L50 (bp) | 93,850 |
| N90 (bp) | 70,540 |
| L90 (bp) | 217,044 |
| Mean Q | 12.11 |
| **4. Statistics of Hi-C sequencing data** |  |
| Clean reads number | 260,052,430 |
| Clean bases (bp) | 38,435,115,515 |
| Q20 (%) | 97.01 |
| Q30 (%) | 93.24 |
| **5. Statistics of BioNano sequencing data** |  |
| Total clean data quantity (Gbp; ≥150 kbp. Minimum site ≥ 9) | 65.82 |
| Clean data Average label (/100Kbp) | 8.9 |
| Clean Data N50 (Mbp; ≥150 kbp. Minimum site ≥ 9) | 0.26 |
| Coverage | 215.8 |

| **Table S2 Genome survey and assembly statistics.** | |
| --- | --- |
| **Item** | **Index** |
| **1. Genome assembly estimation** |  |
| K-mer | 19 |
| K-mer number | 32,460,797,660 |
| K-mer depth | 105.71 |
| Heterozygous ratio (%) | 0.43 |
| Duplication ratio (%) | 39.51 |
| Sequencing depth (×) | 131.87 |
| Estimated T2T Genome size (Mb) | 307.07 |
| **2. Contig assembly** |  |
| Total contig number | 92 |
| Total contig lenth (bp) | 312,151,326 |
| Maxum contig length (bp) | 30,224,176 |
| ≥2000bp contig number | 92 |
| N50 number | 7 |
| N50 length (bp) | 20,095,418 |
| N90 number | 15 |
| N90 length (bp) | 11,026,715 |

| **Table S3 Comparison of the T2T sesame genome with the published SiChr.osome-scaled sesame genomes.** | | | | | | | | | | |
| --- | --- | --- | --- | --- | --- | --- | --- | --- | --- | --- |
| **Index** | **T2T genome (var. Yuzhi 11)** | **Genome v3.0 (var. Yuzhi 11) *** | **Genome (var. Baizhima)**** | **Qiongzhongbai***** | **EC34***** | **Dongyangmi***** | **Zhima8131***** | **Zhongzhi13***** | **Genome (var. Xiaozihei)****** | **S. Schinzianum****** |
| **Assembled bases (Mb)** | 305 | 312 | 309 | 318 | 304 | 321 | 320 | 306 | 305 | 721 |
| **Unplaced bases (Mb)** | 0 | 31.55 | 7.62 | 20 | 22 | 21 | 25 | 20 | - | - |
| **Gap number** | 0 | 1,717 | 35 | - | - | - | - | - | 288 | 432 |
| **Contig number** | 13 | 3,922 | 170 | - | - | - | - | - | 739 | 2159 |
| **Contig N50 (Mb)** | 23.89 | 1.32 | 13.48 | 6.83 | 5.10 | 5.82 | 4.79 | 6.03 | 6.40 | 7.63 |
| **GC (%)** | 35.6 | 35.55 | 35.44 | - | - | - | - | - | 35.93 | 34.63 |
| **Telomeres number** | 23 | - | 24 | - | - | - | - | - | - | - |
| **Centromeres number** | 13 | - | - | - | - | - | - | - | - | - |
| **Transcripts** | 42,781 | 31,462 | 24,345 | - | - | - | - | - | 25265 | 50,320 |
| **Coding protein number** | 31,063 | 31,462 | 24,345 | 28,574 | 28,919 | 28,922 | 28,410 | 28,615 | 25,265 | 50,320 |
| **TE content (%)** | 52.53 | 47.75 | 52.81 | - | - | - | - | - | 36.52 | 34.27 |
| **Genome BUSCO (Complete, %)** | 98.6 | 98.5 | 98.64 | 97.6 | 98.3 | 97.9 | 98.1 | 98.3 | 98.39 | 98.51 |
| **LAI** | 24.92 | 17.71 | 19.22 | - | - | - | - | - | 15 | 2 |
| **QV** | 46.1206 | 32.27 | - | - | - | - | - | - | 26 | 38 |
| **Error rate** | 0.0000244 | 5.92E-04 | - | - | - | - | - | - | - | - |
| **Completeness (%)** | 98.76 | 97.72 | - | - | - | - | - | - | - | - |
| **Gene BUSCO (%)** | 96.5 | 79.2 | 92.9 | 97.5 | 97.5 | 97.6 | 97.6 | 97.6 | - | - |
| **Mean gene length (bp)** | 3,997 | 2,619 | 3,421 | 3,011.09 | 3,045.91 | 3,021.51 | 3,063.76 | 3,024.08 | 3,112 | 3,182 |
| **Mean CDS length (bp)** | 1,215 | 1,310 | 1,206 | 1,180.92 | 1,200.70 | 1,182.88 | 1,196.36 | 1,207.30 | 1,151 | 1,216 |
| **Mean intron length (bp)** | 740 | 388 | 445 | 449.7 | 450.85 | 453.38 | 453.70 | 444.96 | 428 | 437 |
| * The SiChrosome-scaled sesame genome for var. Yuzhi 11 published by Miao et al., 2024. | | | | | | | | | | |
| **The SiChrosome-scaled sesame genome for var. Baizhima published by Wang et al,. 2022. | | | | | | | | | | |
| ***The SiChrosome-scaled sesame genome for var. Zhongzhi13, var.Dongyangmi, var.EC34, var.Qiongzhongbai, var.Zhima8131 published by Song et al,. 2023. | | | | | | | | | | |
| ****The SiChrosome-scaled sesame genome for var.Xiaozihei and *S. schinzianum* published Wang et al,. 2023. | | | | | | | | | | |

| **Table S4 Distribution of telomere region in T2T sesame genome.** | | | | | | |
| --- | --- | --- | --- | --- | --- | --- |
| **SiChr.osome no.** | **5' top of SiChr.osome** | | | **3' top of SiChr.osome** | | |
|  | **Telomere start (bp)** | **Telomere end (bp)** | **Telomere repeat number** | **Telomere start (bp)** | **Telomere end (bp)** | **Telomere repeat number** |
| SiChr.1 | 1 | 30,404 | 4,233 | 31,431,869 | 31,446,239 | 2,045 |
| SiChr.2 | 53 | 2,048 | 270 | 30,257,037 | 30,346,262 | 12,548 |
| SiChr.3 | 1 | 16,529 | 1,888 | 26,539,278 | 26,625,905 | 12,152 |
| SiChr.4 | 118 | 22,831 | 3,301 | 28,271,177 | 28,326,889 | 7,880 |
| SiChr.5 | 1 | 11,282 | 1,613 | 23,873,227 | 23,888,204 | 10,444 |
| SiChr.6 | 1 | 14,477 | 2,072 | 23,587,261 | 23,670,116 | 11,719 |
| SiChr.7 | 1 | 22,018 | 3,113 | 23,873,227 | 23,888,204 | 2,140 |
| SiChr.8 | 1 | 19,443 | 2,708 | 20,237,429 | 20,250,880 | 1,917 |
| SiChr.9 | 1 | 35,966 | 5,109 | 21,330,405 | 21,411,462 | 11,458 |
| SiChr.10 | 1,922 | 15,903 | 1,999 | 17,106,338 | 17,125,041 | 2,603 |
| SiChr.11 | - | - | - | 16,859,241 | 16,940,879 | 11,550 |
| SiChr.12 | - | - | - | 20,107,932 | 20,119,391 | 1,634 |
| SiChr.13 | - | - | - | 19,720,275 | 19,778,149 | 2,045 |
| Note: SiChr.11, 12, and 13 are specific SiChr.osomes with satelite fragments in sesame SiChr.osome group according to the SiChr.osome nomination method of Zhao et al. (2018). | | | | | | |

| **Table S5 Statistics of BUSCO and merqury evaluation of T2T sesame genome.** | | |
| --- | --- | --- |
| **Evaluation type** | **Number** | **Perentage (%)** |
| **1. BUSCO evaluation** |  |  |
| Complete BUSCOs (C) | 1,590 | 98.6 |
| Complete and single-copy BUSCOs (S) | 1,539 | 95.4 |
| Complete and duplicated BUSCOs (D) | 51 | 3.2 |
| Fragmented BUSCOs (F) | 12 | 0.7 |
| Missing BUSCOs (M) | 12 | 0.7 |
| Total BUSCO groups | 1,614 | / |
| **2. Merqury evaluation** |  |  |
| Solid k-mers in the assembly | 203,434,831 |  |
| Total solid k-mers in the read set | 205,982,879 |  |
| Completeness | 98.763 |  |
| QV | 46.1271 |  |
| error_rate | 2.44E-05 |  |

| **Table S6 Statistics of annotated genes in T2T sesame genome using various protein databases.** | | |
| --- | --- | --- |
| **Database** | **Annotated gene number** | **Percentage of total genes (%)** |
| Nr | 29,021 | 93.43 |
| Pfam | 21,089 | 67.89 |
| GO | 19,610 | 63.13 |
| eggNOG | 25,816 | 83.11 |
| KEGG | 12,072 | 38.86 |
| Swiss | 28,729 | 92.49 |
| Total annotated gene number | 29,078 | 93.61 |
| Total gene number | 31,063 | - |

| **Table S7 Distribution of repeat sequences of T2T sesame genome.** | | | |
| --- | --- | --- | --- |
| **Repeat detection** | **Number** | **Repeat length (bp)** | **Percentage of genome (%)** |
| RepeatMasker | 123,739 | 49,829,304 | 16.33 |
| ProteinMask | 66,823 | 20,833,379 | 6.83 |
| Denovo | 405,762 | 109,742,250 | 35.97 |
| Trf | 98,742 | 32,795,652 | 10.75 |
| Total | 343,440 | 162,015,452 | 53.11 |
| **TE repeat type** | **Number** | **Repeat length (bp)** | **Percentage of genome (%)** |
| **1. Class I type** |  | 65,528,067 | 21.48 |
| LTR/Gypsy | 107,521 | 52,573,372 | 17.23 |
| LTR/Copia | 53,573 | 22,571,792 | 7.4 |
| LTR/unknown | 12,092 | 5,623,048 | 1.84 |
| LINE | 4,345 | 3,679,456 | 1.21 |
| Others | 1,013 | 503,070 | 0.16 |
| **2. Class II type** |  | 83,022,827 | 27.21 |
| Tc1–Mariner | 77,032 | 31,599,412 | 10.36 |
| Mutator | 65,155 | 14,945,972 | 4.9 |
| MITE | 91,381 | 27,101,442 | 8.88 |
| CACTA | 3,580 | 1,891,086 | 0.62 |
| hAT | 72,825 | 17,602,682 | 5.77 |
| Helitron | 2,270 | 668,799 | 0.22 |
| Others | 28,708 | 7,063,179 | 2.32 |
| **3. Others** | 53,339 | 14,857,270 | 4.87 |
| **4. Unknown** | 23,490 | 6,818,195 | 2.23 |
| **Total** | 596,324 | 160,240,389 | 52.53 |

| **Table S8 Statistics of ncRNAs in T2T sesame genome.** | | | | | |
| --- | --- | --- | --- | --- | --- |
| **Class** | **Type** | **Copy number** | **Average length (bp)** | **Total length (bp)** | **Percentage of genome (%)** |
| miRNA | miRNA | 120 | 131.54 | 15785 | 0.00517 |
| tRNA | tRNA | 1072 | 73.39 | 78674 | 0.02579 |
| rRNA | 18S | 260 | 1409.09 | 366364 | 0.12009 |
|  | 28S | 235 | 3331.26 | 782847 | 0.25661 |
|  | 5.8S | 214 | 152.68 | 32673 | 0.01071 |
|  | 5S | 3380 | 111.8 | 377875 | 0.12387 |
| snRNA | CD-box | 177 | 107.78 | 19077 | 0.00625 |
|  | HACA-box | 38 | 130.11 | 4944 | 0.00162 |
|  | splicing | 141 | 143.75 | 20269 | 0.00664 |
| Total | | 5637 |  | 1698508 | 0.55675 |

| **Table S9 Distribution of centromere regions predicted in T2T sesame genome.** | | | |
| --- | --- | --- | --- |
| **SiChr.osome no.** | **Start postion (bp)** | **End position (bp)** | **Centromere region size (bp)** |
| SiChr. 1 | 7,066,542 | 8,083,760 | 1,017,218 |
| SiChr. 2 | 9,680,198 | 12,092,389 | 2,412,191 |
| SiChr. 3 | 10,921,195 | 14,018,442 | 3,097,247 |
| SiChr. 4 | 9,363,978 | 9,559,753 | 195,775 |
| SiChr. 5 | 10,146,749 | 10,645,184 | 498,435 |
| SiChr. 6 | 7,844,813 | 11,355,897 | 3,511,084 |
| SiChr. 7 | 9,700,593 | 13,101,956 | 3,401,363 |
| SiChr. 8 | 10,485,454 | 10,602,495 | 117,041 |
| SiChr. 9 | 4,928,070 | 4,970,721 | 42,651 |
| SiChr. 10 | 4,651,032 | 4,972,765 | 321,733 |
| SiChr. 11 | 2,606,117 | 3,262,885 | 656,768 |
| SiChr. 12 | 6,483,890 | 6,870,473 | 386,583 |
| SiChr. 13 | 3,929,733 | 5,859,700 | 1,929,967 |
| *Si-CEN1* sequence with 153bp repeat in lenth: TATACTAACTTGTAGTTATTATTACGCTTAGCAAATTTACCACACAAATTCTTGGGTCTAGACCAACGTTTGGGTCAAGTTTCGTCGAATTCTGAGAACGTTCAAAATTTAGCTGTTTTGCACGGTTTTATTAAAGCAAGTTTGTTTCCCTTT | | | |

| Table S10 Genes description affected by SV between Yuzhi11 and Baizhima. | | | |
| --- | --- | --- | --- |
| GeneID | Description | GeneID | Description |
| Sin10G00289.1 | Uncharacterized protein LOC105180174 isoform X1 | Sin5G00624.1 | Uncharacterized protein LOC105179931 |
| Sin10G00290.1 | Uncharacterized protein LOC105176992 | Sin5G00629.1 | Uncharacterized protein LOC105155873 |
| Sin10G00688.1 | Cytochrome c oxidase-assembly factor COX23, mitochondrial isoform X1 | Sin5G00630.1 | Lysine-specific demethylase REF6 isoform X2 |
| Sin10G00696.1 | Dirigent protein | Sin5G00631.1 | Uncharacterized protein LOC105178690 |
| Sin10G01776.1 | Uncharacterized protein LOC105180343 | Sin5G00632.1 | Uncharacterized protein LOC105179154 |
| Sin11G00138.1 | . | Sin5G00633.1 | Uncharacterized protein LOC105178970 |
| Sin11G00139.1 | Uncharacterized protein | Sin5G00645.1 | . |
| Sin11G00140.1 | Uncharacterized protein LOC105179646 | Sin5G00664.1 | Flowering time control protein FPA-like |
| Sin11G00149.1 | Uncharacterized protein LOC105179618 | Sin5G00665.1 | Flowering time control protein FPA-like |
| Sin11G00150.1 | Uncharacterized protein LOC105167557 | Sin5G00666.1 | Flowering time control protein FPA-like |
| Sin11G00151.1 | Uncharacterized protein LOC105179638 | Sin5G00667.1 | Probable hydroxyacylglutathione hydrolase 2, chloroplastic |
| Sin11G00153.1 | Uncharacterized protein LOC105179618 | Sin5G00668.1 | Uncharacterized protein LOC105180226 |
| Sin11G00154.1 | Uncharacterized protein LOC105155934 | Sin5G00669.1 | Flowering time control protein FPA-like |
| Sin11G00155.1 | Uncharacterized protein LOC105179618 | Sin5G00670.1 | Flowering time control protein FPA-like |
| Sin11G00156.1 | Uncharacterized protein LOC105159808 | Sin5G00671.1 | Probable hydroxyacylglutathione hydrolase 2, chloroplastic |
| Sin11G00157.1 | Uncharacterized protein LOC105179646 | Sin5G00683.1 | Adenylate isopentenyltransferase 5, chloroplastic-like |
| Sin11G00921.1 | Uncharacterized protein LOC105172306 | Sin5G00684.1 | Adenylate isopentenyltransferase 5, chloroplastic-like |
| Sin11G00944.1 | Uncharacterized protein LOC105165934 | Sin5G00690.1 | Adenylate isopentenyltransferase 5, chloroplastic-like |
| Sin11G00945.1 | . | Sin5G00691.1 | Bacterial surface antigen (D15) domain-containing protein |
| Sin11G01003.1 | non-specific serine/threonine protein kinase | Sin5G00692.1 | Nuclear pore complex protein NUP205 |
| Sin11G01010.2 | Ubiquitin thioesterase OTU | Sin5G00693.1 | Uncharacterized protein LOC105179437 |
| Sin11G01020.1 | Phosphoglycerate mutase-like protein AT74 | Sin5G00694.1 | Uncharacterized protein LOC110011984 isoform X3 |
| Sin12G00086.1 | Senescence-associated protein | Sin5G00722.1 | Uncharacterized protein LOC105179638 |
| Sin12G00087.1 | Uncharacterized protein | Sin5G00723.1 | Uncharacterized protein LOC105179618 |
| Sin12G00088.1 | Protein TAR1 | Sin5G00727.3 | Uncharacterized protein LOC105167557 |
| Sin12G00089.1 | Uncharacterized protein | Sin5G00730.1 | Uncharacterized protein LOC105167557 |
| Sin12G00090.1 | DUF4283 domain-containing protein | Sin5G00732.1 | Uncharacterized protein LOC105179618 |
| Sin12G00091.1 | Uncharacterized protein | Sin5G00733.1 | Uncharacterized protein LOC105179646 |
| Sin12G00092.1 | Uncharacterized protein | Sin5G00734.1 | Uncharacterized protein LOC105179618 |
| Sin12G00093.1 | Senescence-associated protein | Sin5G00735.1 | Uncharacterized protein LOC105179646 |
| Sin12G00094.1 | Uncharacterized protein | Sin5G00736.1 | Uncharacterized protein LOC105179931 |
| Sin12G00095.1 | Protein TAR1-like | Sin5G00739.1 | Uncharacterized protein LOC105167557 |
| Sin12G00096.1 | Uncharacterized protein | Sin5G00740.1 | Uncharacterized protein LOC105167557 |
| Sin12G00097.1 | Uncharacterized protein LOC105180093 | Sin5G00741.1 | Uncharacterized protein LOC105179638 |
| Sin12G00098.1 | DUF4283 domain-containing protein | Sin5G00742.1 | Uncharacterized protein LOC105179638 |
| Sin12G00099.1 | Uncharacterized protein | Sin5G00743.1 | Uncharacterized protein LOC105179646 |
| Sin12G00100.1 | Senescence-associated protein | Sin5G00744.1 | Uncharacterized protein LOC105179618 |
| Sin12G00101.1 | Senescence-associated protein | Sin5G00745.1 | Uncharacterized protein LOC105179638 |
| Sin12G00102.1 | Senescence-associated protein | Sin5G00746.1 | Uncharacterized protein LOC105179638 |
| Sin12G00103.1 | Senescence-associated protein | Sin5G00752.2 | Endocytosis protein RME-8, contains DnaJ domain |
| Sin12G00104.1 | Regulator of rDNA transcription protein 15 | Sin5G00753.1 | Uncharacterized protein LOC105179618 |
| Sin12G00105.1 | Regulator of rDNA transcription protein 15 | Sin5G00754.1 | Folylpolyglutamate synthase |
| Sin12G00176.1 | . | Sin5G00755.1 | Uncharacterized protein LOC105179931 |
| Sin12G00441.1 | ubiquitinyl hydrolase 1 | Sin5G00756.1 | Uncharacterized protein LOC110011363 |
| Sin12G00557.1 | Uncharacterized protein LOC105179618 | Sin5G00757.1 | DnaJ homolog subfamily C GRV2-like |
| Sin12G00558.1 | Uncharacterized protein LOC105167557 | Sin5G00758.1 | Uncharacterized protein LOC105167557 |
| Sin12G00559.1 | DNA-directed RNA polymerase | Sin5G00777.1 | Endocytosis protein RME-8, contains DnaJ domain |
| Sin12G00560.1 | Uncharacterized protein LOC105179618 | Sin5G00778.1 | Uncharacterized protein LOC105179931 |
| Sin12G00561.1 | Uncharacterized protein LOC105155263 | Sin5G00779.1 | Uncharacterized protein LOC105180082 |
| Sin12G00562.1 | Uncharacterized protein LOC105179618 | Sin5G00796.1 | Uncharacterized protein LOC110011363 |
| Sin12G00563.1 | Uncharacterized protein LOC105179618 | Sin5G00797.1 | Uncharacterized protein LOC105168593 |
| Sin12G00565.1 | Uncharacterized protein | Sin5G00814.1 | F-box protein At3g03040-like |
| Sin12G00566.2 | Uncharacterized protein LOC105179638 | Sin5G00816.1 | Autophagy-related protein 18a-like isoform X1 |
| Sin12G00569.1 | Uncharacterized protein LOC105179638 | Sin5G00817.1 | . |
| Sin12G00570.1 | Uncharacterized protein LOC105162405 | Sin5G00818.1 | ubiquitinyl hydrolase 1 |
| Sin12G00571.1 | Uncharacterized protein | Sin5G00819.1 | Photosystem II cytochrome b559 N-terminal domain-containing protein |
| Sin12G00574.1 | Uncharacterized protein LOC105179775 | Sin5G00820.1 | ATP-dependent Clp protease proteolytic subunit |
| Sin12G00575.1 | Uncharacterized protein LOC105179638 | Sin5G00821.1 | Small ribosomal subunit protein bS18c |
| Sin12G00576.1 | Uncharacterized protein LOC105162405 | Sin5G00822.1 | Large ribosomal subunit protein bL33c |
| Sin12G00577.1 | Uncharacterized protein LOC110011401 | Sin5G00823.1 | Cytochrome b559 subunit alpha |
| Sin12G00580.1 | Uncharacterized protein LOC105167557 | Sin5G00824.1 | Cytochrome f |
| Sin12G00581.1 | Uncharacterized protein LOC105179646 | Sin5G00825.1 | Photosystem i assembly protein ycf3 |
| Sin12G00582.1 | Uncharacterized protein LOC105179931 | Sin5G00847.1 | Uncharacterized protein LOC105179154 |
| Sin12G00584.1 | Uncharacterized protein LOC105179618 | Sin5G00848.1 | Uncharacterized protein LOC110011297 |
| Sin12G00588.1 | Uncharacterized protein LOC105167557 | Sin5G00849.1 | . |
| Sin12G00902.1 | Uncharacterized protein LOC110012108 isoform X1 | Sin5G00850.1 | Uncharacterized protein LOC105158731 |
| Sin12G00903.1 | Uncharacterized protein LOC110012108 isoform X1 | Sin5G00851.1 | . |
| Sin12G00904.1 | Uncharacterized protein LOC110012108 isoform X1 | Sin5G00852.1 | Uncharacterized protein LOC105167801 |
| Sin12G00905.1 | Uncharacterized protein LOC110012108 isoform X1 | Sin5G00853.1 | Uncharacterized protein LOC105178410 |
| Sin12G00919.1 | Uncharacterized protein LOC105178961 | Sin5G00869.1 | Uncharacterized protein LOC105158731 |
| Sin12G01166.1 | Uncharacterized protein LOC105176825 | Sin5G00883.1 | Uncharacterized protein LOC105162039 |
| Sin12G01167.1 | . | Sin5G00885.1 | GRF-type domain-containing protein |
| Sin12G01182.1 | Uncharacterized protein LOC110012124 | Sin5G00891.1 | Uncharacterized protein LOC105162617 |
| Sin12G01269.1 | . | Sin5G01049.1 | Retrotransposon protein |
| Sin12G01365.1 | Polygalacturonase-like | Sin5G01063.1 | Late embryogenesis abundant protein At1g64065-like |
| Sin12G01366.1 | . | Sin5G01064.1 | . |
| Sin12G01367.3 | Polygalacturonase (Fragment) | Sin5G01065.1 | . |
| Sin12G01377.1 | Uncharacterized protein LOC105155855 | Sin5G01109.1 | . |
| Sin12G01381.1 | S locus-related glycoprotein 1 binding pollen coat | Sin5G01291.3 | Uncharacterized protein LOC105160542 |
| Sin12G01721.1 | . | Sin5G01534.1 | Two-component response regulator ARR14-like |
| Sin12G01904.3 | AAA+ ATPase domain-containing protein | Sin5G01535.1 | Uncharacterized protein |
| Sin13G00257.1 | Uncharacterized protein LOC105162756 | Sin5G02605.1 | Serine/threonine-protein phosphatase 7 long form homolog |
| Sin13G00258.1 | Uncharacterized protein LOC110011493 | Sin6G00523.1 | DUF241 domain protein |
| Sin13G00259.1 | Uncharacterized protein LOC105179740 | Sin6G00525.1 | Uncharacterized protein LOC105155420 |
| Sin13G00260.1 | Retrotransposon gag domain-containing protein | Sin6G00526.1 | Uncharacterized protein LOC105155412 |
| Sin13G00261.1 | Zinc-finger domain-containing protein | Sin6G00527.1 | DUF241 domain protein |
| Sin13G00262.1 | Uncharacterized protein LOC110012326 | Sin6G00528.1 | Uncharacterized protein LOC105155420 |
| Sin13G00263.1 | Protein TAR1 | Sin6G00529.1 | DUF241 domain protein |
| Sin13G00266.2 | Uncharacterized protein LOC105172317 | Sin6G00530.1 | Uncharacterized protein LOC105155420 |
| Sin13G00270.1 | Uncharacterized protein LOC105178794 | Sin6G00531.1 | DUF241 domain protein |
| Sin13G00271.1 | Uncharacterized protein LOC105160173 | Sin6G00532.1 | Uncharacterized protein LOC105155420 |
| Sin13G00272.1 | Uncharacterized protein LOC105179796 | Sin6G00839.1 | Uncharacterized protein LOC105179638 |
| Sin13G00273.1 | peptidylprolyl isomerase | Sin6G00848.1 | Uncharacterized protein LOC105179638 |
| Sin13G00290.1 | Regulator of rDNA transcription protein 15 | Sin6G00851.1 | Uncharacterized protein LOC105179646 |
| Sin13G00504.1 | Uncharacterized protein LOC105179638 | Sin6G00852.1 | Uncharacterized protein LOC105179931 |
| Sin13G00612.1 | Homoserine dehydrogenase | Sin6G00854.1 | Uncharacterized protein LOC105179646 |
| Sin13G00613.1 | . | Sin6G00856.1 | Uncharacterized protein LOC105179646 |
| Sin13G00614.1 | phosphoglycerate mutase (2,3-diphosphoglycerate-independent) | Sin6G00857.1 | Uncharacterized protein LOC105167557 |
| Sin13G00617.1 | . | Sin6G00859.1 | Uncharacterized protein LOC105179618 |
| Sin13G00618.1 | Uncharacterized protein LOC105171199 | Sin6G00860.1 | Uncharacterized protein LOC105179618 |
| Sin13G00619.1 | Uncharacterized protein LOC105178702 | Sin6G00861.1 | Uncharacterized protein LOC105179638 |
| Sin13G00620.1 | Uncharacterized protein LOC105162210 isoform X2 | Sin6G00862.1 | Uncharacterized protein LOC105179638 |
| Sin13G00621.1 | Uncharacterized protein LOC105179909 | Sin6G00908.1 | Ferruginol synthase-like |
| Sin13G00622.1 | SKP1-like protein | Sin6G00909.1 | Uncharacterized protein LOC110011297 |
| Sin13G00623.1 | Uncharacterized protein LOC105168593 | Sin6G00910.1 | Ferruginol synthase-like |
| Sin13G00624.1 | Uncharacterized protein LOC105178794 | Sin6G00912.1 | Uncharacterized protein LOC110012122 |
| Sin13G00625.1 | Reticulon-like protein | Sin6G00927.1 | L10-interacting MYB domain-containing protein-like |
| Sin13G00626.1 | Uncharacterized protein LOC105179637 | Sin6G00943.1 | Protein MICRORCHIDIA 7-like |
| Sin13G00631.1 | Uncharacterized protein | Sin6G00944.1 | Uncharacterized protein LOC105155524 |
| Sin13G00632.1 | Uncharacterized protein LOC105178410 | Sin6G01628.1 | Annexin |
| Sin13G00633.1 | Uncharacterized protein LOC105170846 | Sin7G00156.1 | Organ-specific protein S2-like |
| Sin13G00634.1 | . | Sin7G00740.1 | . |
| Sin13G00639.1 | Lipase | Sin7G00741.1 | . |
| Sin13G00676.1 | Uncharacterized protein LOC105156135 | Sin7G00742.1 | Uncharacterized protein |
| Sin13G00679.1 | Glycosyltransferase | Sin7G00743.1 | Uncharacterized protein |
| Sin13G00686.1 | Zinc ion binding | Sin7G00744.1 | Uncharacterized protein |
| Sin13G00687.1 | Uncharacterized protein LOC105179352 | Sin7G00953.1 | Uncharacterized protein LOC105179618 |
| Sin13G00688.1 | Uncharacterized protein LOC105157440 | Sin7G00954.1 | Uncharacterized protein LOC105179638 |
| Sin13G00689.1 | Uncharacterized protein LOC105173307 isoform X1 | Sin7G00955.1 | Uncharacterized protein LOC105179638 |
| Sin13G00690.1 | Uncharacterized protein LOC110012108 isoform X1 | Sin7G00975.1 | Uncharacterized protein LOC105180082 |
| Sin13G00691.1 | Glycosyltransferase | Sin7G01461.1 | Disease resistance protein RPP8-like |
| Sin13G00696.1 | Uncharacterized protein LOC105178632 | Sin7G01466.1 | Probable disease resistance protein At1g58390 |
| Sin13G00774.1 | Uncharacterized protein LOC110011773 | Sin7G01488.1 | F-box protein PP2-B5 |
| Sin13G00775.1 | Uncharacterized protein LOC105157071 | Sin7G01489.1 | F-box protein PP2-B5 |
| Sin13G00776.1 | . | Sin7G01490.3 | protein-serine/threonine phosphatase |
| Sin13G00999.1 | 1,3-beta-glucan synthase | Sin7G01491.1 | Uncharacterized protein LOC105172056 |
| Sin13G01277.2 | Germacrene-D synthase | Sin7G01495.1 | Crescerin-1 |
| Sin13G01610.1 | Protein aspartic protease in guard cell 1 | Sin7G01508.1 | Probable disease resistance protein RF45 |
| Sin13G02083.1 | Uncharacterized protein LOC105155887 | Sin7G01510.1 | Pentatricopeptide repeat-containing protein At1g12700, mitochondrial |
| Sin13G02084.1 | . | Sin7G01511.1 | . |
| Sin13G02085.1 | Uncharacterized protein LOC105178794 | Sin7G01531.1 | Reverse transcriptase |
| Sin13G02086.1 | Uncharacterized protein LOC105164494 | Sin7G01532.1 | . |
| Sin13G02093.1 | Aquaporin NIP1-1-like | Sin7G01587.1 | Major allergen Pru ar 1-like |
| Sin1G00001.1 | Retrovirus-related Pol polyprotein from transposon TNT 1-94 | Sin7G01623.1 | Uncharacterized protein LOC105171744 |
| Sin1G00266.1 | Uncharacterized protein LOC110012124 | Sin7G01753.1 | 3-ketoacyl-CoA synthase |
| Sin1G00330.1 | Glycine-rich protein DOT1-like | Sin7G02139.1 | Uncharacterized protein LOC105157998 isoform X3 |
| Sin1G00817.1 | Uncharacterized protein | Sin8G00198.1 | Uncharacterized protein LOC105155934 |
| Sin1G00818.1 | Uncharacterized protein | Sin8G00225.1 | Uncharacterized protein |
| Sin1G00819.1 | Cytochrome c oxidase subunit 2 | Sin8G00638.1 | Uncharacterized protein LOC105179931 |
| Sin1G01061.1 | Uncharacterized protein LOC105178983 | Sin8G00682.1 | Uncharacterized protein LOC105179428 |
| Sin1G02138.1 | Uncharacterized protein LOC110011490 | Sin8G00683.1 | Uncharacterized protein LOC105179428 |
| Sin1G02697.1 | . | Sin8G00778.1 | Uncharacterized protein LOC105179638 |
| Sin1G02880.1 | . | Sin8G00779.1 | Uncharacterized protein LOC105179618 |
| Sin2G00373.1 | Uncharacterized protein LOC105156246 | Sin8G00784.1 | Uncharacterized protein LOC105179618 |
| Sin2G00544.1 | Uncharacterized protein LOC105158536 | Sin8G00785.1 | Uncharacterized protein LOC105179786 |
| Sin2G00545.1 | Uncharacterized protein LOC110012108 isoform X1 | Sin8G00786.1 | Uncharacterized protein LOC105179931 |
| Sin2G00546.1 | Uncharacterized protein LOC105173307 isoform X1 | Sin8G00787.1 | Uncharacterized protein LOC105162405 |
| Sin2G00547.1 | Uncharacterized protein LOC105157439 | Sin8G00788.1 | Uncharacterized protein LOC105167557 |
| Sin2G00554.1 | Uncharacterized protein LOC105157237 | Sin8G00789.1 | Uncharacterized protein LOC105179617 |
| Sin2G00592.1 | Heme-binding protein 2-like | Sin8G00790.1 | Uncharacterized protein LOC105167557 |
| Sin2G00640.1 | Late blight resistance protein homolog R1B-17 | Sin8G00791.1 | Uncharacterized protein LOC105179618 |
| Sin2G00641.1 | Uncharacterized protein LOC105158536 | Sin8G00792.1 | Uncharacterized protein LOC105167557 |
| Sin2G00933.1 | Uncharacterized protein LOC105179617 | Sin8G00793.1 | Uncharacterized protein LOC105167557 |
| Sin2G00934.1 | Uncharacterized protein LOC105167557 | Sin8G00794.1 | Uncharacterized protein LOC105179646 |
| Sin2G00935.1 | Uncharacterized protein LOC105167557 | Sin8G00795.1 | Uncharacterized protein LOC105179646 |
| Sin2G00936.1 | Uncharacterized protein LOC105179618 | Sin8G00796.1 | Uncharacterized protein LOC105179638 |
| Sin2G00937.1 | Uncharacterized protein LOC105179778 | Sin8G00826.1 | Uncharacterized protein LOC105162376 |
| Sin2G00938.1 | Uncharacterized protein LOC105179618 | Sin8G00827.1 | ATP-dependent RNA helicase glh-1-like |
| Sin2G00944.1 | Uncharacterized protein LOC105179618 | Sin8G00828.1 | protein O-GlcNAc transferase |
| **Sin2G01042.1** | **Auxin-responsive protein** | Sin8G00829.2 | Protein PXR1-like |
| Sin2G01251.1 | Disease resistance protein RGA1 | Sin8G00912.1 | . |
| Sin2G01351.1 | Uncharacterized protein LOC110011708 | Sin8G00913.1 | . |
| Sin2G01352.1 | Uncharacterized protein LOC110011708 | Sin8G00914.1 | Uncharacterized protein LOC105156328 |
| Sin2G01353.1 | Uncharacterized protein LOC110011708 | Sin8G00915.1 | Uncharacterized protein LOC105164512 |
| Sin2G01354.1 | Uncharacterized protein LOC110011708 | Sin8G00916.1 | Uncharacterized protein LOC105162210 isoform X1 |
| Sin2G01448.1 | LOW QUALITY PROTEIN: transcription factor 25-like | Sin8G00917.1 | Uncharacterized protein LOC105178690 |
| Sin2G01453.1 | . | Sin8G00918.1 | Uncharacterized protein LOC105173349 |
| Sin2G01454.1 | Uncharacterized protein LOC105179637 | Sin8G00934.1 | Serine/threonine-protein phosphatase 7 long form homolog |
| Sin2G01458.1 | non-specific serine/threonine protein kinase | Sin8G00935.1 | . |
| Sin2G01775.1 | Uncharacterized protein LOC105178410 | Sin8G00936.1 | Uncharacterized protein LOC105167668 isoform X1 |
| Sin2G01953.1 | . | Sin8G00937.1 | Uncharacterized protein LOC110011327 |
| Sin2G02108.1 | Uncharacterized protein LOC105177421 | Sin8G00948.1 | Uncharacterized protein LOC105170846 |
| Sin3G00116.1 | Probable carboxylesterase 15 | Sin8G00949.1 | Serine/threonine-protein phosphatase 7 long form homolog |
| Sin3G00487.1 | Uncharacterized protein LOC105170433 | Sin8G00950.1 | Uncharacterized protein LOC105170846 |
| Sin3G00517.1 | Uncharacterized protein LOC105179428 | Sin8G00956.1 | Uncharacterized protein LOC105162328 |
| Sin3G00521.1 | Uncharacterized protein LOC110012607 | Sin8G00957.1 | Plant intracellular Ras-group-related LRR 5-like |
| Sin3G00852.1 | Uncharacterized protein LOC105170882 | Sin8G00958.1 | . |
| Sin3G00912.3 | Uncharacterized protein LOC105170810 | Sin8G00965.1 | . |
| Sin3G00914.1 | Uncharacterized protein LOC105170812 | Sin8G00968.1 | . |
| Sin3G00915.1 | Cingulin-like | Sin8G00973.2 | Uncharacterized protein LOC105162272 |
| Sin3G02219.4 | Methyl esterase 3 | Sin8G00977.1 | Uncharacterized protein LOC105158214 isoform X2 |
| Sin4G01371.1 | Uncharacterized protein LOC105179638 | Sin8G00978.1 | Uncharacterized protein LOC110011965 |
| Sin4G01372.1 | Uncharacterized protein LOC105179618 | Sin8G00981.1 | Uncharacterized protein LOC105179327 |
| Sin4G01585.1 | Uncharacterized protein LOC105161888 | Sin8G00982.1 | Uncharacterized protein LOC105179328 |
| Sin4G01588.1 | . | Sin8G00983.1 | Uncharacterized protein LOC105179775 |
| Sin4G01589.1 | Uncharacterized protein LOC105155545 | Sin8G01007.1 | Uncharacterized protein LOC105158279 |
| Sin4G02327.1 | Uncharacterized protein LOC105163799 | Sin8G01008.1 | Uncharacterized protein LOC105178413 |
| Sin4G02328.4 | Mediator of RNA polymerase II transcription subunit 19a isoform X1 | Sin8G01012.1 | . |
| Sin5G00006.1 | Uncharacterized protein LOC105180350 | Sin8G01037.1 | Uncharacterized protein LOC110012122 |
| Sin5G00007.4 | Uncharacterized protein LOC105167896 isoform X3 | Sin8G01038.1 | Uncharacterized protein LOC105176501 |
| Sin5G00021.1 | Uncharacterized protein LOC110012092 | Sin8G01039.1 | Chromatin assembly factor 1 subunit FAS1-like |
| Sin5G00038.1 | L-ascorbate peroxidase 6 | Sin8G01224.1 | Uncharacterized protein LOC105167668 isoform X2 |
| Sin5G00057.1 | NAC domain-containing protein 7-like | Sin8G01225.1 | Uncharacterized protein LOC110011328 |
| Sin5G00058.1 | . | Sin9G00005.1 | Uncharacterized protein LOC105167896 isoform X3 |
| Sin5G00077.3 | Uncharacterized protein LOC105157862 isoform X1 | Sin9G00883.1 | Uncharacterized protein LOC105168593 |
| Sin5G00223.1 | Uncharacterized protein LOC110011327 | Sin9G00884.1 | Uncharacterized protein LOC105158094 |
| Sin5G00224.1 | Uncharacterized protein LOC105162617 | Sin9G00885.1 | Dirigent protein |
| Sin5G00432.1 | Uncharacterized protein LOC105162193 | Sin9G00886.1 | Uncharacterized protein LOC105158731 |
| Sin5G00433.1 | Uncharacterized protein LOC110012122 | Sin9G00887.1 | Uncharacterized protein LOC105158731 |
| Sin5G00434.1 | Uncharacterized protein LOC105155870 | Sin9G00888.1 | Uncharacterized protein LOC105179796 |
| Sin5G00435.1 | Uncharacterized protein LOC105155725 | Sin9G00889.1 | Uncharacterized protein LOC105180039 |
| Sin5G00550.1 | . | Sin9G00890.1 | Uncharacterized protein LOC110011682 |
| Sin5G00551.1 | Uncharacterized protein LOC105157073 | Sin9G00901.1 | DUF4283 domain-containing protein |
| Sin5G00562.1 | Uncharacterized protein LOC105158875 | Sin9G00902.1 | . |
| Sin5G00563.1 | Uncharacterized protein LOC110012108 isoform X1 | Sin9G01203.1 | . |
| Sin5G00564.1 | Retrotransposon gag domain-containing protein | Sin9G01204.1 | Uncharacterized protein LOC105160393 isoform X1 |
| Sin5G00568.1 | Uncharacterized protein (Fragment) | Sin9G01205.1 | . |
| Sin5G00570.1 | Uncharacterized protein LOC105157071 | Sin9G01206.1 | Probable inactive receptor kinase At4g23740 isoform X1 |
| Sin5G00571.1 | Uncharacterized protein LOC110011682 | Sin9G01207.1 | Uncharacterized protein LOC105160683 |
| Sin5G00572.1 | Uncharacterized protein LOC110012108 isoform X1 |  |  |

| **Table S11 Statistics of different modes of gene duplication in T2T sesame genome and sesame genome var. Baizhima.** | | |
| --- | --- | --- |
| **Duplication type** | **Number of gene pairs in T2T sesame genome** | **Number of gene pairs in sesame genome var. Baizhima** |
| WGD-pairs | 7,467 | 6,228 |
| TD-pairs | 1,074 | 1,302 |
| PD-pairs | 1,135 | 552 |
| TRD-pairs | 6,706 | 4,791 |
| DSD-pairs | 19,152 | 13,193 |

| **Table S12 Statistics of Ka/Ks for different modes of duplicated genes from T2T sesame genome and the genome var. Baizhima.** | | | | | | | | | | | | | | | |
| --- | --- | --- | --- | --- | --- | --- | --- | --- | --- | --- | --- | --- | --- | --- | --- |
| **Genome group** | **Gene 1** | **Gene 2** | **Ka** | **Ks** | **KaKs** | **P-Value** | **Duplication type** | **Genome group** | **Gene 1** | **Gene 2** | **Ka** | **Ks** | **KaKs** | **P-Value** | **Duplication type** |
| Yu11_T2T | Sin10G01426.1 | Sin12G00851.1 | 0.31 | 0.15 | 2.00 | 0.00 | DSD | Baizhima | Sesame00412.t1 | Sesame11259.t1 | 0.31 | 0.15 | 2.05 | 0.04 | DSD |
| Yu11_T2T | Sin11G00102.1 | Sin4G00009.1 | 0.06 | 0.02 | 3.07 | 0.03 | DSD | Baizhima | Sesame02273.t1 | Sesame18342.t1 | 0.58 | 0.51 | 1.12 | 0.00 | DSD |
| Yu11_T2T | Sin11G00177.2 | Sin9G00494.1 | 0.13 | 0.05 | 2.77 | 0.04 | DSD | Baizhima | Sesame02669.t1 | Sesame10068.t1 | 0.34 | 0.20 | 1.73 | 0.04 | DSD |
| Yu11_T2T | Sin11G00555.3 | Sin8G00800.1 | 0.67 | 0.50 | 1.34 | 0.03 | DSD | Baizhima | Sesame04289.t1 | Sesame04416.t1 | 0.08 | 0.03 | 2.36 | 0.01 | DSD |
| Yu11_T2T | Sin12G01083.2 | Sin13G01054.4 | 0.54 | 0.42 | 1.29 | 0.03 | DSD | Baizhima | Sesame08087.t1 | Sesame09961.t1 | 0.12 | 0.06 | 2.01 | 0.03 | DSD |
| Yu11_T2T | Sin12G01398.1 | Sin12G01400.1 | 0.07 | 0.03 | 2.55 | 0.00 | DSD | Baizhima | Sesame08513.t1 | Sesame15985.t1 | 0.48 | 0.25 | 1.98 | 0.00 | DSD |
| Yu11_T2T | Sin2G00276.1 | Sin8G00885.2 | 0.32 | 0.15 | 2.08 | 0.03 | DSD | Baizhima | Sesame18341.t1 | Sesame18343.t1 | 0.04 | 0.03 | 1.25 | 0.00 | DSD |
| Yu11_T2T | Sin2G00742.1 | Sin2G01101.1 | 0.08 | 0.03 | 2.36 | 0.01 | DSD | Baizhima | Sesame18348.t1 | Sesame18324.t1 | 0.26 | 0.15 | 1.73 | 0.05 | DSD |
| Yu11_T2T | Sin2G01668.1 | Sin2G01670.1 | 0.32 | 0.18 | 1.77 | 0.01 | DSD | Baizhima | Sesame19062.t1 | Sesame09999.t1 | 1.17 | 0.56 | 2.11 | 0.00 | DSD |
| Yu11_T2T | Sin2G02446.1 | Sin4G02516.1 | 0.44 | 0.19 | 2.29 | 0.00 | DSD | Baizhima | Sesame21471.t1 | Sesame22047.t1 | 0.07 | 0.03 | 2.25 | 0.05 | DSD |
| Yu11_T2T | Sin3G00438.1 | Sin4G01801.1 | 0.25 | 0.13 | 1.90 | 0.02 | DSD | Baizhima | Sesame13359.t1 | Sesame13361.t1 | 0.03 | 0.00 | 32004.60 | 0.03 | PD |
| Yu11_T2T | Sin3G01301.1 | Sin6G00334.1 | 0.35 | 0.22 | 1.58 | 0.05 | DSD | Baizhima | Sesame16627.t1 | Sesame16629.t1 | 0.24 | 0.10 | 2.29 | 0.02 | PD |
| Yu11_T2T | Sin4G01039.1 | Sin4G01873.1 | 0.13 | 0.06 | 2.11 | 0.02 | DSD | Baizhima | Sesame18793.t1 | Sesame18795.t1 | 0.20 | 0.11 | 1.85 | 0.02 | PD |
| Yu11_T2T | Sin4G01801.1 | Sin6G00960.1 | 0.35 | 0.18 | 1.92 | 0.01 | DSD | Baizhima | Sesame04101.t1 | Sesame04102.t1 | 0.18 | 0.10 | 1.76 | 0.01 | TD |
| Yu11_T2T | Sin5G00713.1 | Sin5G00693.1 | 0.06 | 0.00 | 2944.25 | 0.01 | DSD | Baizhima | Sesame04289.t1 | Sesame04414.t1 | 0.08 | 0.04 | 2.07 | 0.02 | TRD |
| Yu11_T2T | Sin5G00926.1 | Sin3G01898.3 | 1.06 | 0.87 | 1.21 | 0.02 | DSD | Baizhima | Sesame20925.t1 | Sesame22603.t1 | 0.02 | 0.01 | 2.45 | 0.04 | WGD |
| Yu11_T2T | Sin5G01057.1 | Sin8G00971.1 | 0.09 | 0.03 | 3.34 | 0.04 | DSD | Baizhima | Sesame23786.t1 | Sesame16629.t1 | 0.59 | 0.26 | 2.26 | 0.00 | WGD |
| Yu11_T2T | Sin6G00377.1 | Sin7G00738.1 | 0.61 | 0.53 | 1.16 | 0.00 | DSD | Baizhima | Sesame08513.t1 | Sesame15174.t1 | 0.44 | 0.30 | 1.44 | 0.05 | WGD |
| Yu11_T2T | Sin6G00807.1 | Sin5G00635.1 | 0.44 | 0.26 | 1.67 | 0.05 | DSD |  |  |  |  |  |  |  |  |
| Yu11_T2T | Sin6G01098.1 | Sin12G01261.1 | 0.62 | 0.29 | 2.11 | 0.02 | DSD |  |  |  |  |  |  |  |  |
| Yu11_T2T | Sin7G00700.1 | Sin7G00752.1 | 0.15 | 0.07 | 2.00 | 0.01 | DSD |  |  |  |  |  |  |  |  |
| Yu11_T2T | Sin7G00994.1 | Sin7G01134.1 | 0.09 | 0.04 | 2.19 | 0.03 | DSD |  |  |  |  |  |  |  |  |
| Yu11_T2T | Sin8G00694.1 | Sin2G00759.1 | 0.03 | 0.01 | 4.19 | 0.04 | DSD |  |  |  |  |  |  |  |  |
| Yu11_T2T | Sin9G00304.1 | Sin10G00635.1 | 0.13 | 0.08 | 1.62 | 0.05 | DSD |  |  |  |  |  |  |  |  |
| Yu11_T2T | Sin9G00880.1 | Sin9G00890.1 | 0.03 | 0.01 | 3.09 | 0.02 | DSD |  |  |  |  |  |  |  |  |
| Yu11_T2T | Sin5G01465.1 | Sin5G01467.1 | 0.28 | 0.12 | 2.24 | 0.03 | PD |  |  |  |  |  |  |  |  |
| Yu11_T2T | Sin7G00926.1 | Sin7G00932.1 | 0.16 | 0.03 | 4.67 | 0.01 | PD |  |  |  |  |  |  |  |  |
| Yu11_T2T | Sin7G01003.1 | Sin7G01012.1 | 0.09 | 0.02 | 4.49 | 0.02 | PD |  |  |  |  |  |  |  |  |
| Yu11_T2T | Sin1G02291.1 | Sin1G02292.1 | 0.17 | 0.09 | 1.82 | 0.01 | TD |  |  |  |  |  |  |  |  |
| Yu11_T2T | Sin5G00160.1 | Sin5G00161.1 | 0.04 | 0.01 | 3.33 | 0.03 | TD |  |  |  |  |  |  |  |  |
| Yu11_T2T | Sin5G02413.1 | Sin5G02414.1 | 0.68 | 0.43 | 1.59 | 0.03 | TD |  |  |  |  |  |  |  |  |
| Yu11_T2T | Sin1G00940.1 | Sin6G01033.1 | 0.41 | 0.26 | 1.60 | 0.01 | TRD |  |  |  |  |  |  |  |  |
| Yu11_T2T | Sin5G00810.1 | Sin12G00493.1 | 0.30 | 0.16 | 1.83 | 0.03 | TRD |  |  |  |  |  |  |  |  |
| Yu11_T2T | Sin9G00553.1 | Sin4G02214.1 | 0.69 | 0.21 | 3.38 | 0.00 | TRD |  |  |  |  |  |  |  |  |
| Yu11_T2T | Sin4G01086.1 | Sin10G00804.1 | 1.31 | 1.20 | 1.09 | 0.01 | TRD |  |  |  |  |  |  |  |  |
| Yu11_T2T | Sin1G00856.1 | Sin1G00931.1 | 0.17 | 0.06 | 2.85 | 0.01 | WGD |  |  |  |  |  |  |  |  |
| Yu11_T2T | Sin1G00918.1 | Sin8G00555.1 | 0.14 | 0.06 | 2.47 | 0.05 | WGD |  |  |  |  |  |  |  |  |
| Yu11_T2T | Sin10G01527.2 | Sin8G01777.1 | 0.21 | 0.13 | 1.64 | 0.02 | WGD |  |  |  |  |  |  |  |  |
| Yu11_T2T | Sin3G01102.1 | Sin4G02526.1 | 0.28 | 0.08 | 3.69 | 0.00 | WGD |  |  |  |  |  |  |  |  |

| **Table S13 Statistics of genome resequencing data of 927 worldwide sesame accessions** | | | | | | | | | | | | | | | | | | | |
| --- | --- | --- | --- | --- | --- | --- | --- | --- | --- | --- | --- | --- | --- | --- | --- | --- | --- | --- | --- |
| Sample | Clean Bases (Gbp) | Q30(%) | GC(%) | Mapping Rate (%) | Geography | Country | Classified group | Het_kmer(%)* | Het_SNP(%)** | Sample | Clean Bases (Gbp) | Q30(%) | GC(%) | Mapping Rate (%) | Geography | Country | Classified group | Het_kmer(%)* | Het_SNP(%)** |
| S01 | 5.46 | 93.58 | 37.27 | 94.08 | East Asia | China | G2 | 0.66 | 0.01 | S465 | 7.45 | 90.84 | 37.34 | 94.63 | East Asia | China | G1 | 0.45 | 0.02 |
| S02 | 13.31 | 93.53 | 36.99 | 98.79 | East Asia | China | G2 | 0.05 | 0.01 | S466 | 6.41 | 90.73 | 37.57 | 97.89 | East Asia | China | G1 | 0.23 | 0.02 |
| S03 | 7.59 | 92.56 | 37.26 | 97.43 | East Asia | China | G2 | 0.2 | 0.02 | S467 | 6.65 | 90.35 | 38.86 | 96.46 | East Asia | China | G1 | 0.32 | 0.02 |
| S04 | 5.87 | 93.39 | 37.67 | 97.34 | East Asia | China | G2 | 0.34 | 0.01 | S468 | 8.57 | 90.71 | 37.37 | 94.32 | East Asia | China | G2 | 0.2 | 0.02 |
| S05 | 4.97 | 93.31 | 38.75 | 93.8 | East Asia | China | G2 | - | 0.01 | S469 | 6.17 | 90.51 | 37.91 | 97.46 | East Asia | China | G1 | 0.25 | 0.02 |
| S06 | 6.09 | 93.51 | 37.58 | 98.09 | East Asia | China | G2 | 0.21 | 0.02 | S470 | 4.63 | 90.38 | 37.6 | 95.63 | East Asia | China | G1 | - | 0.02 |
| S07 | 7.99 | 93.61 | 38.15 | 96.2 | East Asia | China | G2 | 0.47 | 0.01 | S471 | 5.55 | 90.92 | 37.06 | 98.36 | East Asia | China | G1 | 0.26 | 0.03 |
| S08 | 3.58 | 94.25 | 37.47 | 96.68 | East Asia | China | G1 | - | 0.01 | S472 | 5.99 | 90.12 | 37.16 | 97.37 | East Asia | China | G2 | 0.23 | 0.01 |
| S09 | 4.34 | 93.81 | 38.33 | 96.85 | East Asia | China | G2 | - | 0.01 | S473 | 5.21 | 90.73 | 37.77 | 95.77 | East Asia | China | G1 | 0.54 | 0.02 |
| S10 | 4.04 | 93.87 | 37.89 | 97.59 | East Asia | China | G2 | 0.76 | 0.01 | S474 | 5.08 | 90.78 | 37.39 | 98.42 | East Asia | China | G1 | 0.33 | 0.02 |
| S11 | 12.39 | 93.65 | 37.2 | 98.23 | East Asia | China | G2 | 0.12 | 0.01 | S475 | 5.67 | 91.33 | 37.45 | 96.98 | East Asia | China | G1 | 0.4 | 0.02 |
| S12 | 4.37 | 92.91 | 37.61 | 97.89 | East Asia | China | G2 | 0.51 | 0.01 | S476 | 5.65 | 90.75 | 37.23 | 98.03 | East Asia | China | G1 | 0.25 | 0.02 |
| S13 | 12.59 | 93.27 | 37.24 | 97.63 | East Asia | China | G2 | 0.1 | 0.01 | S477 | 5.24 | 89.7 | 37.5 | 94.89 | East Asia | China | G2 | - | 0.01 |
| S14 | 5.24 | 93.56 | 37.53 | 97.7 | East Asia | China | G2 | 0.48 | 0.01 | S478 | 5.43 | 90.09 | 37.38 | 95.64 | East Asia | China | G1 | 0.4 | 0.01 |
| S15 | 6.2 | 93.96 | 37.38 | 97.85 | East Asia | China | G2 | 0.51 | 0.01 | S479 | 4.79 | 89.34 | 37.45 | 94.69 | East Asia | China | G1 | - | 0.02 |
| S16 | 5.16 | 93.97 | 37.08 | 97.97 | East Asia | China | G2 | 0.65 | 0.06 | S480 | 6.47 | 90 | 37.48 | 93.29 | East Asia | China | G1 | 0.52 | 0.02 |
| S17 | 5.54 | 93.19 | 37 | 98.28 | East Asia | China | G2 | 0.32 | 0.01 | S481 | 7.95 | 89.98 | 37.59 | 97.79 | East Asia | China | G1 | 0.17 | 0.02 |
| S18 | 3.52 | 93.18 | 36.72 | 98.8 | East Asia | China | G2 | 0.62 | 0.01 | S482 | 6.26 | 90.71 | 37.72 | 96.3 | East Asia | China | G1 | 0.41 | 0.02 |
| S19 | 9.45 | 93.93 | 37.5 | 98.44 | East Asia | China | G2 | 0.18 | 0.01 | S483 | 5.37 | 90.37 | 37.41 | 97.51 | East Asia | China | G2 | 0.59 | 0.04 |
| S20 | 9.93 | 93.42 | 37.2 | 98.37 | East Asia | China | G2 | 0.13 | 0.01 | S484 | 4.76 | 90.66 | 39.82 | 95.05 | East Asia | China | G1 | - | 0.01 |
| S21 | 11.66 | 93.88 | 37.04 | 98.85 | East Asia | China | G2 | 0.11 | 0.01 | S485 | 7.54 | 90.75 | 38.05 | 97.71 | East Asia | China | G1 | 0.24 | 0.02 |
| S22 | 6.92 | 93.53 | 37.86 | 97.55 | East Asia | China | G2 | 0.49 | 0.02 | S486 | 5.5 | 90.31 | 37.2 | 97.27 | East Asia | China | G1 | 0.35 | 0.02 |
| S23 | 6.44 | 93.37 | 37.52 | 97.84 | East Asia | China | G2 | 0.33 | 0.01 | S487 | 6.46 | 90.34 | 37.6 | 84.94 | East Asia | China | G2 | - | 0.01 |
| S24 | 8.29 | 93.36 | 37.89 | 97.84 | East Asia | China | G2 | 0.22 | 0.01 | S488 | 6.14 | 90.52 | 37.75 | 97.98 | East Asia | China | G1 | 0.23 | 0.02 |
| S25 | 6.14 | 93.72 | 37.38 | 97.94 | East Asia | China | G2 | 0.39 | 0.01 | S489 | 6.42 | 89.88 | 37.65 | 96.09 | East Asia | China | G1 | 0.32 | 0.01 |
| S26 | 6.64 | 93.69 | 37.98 | 98 | East Asia | China | G2 | 0.35 | 0.01 | S490 | 5.1 | 89.61 | 37.59 | 92.86 | East Asia | China | G1 | - | 0.01 |
| S27 | 10.13 | 93.91 | 37.73 | 98.38 | East Asia | China | G2 | 0.16 | 0.01 | S491 | 6.45 | 90.84 | 38.28 | 92.21 | East Asia | China | G1 | - | 0.02 |
| S28 | 5.5 | 93.71 | 37.36 | 98.16 | East Asia | China | G2 | 0.37 | 0.01 | S492 | 6.68 | 89.89 | 37.79 | 95.82 | East Asia | China | G1 | 0.37 | 0.02 |
| S29 | 8.82 | 93.83 | 37.48 | 97.64 | East Asia | China | G2 | 0.27 | 0.01 | S493 | 6.08 | 90.59 | 37.53 | 97.53 | East Asia | China | G1 | 0.29 | 0.01 |
| S30 | 6.23 | 93.83 | 37.95 | 96.61 | East Asia | China | G2 | - | 0.01 | S494 | 8.1 | 90.35 | 37.96 | 97 | East Asia | China | G1 | 0.25 | 0.02 |
| S31 | 7.14 | 94.16 | 37.44 | 97.57 | East Asia | China | G2 | 0.38 | 0.02 | S495 | 7.11 | 90.28 | 37.48 | 97.25 | East Asia | China | G1 | 0.23 | 0.02 |
| S32 | 7.05 | 93.39 | 37.86 | 96.24 | East Asia | China | G2 | 0.46 | 0.01 | S496 | 7.42 | 90.39 | 38.05 | 96.37 | East Asia | China | G1 | 0.37 | 0.01 |
| S33 | 6.56 | 93.94 | 37.61 | 97.29 | East Asia | China | G2 | 0.58 | 0.01 | S497 | 5.1 | 90.56 | 38.29 | 92.92 | East Asia | China | G1 | - | 0.01 |
| S34 | 4.54 | 93.78 | 37.8 | 94.97 | East Asia | China | G2 | - | 0.01 | S498 | 5.67 | 90.39 | 37.8 | 95.24 | East Asia | China | G1 | 0.64 | 0.02 |
| S35 | 5.16 | 93.34 | 37.66 | 97.16 | East Asia | China | G2 | 0.66 | 0.01 | S499 | 6.56 | 90.72 | 37.41 | 96.09 | East Asia | China | G2 | 0.3 | 0.01 |
| S36 | 6.24 | 93.12 | 37.81 | 95.43 | East Asia | China | G1 | - | 0.02 | S500 | 4.78 | 90.06 | 37.58 | 92.67 | East Asia | China | G1 | - | 0.01 |
| S37 | 5.33 | 93.83 | 37.7 | 96.31 | East Asia | China | G2 | 0.73 | 0.02 | S501 | 5.79 | 90.84 | 37.45 | 98.01 | East Asia | China | G1 | 0.33 | 0.02 |
| S38 | 5.39 | 93.81 | 38.17 | 85.77 | East Asia | China | G1 | - | 0.02 | S502 | 5.55 | 90.57 | 37.34 | 98.14 | East Asia | China | G1 | 0.22 | 0.02 |
| S39 | 6.82 | 93.88 | 37.59 | 96.5 | East Asia | China | G1 | 0.61 | 0.03 | S503 | 6.84 | 90.55 | 37.36 | 96.48 | East Asia | China | G1 | 0.24 | 0.02 |
| S40 | 5.01 | 93.42 | 37.46 | 97.91 | East Asia | China | G2 | 0.57 | 0.01 | S504 | 4.69 | 90.03 | 37.7 | 97.89 | East Asia | China | G1 | 0.53 | 0.04 |
| S41 | 5.87 | 93.9 | 37.55 | 97.91 | East Asia | China | G3 | 0.42 | 0.02 | S505 | 6.15 | 90.78 | 37.92 | 97.14 | East Asia | China | G1 | 0.31 | 0.02 |
| S42 | 5.3 | 93.47 | 38.03 | 96.83 | East Asia | China | G2 | - | 0.01 | S506 | 5.08 | 90.69 | 37.41 | 98.09 | East Asia | China | G1 | 0.47 | 0.03 |
| S43 | 3.99 | 93.81 | 38 | 97.16 | East Asia | China | G2 | - | 0.01 | S507 | 5.52 | 89.9 | 37.96 | 98.05 | East Asia | China | G1 | 0.27 | 0.02 |
| S44 | 4.93 | 93.9 | 37.83 | 96.7 | East Asia | China | G2 | - | 0.01 | S508 | 5.16 | 89.91 | 37.84 | 97.49 | East Asia | China | G1 | 0.59 | 0.05 |
| S45 | 5.27 | 93.78 | 36.91 | 98.72 | East Asia | China | G2 | 0.37 | 0.02 | S509 | 5.06 | 90.34 | 37.72 | 97.97 | East Asia | China | G2 | 0.4 | 0.02 |
| S46 | 5.92 | 93.87 | 37.45 | 97.32 | East Asia | China | G2 | 0.53 | 0.01 | S510 | 6.99 | 91 | 37.59 | 97.52 | East Asia | China | G3 | 0.32 | 0.02 |
| S47 | 4.42 | 94.03 | 37.64 | 97.5 | East Asia | China | G2 | 0.7 | 0.01 | S511 | 4.9 | 90.49 | 37.45 | 97.31 | East Asia | China | G3 | 0.63 | 0.01 |
| S48 | 4.08 | 93.52 | 37.34 | 97.93 | East Asia | China | G2 | 0.66 | 0.01 | S512 | 5.35 | 90.45 | 37.21 | 97.59 | East Asia | China | G2 | 0.5 | 0.02 |
| S49 | 5.41 | 93.9 | 37.45 | 97.62 | East Asia | China | G2 | 0.57 | 0.01 | S513 | 5.78 | 90.34 | 38.3 | 97.81 | East Asia | China | G2 | 0.35 | 0.02 |
| S50 | 4.16 | 93.31 | 37.69 | 93.23 | East Asia | China | G2 | - | 0.01 | S514 | 4.86 | 90.43 | 37.68 | 97.48 | East Asia | China | G2 | 0.5 | 0.02 |
| S51 | 6.11 | 93.43 | 37.75 | 97.38 | East Asia | China | G2 | 0.25 | 0.02 | S515 | 4.66 | 90.57 | 37.19 | 98.18 | East Asia | China | G2 | 0.42 | 0.01 |
| S52 | 4.71 | 92.97 | 37.81 | 97.29 | East Asia | China | G2 | 0.69 | 0.01 | S516 | 5.67 | 90.34 | 37.36 | 97.58 | East Asia | China | G3 | 0.47 | 0.02 |
| S53 | 5.5 | 93.16 | 37.62 | 97.53 | East Asia | China | G2 | 0.54 | 0.02 | S517 | 5.7 | 91.16 | 36.99 | 98.47 | East Asia | China | G3 | 0.25 | 0.02 |
| S54 | 11.56 | 93.38 | 38.27 | 90.21 | East Asia | China | G2 | 0.46 | 0.02 | S518 | 5.66 | 90.59 | 37.39 | 98.05 | East Asia | China | G3 | 0.24 | 0.02 |
| S55 | 4.79 | 93.29 | 37.91 | 97.65 | East Asia | China | G2 | 0.71 | 0.01 | S519 | 6.21 | 89.97 | 37.51 | 98.04 | East Asia | China | G2 | 0.17 | 0.02 |
| S56 | 4.94 | 92.91 | 38.2 | 97.18 | East Asia | China | G2 | 0.71 | 0.01 | S520 | 4.52 | 90.77 | 37.55 | 97.87 | East Asia | China | G2 | 0.6 | 0.01 |
| S57 | 7.2 | 93.19 | 38.44 | 97.1 | East Asia | China | G2 | 0.5 | 0.01 | S521 | 4.42 | 89.91 | 37.47 | 97.28 | East Asia | China | G3 | - | 0.01 |
| S58 | 5.7 | 92.88 | 37.97 | 96.03 | East Asia | China | G1 | - | 0.01 | S522 | 7.64 | 90.94 | 37.7 | 97.36 | East Asia | China | G2 | 0.21 | 0.01 |
| S59 | 6.58 | 93.07 | 38.73 | 95.85 | East Asia | China | G1 | 0.76 | 0.02 | S523 | 6.56 | 90.78 | 37.1 | 98.02 | East Asia | China | G2 | 0.21 | 0.01 |
| S60 | 11.63 | 92.66 | 38.36 | 96.31 | East Asia | China | G1 | 0.21 | 0.02 | S524 | 6.49 | 90.88 | 37.86 | 96.69 | East Asia | China | G3 | 0.35 | 0.02 |
| S61 | 9.12 | 92.98 | 37.96 | 97.39 | East Asia | China | G2 | 0.34 | 0.07 | S525 | 5.22 | 90.44 | 37.63 | 95.68 | East Asia | China | G3 | 0.61 | 0.02 |
| S62 | 6.16 | 93.27 | 38.37 | 96.34 | East Asia | China | G2 | 0.7 | 0.02 | S526 | 5.16 | 90.37 | 38.16 | 94.7 | East Asia | China | G3 | - | 0.01 |
| S63 | 5.99 | 93.23 | 38.14 | 97.06 | East Asia | China | G1 | 0.59 | 0.02 | S527 | 7.25 | 90.84 | 37.5 | 97.11 | East Asia | China | G3 | 0.2 | 0.01 |
| S64 | 6.21 | 93 | 38.88 | 93.09 | East Asia | China | G1 | - | 0.02 | S528 | 5.1 | 90.23 | 38.49 | 94.44 | East Asia | China | G3 | - | 0.01 |
| S65 | 8.05 | 93.02 | 38.02 | 97.17 | East Asia | China | G1 | 0.34 | 0.02 | S529 | 6.83 | 90.58 | 37.5 | 97.04 | East Asia | China | G3 | 0.29 | 0.01 |
| S66 | 7.86 | 92.92 | 38.23 | 96.91 | East Asia | China | G1 | 0.46 | 0.03 | S530 | 5.14 | 90.16 | 37.7 | 93.96 | East Asia | China | G3 | - | 0.05 |
| S67 | 14.24 | 93.25 | 38.06 | 93.7 | East Asia | China | G2 | 0.26 | 0.01 | S531 | 4.55 | 90.64 | 37.5 | 96.55 | East Asia | China | G3 | - | 0.01 |
| S68 | 4.46 | 93.19 | 38.73 | 93.94 | East Asia | China | G1 | - | 0.01 | S532 | 4.62 | 90.42 | 38.1 | 91.21 | East Asia | China | G3 | - | 0.02 |
| S69 | 6.15 | 93.12 | 37.86 | 94.32 | East Asia | China | G1 | 1.06 | 0.02 | S533 | 4.56 | 89.98 | 37.02 | 97.24 | East Asia | China | G3 | 0.49 | 0.01 |
| S70 | 6.31 | 93.25 | 37.96 | 96.91 | East Asia | China | G2 | 0.61 | 0.01 | S534 | 4.78 | 90.13 | 37.64 | 95.95 | East Asia | China | G2 | - | 0.01 |
| S71 | 4.51 | 92.63 | 37.78 | 97.28 | East Asia | China | G3 | 0.68 | 0.02 | S535 | 3.92 | 89.56 | 37.59 | 93.23 | East Asia | China | G3 | - | 0.02 |
| S72 | 5.06 | 92.66 | 37.8 | 97.45 | East Asia | China | G2 | 0.59 | 0.02 | S536 | 9.08 | 90.25 | 38.5 | 97.01 | East Asia | China | G3 | 0.25 | 0.01 |
| S73 | 5.25 | 93.53 | 37.51 | 98.01 | East Asia | China | G2 | 0.54 | 0.01 | S537 | 6.99 | 89.94 | 37.7 | 96.22 | East Asia | China | G3 | 0.27 | 0.02 |
| S74 | 5.89 | 93.19 | 38.28 | 97.27 | East Asia | China | G3 | 0.7 | 0.01 | S538 | 6.17 | 90.65 | 37.67 | 97.72 | East Asia | China | G3 | 0.26 | 0.02 |
| S75 | 5.43 | 92.69 | 37.77 | 97.56 | East Asia | China | G3 | 0.5 | 0.02 | S539 | 5.9 | 90.59 | 37.91 | 94.69 | East Asia | China | G2 | 0.7 | 0.02 |
| S76 | 4.96 | 93.14 | 37.93 | 97.72 | East Asia | China | G3 | 0.69 | 0.02 | S540 | 6.57 | 90.41 | 37.86 | 96.17 | East Asia | China | G3 | 0.49 | 0.01 |
| S77 | 6.5 | 92.81 | 37.96 | 95.73 | East Asia | China | G3 | 0.72 | 0.03 | S541 | 6.46 | 90.73 | 37.98 | 96.67 | East Asia | China | G2 | 0.38 | 0.02 |
| S78 | 6.23 | 93.42 | 37.97 | 98.42 | East Asia | China | G2 | 0.43 | 0.02 | S542 | 5.63 | 91.42 | 37.84 | 92.78 | East Asia | China | G3 | - | 0.01 |
| S79 | 5.59 | 93.35 | 37.69 | 97.75 | East Asia | China | G2 | 0.55 | 0.02 | S543 | 5.73 | 90.55 | 37.94 | 93.65 | East Asia | China | G2 | - | 0.02 |
| S80 | 5.91 | 92.95 | 38.28 | 92.56 | East Asia | China | G3 | 1.13 | 0.02 | S544 | 5.88 | 90.47 | 37.99 | 95.88 | East Asia | China | G3 | - | 0.02 |
| S81 | 4.52 | 92.9 | 37.95 | 97.47 | South Asia | India | G3 | - | 0.01 | S545 | 7.35 | 90.65 | 37.85 | 93.32 | East Asia | China | G2 | 0.72 | 0.02 |
| S82 | 5 | 93.1 | 38.08 | 96.72 | South Asia | India | G3 | - | 0.02 | S546 | 5.07 | 90.28 | 37.36 | 97.36 | East Asia | China | G3 | 0.48 | 0.02 |
| S83 | 7.89 | 93.24 | 38 | 95.2 | South Asia | India | G3 | - | 0.01 | S547 | 4.86 | 91.06 | 37.98 | 96.93 | East Asia | China | G3 | - | 0.02 |
| S84 | 5.21 | 93.08 | 38.47 | 96.16 | South Asia | India | G2 | - | 0.01 | S548 | 5 | 91 | 37.57 | 97.4 | East Asia | China | G3 | 0.58 | 0.02 |
| S85 | 4.47 | 93.11 | 37.66 | 97.94 | South Asia | India | G3 | 0.71 | 0.02 | S549 | 5.02 | 90.62 | 37.74 | 97.48 | East Asia | China | G2 | 0.53 | 0.02 |
| S86 | 6.78 | 94.03 | 36.62 | 97.6 | South Africa | China | G3 | 0.68 | 0.02 | S550 | 5.06 | 90.29 | 37.73 | 97.46 | East Asia | China | G2 | 0.51 | 0.02 |
| S87 | 6.19 | 92.85 | 37.39 | 98.29 | East Africa | China | G4 | 0.34 | 0.02 | S551 | 5.33 | 90.44 | 37.85 | 96.63 | East Asia | China | G2 | - | 0.01 |
| S88 | 6.19 | 93.27 | 37.34 | 98.27 | East Africa | China | G4 | 0.34 | 0.02 | S552 | 5.91 | 90.67 | 37.97 | 94.31 | East Asia | China | G2 | - | 0.02 |
| S89 | 4.92 | 93.08 | 37.74 | 97.98 | East Africa | Sudan | G3 | 0.57 | 0.02 | S553 | 5.23 | 90.95 | 37.64 | 97.65 | East Asia | China | G2 | 0.63 | 0.02 |
| S90 | 5.96 | 92.84 | 37.45 | 96.72 | East Africa | Sudan | G3 | 0.45 | 0.02 | S554 | 4.92 | 90.43 | 37.59 | 97.16 | East Asia | China | G3 | - | 0.01 |
| S91 | 4.3 | 93.02 | 37.78 | 96.68 | East Africa | Sudan | G3 | - | 0.01 | S555 | 5.35 | 90.86 | 37.74 | 94.89 | East Asia | China | G3 | - | 0.02 |
| S92 | 5.33 | 93.62 | 37.51 | 97.32 | East Africa | Sudan | G2 | 0.37 | 0.02 | S556 | 5.62 | 90.58 | 37.11 | 97.73 | East Asia | China | G2 | 0.24 | 0.02 |
| S93 | 5.98 | 93.59 | 37.94 | 97 | East Africa | Sudan | G4 | 0.19 | 0.09 | S557 | 5.41 | 90.71 | 36.42 | 98.71 | East Asia | China | G2 | 0.2 | 0.01 |
| S94 | 6.21 | 93.14 | 37.95 | 97.4 | South-East Asia | Myanmar | G3 | 0.22 | 0.02 | S558 | 4.69 | 90.56 | 37.91 | 96.94 | East Asia | China | G3 | - | 0.02 |
| S95 | 7.63 | 93.46 | 37.41 | 98.2 | South-East Asia | Myanmar | G3 | 0.29 | 0.08 | S559 | 4.48 | 90.42 | 37.45 | 97.27 | East Asia | China | G3 | - | 0.01 |
| S96 | 6.3 | 93.19 | 37.26 | 98.35 | South-East Asia | Myanmar | G3 | 0.31 | 0.01 | S560 | 5 | 90.45 | 37.65 | 97.83 | East Asia | China | G3 | 0.49 | 0.02 |
| S97 | 6.17 | 93.56 | 37.45 | 96.76 | South-East Asia | Myanmar | G2 | 0.48 | 0.01 | S561 | 5.52 | 90.57 | 37.76 | 97.98 | East Asia | China | G3 | 0.32 | 0.02 |
| S98 | 6.97 | 93.81 | 37.25 | 97.35 | South-East Asia | Myanmar | G3 | 0.35 | 0.01 | S562 | 5.01 | 90.59 | 38.06 | 96.12 | East Asia | China | G3 | - | 0.02 |
| S99 | 4.03 | 93.01 | 37.58 | 97.41 | South-East Asia | Myanmar | G4 | - | 0.02 | S563 | 4.9 | 90.75 | 38.21 | 96.63 | East Asia | China | G3 | - | 0.02 |
| S100 | 4.35 | 93.69 | 37.31 | 98.17 | South-East Asia | Myanmar | G2 | 0.68 | 0.01 | S564 | 4.57 | 90.24 | 37.8 | 96.94 | East Asia | China | G2 | - | 0.02 |
| S101 | 5.21 | 93.53 | 36.92 | 98.59 | South-East Asia | Myanmar | G2 | 0.35 | 0.01 | S565 | 5.2 | 90.48 | 38.14 | 92.45 | East Asia | China | G2 | - | 0.01 |
| S102 | 5.37 | 93.3 | 37.58 | 96.93 | Central America | Mexico | G4 | 0.58 | 0.04 | S566 | 4.61 | 90.66 | 37.56 | 95.49 | East Asia | China | G2 | - | 0.01 |
| S103 | 5.39 | 93.79 | 37.31 | 98.68 | Central America | Mexico | G2 | 0.39 | 0.01 | S567 | 4.5 | 91.08 | 38.21 | 94.25 | East Asia | China | G2 | - | 0.02 |
| S104 | 5.04 | 93.53 | 37.99 | 97.8 | Central America | Mexico | G2 | 0.64 | 0.02 | S568 | 5.32 | 90.66 | 37.64 | 97.54 | East Asia | China | G2 | 0.45 | 0.01 |
| S105 | 6.91 | 94.15 | 37.32 | 97.35 | Central America | Mexico | G2 | 0.31 | 0.02 | S569 | 6.28 | 91.28 | 37.3 | 95.93 | East Asia | China | G1 | 0.36 | 0.01 |
| S106 | 5.76 | 93.7 | 38.25 | 93.13 | Central America | Mexico | G2 | 1.12 | 0.01 | S570 | 7.48 | 91.19 | 37.44 | 97 | East Asia | China | G3 | 0.25 | 0.01 |
| S107 | 7.03 | 93.55 | 37.19 | 92.06 | Central America | Mexico | G2 | 0.7 | 0.01 | S571 | 5.88 | 90.62 | 37.52 | 96.36 | East Asia | China | G2 | 0.51 | 0.03 |
| S108 | 5.36 | 93.22 | 37.45 | 97.48 | West Africa | Togo | G4 | 0.6 | 0.01 | S572 | 5.57 | 90.95 | 37.22 | 96.92 | East Asia | China | G3 | 0.42 | 0.02 |
| S109 | 4.93 | 93.46 | 37.41 | 98.12 | East Africa | United Republic of Tanzania | G4 | 0.58 | 0.01 | S573 | 5.96 | 91.03 | 37.43 | 97.01 | East Asia | China | G2 | 0.28 | 0.01 |
| S110 | 5.11 | 93.37 | 37.44 | 97.36 | East Africa | United Republic of Tanzania | G4 | 0.57 | 0.01 | S574 | 6.38 | 91.19 | 37.77 | 96.31 | East Asia | China | G2 | 0.47 | 0.01 |
| S111 | 7.29 | 93.15 | 37.03 | 98.14 | East Africa | United Republic of Tanzania | G4 | 0.18 | 0.01 | S575 | 5.48 | 91.62 | 37.7 | 94.48 | East Asia | China | G2 | 0.63 | 0.01 |
| S112 | 7.77 | 93.96 | 37.09 | 98.78 | East Africa | United Republic of Tanzania | G4 | 0.07 | 0.02 | S576 | 5.76 | 91.17 | 37.63 | 95.92 | East Asia | China | G2 | 0.35 | 0.01 |
| S113 | 5.47 | 93.78 | 39.8 | 92.07 | East Africa | United Republic of Tanzania | G4 | 0.64 | 0.02 | S577 | 5.76 | 91.44 | 37.53 | 96.51 | East Asia | China | G3 | 0.33 | 0.01 |
| S114 | 5.05 | 92.81 | 37.67 | 96.13 | East Africa | United Republic of Tanzania | G4 | - | 0.01 | S578 | 5.69 | 91.25 | 37.29 | 97.36 | East Asia | China | G2 | 0.44 | 0.01 |
| S115 | 6.82 | 92.64 | 37.4 | 97.37 | East Africa | United Republic of Tanzania | G2 | 0.45 | 0.07 | S579 | 7.34 | 90.56 | 37.25 | 98.6 | East Asia | China | G3 | 0.1 | 0.01 |
| S116 | 11.48 | 93.22 | 37.34 | 98.7 | East Africa | United Republic of Tanzania | G4 | 0.08 | 0.01 | S580 | 5.69 | 91.13 | 37.55 | 96.63 | East Asia | China | G3 | 0.35 | 0.01 |
| S117 | 6.44 | 92.59 | 37.93 | 97.41 | West Asia | China | G4 | 0.33 | 0.01 | S581 | 5.56 | 90.91 | 37.09 | 97.26 | East Asia | China | G2 | 0.28 | 0.01 |
| S118 | 3.51 | 93.83 | 38.63 | 96.89 | West Asia | China | G2 | - | 0.01 | S582 | 5.93 | 91.17 | 37.36 | 97.13 | East Asia | China | G2 | 0.34 | 0.01 |
| S119 | 6.16 | 93.16 | 37.45 | 97.22 | West Asia | China | G2 | 0.31 | 0.01 | S583 | 5.19 | 90.56 | 37.02 | 98.06 | East Asia | China | G2 | 0.36 | 0.01 |
| S120 | 7.92 | 92.93 | 37.99 | 96.85 | West Asia | China | G2 | 0.32 | 0.02 | S584 | 5.76 | 90.66 | 37.06 | 97.5 | East Asia | China | G2 | 0.3 | 0.01 |
| S121 | 7.77 | 92.71 | 37.22 | 97.55 | East Africa | Uganda | G4 | 0.18 | 0.02 | S585 | 6.12 | 91.66 | 37.59 | 96.62 | East Asia | China | G4 | 0.35 | 0.02 |
| S122 | 5.09 | 92.96 | 37.64 | 97.45 | West Africa | Mali | G3 | 0.59 | 0.02 | S586 | 6.09 | 91.08 | 37.38 | 94.71 | East Asia | China | G2 | 0.61 | 0.01 |
| S123 | 6.41 | 93.46 | 38.14 | 97.57 | East Asia | Democratic People's Republic of Korea | G1 | 0.31 | 0.02 | S587 | 4.71 | 91.31 | 37.2 | 97.69 | East Asia | China | G2 | 0.53 | 0.01 |
| S124 | 8.14 | 93.5 | 38.02 | 96.85 | East Asia | Republic of Korea | G3 | 0.3 | 0.02 | S588 | 3.33 | 92.82 | 37.28 | 96.73 | East Asia | China | G2 | - | 0.02 |
| S125 | 7.04 | 93.27 | 37.46 | 97.75 | East Asia | China | G3 | 0.24 | 0.01 | S589 | 6.07 | 91.18 | 37.29 | 98.16 | East Asia | China | G3 | 0.23 | 0.01 |
| S126 | 8.23 | 93.39 | 37.78 | 97.73 | East Asia | Japan | G2 | 0.24 | 0.04 | S590 | 3.62 | 92.82 | 36.8 | 98.08 | East Asia | China | G2 | - | 0.02 |
| S127 | 5.54 | 93.36 | 37.56 | 93.91 | East Africa | Mozambique | G2 | 0.93 | 0.02 | S591 | 3.49 | 93.47 | 37.45 | 96.97 | East Asia | China | G3 | - | 0.02 |
| S128 | 8.13 | 93.5 | 37.99 | 96.93 | East Africa | Mozambique | G4 | 0.31 | 0.01 | S592 | 3.86 | 92.62 | 37.11 | 98.47 | East Asia | China | G3 | 0.33 | 0.01 |
| S129 | 4.91 | 93.34 | 37.51 | 98.05 | Centra Africa | Guinea | G2 | 0.69 | 0.01 | S593 | 6.67 | 90.55 | 37.29 | 97.65 | East Asia | China | G2 | 0.17 | 0.01 |
| S130 | 6.15 | 93.34 | 37.27 | 97.93 | Southern Europe | Greece | G2 | 0.4 | 0.02 | S594 | 4.52 | 90.47 | 36.68 | 97.83 | East Asia | China | G3 | 0.29 | 0.01 |
| S131 | 6.74 | 93.03 | 37.66 | 98.09 | Southern Europe | Greece | G2 | 0.29 | 0.02 | S595 | 4.91 | 91.37 | 37.39 | 96.64 | East Asia | China | G2 | 0.49 | 0.01 |
| S132 | 5.99 | 93.31 | 37.49 | 97.95 | Southern Europe | Greece | G2 | 0.29 | 0.02 | S596 | 5.59 | 92.32 | 37.57 | 97.21 | East Asia | China | G2 | 0.6 | 0.02 |
| S133 | 6.01 | 92.82 | 37.64 | 96.9 | Southern Europe | Greece | G2 | 0.37 | 0.02 | S597 | 3.57 | 91.4 | 37.73 | 94.6 | East Asia | China | G2 | - | 0.01 |
| S134 | 7.63 | 93.15 | 36.86 | 98.6 | South-East Asia | Thailand | G2 | 0.19 | 0.01 | S598 | 4.49 | 91.45 | 37.29 | 97.2 | East Asia | China | G2 | 0.53 | 0.01 |
| S135 | 11.79 | 93.28 | 36.98 | 98.41 | South-East Asia | Thailand | G2 | 0.06 | 0.02 | S599 | 3.67 | 91.46 | 37.76 | 97.42 | East Asia | China | G2 | - | 0.01 |
| S136 | 5.63 | 93.72 | 37.62 | 97.61 | South-East Asia | Thailand | G2 | 0.73 | 0.02 | S600 | 8.63 | 91.51 | 37.08 | 97.95 | East Asia | China | G3 | 0.11 | 0.01 |
| S137 | 11.2 | 93.34 | 37.03 | 97.48 | South-East Asia | Thailand | G3 | 0.09 | 0.02 | S601 | 5.43 | 91.09 | 37.02 | 97.8 | East Asia | China | G3 | 0.36 | 0.02 |
| S138 | 7.07 | 93.97 | 37.19 | 97.63 | Caribbean | Cuba | G3 | 0.41 | 0.02 | S602 | 7.78 | 91.41 | 36.91 | 98.52 | East Asia | China | G2 | 0.14 | 0.02 |
| S139 | 3.95 | 94.05 | 37.35 | 97.79 | West Africa | China | G4 | 0.64 | 0.02 | S603 | 7.19 | 91.29 | 36.91 | 98.05 | East Asia | China | G3 | 0.13 | 0.02 |
| S140 | 6.58 | 92.89 | 37.77 | 96.87 | East Africa | Somalia | G4 | 0.55 | 0.02 | S604 | 5.93 | 91.32 | 37.07 | 97.99 | East Asia | China | G2 | 0.25 | 0.02 |
| S141 | 11.2 | 92.81 | 36.89 | 84.02 | West Africa | Nigeria | G4 | 0.13 | 0 | S605 | 5.82 | 91.09 | 37.75 | 97.33 | East Asia | China | G3 | 0.52 | 0.02 |
| S142 | 4.31 | 93.65 | 38.41 | 93.32 | West Africa | Nigeria | G4 | - | 0.02 | S606 | 6.76 | 91.43 | 37.55 | 95.55 | East Asia | China | G2 | 0.5 | 0.02 |
| S143 | 4.46 | 94.52 | 37.44 | 97.98 | West Asia | Turkey | G2 | 0.65 | 0.02 | S607 | 7.42 | 91.19 | 36.86 | 98.44 | East Asia | China | G2 | 0.13 | 0.01 |
| S144 | 4.86 | 93.31 | 38.05 | 97.06 | South Asia | Bangladesh | G4 | 0.73 | 0.03 | S608 | 6.82 | 90.45 | 38.6 | 93.52 | East Asia | China | G1 | 0.78 | 0.03 |
| S145 | 6.04 | 94.02 | 37.26 | 98.27 | West Africa | Senegal | G4 | 0.22 | 0.01 | S609 | 7.11 | 90.25 | 38.33 | 97.2 | East Asia | China | G1 | 0.24 | 0.02 |
| S146 | 7.96 | 93.38 | 37.68 | 97.59 | West Africa | Togo | G2 | 0.29 | 0.02 | S610 | 6.35 | 91.53 | 37.54 | 98.16 | East Asia | China | G1 | 0.24 | 0.02 |
| S147 | 6.9 | 92.89 | 37.77 | 94.94 | East Africa | Uganda | G1 | 0.71 | 0.01 | S611 | 5.09 | 91.09 | 38 | 96.39 | East Asia | China | G1 | 0.51 | 0.01 |
| S148 | 6.3 | 93.53 | 37.81 | 97.31 | West Asia | China | G3 | 0.53 | 0.02 | S612 | 5.92 | 90.31 | 37.61 | 97.14 | East Asia | China | G1 | 0.37 | 0.02 |
| S149 | 4.74 | 93.22 | 37.86 | 94.57 | South Asia | India | G2 | - | 0.02 | S613 | 6.07 | 89.5 | 37.34 | 97.33 | East Asia | China | G1 | 0.22 | 0.01 |
| S150 | 9.27 | 93.15 | 37.93 | 97.62 | Central America | Mexico | G3 | 0.17 | 0.01 | S614 | 5.89 | 89.63 | 37.79 | 92.33 | East Asia | China | G1 | - | 0.02 |
| S151 | 8.37 | 93.36 | 37.72 | 96.76 | Central America | Mexico | G2 | 0.46 | 0.1 | S615 | 5.15 | 89.66 | 37.58 | 96.05 | West Africa | Burkina Faso | G3 | - | 0.01 |
| S152 | 6.23 | 93.49 | 37.7 | 91.26 | East Asia | China | G2 | 0.94 | 0.02 | S616 | 5.2 | 91.06 | 37.64 | 97.19 | East Asia | China | G1 | 0.62 | 0.02 |
| S153 | 5.33 | 93.59 | 37.83 | 98.17 | East Asia | China | G2 | 0.57 | 0.01 | S617 | 5.97 | 90.95 | 37.75 | 97.58 | East Asia | China | G2 | 0.34 | 0.02 |
| S154 | 4.57 | 93.38 | 37.23 | 97.53 | East Asia | China | G2 | 0.7 | 0.02 | S618 | 4.97 | 92.52 | 37.66 | 98.58 | East Asia | China | G2 | 0.29 | 0.03 |
| S155 | 5.64 | 93.58 | 37.58 | 97.64 | East Asia | China | G2 | 0.46 | 0.02 | S619 | 3.65 | 90.81 | 37.31 | 97.9 | East Asia | China | G2 | - | 0.01 |
| S156 | 6.14 | 93.83 | 37.67 | 96.71 | East Asia | China | G2 | 0.42 | 0.02 | S620 | 5.71 | 91.58 | 36.86 | 98.8 | East Asia | China | G2 | 0.11 | 0.01 |
| S157 | 8.14 | 93.2 | 37.38 | 97.47 | East Asia | China | G2 | 0.25 | 0.02 | S621 | 5.26 | 92.9 | 36.86 | 98.9 | East Asia | China | G2 | 0.09 | 0.02 |
| S158 | 8.56 | 93.93 | 37.59 | 97.54 | East Asia | China | G2 | 0.27 | 0.02 | S622 | 5.64 | 92.8 | 37.12 | 98.92 | East Asia | China | G2 | 0.09 | 0.01 |
| S159 | 8.28 | 93.25 | 37.47 | 97.83 | East Asia | China | G2 | 0.22 | 0.01 | S623 | 3.53 | 90.28 | 37.45 | 98.53 | East Asia | China | G2 | 0.36 | 0.02 |
| S160 | 6.94 | 94.33 | 37.2 | 98.27 | East Asia | China | G2 | 0.21 | 0.02 | S624 | 4.32 | 91.81 | 36.38 | 98.83 | East Asia | China | G2 | 0.12 | 0.01 |
| S161 | 7.3 | 94.1 | 37.09 | 98.31 | East Asia | China | G2 | 0.2 | 0.02 | S625 | 4.59 | 90.86 | 36.62 | 98.64 | East Asia | China | G2 | 0.16 | 0.01 |
| S162 | 7.01 | 94.49 | 37.57 | 97.24 | East Asia | China | G2 | 0.42 | 0.01 | S626 | 3.97 | 91.01 | 37.1 | 98.89 | East Asia | China | G2 | 0.26 | 0.01 |
| S163 | 5.34 | 94.14 | 37.84 | 96.42 | East Asia | China | G2 | 0.71 | 0.02 | S627 | 5.79 | 91.08 | 37.25 | 98.83 | East Asia | China | G2 | 0.09 | 0.01 |
| S164 | 5.85 | 94.01 | 37.32 | 97.85 | East Asia | China | G2 | 0.45 | 0.02 | S628 | 4.6 | 91.73 | 36.88 | 98.28 | East Asia | China | G2 | 0.17 | 0.01 |
| S165 | 5.53 | 94.25 | 37.03 | 98.35 | East Asia | China | G2 | 0.25 | 0.01 | S629 | 4.97 | 91.41 | 36.57 | 98.92 | East Asia | China | G2 | 0.12 | 0.02 |
| S166 | 5.23 | 93.94 | 37.33 | 98.5 | East Asia | China | G2 | 0.48 | 0.02 | S630 | 5.68 | 90.92 | 36.88 | 98.95 | East Asia | China | G2 | 0.12 | 0.02 |
| S167 | 7.22 | 94.12 | 37.64 | 97.65 | East Asia | China | G2 | 0.28 | 0.02 | S631 | 4.31 | 91.19 | 37.33 | 98.34 | East Asia | China | G2 | 0.2 | 0.01 |
| S168 | 5.05 | 94.02 | 37.67 | 96.58 | East Asia | China | G2 | 0.8 | 0.02 | S632 | 5.37 | 92.64 | 36.64 | 98.78 | East Asia | China | G2 | 0.1 | 0.01 |
| S169 | 4.88 | 94.26 | 37.36 | 98.16 | East Asia | China | G2 | 0.37 | 0.03 | S633 | 4.65 | 91.66 | 36.91 | 98.7 | East Asia | China | G2 | 0.13 | 0.01 |
| S170 | 5.73 | 94.41 | 37.41 | 97.65 | East Asia | China | G2 | 0.59 | 0.04 | S634 | 4.36 | 91.38 | 37.61 | 98.69 | East Asia | China | G2 | 0.49 | 0.01 |
| S171 | 5.28 | 94.59 | 37.32 | 96.8 | East Asia | China | G3 | 0.62 | 0.02 | S635 | 3.01 | 91.05 | 37.03 | 98.48 | East Asia | China | G1 | - | 0.01 |
| S172 | 6.17 | 94.31 | 37.37 | 97.63 | East Asia | China | G2 | 0.37 | 0.02 | S636 | 4.53 | 91.65 | 37.07 | 98.52 | East Asia | China | G1 | 0.29 | 0.02 |
| S173 | 7.25 | 94 | 36.98 | 98.4 | East Asia | China | G3 | 0.17 | 0.02 | S637 | 4.96 | 92.1 | 36.67 | 98.57 | East Asia | China | G2 | 0.17 | 0.02 |
| S174 | 5.06 | 94.02 | 37.22 | 97.83 | East Asia | China | G2 | 0.52 | 0.02 | S638 | 4.88 | 90.69 | 36.84 | 98.49 | East Asia | China | G1 | 0.13 | 0.01 |
| S175 | 7.79 | 93.78 | 37.67 | 96.51 | East Asia | China | G2 | 0.31 | 0.03 | S639 | 4.77 | 90.6 | 37.31 | 98.6 | East Asia | China | G2 | 0.19 | 0.01 |
| S176 | 10.62 | 93.96 | 37.7 | 94.27 | East Asia | China | G3 | 0.21 | 0.02 | S640 | 6.28 | 90.78 | 40.29 | 98.86 | East Asia | China | G2 | 0.92 | 0.01 |
| S177 | 10.16 | 94.17 | 38.06 | 96.2 | East Asia | China | G2 | 0.16 | 0.02 | S641 | 4.6 | 89.84 | 36.88 | 98.64 | East Asia | China | G2 | 0.17 | 0.01 |
| S178 | 5.93 | 93.41 | 37.46 | 97.74 | East Asia | China | G2 | 0.2 | 0.02 | S642 | 3.28 | 90.3 | 38.19 | 99.12 | East Asia | China | G2 | 0.62 | 0.01 |
| S179 | 8.84 | 94.01 | 37.89 | 97.81 | East Asia | China | G2 | 0.15 | 0.02 | S643 | 5.36 | 91.65 | 37.12 | 98.04 | East Asia | China | G1 | 0.17 | 0.02 |
| S180 | 4.89 | 94.18 | 37.38 | 97.87 | East Asia | China | G2 | 0.46 | 0.02 | S644 | 4.34 | 92.19 | 36.91 | 98.03 | East Africa | China | G4 | 0.25 | 0.02 |
| S181 | 6.53 | 93.7 | 37.88 | 98.71 | East Asia | China | G2 | 0.16 | 0.02 | S645 | 4.78 | 90.92 | 36.81 | 98.61 | South-East Asia | Viet Nam | G2 | 0.12 | 0.01 |
| S182 | 6.5 | 93.87 | 37.41 | 98.36 | East Asia | China | G2 | 0.16 | 0.02 | S646 | 4.67 | 91.29 | 37.17 | 98.07 | North America | United States of America | G2 | 0.19 | 0.01 |
| S183 | 6.17 | 94.55 | 37.64 | 96.45 | East Asia | China | G2 | 0.37 | 0.02 | S647 | 6.19 | 91.03 | 36.94 | 98.91 | North America | United States of America | G2 | 0.09 | 0.01 |
| S184 | 4.72 | 94.48 | 37.3 | 97.78 | East Asia | China | G2 | 0.65 | 0.02 | S648 | 4.3 | 90.2 | 36.9 | 97.77 | North America | United States of America | G2 | 0.34 | 0.01 |
| S185 | 8.29 | 93.57 | 37.22 | 98 | East Asia | China | G2 | 0.14 | 0.02 | S649 | 4.7 | 90.89 | 37.11 | 98.73 | North America | United States of America | G4 | 0.21 | 0.01 |
| S186 | 10.17 | 94.22 | 37.27 | 98.28 | East Asia | China | G2 | 0.09 | 0.02 | S650 | 3.2 | 91.27 | 36.59 | 98.41 | North America | United States of America | G2 | - | 0.01 |
| S187 | 6.84 | 93.96 | 37.66 | 98.24 | East Asia | China | G2 | 0.19 | 0.02 | S651 | 4.98 | 90.75 | 36.81 | 98.43 | North America | United States of America | G2 | 0.11 | 0.02 |
| S188 | 4.82 | 94.49 | 37.28 | 93.92 | East Asia | China | G2 | - | 0.02 | S652 | 5.31 | 92.44 | 36.62 | 99.04 | North America | United States of America | G2 | 0.09 | 0.02 |
| S189 | 8.18 | 94.18 | 37.03 | 98.08 | East Asia | China | G2 | 0.13 | 0.02 | S653 | 3.91 | 92.69 | 37.6 | 98.88 | North America | United States of America | G2 | 1.13 | 0.01 |
| S190 | 5.1 | 94.05 | 37.33 | 97.87 | East Asia | China | G2 | 0.5 | 0.02 | S654 | 6.63 | 91.81 | 36.79 | 99.15 | North America | United States of America | G2 | 0.08 | 0.01 |
| S191 | 5.89 | 93.59 | 37.22 | 97.74 | East Asia | China | G2 | 0.26 | 0.02 | S655 | 3.86 | 91.33 | 36.83 | 98.73 | North America | United States of America | G2 | 0.28 | 0.01 |
| S192 | 7.66 | 93.74 | 37.41 | 97.27 | East Asia | China | G2 | 0.19 | 0.02 | S656 | 4.1 | 91.66 | 36.66 | 98.99 | North America | United States of America | G2 | 0.21 | 0.02 |
| S193 | 5.81 | 93.12 | 37.75 | 97.27 | East Asia | China | G2 | 0.3 | 0.01 | S657 | 3.54 | 92.41 | 36.17 | 98.83 | East Asia | Japan | G2 | - | 0.01 |
| S194 | 7.06 | 93.42 | 37.86 | 96.9 | East Asia | China | G2 | 0.39 | 0.01 | S658 | 5.62 | 92.79 | 36.75 | 98.5 | East Africa | Mozambique | G4 | 0.05 | 0.02 |
| S195 | 7.41 | 92.74 | 37.45 | 97.6 | East Asia | China | G2 | 0.18 | 0.02 | S659 | 5.88 | 93.13 | 36.92 | 97.48 | Southern Europe | Greece | G2 | 0.25 | 0.02 |
| S196 | 8.39 | 93.3 | 37.87 | 97.12 | East Asia | China | G2 | 0.26 | 0.01 | S660 | 3.44 | 92.05 | 36.09 | 99.12 | Caribbean | Cuba | G3 | 0.3 | 0.02 |
| S197 | 5.79 | 93.54 | 37.89 | 97.65 | East Asia | China | G2 | 0.35 | 0.01 | S661 | 5.59 | 92.12 | 36.81 | 96.96 | West Asia | Turkey | G2 | 0.56 | 0.01 |
| S198 | 6.45 | 93.41 | 37.33 | 98.08 | East Asia | China | G2 | 0.17 | 0.01 | S662 | 3.19 | 91.49 | 37.32 | 98.51 | East Asia | China | G2 | - | 0.02 |
| S199 | 5.55 | 93.81 | 37.71 | 97.52 | East Asia | China | G2 | 0.44 | 0.01 | S663 | 4.17 | 91.49 | 36.66 | 98.79 | East Asia | China | G2 | 0.25 | 0.01 |
| S200 | 5.93 | 93.63 | 37.52 | 94 | East Asia | China | G2 | 0.74 | 0.01 | S664 | 3.14 | 91.35 | 37.27 | 98.44 | East Asia | China | G2 | - | 0.02 |
| S201 | 5.43 | 93.57 | 37.76 | 97.17 | East Asia | China | G2 | 0.5 | 0.01 | S665 | 7.15 | 92.54 | 36.97 | 97.88 | East Asia | China | G2 | 0.16 | 0.02 |
| S202 | 7.62 | 93.52 | 37.73 | 97.07 | East Asia | China | G2 | 0.28 | 0.01 | S666 | 4.64 | 91.55 | 36.4 | 98.64 | East Asia | China | G2 | 0.11 | 0.02 |
| S203 | 7.43 | 93.19 | 37.78 | 97.18 | East Asia | China | G2 | 0.25 | 0.01 | S667 | 3.68 | 91.17 | 36.76 | 98.78 | East Asia | China | G2 | 0.27 | 0.02 |
| S204 | 8.08 | 93.58 | 38.19 | 97.53 | East Asia | China | G2 | 0.27 | 0.01 | S668 | 2.82 | 90.72 | 36.86 | 98.87 | East Asia | China | G2 | - | 0.02 |
| S205 | 5.35 | 93.69 | 37.68 | 97.7 | East Asia | China | G2 | 0.66 | 0.01 | S669 | 5.45 | 92.02 | 36.8 | 98.67 | East Asia | China | G2 | 0.12 | 0.02 |
| S206 | 6.68 | 93.96 | 37.34 | 97.29 | East Asia | China | G2 | 0.38 | 0.01 | S670 | 3.5 | 90.34 | 37.36 | 98.5 | East Asia | China | G1 | 0.32 | 0.02 |
| S207 | 5.89 | 93.28 | 37.11 | 98.35 | East Asia | China | G2 | 0.29 | 0.01 | S671 | 3.41 | 92.16 | 37.01 | 97.94 | East Asia | China | G1 | - | 0.02 |
| S208 | 5.18 | 93.21 | 37.84 | 97.95 | East Asia | China | G2 | 0.54 | 0.01 | S672 | 4.46 | 90.77 | 37.03 | 98.09 | East Asia | China | G1 | 0.27 | 0.02 |
| S209 | 5.2 | 93.97 | 37.78 | 96.05 | East Asia | China | G2 | 0.66 | 0.02 | S673 | 4.4 | 91.3 | 37.93 | 89.13 | East Asia | China | G1 | - | 0.03 |
| S210 | 5.35 | 93.22 | 37.89 | 97.83 | East Asia | China | G2 | 0.44 | 0.02 | S674 | 4.51 | 91.44 | 38.45 | 99.12 | East Asia | China | G2 | 0.6 | 0.01 |
| S211 | 5.9 | 93.72 | 37.55 | 98.24 | East Asia | China | G2 | 0.27 | 0.01 | S675 | 3.87 | 90.74 | 36.8 | 98.56 | East Asia | China | G3 | 0.28 | 0.01 |
| S212 | 7.2 | 93.73 | 37.76 | 97.9 | East Asia | China | G2 | 0.35 | 0.01 | S676 | 3.6 | 91.53 | 36.34 | 98.85 | East Asia | China | G3 | 0.27 | 0.02 |
| S213 | 4.74 | 93.17 | 37.92 | 97.01 | East Asia | China | G2 | 0.69 | 0.01 | S677 | 2.52 | 90.18 | 37.7 | 98.77 | East Asia | China | G2 | - | 0.01 |
| S214 | 5.28 | 93.75 | 37.73 | 97.71 | East Asia | China | G2 | 0.58 | 0.01 | S678 | 4.89 | 92.65 | 37.17 | 95.56 | East Asia | China | G1 | 0.4 | 0.02 |
| S215 | 6.26 | 93.85 | 37.92 | 95.92 | East Asia | China | G2 | 0.69 | 0.01 | S679 | 5.22 | 92.03 | 37.16 | 98.38 | East Asia | China | G3 | 0.13 | 0.02 |
| S216 | 5.42 | 93.5 | 37.31 | 98.34 | East Asia | China | G2 | 0.39 | 0.02 | S680 | 4.81 | 92.53 | 37.88 | 98.07 | East Asia | China | G2 | 0.44 | 0.02 |
| S217 | 8.05 | 94.28 | 38.01 | 97.86 | East Asia | China | G2 | 0.29 | 0.01 | S681 | 6.1 | 91.8 | 36.9 | 98.83 | East Asia | China | G2 | 0.08 | 0.02 |
| S218 | 6.72 | 93.62 | 38.2 | 97.31 | East Asia | China | G2 | 0.41 | 0.01 | S682 | 4.53 | 92.72 | 37.3 | 98.34 | East Asia | China | G1 | 0.33 | 0.02 |
| S219 | 11.97 | 93.88 | 37.58 | 95.93 | East Asia | China | G2 | 0.18 | 0.02 | S683 | 5.74 | 93.29 | 37.64 | 98.64 | East Asia | China | G1 | 0.16 | 0.03 |
| S220 | 6.7 | 93.6 | 37.59 | 96.78 | East Asia | China | G2 | 0.36 | 0.01 | S684 | 7.26 | 92.41 | 37.28 | 98.72 | Eastern Europe | Bulgaria | G3 | 0.09 | 0.02 |
| S221 | 5.61 | 93.97 | 37.88 | 96.97 | East Asia | China | G2 | 0.69 | 0.02 | S685 | 7.88 | 92.87 | 36.98 | 98.82 | East Asia | China | G2 | 0.06 | 0.02 |
| S222 | 6.59 | 93.53 | 37.7 | 96.89 | East Asia | China | G2 | 0.41 | 0.01 | S686 | 5.15 | 92.97 | 36.98 | 98.36 | East Asia | China | G2 | 0.1 | 0.02 |
| S223 | 7.66 | 94.04 | 37.68 | 96.56 | East Asia | China | G2 | 0.4 | 0.01 | S687 | 5.87 | 91.45 | 39.33 | 99.12 | East Asia | China | G2 | 0.64 | 0.02 |
| S224 | 6.09 | 94.07 | 37.53 | 98.22 | East Asia | China | G2 | 0.38 | 0.02 | S688 | 6.15 | 93.09 | 37.73 | 98.6 | East Asia | China | G2 | 0.19 | 0.02 |
| S225 | 5.49 | 93.31 | 38.25 | 97.3 | East Asia | China | G2 | 0.65 | 0.02 | S689 | 5.61 | 92.66 | 37.35 | 98.68 | East Asia | China | G2 | 0.15 | 0.02 |
| S226 | 5.56 | 93.23 | 38.3 | 97.77 | East Asia | China | G2 | 0.58 | 0.02 | S690 | 4.05 | 93.15 | 37.52 | 97.38 | East Asia | China | G2 | - | 0.03 |
| S227 | 6.79 | 94.17 | 37.87 | 97.67 | East Asia | China | G2 | 0.4 | 0.02 | S691 | 5.59 | 93.63 | 37.45 | 96.87 | East Asia | China | G1 | 0.22 | 0.02 |
| S228 | 8.05 | 93.66 | 37.51 | 98.05 | East Asia | China | G2 | 0.2 | 0.02 | S692 | 5.14 | 93.26 | 38.1 | 97.75 | East Asia | China | G2 | 0.29 | 0.05 |
| S229 | 6.72 | 93.73 | 38.09 | 97.15 | East Asia | China | G2 | 0.46 | 0.01 | S693 | 5.84 | 93.06 | 36.97 | 98.83 | East Asia | China | G2 | 0.14 | 0.02 |
| S230 | 5.56 | 93.59 | 38.23 | 97.32 | East Asia | China | G2 | 0.64 | 0.01 | S694 | 4.86 | 93.67 | 36.9 | 97.95 | East Asia | China | G2 | 0.24 | 0.03 |
| S231 | 6.26 | 93.74 | 38.42 | 97.89 | East Asia | China | G2 | 0.37 | 0.02 | S695 | 4.91 | 93.78 | 37.09 | 97.89 | East Asia | China | G3 | 0.3 | 0.02 |
| S232 | 5.47 | 93.4 | 38.23 | 96.8 | East Asia | China | G2 | 0.57 | 0.01 | S696 | 7.07 | 93.48 | 36.89 | 98.29 | East Asia | China | G3 | 0.09 | 0.02 |
| S233 | 5.09 | 93.64 | 37.98 | 96.65 | East Asia | China | G2 | 0.68 | 0.01 | S697 | 4.82 | 93.05 | 36.61 | 98.78 | South Asia | China | G4 | 0.1 | 0.02 |
| S234 | 4.46 | 93.5 | 37.86 | 96.42 | East Asia | China | G2 | - | 0.02 | S698 | 2.98 | 93.5 | 37.97 | 98.69 | South Asia | China | G4 | - | 0.02 |
| S235 | 6.21 | 92.85 | 37.46 | 97.12 | East Asia | China | G2 | 0.39 | 0.02 | S699 | 4.31 | 92.54 | 36.86 | 98.48 | East Africa | Sudan | G4 | 0.19 | 0.02 |
| S236 | 8.57 | 93.87 | 37.97 | 97.8 | East Asia | China | G2 | 0.26 | 0.01 | S700 | 5.2 | 93.06 | 37.27 | 98.53 | East Africa | United Republic of Tanzania | G4 | 0.16 | 0.02 |
| S237 | 5.41 | 93.78 | 37.79 | 97.8 | East Asia | China | G2 | 0.57 | 0.02 | S701 | 5.73 | 93.5 | 37.23 | 97.93 | South America | Venezuela | G3 | 0.23 | 0.02 |
| S238 | 5.47 | 93.25 | 38.36 | 93.54 | East Asia | China | G2 | - | 0.02 | S702 | 5.48 | 93.16 | 36.93 | 98.8 | East Africa | Mozambique | G4 | 0.09 | 0.02 |
| S239 | 7.4 | 93.69 | 38.17 | 96.98 | East Asia | China | G3 | 0.46 | 0.01 | S703 | 3.89 | 92.77 | 37.43 | 98.68 | South America | Paraguay | G3 | 0.35 | 0.02 |
| S240 | 4.77 | 93.15 | 37.78 | 97.52 | East Asia | China | G2 | 0.66 | 0.01 | S704 | 4.94 | 93.8 | 36.67 | 98.28 | West Africa | Nigeria | G4 | 0.14 | 0.01 |
| S241 | 4.86 | 94.16 | 38.12 | 97.89 | East Asia | China | G2 | 0.73 | 0.02 | S705 | 5.02 | 92.47 | 37.38 | 98.51 | South-East Asia | Indonesia | G4 | 0.27 | 0.02 |
| S242 | 6.35 | 93.77 | 37.55 | 97.75 | East Asia | China | G2 | 0.43 | 0.02 | S706 | 5.7 | 92.95 | 37.73 | 98.88 | South-East Asia | Indonesia | G4 | 0.14 | 0.02 |
| S243 | 6.98 | 94.04 | 37.66 | 97.48 | East Asia | China | G2 | 0.38 | 0.02 | S707 | 5.47 | 92.41 | 36.84 | 98.46 | Centra Africa | Chad | G4 | 0.14 | 0.02 |
| S244 | 8.86 | 93.66 | 38.52 | 97.91 | East Asia | China | G2 | 0.29 | 0.02 | S708 | 5.76 | 93.19 | 37.02 | 98.43 | South America | Brazil | G3 | 0.31 | 0.08 |
| S245 | 6.91 | 93.41 | 37.78 | 97.81 | East Asia | China | G2 | 0.3 | 0.02 | S709 | 4.48 | 92.03 | 36.02 | 98.39 | West Africa | Niger | G4 | 0.4 | 0.01 |
| S246 | 6.43 | 93.72 | 38.06 | 97.51 | East Asia | China | G1 | 0.4 | 0.02 | S710 | 5.07 | 92.1 | 36.85 | 98.67 | East Africa | China | G4 | 0.39 | 0.01 |
| S247 | 7.81 | 93.3 | 38.31 | 96.88 | East Asia | China | G2 | 0.35 | 0.01 | S711 | 7.18 | 92.63 | 36.98 | 99.35 | East Africa | China | G4 | 0.06 | 0.02 |
| S248 | 6.48 | 93.64 | 38.36 | 93.78 | East Asia | China | G3 | 1.07 | 0.02 | S712 | 6.44 | 93.22 | 37.26 | 99.13 | East Africa | China | G4 | 0.06 | 0.02 |
| S249 | 4.87 | 93.7 | 37.59 | 97 | East Asia | China | G2 | - | 0.01 | S713 | 6.2 | 94.46 | 36.87 | 99.34 | East Africa | Sudan | G4 | 0.06 | 0.02 |
| S250 | 6.65 | 93.74 | 38.23 | 97.55 | East Asia | China | G2 | 0.5 | 0.04 | S714 | 7.65 | 93.47 | 37.57 | 99.47 | East Africa | Sudan | G4 | 0.05 | 0.02 |
| S251 | 7.61 | 93.82 | 37.86 | 97.86 | East Asia | China | G2 | 0.34 | 0.01 | S715 | 6.23 | 93.31 | 37.05 | 99.33 | East Africa | United Republic of Tanzania | G4 | 0.05 | 0.02 |
| S252 | 4.43 | 93.67 | 38.67 | 95.63 | East Asia | China | G2 | - | 0.01 | S716 | 4.48 | 92.5 | 36.8 | 98.91 | East Africa | United Republic of Tanzania | G4 | 0.08 | 0.02 |
| S253 | 7.96 | 93.22 | 37.71 | 98.46 | East Asia | China | G2 | 0.18 | 0.02 | S717 | 4.99 | 92.93 | 36.94 | 99.01 | East Africa | United Republic of Tanzania | G4 | 0.11 | 0.02 |
| S254 | 5.9 | 93.6 | 37.4 | 98.01 | East Asia | China | G2 | 0.34 | 0.02 | S718 | 5.58 | 93.87 | 37.4 | 98.66 | East Africa | Somalia | G4 | 0.11 | 0.02 |
| S255 | 6.91 | 93.13 | 37.52 | 98.42 | East Asia | China | G2 | 0.26 | 0.03 | S719 | 5.93 | 92.91 | 37.75 | 98.89 | West Africa | Nigeria | G4 | 0.05 | 0.02 |
| S256 | 5.63 | 93.82 | 36.91 | 98.65 | East Asia | China | G2 | 0.3 | 0.02 | S720 | 5.08 | 94.43 | 36.74 | 98.75 | West Asia | Turkey | G2 | 0.12 | 0.03 |
| S257 | 8.95 | 93.72 | 38.01 | 97.79 | East Asia | China | G2 | 0.25 | 0.02 | S721 | 7.91 | 93.89 | 37.34 | 99.21 | West Africa | Burkina Faso | G4 | 0.03 | 0.02 |
| S258 | 7.76 | 93.75 | 37.66 | 98.16 | East Asia | China | G2 | 0.24 | 0.02 | S722 | 4.99 | 93.61 | 37.09 | 98.39 | East Asia | China | G1 | 0.12 | 0.02 |
| S259 | 8.36 | 93.41 | 37.15 | 98.07 | East Asia | China | G3 | 0.22 | 0.02 | S723 | 4.26 | 93.25 | 37.45 | 98.56 | East Asia | China | G1 | 0.16 | 0.02 |
| S260 | 8.12 | 93.34 | 37.63 | 96.68 | East Asia | China | G3 | 0.33 | 0.02 | S724 | 3.95 | 93.19 | 37.05 | 98.8 | East Asia | China | G2 | 0.27 | 0.02 |
| S261 | 7.33 | 93.24 | 37.25 | 98.04 | East Asia | China | G2 | 0.23 | 0.02 | S725 | 3.74 | 93.33 | 37.64 | 98.83 | South Asia | India | G2 | 0.15 | 0.02 |
| S262 | 5.11 | 93.94 | 37.72 | 97.01 | East Asia | China | G2 | 0.58 | 0.01 | S726 | 6.39 | 93.77 | 37.3 | 99.05 | South Asia | India | G2 | 0.06 | 0.02 |
| S263 | 7.38 | 93.53 | 37.63 | 97.18 | East Asia | China | G2 | 0.32 | 0.02 | S727 | 6.17 | 93.44 | 37.38 | 98.99 | South-East Asia | Myanmar | G3 | 0.07 | 0.03 |
| S264 | 5.86 | 93.19 | 38 | 96.73 | East Asia | China | G2 | 0.56 | 0.02 | S728 | 7.37 | 93.27 | 37.45 | 98.76 | South-East Asia | Myanmar | G4 | 0.08 | 0.03 |
| S265 | 6.34 | 93.57 | 37.36 | 96.55 | East Asia | China | G2 | 0.42 | 0.02 | S729 | 6.12 | 93.48 | 37.2 | 98.63 | North America | United States of America | G2 | 0.09 | 0.02 |
| S266 | 5.47 | 93.34 | 37.7 | 98.08 | East Asia | China | G2 | 0.52 | 0.02 | S730 | 4.92 | 93.66 | 37.32 | 98.99 | North America | United States of America | G2 | 0.1 | 0.02 |
| S267 | 7.95 | 93.78 | 38.07 | 97.39 | East Asia | China | G2 | 0.33 | 0.02 | S731 | 4.94 | 93.33 | 37.5 | 98.63 | East Asia | Japan | G2 | 0.12 | 0.02 |
| S268 | 7.09 | 93.77 | 37.96 | 96.16 | East Asia | China | G2 | 0.53 | 0.01 | S732 | 5.18 | 93.73 | 36.86 | 99.13 | East Asia | Japan | G2 | 0.07 | 0.02 |
| S269 | 5.04 | 93.37 | 37.8 | 97.88 | East Asia | China | G2 | 0.54 | 0.02 | S733 | 4.26 | 92.64 | 36.94 | 98.3 | East Asia | Japan | G2 | 0.28 | 0.02 |
| S270 | 5.45 | 93.53 | 38.32 | 96.87 | East Asia | China | G2 | - | 0.01 | S734 | 3.75 | 93.18 | 38.08 | 98.2 | East Asia | Japan | G2 | 0.37 | 0.01 |
| S271 | 6.51 | 93.54 | 37.4 | 98.38 | East Asia | China | G2 | 0.28 | 0.02 | S735 | 5.47 | 92.2 | 36.7 | 99.11 | East Asia | Japan | G1 | 0.06 | 0.02 |
| S272 | 5.83 | 93.35 | 37.73 | 95.39 | East Asia | China | G2 | 0.68 | 0.01 | S736 | 4.55 | 92.41 | 37.02 | 98.6 | Centra Africa | Guinea | G2 | 0.26 | 0.02 |
| S273 | 4.91 | 93.74 | 38.65 | 91.82 | East Asia | China | G3 | - | 0.02 | S737 | 4.37 | 92.67 | 36.83 | 98.72 | Southern Europe | Greece | G2 | 0.15 | 0.03 |
| S274 | 6.37 | 93.39 | 38.05 | 97.44 | East Asia | China | G2 | 0.44 | 0.02 | S738 | 7.35 | 92.33 | 36.99 | 98.91 | South America | Venezuela | G2 | 0.04 | 0.02 |
| S275 | 5.77 | 93.35 | 38.16 | 97.25 | East Asia | China | G2 | 0.59 | 0.02 | S739 | 4.52 | 92.27 | 37.33 | 98.8 | South America | Venezuela | G2 | 0.16 | 0.02 |
| S276 | 8.47 | 93.06 | 37.67 | 95.21 | East Asia | China | G2 | 0.35 | 0.02 | S740 | 4.08 | 91.48 | 37.61 | 98.65 | South-East Asia | Thailand | G2 | 0.23 | 0.01 |
| S277 | 5.75 | 93.51 | 37.57 | 97.31 | East Asia | China | G2 | 0.49 | 0.01 | S741 | 5.07 | 92.91 | 36.86 | 98.53 | East Asia | China | G2 | 0.09 | 0.03 |
| S278 | 5.03 | 93.51 | 38.3 | 94.95 | East Asia | China | G2 | - | 0.02 | S742 | 4.77 | 92.76 | 36.73 | 98.29 | East Asia | China | G2 | 0.17 | 0.02 |
| S279 | 5.88 | 93.53 | 37.53 | 97.78 | East Asia | China | G2 | 0.46 | 0.02 | S743 | 4.04 | 90.99 | 36.67 | 99.06 | East Asia | China | G2 | 0.2 | 0.01 |
| S280 | 4.74 | 93.61 | 37.51 | 98.47 | East Asia | China | G2 | 0.55 | 0.01 | S744 | 4.41 | 92.64 | 36.82 | 98.89 | East Asia | China | G2 | 0.17 | 0.02 |
| S281 | 5.41 | 92.67 | 37.52 | 97 | East Asia | China | G2 | 0.47 | 0.02 | S745 | 3.56 | 91.64 | 36.45 | 98.92 | East Asia | China | G2 | 0.41 | 0.01 |
| S282 | 4.26 | 92.56 | 37.67 | 95.78 | East Asia | China | G2 | - | 0.02 | S746 | 4.98 | 92.66 | 36.66 | 98.69 | East Asia | China | G2 | 0.1 | 0.02 |
| S283 | 8.21 | 92.83 | 37.7 | 94.73 | East Asia | China | G2 | 0.3 | 0.02 | S747 | 3.68 | 90.8 | 36.41 | 98.57 | East Asia | China | G2 | 0.34 | 0.02 |
| S284 | 5.85 | 92.13 | 37.61 | 94.4 | East Asia | China | G2 | 0.45 | 0.02 | S748 | 5.04 | 91.41 | 36.59 | 98.77 | East Asia | China | G2 | 0.21 | 0.01 |
| S285 | 4.95 | 92.96 | 37.3 | 97.74 | East Asia | China | G2 | 0.64 | 0.09 | S749 | 4.69 | 92.77 | 36.92 | 98.9 | East Asia | China | G2 | 0.11 | 0.02 |
| S286 | 6.34 | 92.72 | 38.63 | 96.07 | East Asia | China | G3 | 0.45 | 0.02 | S750 | 3.86 | 91.47 | 36.48 | 98.72 | East Asia | China | G2 | 0.27 | 0.01 |
| S287 | 5.07 | 93.16 | 38.37 | 95.2 | East Asia | China | G2 | - | 0.01 | S751 | 4.61 | 91.47 | 36.55 | 98.8 | East Asia | China | G2 | 0.17 | 0.01 |
| S288 | 6.24 | 92.52 | 37.97 | 94.99 | East Asia | China | G3 | - | 0.01 | S752 | 4.78 | 92.41 | 37.05 | 98.57 | East Asia | China | G2 | 0.11 | 0.02 |
| S289 | 4.83 | 93.58 | 37.46 | 95.12 | East Asia | China | G2 | 0.58 | 0.01 | S753 | 4.55 | 93.08 | 36.87 | 98.89 | East Asia | China | G2 | 0.16 | 0.02 |
| S290 | 5.41 | 93.88 | 37.91 | 97.61 | East Asia | China | G2 | 0.47 | 0.01 | S754 | 4.75 | 91.86 | 36.98 | 98.91 | East Asia | China | G2 | 0.18 | 0.01 |
| S291 | 5.55 | 92.92 | 37.58 | 97.84 | East Asia | China | G2 | 0.28 | 0.02 | S755 | 4.19 | 91.44 | 36.89 | 98.36 | East Asia | China | G2 | 0.23 | 0.01 |
| S292 | 5.31 | 93.63 | 37.96 | 96.58 | East Asia | China | G3 | 0.49 | 0.01 | S756 | 4.47 | 92.62 | 36.85 | 98.65 | East Asia | China | G2 | 0.23 | 0.02 |
| S293 | 8.17 | 93.51 | 38.06 | 95.86 | East Asia | China | G2 | 0.26 | 0.01 | S757 | 3.95 | 91.97 | 36.53 | 98.83 | East Asia | China | G3 | 0.24 | 0.01 |
| S294 | 4.48 | 91.49 | 37.75 | 94.55 | East Asia | China | G2 | - | 0.01 | S758 | 4.8 | 91.6 | 36.89 | 98.51 | East Asia | China | G3 | 0.28 | 0.01 |
| S295 | 5.17 | 92.33 | 37.99 | 92.03 | East Asia | China | G2 | - | 0.01 | S759 | 4.15 | 91.12 | 36.29 | 98.56 | East Asia | China | G3 | 0.22 | 0.01 |
| S296 | 5.06 | 93.77 | 37.33 | 96.96 | East Asia | China | G3 | 0.22 | 0.01 | S760 | 4.13 | 91.17 | 37.04 | 98.72 | East Asia | China | G3 | 0.31 | 0.01 |
| S297 | 3.83 | 94.22 | 38.52 | 88.77 | East Asia | China | G2 | - | 0.01 | S761 | 3.76 | 91.05 | 36.55 | 98.32 | East Asia | China | G2 | 0.36 | 0.01 |
| S298 | 5.42 | 93.72 | 39.38 | 93.08 | East Asia | China | G3 | - | 0.01 | S762 | 4.83 | 92.44 | 36.36 | 98.79 | East Asia | China | G2 | 0.15 | 0.02 |
| S299 | 6.48 | 93.81 | 37.75 | 96.89 | East Asia | China | G2 | - | 0.02 | S763 | 5.47 | 91.61 | 37.03 | 98.49 | East Asia | China | G2 | 0.14 | 0.01 |
| S300 | 8.01 | 93.58 | 38.31 | 92.24 | East Asia | China | G2 | 0.54 | 0.01 | S764 | 4.69 | 90 | 37.53 | 99.02 | East Asia | China | G2 | 0.24 | 0.01 |
| S301 | 6.1 | 93.31 | 38.14 | 96.52 | East Asia | China | G2 | 0.47 | 0.01 | S765 | 4.16 | 90.48 | 37.2 | 99.06 | East Asia | China | G2 | 0.33 | 0.01 |
| S302 | 6.48 | 93.25 | 37.79 | 97.44 | East Asia | China | G3 | 0.25 | 0.01 | S766 | 4.55 | 91.97 | 36.86 | 98.4 | East Asia | China | G2 | 0.26 | 0.02 |
| S303 | 6.57 | 93.59 | 37.73 | 97.69 | East Asia | China | G1 | 0.19 | 0.01 | S767 | 4.36 | 91.28 | 37.05 | 98.7 | East Asia | China | G1 | 0.31 | 0.02 |
| S304 | 6.81 | 93.76 | 37.75 | 97.2 | East Asia | China | G2 | 0.27 | 0.01 | S768 | 4.47 | 92.84 | 36.7 | 98.48 | East Asia | China | G2 | 0.15 | 0.02 |
| S305 | 7.16 | 93.16 | 38.13 | 95.87 | East Asia | China | G2 | 0.44 | 0.01 | S769 | 5.33 | 91.66 | 36.36 | 98.85 | East Asia | China | G2 | 0.13 | 0.01 |
| S306 | 4.67 | 93 | 38.4 | 93.34 | East Asia | China | G2 | - | 0.01 | S770 | 5.23 | 91.44 | 37.12 | 98.15 | East Asia | China | G2 | 0.18 | 0.01 |
| S307 | 5.82 | 94.06 | 38.07 | 97.06 | East Asia | China | G2 | 0.53 | 0.02 | S771 | 5.12 | 92.56 | 36.45 | 98.7 | East Asia | China | G2 | 0.07 | 0.03 |
| S308 | 7.29 | 93.21 | 37.8 | 96.88 | East Asia | China | G2 | 0.19 | 0.01 | S772 | 4.18 | 90.34 | 37.23 | 98.57 | East Asia | China | G1 | 0.27 | 0.01 |
| S309 | 5.71 | 93.66 | 38.16 | 96.94 | East Asia | China | G2 | 0.43 | 0.01 | S773 | 4.52 | 90.84 | 37.3 | 98.65 | East Asia | China | G1 | 0.24 | 0.01 |
| S310 | 6.39 | 93.57 | 38.03 | 95.71 | East Asia | China | G2 | 0.49 | 0.01 | S774 | 4.29 | 91.06 | 36.87 | 97.92 | East Asia | China | G2 | 0.27 | 0.01 |
| S311 | 4 | 93.3 | 38.64 | 90.64 | East Asia | China | G2 | - | 0.01 | S775 | 3.55 | 92.26 | 37.17 | 98.74 | East Asia | China | G1 | 0.24 | 0.01 |
| S312 | 6.31 | 93.49 | 38.06 | 97.21 | East Asia | China | G2 | 0.31 | 0.01 | S776 | 3.55 | 92.79 | 36.7 | 98.75 | East Asia | China | G1 | 0.27 | 0.02 |
| S313 | 4.7 | 94 | 38.03 | 95.16 | East Asia | China | G2 | - | 0.01 | S777 | 3.18 | 92.67 | 36.84 | 98.87 | East Asia | China | G1 | 0.23 | 0.01 |
| S314 | 6.4 | 93.35 | 37.83 | 95.65 | East Asia | China | G3 | 0.39 | 0.02 | S778 | 5.39 | 91.86 | 36.7 | 99.17 | East Asia | China | G2 | 0.13 | 0.02 |
| S315 | 10.65 | 94.23 | 37.57 | 98.17 | East Asia | China | G2 | 0.1 | 0.01 | S779 | 4.87 | 92.71 | 36.62 | 98.53 | East Asia | China | G1 | 0.14 | 0.02 |
| S316 | 10.83 | 93.35 | 37.89 | 97.36 | East Asia | China | G3 | 0.1 | 0.01 | S780 | 5.2 | 91.5 | 36.73 | 98.44 | East Asia | China | G2 | 0.16 | 0.02 |
| S317 | 4.8 | 94.03 | 37.53 | 96.11 | East Asia | China | G2 | 0.54 | 0.02 | S781 | 5.3 | 92.94 | 36.84 | 98.8 | East Asia | China | G2 | 0.1 | 0.02 |
| S318 | 5.27 | 93.92 | 38.22 | 91.08 | East Asia | China | G2 | 0.64 | 0.02 | S782 | 4.9 | 91.28 | 38.64 | 98.49 | East Asia | China | G1 | 0.27 | 0.02 |
| S319 | 7.19 | 93.92 | 38.34 | 96.48 | East Asia | China | G2 | 0.54 | 0.02 | S783 | 5.43 | 93.27 | 36.81 | 98.64 | East Asia | China | G2 | 0.14 | 0.02 |
| S320 | 5.78 | 93.62 | 37.9 | 95.78 | East Asia | China | G2 | 0.31 | 0.01 | S784 | 4.74 | 91.23 | 36.53 | 98.87 | East Asia | China | G1 | 0.14 | 0.02 |
| S321 | 6.63 | 94.12 | 38 | 96.28 | East Asia | China | G2 | 0.45 | 0.02 | S785 | 5.06 | 90.81 | 36.27 | 98.6 | East Asia | China | G1 | 0.15 | 0.02 |
| S322 | 7.11 | 93.72 | 38.08 | 96.65 | East Asia | China | G2 | 0.28 | 0.02 | S786 | 5.21 | 91.97 | 36.64 | 98.45 | East Asia | China | G1 | 0.14 | 0.02 |
| S323 | 7.1 | 93.22 | 38.38 | 94.58 | East Asia | China | G2 | 0.51 | 0.02 | S787 | 4.19 | 92.09 | 36.75 | 98.68 | East Asia | China | G2 | 0.26 | 0.02 |
| S324 | 5.42 | 93.14 | 37.84 | 95.25 | East Asia | China | G2 | 0.56 | 0.01 | S788 | 4.45 | 90.78 | 36.48 | 99.24 | East Asia | China | G1 | 0.13 | 0.02 |
| S325 | 4.39 | 94.13 | 38.16 | 93.52 | East Asia | China | G2 | - | 0.01 | S789 | 5.28 | 91.38 | 36.6 | 98.79 | East Asia | China | G1 | 0.15 | 0.02 |
| S326 | 6.01 | 93.36 | 38.09 | 93.4 | East Asia | China | G2 | 0.75 | 0.02 | S790 | 4.07 | 90.91 | 36.59 | 98.97 | East Asia | China | G1 | 0.29 | 0.01 |
| S327 | 4.88 | 92.66 | 38.35 | 93.47 | East Asia | China | G2 | - | 0.01 | S791 | 4.24 | 91.68 | 37.26 | 98.73 | East Asia | China | G1 | 0.28 | 0.02 |
| S328 | 5.85 | 92.75 | 38.23 | 93.73 | East Asia | China | G2 | 0.75 | 0.02 | S792 | 4.83 | 89.98 | 36.81 | 98.68 | East Asia | China | G1 | 0.14 | 0.02 |
| S329 | 4.65 | 92.56 | 37.84 | 96.47 | East Asia | China | G2 | - | 0.02 | S793 | 4.18 | 91.35 | 36.78 | 98.5 | East Asia | China | G1 | 0.23 | 0.02 |
| S330 | 7.03 | 92.66 | 37.97 | 97.24 | East Asia | China | G2 | 0.23 | 0.01 | S794 | 5.22 | 91.71 | 36.97 | 98.93 | East Asia | China | G1 | 0.11 | 0.02 |
| S331 | 4.26 | 92.72 | 37.63 | 95.73 | East Asia | China | G2 | 0.67 | 0.02 | S795 | 4.36 | 91.7 | 36.36 | 98.91 | East Asia | China | G1 | 0.22 | 0.02 |
| S332 | 3.65 | 91.47 | 38.12 | 94.54 | East Asia | China | G2 | - | 0.01 | S796 | 3.78 | 91.59 | 37.19 | 98.64 | East Asia | China | G1 | 0.45 | 0.03 |
| S333 | 4.87 | 93.25 | 38.06 | 96.62 | East Asia | China | G2 | 0.6 | 0.01 | S797 | 5.41 | 91.08 | 37 | 98.2 | East Asia | China | G1 | 0.21 | 0.02 |
| S334 | 4.87 | 93.85 | 38.44 | 92.29 | East Asia | China | G4 | - | 0.01 | S798 | 6.54 | 91.79 | 37.62 | 98.53 | East Asia | China | G1 | 0.12 | 0.02 |
| S335 | 5.9 | 93.44 | 38.02 | 95.95 | East Asia | China | G3 | 0.45 | 0.02 | S799 | 4.22 | 92.59 | 37.2 | 98.21 | East Asia | China | G1 | 0.57 | 0.02 |
| S336 | 5.81 | 93.91 | 37.88 | 96.69 | East Asia | China | G2 | 0.59 | 0.02 | S800 | 5.41 | 91 | 36.95 | 98.5 | East Asia | China | G1 | 0.18 | 0.03 |
| S337 | 5.58 | 93.37 | 37.94 | 96.77 | East Asia | China | G2 | 0.55 | 0.01 | S801 | 5.84 | 91.41 | 36.7 | 98.77 | East Asia | China | G3 | 0.1 | 0.02 |
| S338 | 6.54 | 92.96 | 38.8 | 96.46 | East Asia | China | G3 | 0.45 | 0.02 | S802 | 3.6 | 92.31 | 36.42 | 98.55 | East Asia | China | G3 | 0.34 | 0.02 |
| S339 | 7.92 | 92.92 | 38.27 | 96.26 | East Asia | China | G2 | 0.26 | 0.02 | S803 | 4.4 | 91.96 | 36.01 | 99.12 | East Asia | China | G3 | 0.2 | 0.03 |
| S340 | 4.8 | 93.14 | 38.17 | 95 | East Asia | China | G3 | - | 0.02 | S804 | 3.33 | 90.88 | 36.44 | 98.87 | East Asia | China | G2 | 0.31 | 0.02 |
| S341 | 5.78 | 92.37 | 38.09 | 93.16 | East Asia | China | G3 | 0.6 | 0.02 | S805 | 4.6 | 90.41 | 36.08 | 99.02 | East Asia | China | G3 | 0.16 | 0.01 |
| S342 | 8.7 | 92.44 | 37.88 | 96.92 | East Asia | China | G2 | 0.18 | 0.02 | S806 | 4.42 | 91.43 | 37.14 | 98.75 | East Asia | China | G3 | 0.17 | 0.02 |
| S343 | 4.89 | 93.01 | 37.92 | 96.53 | East Asia | China | G2 | 0.58 | 0.01 | S807 | 5.27 | 91.14 | 37.27 | 98.68 | East Asia | China | G3 | 0.15 | 0.02 |
| S344 | 8.61 | 92.34 | 37.98 | 95.67 | East Asia | China | G2 | 0.21 | 0.01 | S808 | 3.51 | 91.86 | 36.06 | 98.92 | East Asia | China | G3 | 0.27 | 0.03 |
| S345 | 15.29 | 92.93 | 38.28 | 89.5 | East Asia | China | G2 | 0.11 | 0.02 | S809 | 5.33 | 91.81 | 37.09 | 98.26 | East Asia | China | G2 | 0.22 | 0.02 |
| S346 | 6.26 | 92.63 | 37.78 | 96.51 | East Asia | China | G1 | 0.51 | 0.02 | S810 | 3 | 91.85 | 36.7 | 97.97 | East Asia | China | G2 | - | 0.01 |
| S347 | 8.11 | 92.97 | 37.92 | 96.68 | East Asia | China | G1 | 0.25 | 0.03 | S811 | 5.17 | 90.64 | 36.34 | 97.94 | East Asia | China | G2 | 0.28 | 0.01 |
| S348 | 6.66 | 92.9 | 37.75 | 97.11 | East Asia | China | G1 | 0.15 | 0.02 | S812 | 6.26 | 95.31 | 40.37 | 97.25 | East Asia | China | G2 | 0.96 | 0.01 |
| S349 | 5.42 | 92.81 | 38.42 | 92.41 | East Asia | China | G2 | - | 0.02 | S813 | 6.19 | 94.86 | 38.88 | 96.28 | East Asia | China | G2 | 0.87 | 0.01 |
| S350 | 5.42 | 92.59 | 38.15 | 93.31 | East Asia | China | G1 | 0.34 | 0.02 | S814 | 4.53 | 91.66 | 36.98 | 97.98 | East Asia | China | G1 | 0.48 | 0.01 |
| S351 | 5.49 | 92.91 | 37.6 | 92.5 | East Asia | China | G1 | 0.66 | 0.02 | S815 | 1.62 | 90.48 | 37.37 | 94.77 | South Asia | India | G4 | - | 0.01 |
| S352 | 7.4 | 92.59 | 37.27 | 97.63 | East Asia | China | G1 | 0.12 | 0.03 | S816 | 7.84 | 94.16 | 39.09 | 91.18 | South Asia | India | G2 | - | 0.1 |
| S353 | 6.27 | 92.89 | 38.57 | 95.07 | East Asia | China | G2 | 0.26 | 0.02 | S817 | 6.33 | 91.68 | 36.4 | 98.52 | South Asia | India | G4 | 0.15 | 0.01 |
| S354 | 6.99 | 92.78 | 37.41 | 97.46 | East Asia | China | G2 | 0.13 | 0.01 | S818 | 4.75 | 91.82 | 37.15 | 97.76 | South Asia | India | G4 | 0.52 | 0.01 |
| S355 | 6.48 | 92.62 | 38.48 | 95.15 | East Asia | China | G2 | - | 0.02 | S819 | 6.38 | 91.96 | 36.92 | 98.72 | East Africa | China | G4 | 0.28 | 0.02 |
| S356 | 5.12 | 92.74 | 38.11 | 95.14 | East Asia | China | G1 | 0.5 | 0.02 | S820 | 5.11 | 91.58 | 37.21 | 97.52 | East Africa | China | G4 | 0.31 | 0.01 |
| S357 | 4.42 | 92.42 | 38.38 | 90.09 | East Asia | China | G1 | - | 0.02 | S821 | 6.55 | 94.17 | 38.66 | 92.69 | South Africa | China | G3 | 0.74 | 0.01 |
| S358 | 5.05 | 92.71 | 37.42 | 96.03 | East Asia | China | G2 | 0.48 | 0.07 | S822 | 4.04 | 92.06 | 36.34 | 98.7 | East Africa | Sudan | G4 | 0.45 | 0.03 |
| S359 | 3.44 | 93.68 | 37.95 | 95.14 | East Asia | China | G1 | - | 0.01 | S823 | 6.82 | 93.59 | 40.16 | 87.15 | East Africa | Sudan | G4 | - | 0.01 |
| S360 | 6.07 | 92.81 | 38.21 | 97.69 | East Asia | China | G1 | 0.24 | 0.02 | S824 | 3.74 | 91.67 | 36.27 | 97.77 | East Africa | Sudan | G4 | 0.45 | 0.01 |
| S361 | 5.94 | 92.43 | 38.36 | 96.4 | East Asia | China | G2 | 0.35 | 0.02 | S825 | 6.48 | 93.86 | 40.03 | 94.8 | South-East Asia | Myanmar | G3 | 0.65 | 0.01 |
| S362 | 7.12 | 92.77 | 37.91 | 95.61 | East Asia | China | G2 | 0.3 | 0.02 | S826 | 4.56 | 91.77 | 37.44 | 97.91 | South-East Asia | Myanmar | G4 | 0.43 | 0.01 |
| S363 | 4.54 | 92.7 | 37.51 | 97.37 | East Asia | China | G2 | 0.61 | 0.01 | S827 | 4.95 | 92.03 | 37.67 | 97.08 | Central America | Mexico | G4 | 0.58 | 0.01 |
| S364 | 6.39 | 93.22 | 37.22 | 96.63 | East Asia | China | G2 | 0.16 | 0.02 | S828 | 2.6 | 91.41 | 36.47 | 96.42 | Central America | Mexico | G4 | - | 0.01 |
| S365 | 4.6 | 93.21 | 38.3 | 94.59 | East Asia | China | G1 | - | 0.02 | S829 | 6.9 | 93.43 | 38.25 | 96.18 | Central America | Mexico | G3 | 0.38 | 0.02 |
| S366 | 5.67 | 93 | 38.12 | 95.43 | East Asia | China | G1 | 0.34 | 0.02 | S830 | 4.52 | 91.67 | 36.74 | 98.53 | West Africa | Togo | G2 | 0.52 | 0.01 |
| S367 | 4.45 | 92.48 | 37.51 | 97.32 | East Asia | China | G1 | 0.31 | 0.02 | S831 | 4.58 | 90.96 | 37.11 | 95.56 | East Africa | United Republic of Tanzania | G4 | 0.46 | 0.01 |
| S368 | 4.04 | 93.11 | 37.14 | 97.67 | East Asia | China | G1 | 0.72 | 0.02 | S832 | 4.38 | 91.86 | 36.96 | 97.32 | East Africa | United Republic of Tanzania | G4 | 0.45 | 0.01 |
| S369 | 5.52 | 92.75 | 37.45 | 97.71 | East Asia | China | G1 | 0.27 | 0.02 | S833 | 5.23 | 92.84 | 37.11 | 95.93 | West Asia | China | G3 | 0.52 | 0.05 |
| S370 | 5.93 | 92.85 | 37.52 | 97.65 | East Asia | China | G1 | 0.25 | 0.02 | S834 | 6.02 | 94.92 | 41.33 | 89.56 | West Asia | China | G4 | - | 0.01 |
| S371 | 5.95 | 92.95 | 37.94 | 97.44 | East Asia | China | G1 | 0.31 | 0.02 | S835 | 8.78 | 95.53 | 39.87 | 95.42 | East Africa | Uganda | G4 | 0.86 | 0.03 |
| S372 | 4 | 92.83 | 38.27 | 96.99 | East Asia | China | G1 | - | 0.01 | S836 | 8.37 | 87.23 | 35.18 | 96.33 | Centra Africa | Congo | G4 | 0.7 | 0.01 |
| S373 | 3.94 | 92.92 | 37.2 | 97.06 | East Asia | China | G1 | 0.46 | 0.01 | S837 | 6.01 | 91.8 | 36.98 | 97.82 | Centra Africa | China | G3 | 0.28 | 0.01 |
| S374 | 4.91 | 92.97 | 37.62 | 97.38 | East Asia | China | G1 | 0.28 | 0.02 | S838 | 5.01 | 91.84 | 37.17 | 97.35 | West Africa | Mali | G4 | 0.48 | 0.01 |
| S375 | 4.7 | 93.11 | 38.02 | 96.68 | East Asia | China | G1 | 0.37 | 0.02 | S839 | 4 | 91.22 | 39.93 | 93.5 | West Africa | Mali | G4 | - | 0.02 |
| S376 | 4.07 | 93.16 | 37.83 | 97.24 | East Asia | China | G1 | 0.41 | 0.02 | S840 | 9.03 | 90.91 | 36.91 | 95.89 | East Africa | China | G4 | 0.71 | 0.02 |
| S377 | 3.75 | 93.04 | 37.87 | 95.81 | East Asia | China | G1 | - | 0.03 | S841 | 6.96 | 93.34 | 40.84 | 89.86 | East Asia | Democratic People's Republic of Korea | G1 | - | 0.01 |
| S378 | 6.62 | 93.25 | 38.03 | 97.34 | East Asia | China | G3 | 0.23 | 0.03 | S842 | 5.47 | 93.26 | 39.35 | 97.19 | East Asia | China | G1 | 0.89 | 0.01 |
| S379 | 6.04 | 93 | 37.99 | 96.96 | East Asia | China | G2 | 0.22 | 0.02 | S843 | 7.6 | 95.33 | 37.16 | 95.67 | South-East Asia | Viet Nam | G3 | 0.6 | 0.1 |
| S380 | 4.92 | 93.38 | 37.29 | 97.79 | East Asia | China | G1 | 0.22 | 0.02 | S844 | 7.05 | 93.84 | 36.76 | 96.07 | North America | United States of America | G2 | 0.31 | 0.02 |
| S381 | 3.49 | 92.75 | 37.95 | 95.63 | East Asia | China | G1 | - | 0.02 | S845 | 4.56 | 91.7 | 37 | 97.48 | North America | United States of America | G2 | 0.52 | 0.01 |
| S382 | 4.2 | 93.36 | 38.02 | 95.34 | East Asia | China | G1 | 0.63 | 0.02 | S846 | 6.11 | 94.91 | 40.95 | 88.73 | East Asia | Japan | G2 | - | 0.04 |
| S383 | 4.21 | 93.43 | 37.9 | 95.57 | East Asia | China | G3 | - | 0.02 | S847 | 5.67 | 92.53 | 40.48 | 91.74 | South Asia | Sri Lanka | G2 | 1.04 | 0.01 |
| S384 | 3.76 | 93.8 | 38.09 | 89.99 | East Asia | China | G1 | - | 0.01 | S848 | 7.27 | 92.63 | 40.35 | 92.58 | Centra Africa | Guinea | G2 | 1.17 | 0.04 |
| S385 | 3.62 | 93.35 | 38.67 | 86.28 | East Asia | China | G2 | - | 0.01 | S849 | 4.08 | 91.35 | 40.34 | 89.91 | Southern Europe | Greece | G3 | - | 0.01 |
| S386 | 4.4 | 92.91 | 37.63 | 96.71 | East Asia | China | G1 | 0.76 | 0.01 | S850 | 6.65 | 92.66 | 39.42 | 96.84 | South America | Venezuela | G2 | 0.83 | 0.01 |
| S387 | 7.12 | 92.97 | 37.11 | 97.66 | East Asia | China | G2 | 0.2 | 0.01 | S851 | 4.27 | 95.43 | 37.25 | 94.96 | South-East Asia | Indonesia | G3 | - | 0.04 |
| S388 | 6.31 | 92.3 | 37.9 | 92.26 | East Asia | China | G1 | - | 0.02 | S852 | 6.1 | 93.91 | 38.7 | 95.97 | South-East Asia | Thailand | G3 | 0.86 | 0.04 |
| S389 | 8.88 | 92.72 | 37.5 | 93.51 | East Asia | China | G2 | 0.36 | 0.01 | S853 | 5.54 | 91.18 | 39.64 | 93.05 | Caribbean | Cuba | G3 | 0.94 | 0.01 |
| S390 | 7.32 | 92.97 | 37.56 | 97.06 | East Asia | China | G2 | 0.25 | 0.01 | S854 | 3.19 | 91.45 | 40.19 | 90.26 | South Asia | Pakistan | G4 | - | 0.02 |
| S391 | 8.63 | 93.24 | 37.58 | 96.52 | East Asia | China | G1 | 0.33 | 0.02 | S855 | 6.29 | 94.75 | 40 | 93.31 | East Africa | Somalia | G4 | 0.91 | 0.01 |
| S392 | 8.8 | 93.66 | 37.54 | 97.16 | East Asia | China | G1 | 0.23 | 0.01 | S856 | 7.06 | 94.72 | 44.52 | 95.7 | East Asia | China | G2 | 1.08 | 0.01 |
| S393 | 7.35 | 93.03 | 37.49 | 97.2 | East Asia | China | G1 | 0.24 | 0.01 | S857 | 8.59 | 94.44 | 42.63 | 93.28 | East Asia | China | G2 | 0.71 | 0.01 |
| S394 | 6.67 | 93.18 | 37.74 | 96.26 | East Asia | China | G2 | 0.6 | 0.01 | S858 | 8.25 | 95.63 | 39.34 | 96.99 | East Asia | China | G2 | 0.78 | 0.01 |
| S395 | 8.66 | 93.15 | 37.41 | 97.08 | East Asia | China | G1 | 0.22 | 0.02 | S859 | 6.67 | 91.44 | 36.38 | 98.18 | East Asia | China | G2 | 0.19 | 0.01 |
| S396 | 5.27 | 92.75 | 37.67 | 97.18 | East Asia | China | G1 | 0.53 | 0.01 | S860 | 6.01 | 94.71 | 37.61 | 95.52 | East Asia | China | G3 | 0.42 | 0.01 |
| S397 | 5.65 | 93.18 | 37.2 | 96.03 | East Asia | China | G2 | 0.6 | 0.01 | S861 | 4.65 | 91.08 | 36.13 | 98.6 | East Asia | China | G3 | 0.28 | 0.01 |
| S398 | 6.79 | 92.84 | 37.67 | 96.7 | East Asia | China | G2 | 0.32 | 0.01 | S862 | 6.51 | 94.67 | 42.75 | 83.72 | East Asia | China | G2 | - | 0.01 |
| S399 | 6.5 | 92.91 | 37.77 | 93.3 | East Asia | China | G1 | 0.92 | 0.01 | S863 | 4.79 | 91.69 | 36.33 | 98.37 | East Asia | China | G3 | 0.35 | 0.02 |
| S400 | 5.76 | 92.8 | 37.36 | 97.25 | East Asia | China | G1 | 0.39 | 0.01 | S864 | 4.62 | 95.81 | 39.08 | 94.3 | West Africa | Togo | G4 | 0.91 | 0.03 |
| S401 | 7.02 | 92.16 | 38.35 | 96.41 | East Asia | China | G1 | 0.38 | 0.02 | S865 | 4.82 | 92.31 | 35.8 | 97.7 | South Asia | Bangladesh | G4 | 0.43 | 0.01 |
| S402 | 6.81 | 93.4 | 37.19 | 97.82 | East Asia | China | G2 | 0.2 | 0.02 | S866 | 3.09 | 94.98 | 36.95 | 98.82 | East Asia | China | G2 | - | 0.01 |
| S403 | 6.86 | 92.56 | 37.39 | 98.2 | East Asia | China | G1 | 0.21 | 0.02 | S867 | 3.5 | 95.02 | 36.41 | 99 | East Asia | China | G2 | 0.5 | 0.01 |
| S404 | 4.34 | 93.17 | 37.73 | 96.55 | East Asia | China | G1 | 0.75 | 0.01 | S868 | 3.56 | 94.61 | 36.79 | 98.71 | East Asia | China | G2 | 0.53 | 0.01 |
| S405 | 6.82 | 92.99 | 37.51 | 97.29 | East Asia | China | G1 | 0.26 | 0.01 | S869 | 3.96 | 94.64 | 36.8 | 98.73 | East Asia | China | G1 | 0.56 | 0.01 |
| S406 | 7.74 | 92.58 | 37.81 | 91.73 | East Asia | China | G1 | 0.54 | 0.01 | S870 | 3.93 | 94.77 | 36.78 | 98.91 | East Asia | China | G2 | 0.56 | 0.01 |
| S407 | 6.7 | 93.1 | 36.84 | 98.3 | East Asia | China | G1 | 0.16 | 0.01 | S871 | 3.71 | 94.83 | 36.89 | 98.63 | East Asia | China | G1 | 0.59 | 0.02 |
| S408 | 5.11 | 93 | 37.95 | 94.14 | East Asia | China | G1 | - | 0.02 | S872 | 3.41 | 94.89 | 36.38 | 98.57 | East Asia | China | G1 | 0.48 | 0.01 |
| S409 | 6.87 | 93.54 | 36.89 | 98.12 | East Asia | China | G1 | 0.19 | 0.01 | S873 | 7.86 | 94.63 | 37.06 | 98.62 | East Asia | China | G1 | 0.22 | 0.02 |
| S410 | 6.36 | 92.84 | 37.45 | 98.16 | East Asia | China | G1 | 0.23 | 0.02 | S874 | 3.48 | 94.62 | 36.9 | 98.64 | East Asia | China | G2 | 0.52 | 0.01 |
| S411 | 4.68 | 93.25 | 37.33 | 97.76 | East Asia | China | G2 | 0.61 | 0.02 | S875 | 3.39 | 94.48 | 37.42 | 98.57 | East Asia | China | G1 | - | 0.02 |
| S412 | 4.87 | 93.3 | 37.66 | 96.49 | East Asia | China | G2 | 0.68 | 0.02 | S876 | 3.43 | 95.22 | 36.58 | 98.86 | East Asia | China | G2 | 0.32 | 0.01 |
| S413 | 6.05 | 93.31 | 37.55 | 94.34 | East Asia | China | G1 | 0.59 | 0.02 | S877 | 3.65 | 94.88 | 36.22 | 98.7 | East Asia | China | G1 | 0.63 | 0.01 |
| S414 | 6.06 | 92.77 | 37.5 | 96.89 | East Asia | China | G1 | 0.46 | 0.02 | S878 | 3.4 | 95.03 | 36.91 | 98.06 | East Asia | China | G1 | 0.28 | 0.01 |
| S415 | 7.66 | 92.99 | 37.22 | 96.63 | East Asia | China | G1 | 0.24 | 0.02 | S879 | 3.54 | 95.05 | 37.01 | 98.89 | East Asia | China | G1 | 0.26 | 0.01 |
| S416 | 7.25 | 93.07 | 37.47 | 95.26 | East Asia | China | G1 | 0.45 | 0.02 | S880 | 3.56 | 94.91 | 36.77 | 98.81 | East Asia | China | G1 | 0.38 | 0.01 |
| S417 | 7.8 | 92.61 | 37.86 | 96.28 | East Asia | China | G1 | 0.32 | 0.02 | S881 | 3.49 | 94.95 | 37.06 | 98.78 | East Asia | China | G3 | - | 0.01 |
| S418 | 8.09 | 92.34 | 37.99 | 96.76 | East Asia | China | G1 | 0.26 | 0.02 | S882 | 4.3 | 95.06 | 36.56 | 98.74 | East Asia | China | G2 | 0.58 | 0.01 |
| S419 | 5.77 | 93.49 | 37.5 | 94.05 | East Asia | China | G1 | 0.74 | 0.02 | S883 | 3.09 | 94.8 | 36.84 | 98.94 | East Asia | China | G2 | 0.46 | 0.01 |
| S420 | 5.01 | 93.14 | 37.27 | 97.85 | East Asia | China | G1 | 0.39 | 0.02 | S884 | 2.86 | 94.48 | 36.81 | 98.68 | East Asia | China | G3 | - | 0.02 |
| S421 | 7.09 | 93.44 | 37.41 | 98.16 | East Asia | China | G1 | 0.26 | 0.02 | S885 | 2.87 | 94.22 | 36.28 | 98.76 | East Africa | China | G4 | - | 0.01 |
| S422 | 4.86 | 92.91 | 37.38 | 97.46 | East Asia | China | G1 | 0.59 | 0.02 | S886 | 3.74 | 94.38 | 36.52 | 98.54 | East Africa | Sudan | G4 | 0.63 | 0.01 |
| S423 | 11.29 | 93.27 | 37.27 | 96.84 | East Asia | China | G1 | 0.15 | 0.02 | S887 | 3.22 | 94.61 | 37.23 | 98.58 | East Africa | Sudan | G4 | 0.58 | 0.01 |
| S424 | 6.62 | 92.88 | 37.55 | 97.48 | East Asia | China | G1 | 0.35 | 0.02 | S888 | 3.87 | 94.58 | 36.16 | 98.86 | East Africa | Sudan | G4 | 0.59 | 0.01 |
| S425 | 9.06 | 92.88 | 37.52 | 96.14 | East Asia | China | G1 | 0.18 | 0.02 | S889 | 4.12 | 94.55 | 36.18 | 98.74 | East Africa | Sudan | G4 | 0.54 | 0.02 |
| S426 | 5.37 | 92.88 | 37.37 | 96.62 | East Asia | China | G2 | 0.41 | 0.01 | S890 | 3.33 | 94.3 | 36.42 | 98.48 | East Africa | Sudan | G4 | - | 0.05 |
| S427 | 9.18 | 93.53 | 37.64 | 97.83 | East Asia | China | G2 | 0.19 | 0.02 | S891 | 3.16 | 94.64 | 36.08 | 98.95 | East Africa | Sudan | G4 | 0.59 | 0.01 |
| S428 | 6.69 | 93.44 | 37.17 | 97.65 | East Asia | China | G1 | 0.3 | 0.02 | S892 | 3.64 | 94.5 | 36.24 | 98.89 | East Africa | Sudan | G4 | 0.72 | 0.04 |
| S429 | 6.84 | 92.75 | 37.14 | 96.93 | East Asia | China | G2 | 0.27 | 0.02 | S893 | 2.97 | 95.18 | 36.62 | 98.69 | East Africa | Sudan | G4 | - | 0.01 |
| S430 | 7.18 | 93.34 | 37.24 | 98.28 | East Asia | China | G2 | 0.21 | 0.01 | S894 | 3.01 | 95.13 | 36.84 | 99.12 | East Africa | South Sudan | G4 | 0.18 | 0.02 |
| S431 | 9.6 | 92.55 | 37.83 | 92.62 | East Asia | China | G2 | 0.36 | 0.02 | S895 | 3.42 | 95.02 | 36.33 | 99.26 | East Africa | South Sudan | G4 | 0.61 | 0.02 |
| S432 | 9.33 | 93.14 | 37.82 | 97.31 | East Asia | China | G1 | 0.16 | 0.02 | S896 | 4.05 | 95.19 | 36.74 | 98.98 | South-East Asia | Myanmar | G3 | 0.65 | 0.01 |
| S433 | 9.46 | 93.34 | 37.47 | 95.8 | East Asia | China | G1 | 0.14 | 0.02 | S897 | 3.42 | 95.08 | 36.8 | 98.77 | South-East Asia | Myanmar | G4 | 0.45 | 0.01 |
| S434 | 8.83 | 92.97 | 37.49 | 97.22 | East Asia | China | G1 | 0.14 | 0.02 | S898 | 3.22 | 95.02 | 36.61 | 99.12 | Centra Africa | Congo | G3 | 0.38 | 0.02 |
| S435 | 7.35 | 92.81 | 37.69 | 97.38 | East Asia | China | G2 | 0.19 | 0.02 | S899 | 4.54 | 95.09 | 36.53 | 99 | Centra Africa | Congo | G2 | 0.57 | 0.01 |
| S436 | 8.18 | 93.71 | 37.01 | 98.3 | East Asia | China | G1 | 0.16 | 0.02 | S900 | 3.14 | 94.61 | 36.58 | 98.99 | East Africa | Kenya | G4 | 0.47 | 0.01 |
| S437 | 8.05 | 92.92 | 37.72 | 97.79 | East Asia | China | G1 | 0.17 | 0.02 | S901 | 3.89 | 94.99 | 37.09 | 98.69 | East Asia | Japan | G2 | 0.29 | 0.01 |
| S438 | 6.25 | 90.61 | 37.64 | 97.59 | East Asia | China | G1 | 0.26 | 0.02 | S902 | 3.6 | 94.82 | 36.9 | 98.81 | Eastern Europe | Russian Federation | G2 | 0.63 | 0.02 |
| S439 | 7.32 | 90.92 | 37.32 | 97.83 | East Asia | China | G2 | 0.23 | 0.05 | S903 | 3.71 | 94.43 | 37.22 | 98.28 | South Asia | Bangladesh | G4 | 0.57 | 0.01 |
| S440 | 4.52 | 91.22 | 37.14 | 97.75 | East Asia | China | G2 | 0.57 | 0.01 | S904 | 4.44 | 94.75 | 36.26 | 98.85 | South Asia | Bangladesh | G4 | 0.49 | 0.02 |
| S441 | 3.98 | 90.98 | 37.99 | 96.63 | East Asia | China | G1 | - | 0.01 | S905 | 3.01 | 94.37 | 36.3 | 98.61 | South Asia | Bangladesh | G4 | - | 0.02 |
| S442 | 3.55 | 90.96 | 37.47 | 97.32 | East Asia | China | G1 | - | 0.02 | S906 | 3.63 | 94.6 | 37.34 | 98.43 | East Asia | China | G2 | 0.68 | 0.02 |
| S443 | 4.57 | 91.05 | 36.86 | 97.87 | East Asia | China | G2 | 0.52 | 0.01 | S907 | 3.63 | 94.63 | 36.73 | 98.68 | East Asia | China | G2 | 0.59 | 0.01 |
| S444 | 4.48 | 90.81 | 37.38 | 96.89 | East Asia | China | G2 | - | 0.02 | S908 | 3.2 | 94.49 | 37.39 | 98.57 | East Asia | China | G2 | - | 0.01 |
| S445 | 5.44 | 91.04 | 37.67 | 97.56 | East Asia | China | G2 | 0.4 | 0.01 | S909 | 3.48 | 94.93 | 36.53 | 99.17 | East Asia | China | G2 | 0.62 | 0.01 |
| S446 | 5.26 | 91.33 | 37.47 | 97.52 | East Asia | China | G2 | 0.36 | 0.01 | S910 | 3.44 | 94.69 | 36.69 | 98.89 | East Asia | China | G2 | 0.63 | 0.01 |
| S447 | 4.48 | 91.27 | 37.22 | 96.95 | East Asia | China | G2 | 0.71 | 0.01 | S911 | 3.01 | 95.08 | 36.21 | 98.88 | East Asia | China | G3 | 0.2 | 0.01 |
| S448 | 5.17 | 90.97 | 36.84 | 98.62 | East Asia | China | G2 | 0.28 | 0.01 | S912 | 3.23 | 94.84 | 37.14 | 99.21 | East Asia | China | G2 | 0.39 | 0.02 |
| S449 | 6.34 | 90.5 | 37.16 | 98.22 | East Asia | China | G2 | 0.19 | 0.01 | S913 | 3.49 | 95.12 | 36.98 | 98.91 | East Asia | China | G2 | 0.53 | 0.02 |
| S450 | 4.64 | 90.97 | 37.41 | 97.83 | East Asia | China | G2 | 0.61 | 0.02 | S914 | 3.3 | 94.7 | 36.66 | 99.14 | East Asia | China | G2 | 0.64 | 0.02 |
| S451 | 4.27 | 91.09 | 37.63 | 97.63 | East Asia | China | G1 | 0.69 | 0.02 | S915 | 3.22 | 94.79 | 37.12 | 98.83 | East Asia | China | G2 | - | 0.01 |
| S452 | 4.9 | 90.49 | 37.39 | 97.59 | East Asia | China | G2 | 0.45 | 0.01 | S916 | 3.94 | 94.6 | 37.09 | 98.8 | East Asia | China | G3 | 0.62 | 0.02 |
| S453 | 7.39 | 90.93 | 37.11 | 97.73 | East Asia | China | G2 | 0.19 | 0.02 | S917 | 3.6 | 94.61 | 36.94 | 99.08 | East Asia | China | G2 | 0.62 | 0.01 |
| S454 | 7.11 | 90.78 | 37.02 | 98.38 | East Asia | China | G2 | 0.16 | 0.01 | S918 | 3.22 | 95.52 | 36.13 | 99.14 | East Asia | China | G2 | - | 0.01 |
| S455 | 5.09 | 91.29 | 37.03 | 97.98 | East Asia | China | G2 | 0.5 | 0.02 | S919 | 3.13 | 94.53 | 36.53 | 98.7 | East Asia | China | G3 | - | 0.02 |
| S456 | 4.91 | 91.38 | 37.72 | 97.03 | East Asia | China | G2 | 0.72 | 0.02 | S920 | 3.58 | 95.2 | 37.02 | 99.05 | East Asia | China | G2 | 0.54 | 0.02 |
| S457 | 4.73 | 90.89 | 36.88 | 98.01 | East Asia | China | G2 | 0.49 | 0.02 | S921 | 3.18 | 94.99 | 36.65 | 99.04 | East Asia | China | G2 | 0.46 | 0.02 |
| S458 | 5.04 | 90.48 | 37.72 | 97.28 | East Asia | China | G2 | 0.5 | 0.02 | S922 | 3.42 | 95.05 | 36.9 | 99.12 | East Asia | China | G3 | 0.53 | 0.02 |
| S459 | 6.3 | 90.75 | 37.3 | 97.83 | East Asia | China | G1 | 0.27 | 0.03 | S923 | 3.56 | 95.16 | 37.49 | 99.03 | East Asia | China | G4 | 0.11 | 0.01 |
| S460 | 7.3 | 91.13 | 37.61 | 98.69 | East Asia | China | G2 | 0.2 | 0.02 | S924 | 3.96 | 94.67 | 36.53 | 98.77 | East Asia | China | G4 | 0.71 | 0.04 |
| S461 | 7.73 | 91.07 | 36.72 | 98.44 | East Asia | China | G2 | 0.15 | 0.02 | S925 | 3.72 | 94.94 | 37.2 | 98.24 | East Asia | China | G2 | 0.36 | 0.02 |
| S462 | 10.42 | 90.87 | 37.13 | 98.27 | East Asia | China | G1 | 0.1 | 0.02 | S926 | 3.34 | 94.85 | 36.89 | 98.79 | East Africa | Sudan | G4 | 0.53 | 0.01 |
| S463 | 6.9 | 90.68 | 37.28 | 97.84 | East Asia | China | G1 | 0.19 | 0.02 | S927 | 2.98 | 94.89 | 37.27 | 98.55 | East Africa | Sudan | G4 | - | 0.01 |
| S464 | 6.98 | 90.8 | 37.79 | 97.34 | East Asia | China | G1 | 0.27 | 0.02 | Total/Mean | 52.89 | 92.65 | 37.53 | 97.09 | - | - | - | 0.38 | 0.02 |

* Calculate heterozygosity using genomescope2.

** Calculate heterozygosity using genotype of SNPs.

| **Table S14 Summary of sample sizes by collection site and group.** | | | | | |
| --- | --- | --- | --- | --- | --- |
| Location | Group | Number | Location | Group | Number |
| South Asia | G1 | 0 | Middle Africa | G1 | 0 |
| South Asia | G2 | 0 | Middle Africa | G2 | 0 |
| South Asia | G3 | 10 | Middle Africa | G3 | 6 |
| South Asia | G4 | 11 | Middle Africa | G4 | 2 |
| South-Est Asia | G1 | 0 | East Asia | G1 | 210 |
| South-Est Asia | G2 | 2 | East Asia | G2 | 234 |
| South-Est Asia | G3 | 18 | East Asia | G3 | 296 |
| South-Est Asia | G4 | 5 | East Asia | G4 | 3 |
| South Africa | G1 | 0 | North America | G1 | 0 |
| South Africa | G2 | 0 | North America | G2 | 4 |
| South Africa | G3 | 2 | North America | G3 | 11 |
| South Africa | G4 | 0 | North America | G4 | 0 |
| East Africa | G1 | 1 | South Europe | G1 | 0 |
| East Africa | G2 | 1 | South Europe | G2 | 0 |
| East Africa | G3 | 7 | South Europe | G3 | 7 |
| East Africa | G4 | 49 | South Europe | G4 | 0 |
| Central America | G1 | 0 | South America | G1 | 2 |
| Central America | G2 | 2 | South America | G2 | 1 |
| Central America | G3 | 8 | South America | G3 | 3 |
| Central America | G4 | 1 | South America | G4 | 0 |
| West Africa | G1 | 0 | Caribbean | G1 | 0 |
| West Africa | G2 | 2 | Caribbean | G2 | 0 |
| West Africa | G3 | 3 | Caribbean | G3 | 3 |
| West Africa | G4 | 11 | Caribbean | G4 | 0 |
| West Asia | G1 | 0 | East Europe | G1 | 0 |
| West Asia | G2 | 0 | East Europe | G2 | 0 |
| West Asia | G3 | 8 | East Europe | G3 | 2 |
| West Asia | G4 | 2 | East Europe | G4 | 0 |

| **Table S15 Statistics of gene flow among the eight populations of 927 sesame accessions.** | | | | | | | | |
| --- | --- | --- | --- | --- | --- | --- | --- | --- |
| **Pop1** | **Pop2** | **Pop3** | **Pop4** | **Estimate** | **SE** | **Z_score** | **P_value** | **Significance** |
| Root | North China | South China | South-East Asia | -0.001249132 | 2.67E-04 | -4.685001013 | 2.80E-06 | *** |
| Root | South China | South-East Asia | South Asia | -0.001015945 | 3.05E-04 | -3.335716862 | 8.51E-04 | *** |
| South China | South-East Asia | North China | - | 0.001754448 | 2.03E-04 | 8.637784998 | 5.73E-18 | *** |
| Root | East Africa | West Africa | - | 0.006748865 | 0.00179267 | 3.76469904 | 1.67E-04 | *** |
| Root | South Asia | North China | West Asia | 0.005115482 | 4.35E-04 | 11.75608658 | 6.57E-32 | *** |
| Root | East Africa | West Africa | South Asia | 0.001762638 | 3.18E-04 | 5.540291987 | 3.02E-08 | *** |
| Root | South-East Asia | South Asia | North China | -0.00207977 | 3.50E-04 | -5.93514812 | 2.94E-09 | *** |
| Root | North America | South China | West Asia | 6.09E-04 | 2.90E-04 | 2.101767949 | 0.035573608 | * |
| Root | West Asia | South China | South Asia | -8.76E-04 | 3.89E-04 | -2.253168718 | 0.024248513 | * |
| Root | South Asia | North China | South-East Asia | 0.003656808 | 3.65E-04 | 10.02584937 | 1.17E-23 | *** |
| Root | North China | South China | South-East Asia | -0.001249132 | 2.67E-04 | -4.685001013 | 2.80E-06 | *** |
| Root | East Africa | West Africa | South Asia | 0.001762638 | 3.18E-04 | 5.540291987 | 3.02E-08 | *** |
| Root | South Asia | South China | North China | -0.00407918 | 3.91E-04 | -10.41968317 | 2.02E-25 | *** |
| Root | South-East Asia | South China | North China | -0.003518088 | 3.90E-04 | -9.017948515 | 1.92E-19 | *** |
| Root | East Africa | South Asia | West Asia | -0.001168653 | 2.93E-04 | -3.991270527 | 6.57E-05 | *** |
| Root | East Africa | West Africa | South Asia | 0.001762638 | 3.18E-04 | 5.540291987 | 3.02E-08 | *** |
| Root | South-East Asia | South Asia | South China | 0.001438318 | 3.32E-04 | 4.337642453 | 1.44E-05 | *** |
| Root | South Asia | South-East Asia | East Africa | -0.001532393 | 3.29E-04 | -4.66011731 | 3.16E-06 | *** |
| South China | North China | South-East Asia | Root | 0.003518088 | 3.90E-04 | 9.017948515 | 1.92E-19 | *** |
| South-East Asia | North China | South China | Root | 0.002268956 | 3.94E-04 | 5.753500122 | 8.74E-09 | *** |
| Root | West Asia | South Asia | South-East Asia | -1.61E-04 | 2.97E-04 | -0.542844801 | 0.587236664 | ns |
| Root | West Asia | East Africa | South Asia | 0.00415972 | 3.71E-04 | 11.21237683 | 3.55E-29 | *** |
| Root | North America | South China | South-East Asia | -6.20E-04 | 2.30E-04 | -2.697604848 | 0.006984029 | ** |
| Root | North America | South China | West Asia | 6.09E-04 | 2.90E-04 | 2.101767949 | 0.035573608 | * |
| Root | East Africa | South Asia | West Asia | -0.001168653 | 2.93E-04 | -3.991270527 | 6.57E-05 | *** |
| East Africa | West Asia | West Africa | South Asia | -0.002876646 | 3.69E-04 | -7.795936688 | 6.39E-15 | *** |
| East Africa | South Asia | West Africa | South-East Asia | -1.63E-05 | 1.74E-04 | -0.094014817 | 0.925097386 | ns |
| West Asia | South Asia | East Africa | South-East Asia | -0.002466258 | 2.31E-04 | -10.69903148 | 1.03E-26 | *** |
| Root | East Africa | South Asia | South China | -9.30E-04 | 2.95E-04 | -3.155343547 | 0.001603092 | *** |
| West Asia | North China | East Africa | South Asia | -9.70E-04 | 2.40E-04 | -4.03559804 | 5.45E-05 | *** |
| South-East Asia | South China | South Asia | Root | -4.22E-04 | 2.50E-04 | -1.686295902 | 0.09173882 | ns |
| South Asia | South China | South-East Asia | Root | -0.001438318 | 3.32E-04 | -4.337642453 | 1.44E-05 | *** |
| Root | South China | South Asia | South-East Asia | 0.001015945 | 3.05E-04 | 3.335716862 | 8.51E-04 | *** |
| South China | South Asia | South-East Asia | - | 0.002581207 | 2.33E-04 | 11.08047937 | 1.56E-28 | *** |
| Root: six wild sesame accessions  ns: non-significant  *: <0.05  **:<0.01  ***<0.001 | | | | | | | | |

| **Table S16 Statistics of DF trait of 927 sesame germplasm accessions in two positions for four years .** | | | | | | | | | | | | | | | | | | | |
| --- | --- | --- | --- | --- | --- | --- | --- | --- | --- | --- | --- | --- | --- | --- | --- | --- | --- | --- | --- |
| **Sample no.** | **Geography** | **Sanya position** | | | | **Yuanyang position** | | | | **Sample no.** | **Geography** | **Sanya position** | | | | **Yuanyang position** | | | |
|  |  | **2018** | **2019** | **2021** | **2022** | **2019** | **2020** | **2021** | **2022** |  |  | **2018** | **2019** | **2021** | **2022** | **2019** | **2020** | **2021** | **2022** |
| S01 | East Asia | 31 | 31 | NA | 35 | 28 | 31 | 40 | 51 | S465 | East Asia | 28 | 32 | 34 | 35 | 47 | 36 | 33 | 39 |
| S02 | East Asia | 29 | 31 | NA | 35 | 28 | 45 | 40 | 41 | S466 | East Asia | 30 | 33 | 34 | 34 | 41 | 36 | 35 | 32 |
| S03 | East Asia | 29 | 33 | NA | 34 | 28 | 55 | 44 | 41 | S467 | East Asia | 33 | 31 | 34 | 35 | 44 | 44 | 40 | 32 |
| S04 | East Asia | 29 | 32 | NA | 36 | 28 | 47 | 48 | 46 | S468 | East Asia | NA | 31 | 34 | 37 | 45 | 47 | 39 | 48 |
| S05 | East Asia | 29 | 31 | NA | 35 | 34 | 45 | 39 | 42 | S469 | East Asia | 28 | 30 | 35 | 40 | 35 | 30 | 33 | 50 |
| S06 | East Asia | 28 | 34 | NA | 33 | 36 | 45 | 44 | 44 | S470 | East Asia | 28 | 31 | 32 | 34 | 36 | 42 | 35 | 35 |
| S07 | East Asia | 28 | 35 | NA | 35 | 28 | 46 | 42 | 40 | S471 | East Asia | 29 | 30 | 33 | 38 | 36 | 53 | 37 | 38 |
| S08 | East Asia | 28 | 35 | NA | 35 | 32 | 45 | 37 | 42 | S472 | East Asia | 33 | 30 | 34 | 34 | 32 | 47 | 38 | 49 |
| S09 | East Asia | 29 | 31 | NA | 34 | 39 | 46 | 40 | 42 | S473 | East Asia | 33 | 30 | 33 | 34 | 32 | 47 | 33 | 43 |
| S10 | East Asia | 29 | 31 | NA | 35 | 35 | 47 | 39 | 42 | S474 | East Asia | 30 | 30 | 33 | 34 | 35 | 31 | 38 | 43 |
| S11 | East Asia | 30 | 31 | NA | 35 | 40 | 51 | 39 | 46 | S475 | East Asia | 30 | 31 | 32 | 32 | 32 | 51 | 38 | 43 |
| S12 | East Asia | 27 | 30 | NA | 35 | 39 | 51 | 41 | 42 | S476 | East Asia | 30 | 30 | 33 | 34 | 35 | 42 | 37 | 46 |
| S13 | East Asia | 27 | 32 | NA | 35 | 39 | 47 | 45 | 45 | S477 | East Asia | 28 | 31 | 32 | 34 | 35 | 48 | 37 | 37 |
| S14 | East Asia | 29 | 32 | NA | 43 | 46 | 54 | 49 | 43 | S478 | East Asia | 35 | 32 | 31 | 34 | 32 | 47 | 38 | 38 |
| S15 | East Asia | 30 | 32 | NA | 35 | 29 | 52 | 41 | 50 | S479 | East Asia | 30 | 31 | 34 | 35 | 36 | 47 | 38 | 51 |
| S16 | East Asia | 27 | 31 | NA | 38 | 41 | 47 | 41 | 46 | S480 | East Asia | 33 | 30 | 32 | 35 | 32 | 48 | 37 | 43 |
| S17 | East Asia | 27 | 30 | NA | 35 | 46 | 47 | 40 | 43 | S481 | East Asia | 33 | 30 | 42 | 35 | 34 | 44 | 36 | 44 |
| S18 | East Asia | 29 | 29 | NA | 34 | 46 | 45 | 41 | 42 | S482 | East Asia | 30 | 30 | 33 | 34 | 38 | 30 | 31 | 42 |
| S19 | East Asia | 29 | 33 | NA | 35 | 29 | 56 | 42 | 40 | S483 | East Asia | 32 | 30 | 34 | 36 | 34 | 47 | 39 | 35 |
| S20 | East Asia | 28 | 32 | NA | 33 | 31 | 51 | 42 | 50 | S484 | East Asia | 34 | 32 | 54 | 35 | 30 | 44 | 45 | 43 |
| S21 | East Asia | 28 | 38 | NA | 33 | 44 | 57 | 40 | 46 | S485 | East Asia | 33 | 29 | 52 | 35 | 36 | 42 | 37 | 38 |
| S22 | East Asia | 29 | 32 | NA | 35 | 38 | 31 | 41 | 49 | S486 | East Asia | 33 | 32 | 35 | 35 | 55 | 44 | 40 | 38 |
| S23 | East Asia | 29 | 30 | NA | 35 | 41 | 57 | 44 | 45 | S487 | East Asia | 34 | 32 | 34 | 37 | 36 | 31 | 40 | 40 |
| S24 | East Asia | 28 | 32 | NA | 32 | 38 | 31 | 40 | 51 | S488 | East Asia | 33 | 30 | 32 | 37 | 38 | 31 | 38 | 44 |
| S25 | East Asia | 28 | 32 | NA | 34 | 28 | 31 | 39 | 45 | S489 | East Asia | 30 | 31 | 33 | 35 | 34 | 48 | 38 | 43 |
| S26 | East Asia | 32 | 32 | NA | 45 | 38 | 54 | 44 | 43 | S490 | East Asia | 28 | 33 | 35 | 35 | 39 | 47 | 35 | 42 |
| S27 | East Asia | 32 | 33 | NA | 43 | 40 | 62 | 45 | 49 | S491 | East Asia | 28 | 32 | 34 | 35 | 45 | 44 | 34 | 41 |
| S28 | East Asia | 30 | 34 | NA | 34 | 28 | 52 | 44 | 42 | S492 | East Asia | 31 | 32 | 35 | 34 | 38 | 31 | 42 | 40 |
| S29 | East Asia | 28 | 35 | NA | 35 | 38 | 47 | 42 | 46 | S493 | East Asia | 29 | 32 | 32 | 36 | 33 | 41 | 36 | 43 |
| S30 | East Asia | 29 | 35 | NA | 35 | 38 | 45 | 41 | 42 | S494 | East Asia | 30 | 32 | 34 | 36 | 38 | 40 | 36 | 35 |
| S31 | East Asia | 31 | 33 | NA | 35 | 43 | 55 | 46 | 39 | S495 | East Asia | 27 | 33 | 34 | 34 | 28 | 38 | 34 | 35 |
| S32 | East Asia | 29 | 30 | NA | 33 | 28 | 47 | 40 | 50 | S496 | East Asia | 29 | 32 | 34 | 34 | 34 | 43 | 38 | 34 |
| S33 | East Asia | 29 | 32 | NA | 34 | 38 | 47 | 40 | 42 | S497 | East Asia | 31 | 32 | 33 | 35 | 35 | 44 | 38 | 38 |
| S34 | East Asia | 34 | 31 | NA | 36 | 46 | 57 | 42 | 40 | S498 | East Asia | 30 | 32 | 34 | 34 | 34 | 43 | 37 | 40 |
| S35 | East Asia | 30 | 31 | NA | 34 | 40 | 54 | 42 | 51 | S499 | East Asia | 28 | 32 | 32 | 35 | 35 | 47 | 40 | 38 |
| S36 | East Asia | 28 | 30 | NA | 37 | 36 | 45 | 38 | 48 | S500 | East Asia | 32 | 29 | 33 | 34 | 38 | 48 | 39 | 43 |
| S37 | East Asia | 27 | 30 | NA | 37 | 40 | 47 | 38 | 38 | S501 | East Asia | 28 | 33 | 32 | 35 | 35 | 41 | 37 | 38 |
| S38 | East Asia | 31 | 35 | NA | 37 | 40 | 47 | 39 | 41 | S502 | East Asia | 29 | 32 | 32 | 35 | 33 | 41 | 34 | 35 |
| S39 | East Asia | 27 | 32 | NA | 35 | 42 | 31 | 42 | 40 | S503 | East Asia | 28 | 31 | 31 | 35 | 29 | 53 | 44 | 38 |
| S40 | East Asia | 29 | 32 | NA | 35 | 40 | 54 | 40 | 42 | S504 | East Asia | 28 | 31 | 34 | 34 | 29 | 30 | 33 | 34 |
| S41 | East Asia | 29 | 34 | NA | 36 | 28 | 54 | 43 | 50 | S505 | East Asia | 29 | 31 | 31 | 39 | 29 | 30 | 32 | 33 |
| S42 | East Asia | 29 | 35 | NA | 32 | 28 | 47 | 40 | 48 | S506 | East Asia | 28 | 31 | NA | 35 | 35 | 40 | 38 | 34 |
| S43 | East Asia | 30 | 33 | NA | 36 | 40 | 50 | 47 | 50 | S507 | East Asia | 30 | 29 | NA | 36 | 33 | 30 | 40 | 34 |
| S44 | East Asia | 30 | 34 | NA | 37 | 39 | 63 | 54 | 44 | S508 | East Asia | 28 | 32 | NA | 35 | 33 | 43 | 38 | 35 |
| S45 | East Asia | 30 | 33 | NA | 35 | 44 | 53 | 40 | 45 | S509 | East Asia | 29 | 30 | NA | 35 | 44 | 31 | 43 | 38 |
| S46 | East Asia | 31 | 29 | NA | 35 | 39 | 62 | 41 | 48 | S510 | East Asia | 25 | 32 | NA | 35 | 32 | 31 | 43 | 43 |
| S47 | East Asia | 31 | 32 | NA | 37 | 39 | 31 | 44 | 57 | S511 | East Asia | 27 | 32 | NA | 34 | 32 | 47 | 41 | 45 |
| S48 | East Asia | 31 | 34 | NA | 38 | 44 | 54 | 52 | 43 | S512 | East Asia | 29 | 30 | NA | 35 | 46 | 47 | 44 | 42 |
| S49 | East Asia | 31 | 30 | NA | 35 | 40 | 50 | 50 | 49 | S513 | East Asia | 27 | 28 | NA | 37 | 29 | 44 | 41 | 42 |
| S50 | East Asia | 30 | 31 | NA | 35 | 44 | 57 | 42 | 45 | S514 | East Asia | 29 | 33 | NA | 34 | 29 | 31 | 41 | 38 |
| S51 | East Asia | 31 | 32 | NA | 37 | 45 | 55 | 47 | 51 | S515 | East Asia | NA | 30 | NA | 34 | 32 | 51 | 45 | 53 |
| S52 | East Asia | 31 | 34 | NA | 37 | 40 | 51 | 44 | 50 | S516 | East Asia | 36 | 35 | NA | 35 | 35 | 31 | 44 | 46 |
| S53 | East Asia | 31 | 30 | NA | 39 | 44 | 63 | 46 | 46 | S517 | East Asia | 29 | 32 | NA | 36 | 32 | 32 | 43 | 47 |
| S54 | East Asia | 32 | 32 | NA | 38 | 29 | 31 | 41 | 57 | S518 | East Asia | 37 | 35 | NA | 37 | 29 | 86 | 38 | 45 |
| S55 | East Asia | 29 | 32 | NA | 38 | 45 | 51 | 45 | 45 | S519 | East Asia | 37 | 34 | NA | 35 | 35 | 51 | 39 | 80 |
| S56 | East Asia | 31 | 33 | NA | 35 | 45 | 54 | 54 | 45 | S520 | East Asia | 29 | 34 | NA | 34 | 40 | 46 | 39 | 45 |
| S57 | East Asia | 28 | 32 | NA | 49 | 41 | 51 | 40 | 48 | S521 | East Asia | 32 | 34 | NA | 37 | 40 | 48 | 39 | 40 |
| S58 | East Asia | 28 | 34 | NA | 38 | 41 | 51 | 40 | 45 | S522 | East Asia | 28 | 32 | NA | 37 | 40 | 47 | 39 | 43 |
| S59 | East Asia | 29 | 34 | NA | 40 | 45 | 31 | 47 | 45 | S523 | East Asia | 37 | 33 | NA | 34 | 35 | 47 | 39 | 42 |
| S60 | East Asia | 29 | 34 | NA | 33 | 38 | 46 | 45 | 43 | S524 | East Asia | 34 | 30 | NA | 35 | 39 | 45 | 42 | 42 |
| S61 | East Asia | 29 | 33 | NA | 39 | 45 | 31 | 40 | 40 | S525 | East Asia | 34 | 32 | NA | 37 | 39 | 47 | 38 | 39 |
| S62 | East Asia | 30 | 30 | NA | 37 | 43 | 62 | 53 | 51 | S526 | East Asia | 33 | 32 | NA | 35 | 46 | 47 | 44 | 42 |
| S63 | East Asia | 26 | 32 | NA | 33 | 32 | 55 | 37 | 56 | S527 | East Asia | 38 | 31 | NA | 34 | 41 | 51 | 44 | 42 |
| S64 | East Asia | 29 | 29 | NA | 35 | 38 | 58 | 40 | 42 | S528 | East Asia | 33 | 32 | NA | 35 | 47 | 51 | 54 | 45 |
| S65 | East Asia | 28 | 29 | NA | 34 | 32 | 55 | 38 | 52 | S529 | East Asia | 30 | 32 | NA | 37 | 42 | 51 | 47 | 47 |
| S66 | East Asia | 30 | 29 | NA | 33 | 29 | 44 | 35 | 49 | S530 | East Asia | 37 | 32 | NA | 37 | 43 | 51 | 44 | 46 |
| S67 | East Asia | 33 | 33 | NA | 35 | 38 | 57 | 49 | 41 | S531 | East Asia | 36 | 31 | NA | 35 | 39 | 53 | 52 | 46 |
| S68 | East Asia | 28 | 31 | NA | 40 | 32 | 54 | 44 | 47 | S532 | East Asia | 34 | 31 | NA | 37 | 44 | 51 | 50 | 49 |
| S69 | East Asia | 28 | 31 | NA | 36 | 35 | 47 | 47 | 49 | S533 | East Asia | 36 | 32 | NA | 35 | 37 | 54 | 45 | 46 |
| S70 | East Asia | 28 | 32 | NA | 33 | 35 | 50 | 38 | 49 | S534 | East Asia | 28 | 33 | NA | 35 | 40 | 55 | 42 | 49 |
| S71 | East Asia | 30 | 33 | NA | 34 | 45 | 55 | 53 | 44 | S535 | East Asia | 29 | 32 | NA | 35 | 40 | 54 | 53 | 49 |
| S72 | East Asia | 30 | 31 | NA | 35 | 41 | 55 | 43 | 50 | S536 | East Asia | 27 | 34 | NA | 35 | 40 | 47 | 52 | 48 |
| S73 | East Asia | 30 | 36 | NA | 34 | 53 | 63 | 47 | 49 | S537 | East Asia | 32 | 33 | NA | 37 | 45 | 31 | 43 | NA |
| S74 | East Asia | 27 | 30 | NA | 36 | 66 | 51 | 44 | 57 | S538 | East Asia | 27 | 32 | NA | 35 | 44 | 52 | 44 | 44 |
| S75 | East Asia | 28 | 30 | NA | 35 | 39 | 58 | 42 | 46 | S539 | East Asia | 28 | 32 | NA | 37 | 39 | 55 | 43 | 47 |
| S76 | East Asia | 25 | 31 | NA | 36 | 40 | 58 | 47 | 53 | S540 | East Asia | 32 | 32 | NA | 35 | 59 | 55 | 47 | 50 |
| S77 | East Asia | 27 | 33 | NA | 35 | 41 | 63 | 52 | 53 | S541 | East Asia | 33 | 33 | NA | 35 | 29 | 52 | 47 | 45 |
| S78 | East Asia | 30 | 32 | NA | 36 | 40 | 63 | 44 | 49 | S542 | East Asia | 28 | 34 | NA | 35 | 38 | 44 | 44 | 43 |
| S79 | East Asia | 29 | 32 | NA | 35 | 40 | 57 | 47 | 53 | S543 | East Asia | 29 | 31 | NA | 34 | 45 | 52 | 53 | 38 |
| S80 | East Asia | 29 | 34 | NA | 34 | 28 | 54 | 39 | 51 | S544 | East Asia | 30 | 30 | NA | 35 | 39 | 63 | 53 | 46 |
| S81 | South Asia | 29 | 30 | 31 | 34 | 42 | 64 | 56 | 58 | S545 | East Asia | 33 | 30 | NA | 35 | 43 | 55 | 47 | 57 |
| S82 | South Asia | 29 | 31 | 32 | 34 | 39 | 63 | 52 | 57 | S546 | East Asia | 32 | 29 | NA | 34 | 42 | 55 | 52 | 50 |
| S83 | South Asia | 30 | 31 | 35 | 38 | 40 | 64 | 55 | 58 | S547 | East Asia | 27 | 30 | NA | 34 | 40 | 44 | 41 | 41 |
| S84 | South Asia | 30 | 32 | 34 | 34 | 40 | NA | 44 | 50 | S548 | East Asia | 33 | 27 | NA | 35 | 39 | 50 | 41 | 39 |
| S85 | South Asia | 28 | 29 | 30 | 33 | 42 | 51 | 47 | 44 | S549 | East Asia | 29 | 32 | NA | 33 | 43 | 47 | 40 | 49 |
| S86 | South Africa | 32 | 31 | 35 | 36 | 52 | 62 | 53 | 57 | S550 | East Asia | 29 | 27 | NA | 36 | 42 | 32 | 42 | 42 |
| S87 | East Africa | 29 | 29 | 34 | 34 | 40 | 51 | 44 | 46 | S551 | East Asia | 28 | 29 | NA | 40 | 42 | 51 | 40 | 54 |
| S88 | East Africa | 41 | 44 | 45 | 54 | 94 | NA | 80 | 120 | S552 | East Asia | 29 | 29 | NA | 33 | 43 | 54 | 42 | 45 |
| S89 | East Africa | 36 | 33 | 34 | 35 | 30 | 78 | 59 | 72 | S553 | East Asia | 30 | 33 | NA | 34 | 43 | 47 | 39 | 45 |
| S90 | East Africa | 30 | 29 | 30 | 31 | 66 | 82 | 75 | 76 | S554 | East Asia | 28 | 29 | NA | 36 | 36 | 48 | 43 | 42 |
| S91 | East Africa | 28 | 31 | 30 | 34 | 53 | 91 | 54 | 94 | S555 | East Asia | 29 | 29 | NA | 34 | 33 | 51 | 42 | 43 |
| S92 | East Africa | 28 | 31 | 31 | 33 | 35 | 53 | 40 | 47 | S556 | East Asia | 29 | 32 | NA | 34 | 45 | 54 | 48 | 45 |
| S93 | East Africa | 34 | 34 | 34 | 35 | 91 | 120 | 120 | 120 | S557 | East Asia | 31 | 29 | NA | 35 | 51 | 58 | 50 | 48 |
| S94 | South-East Asia | 29 | 31 | 34 | 34 | 67 | 57 | 68 | 49 | S558 | East Asia | 29 | 29 | NA | 34 | 45 | 54 | 51 | 52 |
| S95 | South-East Asia | 28 | 28 | 32 | 35 | 60 | 48 | 51 | 42 | S559 | East Asia | 29 | 31 | NA | 34 | 53 | 55 | 50 | 48 |
| S96 | South-East Asia | 33 | 31 | 34 | 36 | 42 | 64 | 52 | 58 | S560 | East Asia | 35 | 31 | NA | 35 | 43 | 56 | 56 | 49 |
| S97 | South-East Asia | 41 | 34 | 35 | 37 | 38 | 65 | 52 | 58 | S561 | East Asia | 26 | 30 | NA | 35 | 51 | 57 | 53 | 50 |
| S98 | South-East Asia | 28 | 29 | 31 | 36 | 29 | 53 | 44 | 46 | S562 | East Asia | 34 | 32 | NA | 35 | 52 | 55 | 44 | 51 |
| S99 | South-East Asia | 34 | 35 | 35 | 37 | 44 | 71 | 72 | 65 | S563 | East Asia | 30 | 29 | NA | 35 | 46 | 54 | 52 | 50 |
| S100 | South-East Asia | 28 | 30 | 32 | 35 | 41 | 65 | 47 | 59 | S564 | East Asia | 33 | 29 | NA | 35 | 45 | 60 | 55 | 48 |
| S101 | South-East Asia | 31 | 32 | 34 | 40 | 49 | 60 | 56 | 63 | S565 | East Asia | 32 | 29 | NA | 38 | 53 | 47 | 44 | 43 |
| S102 | Central America | 30 | 31 | 34 | 38 | 36 | 82 | 66 | 76 | S566 | East Asia | 29 | 30 | NA | 36 | 45 | 66 | 41 | 41 |
| S103 | Central America | 33 | 32 | 34 | 37 | 83 | 51 | 47 | 46 | S567 | East Asia | 34 | 34 | NA | 34 | NA | 44 | 38 | 61 |
| S104 | Central America | 31 | 32 | 34 | 38 | 39 | 57 | 52 | 51 | S568 | East Asia | 33 | 30 | NA | 34 | 40 | 54 | 44 | 39 |
| S105 | Central America | 30 | 32 | 34 | 33 | 52 | 57 | 53 | 51 | S569 | East Asia | 31 | 32 | NA | 35 | 52 | 48 | 39 | 48 |
| S106 | Central America | 31 | 32 | 34 | 34 | 28 | 55 | 47 | 50 | S570 | East Asia | 29 | 32 | NA | 36 | 45 | 48 | 38 | 43 |
| S107 | Central America | 28 | 31 | 33 | 33 | 46 | 54 | 43 | 47 | S571 | East Asia | 33 | 31 | NA | 35 | 45 | 57 | 44 | 42 |
| S108 | West Africa | 31 | 31 | 30 | 32 | 39 | 118 | 120 | 51 | S572 | East Asia | 33 | 32 | NA | 37 | 45 | 57 | 48 | 51 |
| S109 | East Africa | 39 | 33 | 37 | 39 | 43 | 92 | 120 | 104 | S573 | East Asia | 34 | 31 | NA | 35 | 51 | 47 | 47 | 51 |
| S110 | East Africa | 34 | 34 | 34 | 39 | 42 | 120 | 120 | 120 | S574 | East Asia | 29 | 30 | NA | 35 | 49 | 57 | 52 | 54 |
| S111 | East Africa | 35 | 33 | 37 | 42 | 35 | 107 | 120 | 102 | S575 | East Asia | 29 | 30 | NA | 35 | 38 | 51 | 41 | 50 |
| S112 | East Africa | 34 | 34 | 38 | 39 | 94 | 120 | 120 | 120 | S576 | East Asia | 33 | 31 | NA | 35 | 36 | 55 | 48 | 46 |
| S113 | East Africa | 41 | 40 | 41 | 44 | 75 | 117 | 120 | 111 | S577 | East Asia | 29 | 31 | NA | 35 | 33 | 47 | 44 | 51 |
| S114 | East Africa | 35 | 35 | 34 | 37 | 37 | 122 | 120 | 116 | S578 | East Asia | 30 | 32 | NA | 33 | 33 | 53 | 47 | 43 |
| S115 | East Africa | 35 | 34 | 38 | 41 | 92 | 126 | 120 | 120 | S579 | East Asia | 35 | 32 | NA | 33 | 45 | 50 | 53 | 47 |
| S116 | East Africa | NA | 42 | 42 | 48 | 29 | 123 | 120 | 117 | S580 | East Asia | NA | 31 | NA | 35 | 37 | 55 | 44 | 44 |
| S117 | West Asia | 28 | 30 | 32 | 34 | 76 | 54 | 39 | 49 | S581 | East Asia | 33 | 31 | NA | 32 | 36 | 71 | 53 | 49 |
| S118 | West Asia | 29 | 30 | 33 | 33 | 48 | 32 | 41 | 53 | S582 | East Asia | 33 | 29 | NA | 33 | 42 | 57 | 51 | NA |
| S119 | West Asia | 35 | 34 | 41 | 40 | 42 | 67 | 69 | 61 | S583 | East Asia | 29 | 32 | NA | 34 | 53 | 45 | 45 | 61 |
| S120 | West Asia | 29 | 30 | 34 | 34 | 38 | 51 | 42 | 46 | S584 | East Asia | 31 | 30 | NA | 33 | 47 | 53 | 47 | 51 |
| S121 | East Africa | 42 | 38 | 41 | 43 | 45 | 107 | 120 | 101 | S585 | East Asia | 33 | 28 | NA | 34 | 49 | 55 | 54 | 39 |
| S122 | West Africa | 30 | 32 | 34 | 34 | 102 | 51 | 47 | 46 | S586 | East Asia | 33 | 31 | NA | 34 | 54 | 67 | 42 | 62 |
| S123 | East Asia | 30 | 29 | 33 | 36 | 94 | 47 | 42 | 42 | S587 | East Asia | 31 | 31 | NA | 35 | 47 | 58 | 44 | 54 |
| S124 | East Asia | 28 | 29 | 32 | 34 | 80 | 44 | 44 | 39 | S588 | East Asia | 31 | 29 | NA | 37 | 53 | 55 | 43 | 53 |
| S125 | East Asia | 29 | 32 | 33 | 34 | 45 | 64 | 48 | 59 | S589 | East Asia | 30 | 33 | NA | 33 | 36 | 32 | 39 | 49 |
| S126 | East Asia | 27 | 27 | 30 | 33 | 53 | 58 | 42 | 52 | S590 | East Asia | 36 | 30 | NA | 32 | 49 | 51 | 45 | 54 |
| S127 | East Africa | 33 | 32 | 34 | 38 | 92 | 61 | 44 | 55 | S591 | East Asia | 30 | 30 | NA | 33 | 36 | 51 | 44 | 46 |
| S128 | East Africa | 34 | 35 | 37 | 37 | 97 | NA | 120 | NA | S592 | East Asia | 30 | 32 | NA | 32 | 47 | 51 | 44 | 46 |
| S129 | Centra Africa | 27 | 30 | 30 | 34 | 45 | 44 | 38 | 40 | S593 | East Asia | 32 | 30 | NA | 33 | 45 | 55 | 51 | 46 |
| S130 | Southern Europe | 31 | 32 | 34 | 35 | 48 | 64 | 44 | 59 | S594 | East Asia | 29 | 30 | NA | 35 | 42 | 47 | 51 | 49 |
| S131 | Southern Europe | 31 | 32 | 32 | 34 | 34 | 50 | 44 | 46 | S595 | East Asia | 31 | 32 | NA | 32 | 55 | 53 | 41 | 50 |
| S132 | Southern Europe | 30 | 30 | 32 | 34 | 39 | 47 | 40 | 42 | S596 | East Asia | 30 | 31 | NA | 34 | 50 | 47 | 38 | 43 |
| S133 | Southern Europe | 33 | 32 | 34 | 34 | 35 | 53 | 47 | 46 | S597 | East Asia | 31 | 30 | NA | 33 | 46 | 47 | 40 | 41 |
| S134 | South-East Asia | 33 | 32 | 34 | 37 | 50 | 52 | 46 | 45 | S598 | East Asia | 32 | 27 | NA | 35 | 53 | 57 | 48 | 42 |
| S135 | South-East Asia | 32 | 32 | 34 | 34 | 45 | 51 | 45 | 47 | S599 | East Asia | 30 | 30 | NA | 33 | 44 | 53 | 47 | 51 |
| S136 | South-East Asia | 33 | 31 | 34 | 34 | 43 | 57 | 46 | 52 | S600 | East Asia | NA | 30 | NA | 34 | 39 | 98 | 81 | 45 |
| S137 | South-East Asia | 30 | 31 | 33 | 34 | 37 | 53 | 52 | 47 | S601 | East Asia | 31 | 29 | NA | 35 | 36 | 71 | 55 | 47 |
| S138 | Caribbean | 29 | 30 | 33 | 33 | 43 | 47 | 46 | 42 | S602 | East Asia | 35 | 29 | NA | 38 | 44 | 53 | 41 | 66 |
| S139 | West Africa | 34 | 33 | 37 | 35 | 80 | 120 | 120 | 120 | S603 | East Asia | 33 | 30 | NA | 34 | 43 | 62 | 55 | 92 |
| S140 | East Africa | 34 | NA | 35 | 37 | NA | 120 | 120 | 120 | S604 | East Asia | 35 | 32 | NA | 35 | 47 | 57 | 44 | 57 |
| S141 | West Africa | 36 | 35 | 37 | 42 | 36 | 106 | 120 | 99 | S605 | East Asia | 33 | 30 | NA | 34 | 39 | 57 | 52 | 39 |
| S142 | West Africa | NA | 45 | 34 | 35 | 39 | 120 | 120 | 120 | S606 | East Asia | 30 | 30 | NA | 33 | 37 | 55 | 47 | 42 |
| S143 | West Asia | 33 | 30 | 34 | 34 | 40 | 51 | 51 | 46 | S607 | East Asia | 32 | 33 | 35 | 39 | 39 | 57 | 47 | 45 |
| S144 | South Asia | 38 | 37 | 37 | 40 | 90 | 31 | 120 | 42 | S608 | East Asia | 29 | 36 | 34 | 35 | 40 | 55 | 50 | 49 |
| S145 | West Africa | 35 | 34 | 37 | 37 | 29 | 91 | 120 | 94 | S609 | East Asia | 28 | 32 | 32 | 33 | 47 | 44 | 37 | 42 |
| S146 | West Africa | 29 | 30 | 34 | 37 | 40 | 48 | 48 | 43 | S610 | East Asia | 30 | 34 | 32 | 34 | 43 | 51 | 39 | 45 |
| S147 | East Africa | 32 | 32 | 36 | 35 | 62 | 57 | 48 | 52 | S611 | East Asia | 30 | 29 | 32 | 35 | 40 | 51 | 48 | 51 |
| S148 | West Asia | 31 | 32 | 37 | 38 | 41 | 62 | 48 | 57 | S612 | East Asia | 29 | 32 | 32 | 34 | 37 | 31 | 45 | 45 |
| S149 | South Asia | 29 | 31 | 34 | 34 | 39 | 51 | 47 | 44 | S613 | East Asia | 34 | 33 | 35 | 33 | 38 | 31 | 45 | 45 |
| S150 | Central America | 25 | 27 | 31 | 33 | 43 | 48 | 42 | 42 | S614 | East Asia | 28 | 30 | 34 | 37 | 42 | 52 | 38 | 35 |
| S151 | Central America | 30 | 30 | 34 | 34 | 40 | 48 | 41 | 42 | S615 | West Africa | 31 | 30 | 34 | 34 | 52 | 54 | 47 | 49 |
| S152 | East Asia | 28 | 33 | 34 | 38 | 120 | 63 | 52 | 51 | S616 | East Asia | 31 | 31 | 34 | 35 | 41 | 46 | 38 | 37 |
| S153 | East Asia | 29 | 42 | 35 | 34 | 125 | 32 | 42 | 47 | S617 | East Asia | 28 | 33 | 32 | 34 | 40 | 45 | 48 | 49 |
| S154 | East Asia | 28 | 43 | 38 | 39 | 119 | 55 | 45 | 50 | S618 | East Asia | 28 | 32 | NA | 35 | 43 | 53 | 40 | 45 |
| S155 | East Asia | 30 | 38 | 40 | 37 | 77 | 54 | 46 | 47 | S619 | East Asia | 30 | 31 | NA | 35 | 46 | 47 | 51 | 49 |
| S156 | East Asia | 29 | 36 | 38 | 40 | NA | 55 | 47 | 47 | S620 | East Asia | 31 | 30 | NA | 37 | 39 | 47 | 52 | 42 |
| S157 | East Asia | 27 | 31 | 32 | 32 | 38 | 48 | 44 | 43 | S621 | East Asia | 32 | 32 | NA | 35 | 40 | 51 | 41 | 42 |
| S158 | East Asia | 28 | 34 | 35 | 37 | 43 | 31 | 39 | 58 | S622 | East Asia | 29 | 31 | NA | 35 | 38 | 47 | 42 | 43 |
| S159 | East Asia | 29 | 42 | 41 | 45 | 46 | 54 | 44 | 45 | S623 | East Asia | 30 | 34 | NA | 37 | 28 | 55 | 41 | 43 |
| S160 | East Asia | 29 | 34 | 36 | 38 | 40 | 48 | 41 | 48 | S624 | East Asia | 31 | 30 | NA | 38 | 40 | 47 | 45 | 45 |
| S161 | East Asia | 33 | 35 | 37 | 40 | 40 | 55 | 44 | 41 | S625 | East Asia | 29 | 34 | NA | 43 | 28 | 31 | 41 | 43 |
| S162 | East Asia | 30 | 31 | 32 | 33 | 40 | 48 | 40 | 48 | S626 | East Asia | 28 | 31 | NA | 40 | 28 | 47 | 40 | 44 |
| S163 | East Asia | 27 | 32 | 34 | 35 | 40 | 48 | 40 | 42 | S627 | East Asia | 29 | 31 | NA | 35 | 38 | 46 | 41 | 41 |
| S164 | East Asia | 28 | 37 | 37 | 42 | 38 | 48 | 40 | 42 | S628 | East Asia | 29 | 30 | NA | 33 | 38 | 51 | 42 | 40 |
| S165 | East Asia | 28 | 31 | 30 | 32 | 41 | 48 | 40 | 42 | S629 | East Asia | 30 | 30 | NA | 35 | 48 | 54 | 41 | 47 |
| S166 | East Asia | 31 | 41 | 42 | 48 | 39 | 51 | 40 | 42 | S630 | East Asia | 30 | 30 | NA | 35 | 46 | 47 | 43 | 48 |
| S167 | East Asia | 32 | 34 | 41 | 39 | 46 | 51 | 46 | 44 | S631 | East Asia | 28 | 37 | NA | 38 | 39 | 48 | 42 | 43 |
| S168 | East Asia | 31 | 34 | 33 | 33 | 41 | 51 | 44 | 51 | S632 | East Asia | 28 | 33 | NA | 37 | 28 | 51 | 40 | 47 |
| S169 | East Asia | 33 | 35 | 31 | 37 | 40 | 54 | 42 | 45 | S633 | East Asia | 28 | 32 | NA | 45 | 36 | 31 | 39 | 44 |
| S170 | East Asia | 33 | 29 | 33 | 40 | 44 | 55 | 43 | 47 | S634 | East Asia | 29 | 32 | NA | 35 | 35 | 47 | 41 | 56 |
| S171 | East Asia | 29 | 34 | 32 | 34 | 45 | 67 | 53 | 49 | S635 | East Asia | 30 | 31 | NA | 33 | 44 | 47 | 44 | 41 |
| S172 | East Asia | 29 | 36 | 33 | 34 | 45 | 57 | 48 | 60 | S636 | East Asia | 27 | 34 | NA | 35 | 38 | 50 | 40 | 42 |
| S173 | East Asia | 28 | 31 | 35 | 37 | 40 | 55 | 46 | 50 | S637 | East Asia | 27 | 30 | NA | 38 | 35 | 46 | 38 | 44 |
| S174 | East Asia | 32 | 44 | 37 | 41 | 45 | 65 | 53 | 48 | S638 | East Asia | 29 | 34 | NA | 41 | 28 | 46 | 39 | 41 |
| S175 | East Asia | 29 | 35 | 34 | 39 | 39 | 51 | 50 | 58 | S639 | East Asia | 35 | 30 | NA | 35 | 41 | 31 | 57 | 57 |
| S176 | East Asia | 29 | 33 | 35 | 34 | 41 | 51 | 44 | 46 | S640 | East Asia | 31 | 28 | NA | 35 | 29 | 62 | 53 | 45 |
| S177 | East Asia | 32 | 32 | 34 | 34 | 38 | 62 | 49 | 46 | S641 | East Asia | 29 | 33 | NA | 35 | 28 | 31 | 41 | 48 |
| S178 | East Asia | 29 | 32 | 32 | 37 | 45 | 63 | 53 | 55 | S642 | East Asia | 32 | 31 | NA | 38 | 38 | 51 | 40 | 43 |
| S179 | East Asia | 29 | 34 | 36 | 37 | 40 | 63 | 50 | 56 | S643 | East Asia | 28 | 32 | NA | 34 | 39 | 55 | 47 | 59 |
| S180 | East Asia | 31 | 32 | 31 | 40 | 45 | 46 | 38 | 44 | S644 | East Africa | NA | 34 | 35 | 35 | 114 | 118 | 120 | NA |
| S181 | East Asia | 28 | 41 | 34 | 33 | 51 | 51 | 44 | 38 | S645 | South-East Asia | 28 | 30 | 31 | 33 | 39 | 51 | 42 | 46 |
| S182 | East Asia | 30 | 32 | 41 | 37 | 45 | 51 | 45 | 44 | S646 | North America | NA | 32 | 35 | 38 | 48 | 54 | 47 | 49 |
| S183 | East Asia | 32 | 36 | 30 | 31 | 41 | 48 | 52 | 46 | S647 | North America | 29 | 31 | 34 | 37 | 46 | 54 | 44 | 49 |
| S184 | East Asia | 30 | 29 | 31 | 32 | 48 | 56 | 53 | 42 | S648 | North America | 30 | 30 | 33 | 34 | 40 | 65 | 66 | 60 |
| S185 | East Asia | 30 | 29 | 31 | 31 | 46 | 61 | 44 | 50 | S649 | North America | 27 | 33 | 32 | 35 | 46 | 51 | 42 | 46 |
| S186 | East Asia | 30 | 39 | 41 | 46 | 45 | 62 | 50 | 47 | S650 | North America | 33 | 33 | 36 | 38 | 42 | 52 | 47 | 47 |
| S187 | East Asia | 30 | 44 | 34 | 34 | 46 | 63 | 52 | 55 | S651 | North America | 30 | 31 | 33 | 34 | 29 | 47 | 47 | 42 |
| S188 | East Asia | 30 | 34 | 33 | 34 | 54 | 63 | 52 | 56 | S652 | North America | 26 | 31 | 31 | 33 | 40 | 47 | 40 | 41 |
| S189 | East Asia | 32 | 32 | 36 | 34 | 49 | 57 | 42 | 56 | S653 | North America | 27 | 29 | 30 | 33 | 40 | 45 | 41 | 41 |
| S190 | East Asia | 30 | 33 | 34 | 41 | 41 | 55 | 42 | 50 | S654 | North America | 33 | 34 | 34 | 38 | 48 | 52 | 47 | 48 |
| S191 | East Asia | 33 | 47 | 36 | 44 | 48 | 66 | 52 | 48 | S655 | North America | 33 | 32 | 35 | 36 | 56 | 55 | 52 | 51 |
| S192 | East Asia | 32 | 49 | 46 | 45 | 45 | 57 | 51 | 60 | S656 | North America | 30 | 30 | 34 | 34 | 39 | 52 | 45 | 46 |
| S193 | East Asia | 29 | 32 | 38 | 38 | 48 | 47 | 38 | 49 | S657 | East Asia | 28 | 29 | 32 | 34 | 45 | 64 | 45 | 58 |
| S194 | East Asia | 30 | 35 | 29 | 32 | 46 | 51 | 40 | 41 | S658 | East Africa | 36 | 38 | 38 | 38 | 76 | 122 | 120 | 116 |
| S195 | East Asia | 33 | 38 | 36 | 37 | 43 | 57 | 39 | 45 | S659 | Southern Europe | 28 | 30 | 31 | 33 | 39 | 47 | 40 | 41 |
| S196 | East Asia | 30 | 35 | 37 | 35 | 38 | 53 | 52 | 45 | S660 | Caribbean | 34 | 34 | 44 | 41 | 37 | 81 | 66 | 76 |
| S197 | East Asia | 30 | 35 | 37 | 40 | 40 | 51 | 41 | 47 | S661 | West Asia | 28 | 28 | 30 | 33 | 37 | 44 | 37 | 39 |
| S198 | East Asia | 33 | 38 | 31 | 33 | 45 | 55 | 40 | 45 | S662 | East Asia | 29 | 31 | 33 | 33 | 45 | 53 | 43 | 39 |
| S199 | East Asia | 30 | 31 | 32 | 32 | 45 | 45 | 41 | 49 | S663 | East Asia | 32 | 30 | 34 | 36 | 40 | 57 | 42 | 47 |
| S200 | East Asia | 29 | 31 | 34 | 37 | 44 | 55 | 51 | 48 | S664 | East Asia | 32 | 31 | 33 | 34 | 38 | 47 | 38 | 41 |
| S201 | East Asia | 30 | 35 | 35 | 37 | 39 | 31 | 42 | 50 | S665 | East Asia | 32 | 32 | 37 | 35 | 35 | 63 | 42 | 57 |
| S202 | East Asia | 29 | 35 | 34 | 33 | 39 | 45 | 39 | 44 | S666 | East Asia | 33 | 32 | 32 | 36 | 37 | 55 | 47 | 48 |
| S203 | East Asia | 30 | 34 | 34 | 33 | 41 | 55 | 41 | 39 | S667 | East Asia | 29 | 32 | 33 | 37 | 45 | 51 | 46 | 55 |
| S204 | East Asia | 32 | 33 | 34 | 34 | 41 | 54 | 38 | 47 | S668 | East Asia | 30 | 32 | 35 | 34 | 40 | 54 | 43 | 49 |
| S205 | East Asia | 33 | 32 | 32 | NA | 48 | 51 | 47 | 45 | S669 | East Asia | 37 | 30 | 34 | 33 | 44 | 47 | 39 | 44 |
| S206 | East Asia | 29 | 32 | 34 | 33 | 43 | 51 | 38 | 53 | S670 | East Asia | 30 | 30 | 32 | 37 | 36 | 52 | 43 | 47 |
| S207 | East Asia | 30 | 34 | 31 | 33 | 41 | 58 | 43 | 45 | S671 | East Asia | 33 | 32 | 34 | 35 | 44 | 46 | 40 | 40 |
| S208 | East Asia | 30 | 32 | 33 | 32 | 43 | 57 | 44 | 53 | S672 | East Asia | 30 | 32 | 35 | 35 | 39 | 42 | 38 | 44 |
| S209 | East Asia | 32 | 30 | 37 | 34 | 40 | 58 | 53 | 52 | S673 | East Asia | 28 | 31 | 35 | 32 | 37 | 41 | 35 | 33 |
| S210 | East Asia | 29 | 34 | 32 | 34 | 35 | 55 | 47 | 52 | S674 | East Asia | 33 | 30 | NA | 32 | 44 | 31 | 40 | 47 |
| S211 | East Asia | 29 | 38 | 34 | 33 | 38 | 54 | 43 | 52 | S675 | East Asia | 32 | 31 | NA | 35 | 44 | 51 | 44 | 47 |
| S212 | East Asia | 30 | 31 | 35 | 34 | 35 | 51 | 41 | 48 | S676 | East Asia | 30 | NA | NA | 33 | 47 | 53 | 40 | 51 |
| S213 | East Asia | 29 | 36 | 49 | NA | 35 | 54 | 42 | 45 | S677 | East Asia | 32 | 31 | NA | 33 | 39 | 56 | 45 | 47 |
| S214 | East Asia | 30 | 52 | 50 | NA | 40 | 51 | 40 | 48 | S678 | East Asia | 30 | 30 | NA | 36 | 39 | 45 | 40 | 50 |
| S215 | East Asia | 28 | 47 | 35 | 39 | 46 | 45 | 42 | 44 | S679 | East Asia | 32 | 34 | NA | 35 | 37 | 47 | 43 | 39 |
| S216 | East Asia | 28 | 34 | 34 | 34 | 35 | 48 | 40 | 40 | S680 | East Asia | 27 | 31 | NA | 34 | 48 | 45 | 37 | 56 |
| S217 | East Asia | 28 | 32 | 41 | NA | 43 | 54 | 44 | 42 | S681 | East Asia | 33 | 33 | NA | 33 | 48 | 45 | 38 | 40 |
| S218 | East Asia | 28 | 40 | 35 | 34 | 41 | 31 | 39 | 48 | S682 | East Asia | 29 | 30 | NA | 35 | 51 | 30 | 30 | 40 |
| S219 | East Asia | 28 | 35 | 31 | 32 | 43 | 45 | 38 | 43 | S683 | East Asia | 29 | 31 | NA | 34 | 43 | 41 | 36 | 33 |
| S220 | East Asia | 28 | 32 | 36 | 38 | 37 | 51 | 130 | 38 | S684 | Eastern Europe | 31 | 29 | 31 | 33 | 38 | 47 | 47 | 42 |
| S221 | East Asia | 30 | 38 | 34 | 47 | 40 | 51 | 44 | 46 | S685 | East Asia | 33 | 31 | NA | 35 | 45 | NA | 69 | 53 |
| S222 | East Asia | 28 | 33 | 42 | 39 | 37 | 48 | 39 | 45 | S686 | East Asia | 33 | 31 | NA | 34 | 36 | NA | 60 | NA |
| S223 | East Asia | 28 | 41 | 35 | 36 | 37 | 51 | 39 | 42 | S687 | East Asia | 33 | 32 | NA | 35 | 39 | 31 | 47 | NA |
| S224 | East Asia | 30 | 34 | 44 | 44 | 45 | 51 | 44 | 45 | S688 | East Asia | 35 | 32 | NA | 33 | 47 | 60 | 57 | 45 |
| S225 | East Asia | 27 | 44 | 31 | 32 | 40 | 51 | 40 | 45 | S689 | East Asia | 30 | 30 | NA | 37 | 45 | 47 | 39 | 35 |
| S226 | East Asia | 31 | 44 | 32 | 32 | 41 | 72 | 41 | 46 | S690 | East Asia | 32 | 30 | NA | 33 | 70 | 31 | 40 | 42 |
| S227 | East Asia | 29 | 44 | 41 | 41 | 45 | 32 | 42 | 66 | S691 | East Asia | 27 | 30 | NA | 37 | 68 | 45 | 37 | 43 |
| S228 | East Asia | 27 | 31 | 34 | 38 | 42 | 51 | 44 | 52 | S692 | East Asia | 33 | 31 | NA | 34 | 48 | 47 | 38 | 38 |
| S229 | East Asia | 28 | 34 | 38 | 37 | 45 | 47 | 38 | 45 | S693 | East Asia | 34 | 33 | NA | 35 | 45 | 63 | 48 | 42 |
| S230 | East Asia | 29 | 35 | 35 | 37 | 36 | 55 | 41 | 41 | S694 | East Asia | 33 | 31 | NA | 35 | 40 | 51 | 51 | NA |
| S231 | East Asia | 29 | 36 | 30 | 31 | 40 | 55 | 40 | 46 | S695 | East Asia | 27 | 31 | 34 | 32 | 70 | 57 | 47 | 114 |
| S232 | East Asia | 32 | 36 | 31 | 31 | 39 | 55 | 42 | 48 | S696 | East Asia | 26 | 32 | 34 | 34 | 39 | 61 | 41 | 51 |
| S233 | East Asia | 27 | 32 | 28 | 34 | 40 | 48 | 37 | 49 | S697 | South Asia | NA | 40 | 42 | 42 | 125 | 120 | 120 | 120 |
| S234 | East Asia | 28 | 35 | 30 | 32 | 41 | 31 | 40 | 42 | S698 | South Asia | NA | 39 | 45 | 41 | 125 | 120 | 120 | 120 |
| S235 | East Asia | 32 | 32 | 31 | 32 | 45 | 54 | 42 | 44 | S699 | East Africa | 30 | 31 | 32 | 32 | 43 | 93 | 120 | 115 |
| S236 | East Asia | 29 | 37 | 31 | 33 | 46 | 53 | 43 | 46 | S700 | East Africa | 34 | 34 | 37 | 34 | 81 | 120 | 120 | 120 |
| S237 | East Asia | 28 | NA | 32 | 31 | 45 | 53 | 38 | 45 | S701 | South America | 33 | 30 | 32 | 33 | 32 | 52 | 53 | 47 |
| S238 | East Asia | 29 | 42 | 34 | 32 | 46 | 53 | 38 | 48 | S702 | East Africa | 34 | 34 | 37 | 36 | 78 | 119 | 120 | 114 |
| S239 | East Asia | 28 | 36 | 34 | 35 | 39 | 56 | 38 | 57 | S703 | South America | 30 | 33 | 34 | 34 | 32 | 60 | 47 | 55 |
| S240 | East Asia | 30 | 37 | 34 | 34 | 35 | 61 | 47 | 51 | S704 | West Africa | NA | 37 | 45 | 56 | 33 | 124 | 120 | NA |
| S241 | East Asia | 29 | 30 | 37 | 34 | 36 | 81 | 46 | 56 | S705 | South-East Asia | 39 | 42 | 38 | 49 | 88 | 92 | 120 | 103 |
| S242 | East Asia | 33 | 27 | 34 | 33 | 36 | 55 | 43 | 75 | S706 | South-East Asia | 39 | 39 | 37 | 38 | 67 | 92 | 120 | 103 |
| S243 | East Asia | 33 | 31 | 34 | 34 | 32 | 65 | 57 | 50 | S707 | Centra Africa | 31 | 40 | 32 | 33 | NA | 92 | 120 | 104 |
| S244 | East Asia | 34 | 30 | 32 | 34 | 35 | 62 | 47 | 60 | S708 | South America | 28 | 30 | 32 | 35 | 42 | 61 | 41 | 57 |
| S245 | East Asia | 32 | 30 | 32 | 35 | 38 | 63 | 53 | 57 | S709 | West Africa | 33 | 34 | 32 | 34 | 39 | 92 | 120 | 104 |
| S246 | East Asia | 33 | 31 | 31 | 34 | 37 | 50 | 41 | 58 | S710 | East Africa | NA | 37 | 45 | 47 | 92 | 120 | 120 | 120 |
| S247 | East Asia | 33 | 30 | 34 | 33 | 35 | 55 | 45 | 44 | S711 | East Africa | 41 | 37 | 45 | 50 | 74 | 84 | 120 | 79 |
| S248 | East Asia | 33 | 30 | 32 | 33 | 35 | 80 | NA | 50 | S712 | East Africa | 35 | 43 | 42 | 38 | 106 | 120 | 120 | 120 |
| S249 | East Asia | 29 | 32 | 34 | 32 | 37 | 45 | 39 | 74 | S713 | East Africa | 30 | 32 | 30 | 32 | 75 | 107 | 69 | 102 |
| S250 | East Asia | 28 | 30 | 32 | 34 | 32 | 55 | 45 | 39 | S714 | East Africa | 30 | 32 | 32 | 36 | 77 | 114 | 86 | 108 |
| S251 | East Asia | 29 | 31 | 37 | 33 | 37 | 60 | 44 | 49 | S715 | East Africa | 41 | 40 | 38 | 39 | 92 | 92 | 120 | 105 |
| S252 | East Asia | 28 | 29 | 30 | 33 | 43 | 31 | 39 | 54 | S716 | East Africa | 37 | 36 | 38 | 42 | 94 | 113 | 120 | 107 |
| S253 | East Asia | 30 | 32 | 32 | 37 | 37 | 57 | 54 | 43 | S717 | East Africa | 35 | 35 | 35 | 43 | 119 | 120 | 120 | 120 |
| S254 | East Asia | 30 | 31 | 33 | 34 | 49 | 53 | 42 | 53 | S718 | East Africa | 34 | 35 | 37 | 39 | 90 | 122 | 120 | 116 |
| S255 | East Asia | 33 | 30 | 34 | 36 | 49 | 51 | 41 | 51 | S719 | West Africa | NA | 43 | 45 | 47 | 59 | 118 | 120 | 112 |
| S256 | East Asia | 30 | 32 | 32 | 34 | 39 | 51 | 44 | 43 | S720 | West Asia | 31 | 31 | 33 | 32 | 91 | 53 | 59 | 45 |
| S257 | East Asia | 30 | 31 | 32 | 34 | 42 | 57 | 46 | 45 | S721 | West Africa | NA | 42 | 41 | 47 | 47 | NA | 120 | NA |
| S258 | East Asia | 33 | 31 | 34 | 35 | 30 | 57 | 58 | 46 | S722 | East Asia | 28 | 30 | NA | 35 | 33 | 46 | 38 | 38 |
| S259 | East Asia | 27 | 30 | 33 | 34 | 39 | 31 | 41 | 45 | S723 | East Asia | 30 | 29 | NA | 36 | 38 | 53 | 48 | 53 |
| S260 | East Asia | 28 | 32 | 32 | 37 | 36 | 57 | 42 | 44 | S724 | East Asia | 31 | 32 | NA | 36 | 46 | 63 | 51 | 49 |
| S261 | East Asia | 33 | 30 | 32 | 33 | 36 | 55 | 50 | 49 | S725 | South Asia | 30 | 29 | 33 | 34 | 46 | 64 | 52 | 58 |
| S262 | East Asia | 28 | 32 | 33 | 32 | 38 | 51 | 42 | 49 | S726 | South Asia | 30 | 31 | 33 | 34 | 39 | 53 | 47 | 46 |
| S263 | East Asia | 26 | 36 | 34 | 38 | 43 | 51 | 40 | 45 | S727 | South-East Asia | 33 | 34 | 35 | 37 | 35 | 82 | 52 | 75 |
| S264 | East Asia | 27 | 33 | 35 | 34 | 38 | 47 | 48 | 45 | S728 | South-East Asia | 31 | 32 | 34 | 39 | 40 | 67 | 56 | 61 |
| S265 | East Asia | 29 | 31 | 37 | 34 | 40 | 61 | 48 | 40 | S729 | North America | 28 | 28 | 31 | 34 | 43 | 47 | 43 | 42 |
| S266 | East Asia | 28 | 29 | 33 | 35 | 35 | 57 | 44 | 56 | S730 | North America | 29 | 33 | 34 | 39 | 40 | 70 | 52 | 64 |
| S267 | East Asia | 29 | 30 | 33 | 35 | 36 | 47 | 41 | 43 | S731 | East Asia | 31 | 30 | 34 | 34 | 44 | 82 | 69 | 76 |
| S268 | East Asia | 28 | 34 | 32 | 34 | 40 | 47 | 47 | 39 | S732 | East Asia | 31 | 34 | 34 | 38 | 39 | 62 | 48 | 56 |
| S269 | East Asia | 28 | 31 | 34 | 33 | 45 | 51 | 39 | 41 | S733 | East Asia | 28 | 30 | 30 | 34 | 28 | 57 | 44 | 51 |
| S270 | East Asia | 28 | 30 | 34 | 35 | 39 | 47 | 40 | 46 | S734 | East Asia | 28 | 27 | 29 | 32 | 43 | 64 | 44 | 58 |
| S271 | East Asia | NA | 30 | 33 | 34 | 38 | 55 | 39 | 41 | S735 | East Asia | 28 | 28 | 32 | 32 | 49 | 63 | 50 | 57 |
| S272 | East Asia | 29 | 30 | 30 | 35 | 45 | 45 | 39 | 48 | S736 | Centra Africa | 33 | 32 | 34 | 34 | 41 | 65 | 47 | 59 |
| S273 | East Asia | 27 | 30 | 31 | 35 | 46 | 44 | 40 | 39 | S737 | Southern Europe | 33 | 33 | 34 | 34 | 40 | 48 | 41 | 42 |
| S274 | East Asia | 28 | 31 | 32 | 38 | 48 | 51 | 39 | 39 | S738 | South America | 32 | 32 | 36 | 38 | 43 | 67 | 53 | 63 |
| S275 | East Asia | 33 | 31 | 33 | 34 | 39 | 51 | 40 | 46 | S739 | South America | 31 | 30 | 32 | 34 | 39 | 31 | 50 | 44 |
| S276 | East Asia | 31 | 31 | 31 | 36 | 47 | 31 | 40 | 47 | S740 | South-East Asia | 34 | 32 | 34 | 38 | 40 | 62 | 46 | 55 |
| S277 | East Asia | 33 | 32 | 31 | 36 | 38 | 51 | 43 | 43 | S741 | East Asia | 32 | 44 | 44 | 46 | 78 | 57 | 46 | 55 |
| S278 | East Asia | 28 | 31 | 35 | 41 | 40 | 57 | 41 | 47 | S742 | East Asia | 28 | 34 | 34 | 38 | 39 | 48 | 44 | 46 |
| S279 | East Asia | 28 | 34 | 32 | 36 | 40 | 50 | 39 | 53 | S743 | East Asia | 31 | 34 | 36 | 39 | 38 | 65 | 45 | 42 |
| S280 | East Asia | 28 | 32 | 33 | 33 | 38 | 53 | 40 | 49 | S744 | East Asia | 33 | 29 | 37 | 45 | 47 | 51 | 40 | 44 |
| S281 | East Asia | 30 | 33 | 32 | 36 | 36 | 63 | 44 | 43 | S745 | East Asia | 33 | 34 | 35 | 37 | 37 | 51 | 41 | 47 |
| S282 | East Asia | 34 | 31 | 30 | 35 | 34 | 57 | 52 | 49 | S746 | East Asia | 30 | 32 | 31 | 33 | 39 | 51 | 40 | 48 |
| S283 | East Asia | 32 | 32 | 36 | 35 | 37 | 57 | 38 | 50 | S747 | East Asia | 30 | 32 | 31 | 33 | 48 | 51 | 40 | 45 |
| S284 | East Asia | 34 | 33 | 29 | 33 | 34 | 62 | 40 | 50 | S748 | East Asia | 28 | 32 | 34 | 33 | 39 | 51 | 39 | 49 |
| S285 | East Asia | 36 | 32 | 34 | 37 | 33 | 64 | 45 | 56 | S749 | East Asia | 28 | 44 | 34 | 33 | 35 | 62 | 40 | 48 |
| S286 | East Asia | 28 | 30 | 32 | 34 | 39 | 56 | 47 | NA | S750 | East Asia | 30 | 31 | 35 | 35 | 55 | 51 | 47 | 47 |
| S287 | East Asia | 33 | 30 | 30 | 33 | 37 | 55 | 45 | 50 | S751 | East Asia | 29 | 28 | 33 | 35 | 38 | 55 | 43 | 52 |
| S288 | East Asia | 28 | 31 | 33 | 35 | 35 | 48 | 43 | 48 | S752 | East Asia | 29 | 29 | 35 | 37 | 38 | 51 | 42 | 48 |
| S289 | East Asia | 33 | 30 | 34 | 35 | 33 | 55 | 45 | 43 | S753 | East Asia | 29 | 31 | 33 | 34 | 40 | 31 | 42 | 51 |
| S290 | East Asia | 33 | 33 | 33 | 36 | 38 | 74 | 52 | 43 | S754 | East Asia | 31 | 31 | 32 | 35 | 46 | 53 | 41 | 42 |
| S291 | East Asia | 29 | 32 | 32 | 35 | 37 | 63 | 49 | 68 | S755 | East Asia | 34 | 34 | 32 | 34 | 34 | 57 | 41 | 58 |
| S292 | East Asia | 34 | 32 | 34 | 38 | 40 | 31 | 42 | 44 | S756 | East Asia | 32 | 30 | 34 | 35 | 36 | 57 | 41 | 50 |
| S293 | East Asia | 32 | 32 | 32 | 36 | 38 | 44 | 37 | 43 | S757 | East Asia | 28 | 30 | 33 | 34 | 32 | 54 | 47 | 48 |
| S294 | East Asia | 27 | 31 | 33 | 36 | 40 | 51 | 39 | 37 | S758 | East Asia | 27 | 31 | 34 | 34 | 37 | NA | 48 | 49 |
| S295 | East Asia | 32 | 34 | 34 | 35 | 40 | 54 | 44 | 44 | S759 | East Asia | 26 | 31 | 31 | 37 | 39 | 31 | 43 | 49 |
| S296 | East Asia | 36 | 31 | 32 | 35 | 39 | 58 | 53 | 42 | S760 | East Asia | 32 | 32 | 30 | 35 | 40 | 47 | 45 | 43 |
| S297 | East Asia | 29 | 31 | 32 | 35 | 47 | 45 | 41 | 49 | S761 | East Asia | 32 | 30 | 31 | 34 | 40 | 55 | 49 | 53 |
| S298 | East Asia | 29 | 29 | 32 | 37 | 39 | 47 | 52 | 42 | S762 | East Asia | 29 | 32 | 34 | 34 | 46 | 47 | 52 | 39 |
| S299 | East Asia | 31 | 30 | 34 | 33 | 39 | 47 | 44 | 42 | S763 | East Asia | 33 | 30 | 34 | 34 | 40 | 47 | 44 | 40 |
| S300 | East Asia | 28 | 29 | 35 | 34 | 35 | 42 | 38 | 41 | S764 | East Asia | 29 | 30 | 31 | 34 | 39 | 57 | 47 | 49 |
| S301 | East Asia | 33 | 28 | 35 | 36 | 36 | 45 | 43 | 36 | S765 | East Asia | 34 | 30 | 38 | 37 | 41 | 54 | 40 | 46 |
| S302 | East Asia | 31 | 29 | 32 | 35 | 39 | 48 | 40 | 42 | S766 | East Asia | 29 | 32 | 33 | 35 | 37 | 45 | 38 | 48 |
| S303 | East Asia | 29 | 30 | 32 | 35 | 40 | 44 | 38 | 43 | S767 | East Asia | 32 | 31 | 34 | 35 | 39 | 48 | 42 | 45 |
| S304 | East Asia | 33 | 32 | 33 | 34 | 48 | 48 | 38 | 38 | S768 | East Asia | 29 | 32 | 31 | 35 | 39 | 44 | 38 | 37 |
| S305 | East Asia | 34 | 29 | 35 | 34 | 45 | 46 | 44 | 42 | S769 | East Asia | 30 | 31 | 32 | 35 | 43 | 55 | 48 | 37 |
| S306 | East Asia | 29 | 30 | 33 | 35 | 36 | 45 | 40 | 41 | S770 | East Asia | 33 | 31 | 34 | 34 | 36 | 66 | 48 | 51 |
| S307 | East Asia | 31 | 28 | 34 | 34 | 38 | 32 | 53 | 45 | S771 | East Asia | 30 | 31 | 29 | 35 | 38 | 54 | 49 | 48 |
| S308 | East Asia | 27 | 31 | 33 | 35 | 37 | 42 | 41 | 40 | S772 | East Asia | 30 | 31 | 34 | 35 | 35 | 45 | 38 | 44 |
| S309 | East Asia | 29 | 29 | 32 | 34 | 37 | 65 | 48 | 37 | S773 | East Asia | 30 | 33 | 32 | 35 | 33 | 47 | 38 | 42 |
| S310 | East Asia | 29 | 30 | 32 | 38 | 38 | 51 | 44 | 59 | S774 | East Asia | 29 | 31 | 34 | 33 | 37 | 44 | 40 | 43 |
| S311 | East Asia | 29 | 31 | 34 | 33 | 32 | 55 | 40 | 46 | S775 | East Asia | 32 | 31 | 31 | 35 | 36 | 47 | 43 | 41 |
| S312 | East Asia | 29 | 29 | 31 | 37 | 40 | 65 | 55 | 51 | S776 | East Asia | 34 | 33 | 31 | 37 | 37 | 31 | 38 | 45 |
| S313 | East Asia | 30 | 36 | 32 | 35 | 37 | 55 | 51 | 59 | S777 | East Asia | 28 | 31 | 35 | 35 | 39 | 31 | 38 | 43 |
| S314 | East Asia | 28 | 36 | 32 | 34 | 40 | 65 | 44 | 50 | S778 | East Asia | 32 | 35 | 33 | 35 | 40 | 52 | 38 | 44 |
| S315 | East Asia | 29 | 29 | 31 | 34 | 47 | 51 | 38 | 60 | S779 | East Asia | 29 | 30 | 33 | 37 | 35 | 48 | 38 | 46 |
| S316 | East Asia | 28 | 30 | 31 | 35 | 44 | 45 | 44 | 43 | S780 | East Asia | 29 | 32 | 31 | 35 | 35 | 48 | 42 | 43 |
| S317 | East Asia | 30 | 30 | 31 | 35 | 44 | 46 | 41 | 39 | S781 | East Asia | 33 | 30 | 34 | 37 | 38 | 31 | 52 | 38 |
| S318 | East Asia | 30 | 29 | 32 | 36 | 44 | 56 | 41 | 40 | S782 | East Asia | 28 | 27 | 41 | 36 | 48 | 41 | 34 | 37 |
| S319 | East Asia | 30 | 30 | 32 | 35 | 47 | 52 | 39 | 50 | S783 | East Asia | 29 | 34 | 34 | 35 | 44 | 44 | 34 | 37 |
| S320 | East Asia | 29 | 29 | 36 | 35 | 44 | 52 | 40 | 48 | S784 | East Asia | 28 | 31 | 34 | 38 | 47 | 30 | 35 | 40 |
| S321 | East Asia | 29 | 32 | 32 | 36 | 48 | 51 | 44 | 46 | S785 | East Asia | 29 | 32 | 36 | 35 | 53 | 30 | 35 | 43 |
| S322 | East Asia | 29 | 31 | 34 | 33 | 46 | 46 | 40 | 45 | S786 | East Asia | 33 | 32 | 34 | 35 | 40 | 47 | 35 | 35 |
| S323 | East Asia | 28 | 33 | 31 | 35 | 44 | 47 | 44 | 41 | S787 | East Asia | 31 | 30 | 34 | 35 | 37 | 57 | 43 | 43 |
| S324 | East Asia | 32 | 30 | 32 | 38 | 47 | 54 | 44 | 42 | S788 | East Asia | 33 | 31 | 32 | 34 | 34 | 55 | 48 | 41 |
| S325 | East Asia | 28 | 32 | 34 | 38 | 45 | 46 | 40 | 49 | S789 | East Asia | 30 | 29 | 31 | 35 | 39 | 44 | 36 | 40 |
| S326 | East Asia | 34 | 33 | 32 | 35 | 38 | 50 | 43 | 41 | S790 | East Asia | 34 | 33 | 31 | 35 | 38 | 44 | 43 | 40 |
| S327 | East Asia | 33 | 32 | 32 | 35 | 43 | 51 | 44 | 45 | S791 | East Asia | 30 | 32 | 31 | 34 | 35 | 44 | 38 | 40 |
| S328 | East Asia | 33 | 32 | 35 | 35 | 48 | 55 | 41 | 46 | S792 | East Asia | 33 | 30 | 30 | 35 | 34 | 47 | 39 | 40 |
| S329 | East Asia | 33 | 34 | 34 | 33 | 38 | 56 | 41 | 50 | S793 | East Asia | 33 | 30 | 35 | 34 | 35 | 48 | 40 | 42 |
| S330 | East Asia | 30 | 30 | 32 | 34 | 43 | 67 | 46 | 48 | S794 | East Asia | 32 | 30 | 32 | 34 | 31 | 43 | 38 | 35 |
| S331 | East Asia | 30 | 31 | 31 | 34 | 38 | 60 | 40 | 60 | S795 | East Asia | 28 | 31 | 34 | 34 | 35 | 44 | 34 | 37 |
| S332 | East Asia | 30 | 32 | 32 | 39 | 43 | 60 | 48 | 53 | S796 | East Asia | 29 | 31 | 34 | 35 | 29 | 40 | 35 | 47 |
| S333 | East Asia | 30 | 28 | 32 | 35 | 38 | 47 | 47 | 53 | S797 | East Asia | 29 | 30 | 35 | 34 | 35 | 41 | 34 | 35 |
| S334 | East Asia | 34 | 32 | 41 | 34 | 41 | 65 | 66 | 53 | S798 | East Asia | 29 | 30 | NA | 35 | 36 | 40 | 35 | 35 |
| S335 | East Asia | 30 | 30 | 34 | 34 | 43 | 47 | 40 | 41 | S799 | East Asia | 27 | 30 | NA | 35 | 29 | 30 | 35 | 39 |
| S336 | East Asia | 32 | 31 | 32 | 34 | 48 | 51 | 41 | 41 | S800 | East Asia | 26 | 37 | NA | 35 | 38 | 41 | 32 | 33 |
| S337 | East Asia | 28 | 33 | 33 | 35 | 35 | 51 | 49 | 45 | S801 | East Asia | 25 | 30 | NA | 34 | 32 | 44 | 40 | 35 |
| S338 | East Asia | 31 | 31 | 32 | 38 | 37 | 56 | 49 | 45 | S802 | East Asia | NA | 32 | NA | 34 | 29 | 58 | 47 | 44 |
| S339 | East Asia | 33 | 32 | 32 | 35 | 43 | 48 | 44 | 50 | S803 | East Asia | 28 | 33 | NA | 37 | 33 | 53 | 40 | 43 |
| S340 | East Asia | 29 | 32 | 34 | 34 | 39 | 51 | 58 | 43 | S804 | East Asia | 36 | 32 | NA | 37 | 32 | 51 | 39 | 54 |
| S341 | East Asia | 32 | 32 | 33 | 34 | 43 | 51 | 42 | 46 | S805 | East Asia | 28 | 35 | NA | 35 | 33 | 31 | 41 | 44 |
| S342 | East Asia | 34 | 32 | 34 | 34 | 45 | 54 | 48 | 46 | S806 | East Asia | 28 | 28 | NA | 35 | 41 | 46 | 40 | 51 |
| S343 | East Asia | 33 | 30 | 33 | 36 | 45 | 56 | 55 | 40 | S807 | East Asia | 36 | 30 | NA | 34 | 44 | 73 | 59 | 42 |
| S344 | East Asia | 34 | 32 | 33 | 35 | 38 | 51 | 42 | 51 | S808 | East Asia | 34 | 30 | NA | 35 | 47 | 71 | 60 | 67 |
| S345 | East Asia | 33 | 30 | 33 | 35 | 48 | 54 | 43 | 59 | S809 | East Asia | 33 | 32 | NA | 35 | 51 | 58 | 52 | 65 |
| S346 | East Asia | 34 | 32 | 34 | 40 | 54 | 51 | 42 | 48 | S810 | East Asia | 33 | 28 | NA | 33 | 47 | 48 | 40 | 41 |
| S347 | East Asia | 34 | 31 | 33 | 36 | 45 | 48 | 44 | 42 | S811 | East Asia | 29 | 36 | NA | 35 | 28 | 51 | 51 | 49 |
| S348 | East Asia | 33 | 30 | 34 | 35 | 38 | 31 | 42 | 42 | S812 | East Asia | 28 | 30 | NA | 35 | 36 | 46 | 38 | 41 |
| S349 | East Asia | 31 | 33 | 34 | 35 | 49 | 31 | 45 | 43 | S813 | East Asia | 31 | 35 | NA | 47 | 38 | 54 | 41 | 43 |
| S350 | East Asia | 33 | 32 | 34 | 35 | 32 | 51 | 42 | 43 | S814 | East Asia | 28 | 35 | NA | 35 | 33 | 51 | 40 | 47 |
| S351 | East Asia | 33 | 31 | 34 | 34 | 44 | 48 | 42 | 43 | S815 | South Asia | 37 | 41 | 38 | 38 | 61 | 74 | 69 | 67 |
| S352 | East Asia | 28 | 31 | 33 | 34 | 44 | 47 | 42 | 48 | S816 | South Asia | 30 | 30 | 32 | 34 | 59 | 82 | 48 | 75 |
| S353 | East Asia | 28 | 31 | 33 | 35 | 42 | 55 | 48 | 46 | S817 | South Asia | 34 | 35 | 34 | 36 | 49 | 83 | 71 | 77 |
| S354 | East Asia | 28 | 29 | 33 | 34 | 54 | 45 | 39 | 41 | S818 | South Asia | 25 | 30 | 30 | 31 | 52 | 47 | 54 | 40 |
| S355 | East Asia | 29 | 32 | 34 | 35 | 43 | 45 | 39 | 40 | S819 | East Africa | 35 | 35 | 38 | 39 | 59 | 83 | 80 | 78 |
| S356 | East Asia | 31 | 31 | 34 | 34 | 38 | 45 | 40 | 39 | S820 | East Africa | 40 | 37 | 44 | 46 | NA | 120 | 89 | 120 |
| S357 | East Asia | 33 | 30 | 33 | 33 | 39 | 47 | 39 | 40 | S821 | South Africa | 30 | 31 | 32 | 40 | 40 | 51 | 53 | 46 |
| S358 | East Asia | 33 | 32 | 34 | 35 | 39 | 45 | 38 | 42 | S822 | East Africa | 30 | 31 | 33 | 32 | 40 | 54 | 53 | 48 |
| S359 | East Asia | 28 | 32 | 31 | 32 | 38 | 42 | 40 | 40 | S823 | East Africa | 31 | 32 | 32 | 34 | 62 | NA | 75 | 120 |
| S360 | East Asia | 30 | 30 | 31 | 35 | 42 | 44 | 40 | 38 | S824 | East Africa | NA | 33 | 35 | 34 | 36 | 123 | 120 | NA |
| S361 | East Asia | 29 | 32 | 33 | 35 | 39 | 45 | 41 | 39 | S825 | South-East Asia | 35 | 35 | 41 | 42 | 60 | 107 | 71 | 101 |
| S362 | East Asia | 30 | 30 | 33 | 33 | 39 | 51 | 43 | 40 | S826 | South-East Asia | 34 | 35 | 37 | 40 | 29 | 60 | 69 | 63 |
| S363 | East Asia | 28 | 31 | 32 | 35 | 36 | 46 | 40 | 46 | S827 | Central America | 29 | 33 | 33 | 35 | 43 | 55 | 40 | 50 |
| S364 | East Asia | 27 | 30 | 31 | 34 | 36 | 42 | 38 | 41 | S828 | Central America | 29 | 42 | 44 | 47 | 45 | 120 | 120 | 120 |
| S365 | East Asia | 29 | 31 | 34 | 34 | 38 | 47 | 40 | 50 | S829 | Central America | 29 | 30 | 31 | 32 | 59 | 48 | 42 | 42 |
| S366 | East Asia | 29 | 31 | 33 | 33 | 40 | 45 | 39 | 40 | S830 | West Africa | 28 | 31 | 33 | 34 | 35 | 31 | 47 | 43 |
| S367 | East Asia | 29 | 30 | 34 | 35 | 40 | 45 | 37 | 40 | S831 | East Africa | 40 | 37 | 36 | 34 | 79 | 92 | 120 | 104 |
| S368 | East Asia | 36 | 30 | 29 | 35 | 38 | 45 | 38 | 40 | S832 | East Africa | 35 | 34 | 37 | 40 | 35 | 124 | 120 | 118 |
| S369 | East Asia | 30 | 31 | 29 | 34 | 35 | 48 | 40 | 39 | S833 | West Asia | 29 | 30 | 32 | 34 | 39 | 63 | 54 | 58 |
| S370 | East Asia | 30 | 31 | 22 | 40 | 35 | 50 | 39 | 43 | S834 | West Asia | 27 | 29 | 31 | 32 | 91 | 54 | 39 | 48 |
| S371 | East Asia | 28 | 32 | 31 | 33 | 38 | 51 | 40 | 45 | S835 | East Africa | 38 | 41 | 38 | 41 | 40 | 80 | 69 | 74 |
| S372 | East Asia | 27 | 32 | 30 | 34 | 45 | 42 | 39 | 46 | S836 | Centra Africa | 36 | 35 | 35 | 35 | 49 | 116 | 120 | 111 |
| S373 | East Asia | 33 | 32 | 30 | 35 | 44 | 51 | 41 | 36 | S837 | Centra Africa | 31 | 32 | 34 | 35 | 79 | 31 | 45 | 44 |
| S374 | East Asia | 34 | 35 | 33 | 34 | 46 | 46 | 37 | 45 | S838 | West Africa | 30 | 32 | 31 | 32 | 53 | 109 | 120 | 104 |
| S375 | East Asia | 32 | 32 | 32 | 37 | 40 | 51 | 47 | 41 | S839 | West Africa | 40 | 39 | 44 | 43 | 39 | 109 | 47 | 104 |
| S376 | East Asia | 30 | 29 | 32 | 35 | 37 | 47 | 41 | 46 | S840 | East Africa | NA | 41 | 42 | 47 | 84 | 118 | 120 | 113 |
| S377 | East Asia | 31 | 32 | 32 | 39 | 35 | 53 | 42 | 42 | S841 | East Asia | 33 | 30 | 32 | 33 | 43 | 51 | 42 | 46 |
| S378 | East Asia | 36 | 32 | 33 | 34 | 39 | 66 | 45 | 48 | S842 | East Asia | 33 | 32 | 37 | 38 | 39 | 57 | 47 | 52 |
| S379 | East Asia | 36 | 36 | 33 | 34 | 39 | 51 | 41 | 61 | S843 | South-East Asia | 26 | 29 | 31 | 32 | 39 | 55 | 48 | 50 |
| S380 | East Asia | 37 | 31 | 32 | 33 | 39 | 51 | 40 | 45 | S844 | North America | 31 | 32 | 35 | 37 | 45 | 55 | 53 | 50 |
| S381 | East Asia | 37 | 35 | 33 | 33 | 39 | 56 | 40 | 46 | S845 | North America | 29 | 31 | 34 | 35 | 46 | 63 | 47 | 58 |
| S382 | East Asia | 40 | 31 | 32 | 34 | 34 | 57 | 43 | 50 | S846 | East Asia | 28 | 29 | 31 | 34 | 43 | 67 | 44 | 61 |
| S383 | East Asia | 36 | 30 | 35 | 35 | 35 | 57 | 44 | 56 | S847 | South Asia | 32 | 32 | 35 | 36 | 78 | 60 | 52 | 55 |
| S384 | East Asia | 29 | 31 | 31 | 34 | 38 | 53 | 38 | 47 | S848 | Centra Africa | 34 | 32 | 34 | 34 | 48 | NA | 44 | 120 |
| S385 | East Asia | 28 | 32 | 31 | 35 | 44 | 46 | 38 | 49 | S849 | Southern Europe | 29 | 28 | 32 | 33 | 47 | 44 | 38 | 40 |
| S386 | East Asia | 28 | 34 | 32 | 34 | 35 | 44 | 45 | 41 | S850 | South America | 30 | 30 | 32 | 33 | 40 | 48 | 44 | 42 |
| S387 | East Asia | 30 | 31 | 32 | 34 | 36 | 51 | 42 | 39 | S851 | South-East Asia | 29 | 30 | 33 | 35 | 42 | 54 | 48 | 48 |
| S388 | East Asia | 28 | 30 | 36 | 35 | 43 | 54 | 38 | 47 | S852 | South-East Asia | 32 | 32 | 32 | 34 | 40 | 52 | 53 | 45 |
| S389 | East Asia | 28 | 32 | 32 | 33 | 42 | 53 | 39 | 49 | S853 | Caribbean | 33 | 34 | 37 | 39 | 65 | 80 | 52 | 74 |
| S390 | East Asia | 28 | 29 | 35 | 40 | 46 | 47 | 40 | 50 | S854 | South Asia | 29 | 30 | 30 | 34 | 88 | 107 | 120 | 101 |
| S391 | East Asia | 28 | 33 | 33 | 37 | 44 | 47 | 38 | 43 | S855 | East Africa | 35 | 33 | 37 | 33 | 89 | 120 | 120 | 120 |
| S392 | East Asia | 37 | 29 | 34 | 34 | 35 | 47 | 38 | 43 | S856 | East Asia | 29 | 36 | 34 | 34 | 87 | 54 | 41 | 49 |
| S393 | East Asia | 28 | 31 | 34 | 33 | 38 | 47 | 38 | 43 | S857 | East Asia | 29 | 36 | 32 | 32 | 78 | 51 | 40 | 48 |
| S394 | East Asia | 28 | 36 | 35 | 33 | 38 | 47 | 38 | 43 | S858 | East Asia | 30 | 32 | 34 | 33 | 44 | 57 | 45 | 53 |
| S395 | East Asia | 35 | 35 | 36 | 37 | 39 | 47 | 38 | 43 | S859 | East Asia | 34 | 31 | 34 | 39 | 40 | 32 | 45 | 49 |
| S396 | East Asia | 29 | 30 | 37 | 33 | 36 | 31 | 39 | 41 | S860 | East Asia | 30 | 32 | NA | 34 | 46 | 54 | 49 | 49 |
| S397 | East Asia | 36 | 30 | 34 | 33 | 34 | 48 | 39 | 42 | S861 | East Asia | 29 | 30 | NA | 35 | 52 | 47 | 41 | 63 |
| S398 | East Asia | 37 | 31 | 34 | 35 | 36 | 55 | 41 | 43 | S862 | East Asia | 31 | 30 | NA | 35 | 39 | 67 | 51 | 47 |
| S399 | East Asia | 36 | 29 | 34 | 35 | 39 | 48 | 38 | 51 | S863 | East Asia | 34 | 29 | NA | 33 | 44 | 61 | 48 | 51 |
| S400 | East Asia | 37 | 30 | 32 | 37 | 39 | 48 | 39 | 44 | S864 | West Africa | 36 | 34 | 37 | 42 | 38 | 122 | 87 | 116 |
| S401 | East Asia | 28 | 32 | 31 | 33 | 35 | 44 | 38 | 41 | S865 | South Asia | NA | 38 | 38 | 47 | 92 | 120 | 120 | 120 |
| S402 | East Asia | 29 | 32 | 34 | 33 | 35 | 46 | 43 | 39 | S866 | East Asia | 30 | 33 | NA | 37 | 39 | 47 | 44 | 42 |
| S403 | East Asia | 30 | 31 | 35 | 33 | 35 | 51 | 41 | 43 | S867 | East Asia | 30 | 33 | NA | 35 | 38 | 48 | 40 | 40 |
| S404 | East Asia | 28 | 29 | 33 | 39 | 37 | 44 | 38 | 47 | S868 | East Asia | 29 | 32 | NA | 35 | 38 | 53 | 44 | 43 |
| S405 | East Asia | 29 | 31 | 33 | 34 | 40 | 45 | 39 | 40 | S869 | East Asia | 27 | 35 | NA | 34 | 28 | 45 | 39 | 46 |
| S406 | East Asia | 29 | 31 | 30 | 35 | 36 | 52 | 41 | 41 | S870 | East Asia | 31 | 34 | NA | 35 | 28 | 54 | 40 | 57 |
| S407 | East Asia | 33 | 28 | 31 | 35 | 38 | 31 | 39 | 48 | S871 | East Asia | 30 | 33 | NA | 34 | 41 | 31 | 37 | 43 |
| S408 | East Asia | 30 | 32 | 31 | 35 | 35 | 31 | 48 | 45 | S872 | East Asia | 30 | 33 | NA | 36 | 36 | 44 | 40 | 43 |
| S409 | East Asia | 32 | 30 | 32 | 35 | 37 | 47 | 41 | 45 | S873 | East Asia | NA | 32 | NA | 36 | 40 | 47 | 44 | 40 |
| S410 | East Asia | 30 | 32 | 33 | 34 | 36 | 46 | 41 | 43 | S874 | East Asia | 33 | 32 | NA | 38 | 51 | 57 | 52 | 42 |
| S411 | East Asia | 31 | 34 | 31 | 35 | 36 | 52 | 44 | 41 | S875 | East Asia | 29 | 31 | NA | 33 | 35 | 57 | 42 | 52 |
| S412 | East Asia | 32 | 35 | 33 | 34 | 37 | 60 | 45 | 48 | S876 | East Asia | 27 | 33 | NA | 34 | 32 | 51 | 40 | 50 |
| S413 | East Asia | 32 | 35 | 34 | 35 | 34 | 56 | 44 | 65 | S877 | East Asia | 27 | 30 | NA | 34 | 33 | 48 | 44 | 45 |
| S414 | East Asia | 30 | 31 | 31 | 34 | 34 | 46 | 42 | 52 | S878 | East Asia | 29 | 32 | NA | 35 | 34 | 52 | 44 | 42 |
| S415 | East Asia | 29 | 33 | 34 | 35 | 35 | 46 | 42 | 42 | S879 | East Asia | 29 | 32 | NA | 37 | 38 | 52 | 42 | 46 |
| S416 | East Asia | 30 | 31 | 33 | 34 | 37 | 45 | 40 | 42 | S880 | East Asia | 29 | 32 | NA | 35 | 36 | 55 | 38 | 46 |
| S417 | East Asia | 30 | 31 | 34 | 34 | 36 | 47 | 41 | 41 | S881 | East Asia | 28 | 31 | NA | 34 | 39 | 55 | 40 | 58 |
| S418 | East Asia | 33 | 30 | 33 | 35 | 40 | 47 | 45 | 43 | S882 | East Asia | 31 | 32 | NA | 34 | 43 | 63 | 41 | 50 |
| S419 | East Asia | 33 | 30 | 34 | 35 | 39 | 51 | 38 | 40 | S883 | East Asia | 29 | 31 | NA | 37 | 45 | 55 | 53 | 58 |
| S420 | East Asia | 29 | 30 | 32 | 35 | 41 | 47 | 40 | 43 | S884 | East Asia | 28 | 32 | NA | 36 | 29 | 54 | 42 | 50 |
| S421 | East Asia | 30 | 31 | 34 | 35 | 39 | 47 | 39 | 43 | S885 | East Africa | NA | 40 | 46 | 50 | 92 | 120 | 120 | 120 |
| S422 | East Asia | 29 | 29 | 34 | 35 | 39 | 46 | 41 | 41 | S886 | East Africa | 34 | 34 | 35 | 33 | NA | 66 | 59 | 61 |
| S423 | East Asia | 28 | 32 | 33 | 35 | 40 | 55 | 45 | 42 | S887 | East Africa | NA | 44 | 44 | 46 | 60 | 120 | 120 | 120 |
| S424 | East Asia | 30 | 31 | 35 | 35 | 40 | 44 | 39 | 43 | S888 | East Africa | 38 | 35 | 32 | 32 | 40 | 122 | 120 | 114 |
| S425 | East Asia | 30 | 31 | 34 | 33 | 37 | 31 | 41 | 39 | S889 | East Africa | 33 | 30 | 33 | 33 | 71 | 122 | 45 | 117 |
| S426 | East Asia | 34 | 32 | 33 | 35 | 35 | 55 | 44 | 42 | S890 | East Africa | 33 | 31 | 32 | 34 | 60 | 120 | 120 | 120 |
| S427 | East Asia | 33 | 30 | 32 | 37 | 35 | 31 | 41 | 49 | S891 | East Africa | 34 | 31 | 32 | 33 | 60 | 122 | 86 | 116 |
| S428 | East Asia | 33 | 32 | 32 | 34 | 35 | 47 | 41 | 43 | S892 | East Africa | 35 | 34 | 35 | 37 | 77 | 107 | 120 | 101 |
| S429 | East Asia | 35 | 32 | 33 | 38 | 36 | 60 | 42 | 41 | S893 | East Africa | 39 | 34 | 31 | 43 | 61 | 82 | 67 | 76 |
| S430 | East Asia | 30 | 33 | 32 | 35 | 38 | 51 | 39 | 53 | S894 | East Africa | 41 | 31 | 42 | 43 | 65 | 107 | 120 | 102 |
| S431 | East Asia | 33 | 32 | 35 | 37 | 40 | 52 | 48 | 42 | S895 | East Africa | 36 | 37 | 38 | 43 | 62 | 91 | 120 | 94 |
| S432 | East Asia | 33 | 32 | 33 | 35 | 38 | 45 | 42 | 47 | S896 | South-East Asia | 31 | 32 | 37 | 36 | 60 | 63 | 56 | 57 |
| S433 | East Asia | 35 | 34 | 34 | 35 | 37 | 44 | 41 | 39 | S897 | South-East Asia | 29 | 29 | 33 | 32 | 43 | 65 | 56 | 59 |
| S434 | East Asia | 30 | 31 | 34 | 35 | 32 | 44 | 45 | 39 | S898 | Centra Africa | 34 | 33 | 36 | 38 | 41 | 65 | 67 | 59 |
| S435 | East Asia | 34 | 31 | 34 | 35 | 36 | 46 | 44 | 38 | S899 | Centra Africa | 32 | 32 | 34 | 35 | 60 | 53 | 47 | 47 |
| S436 | East Asia | 32 | 32 | 32 | 35 | 35 | 48 | 43 | 40 | S900 | East Africa | 40 | 39 | 38 | 42 | 84 | 107 | 73 | 103 |
| S437 | East Asia | 34 | 31 | 35 | 35 | 33 | 54 | 48 | 42 | S901 | East Asia | 30 | 30 | 31 | 34 | 49 | 65 | 44 | 59 |
| S438 | East Asia | 32 | 32 | 34 | 35 | 36 | 54 | 52 | 48 | S902 | Eastern Europe | 33 | 32 | 37 | 34 | 38 | 62 | 68 | 57 |
| S439 | East Asia | 32 | 32 | 34 | 35 | 36 | 30 | 47 | 48 | S903 | South Asia | 33 | 31 | 32 | 34 | NA | 67 | 62 | 61 |
| S440 | East Asia | 32 | 31 | 33 | 34 | 35 | 48 | 52 | 34 | S904 | South Asia | NA | 46 | 45 | 48 | 81 | 120 | 120 | 120 |
| S441 | East Asia | 31 | 31 | 34 | 33 | 37 | 42 | 42 | 41 | S905 | South Asia | NA | 40 | 45 | 44 | 40 | 120 | 120 | 120 |
| S442 | East Asia | 33 | 30 | 32 | 34 | 38 | 44 | 40 | 43 | S906 | East Asia | 29 | 32 | 34 | 37 | 53 | 55 | 41 | 57 |
| S443 | East Asia | 33 | 30 | 36 | 34 | 40 | 54 | 45 | 38 | S907 | East Asia | 28 | 37 | 37 | 40 | 37 | 52 | 42 | 44 |
| S444 | East Asia | 33 | 30 | 35 | 34 | 41 | 47 | 45 | 48 | S908 | East Asia | 30 | 44 | 31 | 36 | 45 | 51 | 45 | 57 |
| S445 | East Asia | 32 | 32 | 36 | 35 | 44 | 52 | 44 | 43 | S909 | East Asia | 33 | 35 | 35 | 35 | 43 | 32 | 48 | 45 |
| S446 | East Asia | 31 | 30 | 35 | 35 | 40 | 48 | 40 | 48 | S910 | East Asia | 27 | 31 | 34 | 34 | 46 | 31 | 41 | 43 |
| S447 | East Asia | 31 | 29 | 33 | 35 | 38 | 31 | 41 | 44 | S911 | East Asia | 27 | 32 | 33 | 34 | 36 | 55 | 45 | 51 |
| S448 | East Asia | 34 | 30 | 35 | 35 | 41 | 55 | 44 | 45 | S912 | East Asia | 30 | 30 | 31 | 33 | 40 | 55 | 49 | 41 |
| S449 | East Asia | 33 | 29 | 36 | 35 | 44 | 53 | 41 | 51 | S913 | East Asia | 30 | 31 | 32 | 37 | 40 | 55 | 39 | 44 |
| S450 | East Asia | 30 | 30 | 37 | 37 | 42 | 51 | 40 | 49 | S914 | East Asia | 29 | 33 | 44 | 34 | 40 | 51 | 40 | 47 |
| S451 | East Asia | 33 | 32 | 34 | 36 | 35 | 53 | 48 | 47 | S915 | East Asia | 30 | 32 | 31 | 33 | 36 | 31 | 39 | 46 |
| S452 | East Asia | 32 | 30 | 34 | 34 | 43 | 51 | 44 | 49 | S916 | East Asia | 33 | 33 | 32 | 37 | 39 | 55 | 42 | 53 |
| S453 | East Asia | 33 | 30 | 34 | 34 | 40 | 31 | 42 | 47 | S917 | East Asia | 31 | 33 | 31 | 33 | 33 | 55 | 45 | 51 |
| S454 | East Asia | 32 | 31 | 35 | 38 | 36 | 54 | 44 | 55 | S918 | East Asia | 33 | 30 | 32 | 35 | 36 | 57 | 41 | 49 |
| S455 | East Asia | 32 | 33 | 32 | 34 | 38 | 55 | 46 | 45 | S919 | East Asia | 29 | 30 | 33 | 38 | 38 | 31 | 44 | 51 |
| S456 | East Asia | 31 | 31 | 33 | 35 | 36 | 51 | 42 | 51 | S920 | East Asia | 32 | 30 | 34 | 35 | 40 | 52 | 44 | 42 |
| S457 | East Asia | 31 | 33 | 34 | 33 | 44 | 60 | 52 | 51 | S921 | East Asia | 38 | 28 | 35 | 35 | 35 | 55 | 40 | 50 |
| S458 | East Asia | 34 | 30 | 34 | 35 | 37 | 38 | 42 | 51 | S922 | East Asia | 31 | 32 | NA | 35 | 48 | 32 | 41 | 49 |
| S459 | East Asia | 35 | 32 | 34 | 34 | 37 | 31 | 40 | 33 | S923 | East Asia | 30 | 31 | NA | 35 | 45 | 45 | 44 | 65 |
| S460 | East Asia | 31 | 32 | 37 | 34 | 38 | 50 | 42 | 45 | S924 | East Asia | 30 | 29 | NA | 33 | 44 | 46 | 57 | 40 |
| S461 | East Asia | 29 | 32 | 37 | 35 | 42 | 51 | 43 | 46 | S925 | East Asia | 33 | 33 | NA | 34 | 49 | 58 | 53 | 40 |
| S462 | East Asia | 30 | 32 | 37 | 35 | 37 | 41 | 39 | 47 | S926 | East Africa | 33 | 33 | 32 | 34 | 74 | 82 | 67 | 76 |
| S463 | East Asia | 28 | 31 | 35 | 35 | 34 | 36 | 31 | 39 | S927 | East Africa | 34 | 34 | 37 | 41 | 46 | 91 | 120 | 85 |
| S464 | East Asia | 28 | 32 | 33 | 35 | 42 | 44 | 38 | 32 |  |  |  |  |  |  |  |  |  |  |

| **Table S17 Phenotypic characteristics for flowering time in the 245** Sesamum indicum **accessions planted in Yuanyang.** | | | | | |
| --- | --- | --- | --- | --- | --- |
| **Year** | **Flowering time (days)** | | | | |
|  | **Min.** | **Max.** | **Mean ± SD** | **CV%** | ***H***^2^ ***± SE*** |
| 2019 | 29 | 125 | 53.04 ± 21.97 | 41.32 | 0.93 ± 0.06 |
| 2020 | 39 | 126 | 69.84 ± 28.71 | 41.01 | 0.99 ± 0.04 |
| 2021 | 32 | 120 | 63.19± 32.48 | 51.29 | 0.99 ± 0.04 |
| 2022 | 26 | 120 | 45.16 ± 19.38 | 42.81 | 0.99 ± 0.05 |
| SD, standard deviation; CV, coefficient of variation; H^2^, broad-sense heritability; SE, Standard Error. | | | | | |

| **Table S18 Genomic regions with high divergence between 'North China' and 'South Asia' group among the 90 sesame accessions.** | | | | | | | |
| --- | --- | --- | --- | --- | --- | --- | --- |
| **SiChr.** | **BIN_START** | **BIN_END** | **N_VARIANTS** | **Mean_WEIGHTED_FST** | **Mean_FST** | **Mean_xpclr_norm** | **Gene_IDs** |
| SiChr.12 | 11265001 | 11770000 | 10505 | 0.80 | 0.75 | 5.53 | Sin12G01063.1,Sin12G01064.1,Sin12G01065.1,Sin12G01066.1,Sin12G01067.1,Sin12G01068.1,Sin12G01069.1,Sin12G01070.1,Sin12G01071.1,Sin12G01072.1,Sin12G01073.1,Sin12G01074.1,Sin12G01075.1,Sin12G01076.1,Sin12G01077.1,Sin12G01078.1,Sin12G01079.1,Sin12G01080.1,Sin12G01081.1,Sin12G01082.1,Sin12G01083.2,Sin12G01084.1,Sin12G01085.1,Sin12G01086.1,Sin12G01087.1,Sin12G01088.1,Sin12G01089.1,Sin12G01090.1,Sin12G01091.1,Sin12G01092.1,Sin12G01093.2,Sin12G01094.1,Sin12G01095.1,Sin12G01096.1,Sin12G01097.1,Sin12G01098.1,Sin12G01099.1,Sin12G01100.1,Sin12G01101.2,Sin12G01102.1,Sin12G01103.1,Sin12G01104.1,Sin12G01105.1,Sin12G01106.1,Sin12G01107.1,Sin12G01108.1,Sin12G01109.1,Sin12G01110.1,Sin12G01111.1,Sin12G01112.1,Sin12G01113.1 |
| SiChr.3 | 395001 | 565000 | 1536 | 0.83 | 0.76 | 6.76 | Sin3G00065.1,Sin3G00066.1,Sin3G00067.2,Sin3G00068.1,Sin3G00069.1,Sin3G00070.4,Sin3G00071.1,Sin3G00072.1,Sin3G00073.2,Sin3G00074.1,Sin3G00075.1,Sin3G00076.1,Sin3G00077.1,Sin3G00078.1,Sin3G00079.1,Sin3G00080.1,Sin3G00081.1,Sin3G00082.3,Sin3G00083.2,Sin3G00084.1,Sin3G00085.2,Sin3G00086.1,Sin3G00087.1,Sin3G00088.1,Sin3G00089.1,Sin3G00090.1,Sin3G00091.1,Sin3G00092.1,Sin3G00093.1,Sin3G00094.1 |
| SiChr.3 | 1640001 | 1750000 | 1191 | 0.76 | 0.68 | 5.68 | Sin3G00271.1,Sin3G00272.1,Sin3G00273.1,Sin3G00274.1,Sin3G00275.1,Sin3G00276.1,Sin3G00277.1,Sin3G00278.1,Sin3G00279.1,Sin3G00280.2,Sin3G00281.1,Sin3G00282.1,Sin3G00283.2,Sin3G00284.1,Sin3G00285.1,Sin3G00286.3 |
| SiChr.4 | 5670001 | 5705000 | 431 | 0.73 | 0.63 | 3.24 | Sin4G00728.1,Sin4G00729.3,Sin4G00730.1 |
| SiChr.12 | 9710001 | 9790000 | 1360 | 0.72 | 0.65 | 6.49 | Sin12G00869.1,Sin12G00870.1,Sin12G00871.2,Sin12G00872.1,Sin12G00873.1,Sin12G00874.1,Sin12G00875.1,Sin12G00876.1,Sin12G00877.1,Sin12G00878.1,Sin12G00879.2,Sin12G00880.1,Sin12G00881.1 |
| SiChr.3 | 5001 | 390000 | 1965 | 0.82 | 0.76 | 4.06 | Sin3G00001.1,Sin3G00002.1,Sin3G00003.1,Sin3G00004.1,Sin3G00005.1,Sin3G00006.3,Sin3G00007.1,Sin3G00008.1,Sin3G00009.1,Sin3G00010.1,Sin3G00011.1,Sin3G00012.1,Sin3G00013.1,Sin3G00014.1,Sin3G00015.1,Sin3G00016.1,Sin3G00017.1,Sin3G00018.1,Sin3G00019.1,Sin3G00020.1,Sin3G00021.1,Sin3G00022.1,Sin3G00023.1,Sin3G00024.1,Sin3G00025.1,Sin3G00026.1,Sin3G00027.1,Sin3G00028.1,Sin3G00029.1,Sin3G00030.1,Sin3G00031.1,Sin3G00032.1,Sin3G00033.1,Sin3G00034.1,Sin3G00035.1,Sin3G00036.1,Sin3G00037.1,Sin3G00038.7,Sin3G00039.1,Sin3G00040.1,Sin3G00041.1,Sin3G00042.1,Sin3G00043.1,Sin3G00044.1,Sin3G00045.1,Sin3G00046.2,Sin3G00047.1,Sin3G00048.1,Sin3G00049.1,Sin3G00050.1,Sin3G00051.1,Sin3G00052.1,Sin3G00053.1,Sin3G00054.1,Sin3G00055.1,Sin3G00056.1,Sin3G00057.1,Sin3G00058.1,Sin3G00059.1,Sin3G00060.1,Sin3G00061.2,Sin3G00062.1,Sin3G00063.5,Sin3G00064.1 |
| SiChr.4 | 7275001 | 7425000 | 1629 | 0.79 | 0.71 | 7.25 | Sin4G00969.1,Sin4G00970.1,Sin4G00971.1,Sin4G00972.2,Sin4G00973.1,Sin4G00974.1,Sin4G00975.1,Sin4G00976.3,Sin4G00977.1,Sin4G00978.1,Sin4G00979.1,Sin4G00980.1,Sin4G00981.2,Sin4G00982.1,Sin4G00983.1,Sin4G00984.3,Sin4G00985.1,Sin4G00986.1,Sin4G00987.1,Sin4G00988.1,Sin4G00989.1,Sin4G00990.1,Sin4G00991.1,Sin4G00992.1,Sin4G00993.1,Sin4G00994.1,Sin4G00995.1 |
| SiChr.2 | 24690001 | 24920000 | 3688 | 0.77 | 0.62 | 6.23 | Sin2G02237.1,Sin2G02238.1,Sin2G02239.1,Sin2G02240.1,Sin2G02241.1,Sin2G02242.1,Sin2G02243.1,Sin2G02244.2,Sin2G02245.1,Sin2G02246.1,Sin2G02247.1,Sin2G02248.1,Sin2G02249.1,Sin2G02250.1,Sin2G02251.1,Sin2G02252.3,Sin2G02253.1,Sin2G02254.1,Sin2G02255.3,Sin2G02256.1,Sin2G02257.1,Sin2G02258.1,Sin2G02259.1,Sin2G02260.1,Sin2G02261.1,Sin2G02262.2 |
| SiChr.4 | 7160001 | 7265000 | 452 | 0.74 | 0.62 | 4.56 | Sin4G00955.1,Sin4G00956.1,Sin4G00957.1,Sin4G00958.1,Sin4G00959.3,Sin4G00960.1,Sin4G00961.1,Sin4G00962.1,Sin4G00963.1,Sin4G00964.3,Sin4G00965.4,Sin4G00966.1,Sin4G00967.2 |
| SiChr.12 | 11810001 | 12035000 | 3422 | 0.74 | 0.63 | 4.93 | Sin12G01120.1,Sin12G01121.1,Sin12G01122.1,Sin12G01123.1,Sin12G01124.1,Sin12G01125.1,Sin12G01126.1,Sin12G01127.1,Sin12G01128.1,Sin12G01129.1,Sin12G01130.1,Sin12G01131.1,Sin12G01132.2,Sin12G01133.1,Sin12G01134.1,Sin12G01135.1,Sin12G01136.1,Sin12G01137.1,Sin12G01138.1,Sin12G01139.1,Sin12G01140.1,Sin12G01141.1,Sin12G01142.1 |
| SiChr.4 | 5570001 | 5655000 | 1102 | 0.72 | 0.51 | 7.13 | Sin4G00705.1,Sin4G00706.1,Sin4G00707.1,Sin4G00708.1,Sin4G00709.3,Sin4G00710.1,Sin4G00711.1,Sin4G00712.1,Sin4G00713.1,Sin4G00714.1,Sin4G00715.1,Sin4G00716.1,Sin4G00717.1,Sin4G00718.1,Sin4G00719.1,Sin4G00720.1,Sin4G00721.1,Sin4G00722.1,Sin4G00723.1 |
| SiChr.2 | 26220001 | 26320000 | 1534 | 0.74 | 0.57 | 4.36 | Sin2G02417.1,Sin2G02418.5,Sin2G02419.1,Sin2G02420.1,Sin2G02421.1,Sin2G02422.1,Sin2G02423.1,Sin2G02424.1,Sin2G02425.1,Sin2G02426.1,Sin2G02427.1,Sin2G02428.1,Sin2G02429.1,Sin2G02430.1,Sin2G02431.1,Sin2G02432.1 |
| SiChr.2 | 24990001 | 25165000 | 3635 | 0.71 | 0.52 | 7.68 | Sin2G02274.1,Sin2G02275.1,Sin2G02276.1,Sin2G02277.1,Sin2G02278.1,Sin2G02279.1,Sin2G02280.2,Sin2G02281.1,Sin2G02282.1,Sin2G02283.1,Sin2G02284.1,Sin2G02285.1,Sin2G02286.1,Sin2G02287.1,Sin2G02288.1,Sin2G02289.1,Sin2G02290.2,Sin2G02291.1,Sin2G02292.1,Sin2G02293.1 |
| SiChr.2 | 25180001 | 25460000 | 3966 | 0.73 | 0.60 | 5.41 | Sin2G02296.1,Sin2G02297.1,Sin2G02298.1,Sin2G02299.1,Sin2G02300.1,Sin2G02301.1,Sin2G02302.3,Sin2G02303.5,Sin2G02304.2,Sin2G02305.1,Sin2G02306.1,Sin2G02307.1,Sin2G02308.1,Sin2G02309.1,Sin2G02310.1,Sin2G02311.1,Sin2G02312.1,Sin2G02313.1,Sin2G02314.1,Sin2G02315.2,Sin2G02316.1,Sin2G02317.1,Sin2G02318.1,Sin2G02319.1,Sin2G02320.1,Sin2G02321.1,Sin2G02322.1,Sin2G02323.1,Sin2G02324.4,Sin2G02325.1,Sin2G02326.1,Sin2G02327.1,Sin2G02328.1,Sin2G02329.1 |
| SiChr.13 | 7010001 | 7105000 | 1264 | 0.71 | 0.63 | 5.11 | Sin13G00601.1,Sin13G00602.1,Sin13G00603.1,Sin13G00604.1,Sin13G00605.1,Sin13G00606.1,Sin13G00607.1,Sin13G00608.1,Sin13G00609.1,Sin13G00610.5,Sin13G00611.1,Sin13G00612.1 |
| SiChr.12 | 1725001 | 1830000 | 3985 | 0.73 | 0.56 | 6.94 | Sin12G00253.1,Sin12G00254.1,Sin12G00255.1,Sin12G00256.1,Sin12G00257.1,Sin12G00258.1,Sin12G00259.1,Sin12G00260.1 |
| SiChr.11 | 9495001 | 9700000 | 3501 | 0.76 | 0.69 | 7.79 | Sin11G00805.1,Sin11G00806.1,Sin11G00807.1,Sin11G00808.1,Sin11G00809.1,Sin11G00810.1,Sin11G00811.1,Sin11G00812.1,Sin11G00813.1,Sin11G00814.1,Sin11G00815.1,Sin11G00816.1,Sin11G00817.1,Sin11G00818.1,Sin11G00819.1,Sin11G00820.1,Sin11G00821.1,Sin11G00822.1,Sin11G00823.1,Sin11G00824.1,Sin11G00825.1,Sin11G00826.1,Sin11G00827.1,Sin11G00828.5,Sin11G00829.1 |
| SiChr.4 | 27590001 | 27675000 | 814 | 0.76 | 0.51 | 4.09 | Sin4G02952.1,Sin4G02953.1,Sin4G02954.1,Sin4G02955.1,Sin4G02956.1,Sin4G02957.1,Sin4G02958.1,Sin4G02959.1,Sin4G02960.1,Sin4G02961.5,Sin4G02962.1,Sin4G02963.2,Sin4G02964.1,Sin4G02965.4,Sin4G02966.1,Sin4G02967.1,Sin4G02968.1 |
| SiChr.4 | 6795001 | 6880000 | 793 | 0.74 | 0.66 | 6.26 | Sin4G00894.1,Sin4G00895.4,Sin4G00896.1,Sin4G00897.1,Sin4G00898.1,Sin4G00899.1,Sin4G00900.1,Sin4G00901.1,Sin4G00902.1,Sin4G00903.3,Sin4G00904.1,Sin4G00905.5,Sin4G00906.1,Sin4G00907.1,Sin4G00908.1 |
| SiChr.3 | 2525001 | 2575000 | 157 | 0.72 | 0.58 | 1.96 | Sin3G00374.1,Sin3G00375.1,Sin3G00376.1,Sin3G00377.1,Sin3G00378.1,Sin3G00379.1,Sin3G00380.1 |
| SiChr.1 | 22165001 | 22315000 | 2240 | 0.75 | 0.71 | 3.22 | Sin1G01838.1,Sin1G01839.1,Sin1G01840.1,Sin1G01841.1,Sin1G01842.1,Sin1G01843.1,Sin1G01844.1,Sin1G01845.1,Sin1G01846.2,Sin1G01847.1,Sin1G01848.1,Sin1G01849.1,Sin1G01850.1,Sin1G01851.1 |
| SiChr.4 | 6880001 | 7160000 | 3248 | 0.69 | 0.54 | 3.87 | Sin4G00907.1,Sin4G00909.1,Sin4G00910.1,Sin4G00911.1,Sin4G00912.1,Sin4G00913.1,Sin4G00914.2,Sin4G00915.1,Sin4G00916.1,Sin4G00917.1,Sin4G00918.1,Sin4G00919.2,Sin4G00920.1,Sin4G00921.1,Sin4G00922.1,Sin4G00923.1,Sin4G00924.1,Sin4G00925.1,Sin4G00926.1,Sin4G00927.1,Sin4G00928.1,Sin4G00929.1,Sin4G00930.3,Sin4G00931.1,Sin4G00932.1,Sin4G00933.1,Sin4G00934.1,Sin4G00935.1,Sin4G00936.1,Sin4G00937.1,Sin4G00938.3,Sin4G00939.4,Sin4G00940.1,Sin4G00941.1,Sin4G00942.1,Sin4G00943.1,Sin4G00944.1,Sin4G00945.1,Sin4G00946.1,Sin4G00947.1,Sin4G00948.1,Sin4G00949.1,Sin4G00950.1,Sin4G00951.2,Sin4G00952.1,Sin4G00953.1,Sin4G00954.1,Sin4G00955.1 |
| SiChr.1 | 22350001 | 22490000 | 1737 | 0.72 | 0.70 | 2.47 | Sin1G01858.5,Sin1G01859.2,Sin1G01860.1,Sin1G01861.1,Sin1G01862.1,Sin1G01863.1,Sin1G01864.1,Sin1G01865.1,Sin1G01866.1,Sin1G01867.1,Sin1G01868.1,Sin1G01869.1,Sin1G01870.1 |
| SiChr.1 | 22065001 | 22165000 | 1621 | 0.76 | 0.67 | 3.97 | Sin1G01828.1,Sin1G01829.1,Sin1G01830.1,Sin1G01831.1,Sin1G01832.1,Sin1G01833.1,Sin1G01834.3,Sin1G01835.1,Sin1G01836.1,Sin1G01837.1 |
| SiChr.13 | 19390001 | 19415000 | 2 | 0.77 | 0.41 | 4.93 | Sin13G02313.1,Sin13G02314.1,Sin13G02315.1,Sin13G02316.1,Sin13G02317.1,Sin13G02318.1 |
| SiChr.3 | 24135001 | 24270000 | 1067 | 0.69 | 0.61 | 3.01 | Sin3G02379.1,Sin3G02380.1,Sin3G02381.1,Sin3G02382.1,Sin3G02383.1,Sin3G02384.1,Sin3G02385.1,Sin3G02386.1,Sin3G02387.1,Sin3G02388.1,Sin3G02389.1,Sin3G02390.1,Sin3G02391.1,Sin3G02392.1,Sin3G02393.1,Sin3G02394.1,Sin3G02395.1,Sin3G02396.1,Sin3G02397.1,Sin3G02398.1,Sin3G02399.1,Sin3G02400.1 |
| SiChr.1 | 21595001 | 21790000 | 3599 | 0.71 | 0.61 | 4.24 | Sin1G01778.1,Sin1G01779.1,Sin1G01780.1,Sin1G01781.1,Sin1G01782.1,Sin1G01783.1,Sin1G01784.1,Sin1G01785.1,Sin1G01786.1,Sin1G01787.1,Sin1G01788.4,Sin1G01789.5,Sin1G01790.1,Sin1G01791.1,Sin1G01792.1,Sin1G01793.1,Sin1G01794.1,Sin1G01795.1,Sin1G01796.1,Sin1G01797.1,Sin1G01798.1,Sin1G01799.1,Sin1G01800.1,Sin1G01801.1 |
| SiChr.9 | 21185001 | 21330000 | 844 | 0.72 | 0.59 | 3.01 | Sin9G02153.1,Sin9G02154.1,Sin9G02155.1,Sin9G02156.1,Sin9G02157.1,Sin9G02158.1,Sin9G02159.1,Sin9G02160.1,Sin9G02161.1,Sin9G02162.1,Sin9G02163.1,Sin9G02164.1,Sin9G02165.1,Sin9G02166.1,Sin9G02167.1,Sin9G02168.1,Sin9G02169.1,Sin9G02170.1,Sin9G02171.1,Sin9G02172.1,Sin9G02173.3,Sin9G02174.1,Sin9G02175.1,Sin9G02176.1 |
| SiChr.5 | 12870001 | 13100000 | 3146 | 0.72 | 0.64 | 6.05 | Sin5G00987.1,Sin5G00988.1,Sin5G00989.1,Sin5G00990.1,Sin5G00991.1,Sin5G00992.2,Sin5G00993.1,Sin5G00994.1,Sin5G00995.1,Sin5G00996.1,Sin5G00997.1,Sin5G00998.1,Sin5G00999.2,Sin5G01000.1,Sin5G01001.1,Sin5G01002.1,Sin5G01003.1,Sin5G01004.1,Sin5G01005.1 |
| SiChr.1 | 2005001 | 2125000 | 1880 | 0.72 | 0.58 | 2.85 | Sin1G00275.1,Sin1G00276.1,Sin1G00277.1,Sin1G00278.1,Sin1G00279.3,Sin1G00280.1,Sin1G00281.1,Sin1G00282.1,Sin1G00283.1,Sin1G00284.1,Sin1G00285.1,Sin1G00286.1,Sin1G00287.1,Sin1G00288.1,Sin1G00289.1,Sin1G00290.1,Sin1G00291.1,Sin1G00292.1,Sin1G00293.1,Sin1G00294.1 |
| SiChr.3 | 3865001 | 3945000 | 698 | 0.72 | 0.70 | 1.91 | Sin3G00556.1,Sin3G00557.1,Sin3G00558.1,Sin3G00559.1,Sin3G00560.2,Sin3G00561.1,Sin3G00562.1,Sin3G00563.1,Sin3G00564.1,Sin3G00565.1,Sin3G00566.1,Sin3G00567.1,Sin3G00568.1,Sin3G00569.1,Sin3G00570.1,Sin3G00571.1 |
| SiChr.1 | 1630001 | 1705000 | 1065 | 0.73 | 0.64 | 3.48 | Sin1G00231.1,Sin1G00232.1,Sin1G00233.1,Sin1G00234.1,Sin1G00235.1,Sin1G00236.1,Sin1G00237.1,Sin1G00238.1,Sin1G00239.1,Sin1G00240.1,Sin1G00241.1 |
| SiChr.7 | 16470001 | 16580000 | 2028 | 0.69 | 0.59 | 8.40 | Sin7G01241.1,Sin7G01242.2,Sin7G01243.1,Sin7G01244.2,Sin7G01245.1,Sin7G01246.1,Sin7G01247.1,Sin7G01248.1,Sin7G01249.1,Sin7G01250.1,Sin7G01251.1,Sin7G01252.1 |
| SiChr.5 | 13600001 | 13675000 | 1349 | 0.72 | 0.65 | 5.04 | Sin5G01056.1,Sin5G01057.1,Sin5G01058.1,Sin5G01059.1,Sin5G01060.1,Sin5G01061.1,Sin5G01062.1,Sin5G01063.1,Sin5G01064.1,Sin5G01065.1,Sin5G01066.1 |
| SiChr.1 | 21860001 | 21930000 | 590 | 0.73 | 0.72 | 4.18 | Sin1G01807.1,Sin1G01808.1,Sin1G01809.1,Sin1G01810.1,Sin1G01811.1,Sin1G01812.1,Sin1G01813.1 |
| SiChr.3 | 3430001 | 3485000 | 510 | 0.68 | 0.63 | 2.26 | Sin3G00496.4,Sin3G00497.1,Sin3G00498.1,Sin3G00499.1,Sin3G00500.1,Sin3G00501.1,Sin3G00502.1,Sin3G00503.2,Sin3G00504.1,Sin3G00505.1 |
| SiChr.1 | 2465001 | 2555000 | 990 | 0.71 | 0.60 | 2.70 | Sin1G00335.2,Sin1G00336.1,Sin1G00337.1,Sin1G00338.2,Sin1G00339.1,Sin1G00340.1,Sin1G00341.1,Sin1G00342.1,Sin1G00343.1 |
| SiChr.3 | 24295001 | 24340000 | 267 | 0.71 | 0.69 | 2.11 | Sin3G02405.1,Sin3G02406.1,Sin3G02407.1,Sin3G02408.1,Sin3G02409.1,Sin3G02410.1,Sin3G02411.1,Sin3G02412.1,Sin3G02413.1,Sin3G02414.1,Sin3G02415.1,Sin3G02416.1 |
| SiChr.3 | 25790001 | 25840000 | 363 | 0.68 | 0.62 | 2.96 | Sin3G02655.1,Sin3G02656.1,Sin3G02657.1,Sin3G02658.1,Sin3G02659.1,Sin3G02660.1,Sin3G02661.1,Sin3G02662.1,Sin3G02663.1,Sin3G02664.1,Sin3G02665.1,Sin3G02666.3,Sin3G02667.1,Sin3G02668.3,Sin3G02669.1,Sin3G02670.1,Sin3G02671.1 |
| SiChr.2 | 29260001 | 29360000 | 788 | 0.70 | 0.69 | 2.75 | Sin2G02835.1,Sin2G02836.1,Sin2G02837.1,Sin2G02838.1,Sin2G02839.1,Sin2G02840.1,Sin2G02841.1,Sin2G02842.1,Sin2G02843.1,Sin2G02844.1,Sin2G02845.1,Sin2G02846.1,Sin2G02847.1,Sin2G02848.1,Sin2G02849.1,Sin2G02850.1,Sin2G02851.1,Sin2G02852.1 |
| SiChr.2 | 27145001 | 27260000 | 918 | 0.70 | 0.66 | 3.32 | Sin2G02516.1,Sin2G02517.1,Sin2G02518.1,Sin2G02519.2,Sin2G02520.1,Sin2G02521.1,Sin2G02522.1,Sin2G02523.1,Sin2G02524.1,Sin2G02525.1,Sin2G02526.1,Sin2G02527.1 |
| SiChr.13 | 9070001 | 9120000 | 745 | 0.70 | 0.66 | 3.57 | Sin13G00784.1,Sin13G00785.1,Sin13G00786.1,Sin13G00787.1,Sin13G00788.1,Sin13G00789.1 |
| SiChr.9 | 675001 | 820000 | 1620 | 0.68 | 0.63 | 5.99 | Sin9G00097.2,Sin9G00098.1,Sin9G00099.1,Sin9G00100.1,Sin9G00101.1,Sin9G00102.1,Sin9G00103.1,Sin9G00104.1,Sin9G00105.2,Sin9G00106.1,Sin9G00107.1,Sin9G00108.1,Sin9G00109.1,Sin9G00110.1,Sin9G00111.1,Sin9G00112.3,Sin9G00113.1,Sin9G00114.1,Sin9G00115.1, |
| SiChr.12 | 9655001 | 9690000 | 632 | 0.68 | 0.61 | 3.38 | Sin12G00860.1,Sin12G00861.1,Sin12G00862.3,Sin12G00863.1,Sin12G00864.1,Sin12G00865.1 |
| SiChr.2 | 2485001 | 2615000 | 2525 | 0.69 | 0.66 | 3.51 | Sin2G00300.1,Sin2G00302.1,Sin2G00303.1,Sin2G00304.1,Sin2G00305.1,Sin2G00306.1,Sin2G00307.1,Sin2G00308.1,Sin2G00309.1,Sin2G00310.1,Sin2G00311.1,Sin2G00312.1,Sin2G00313.1,Sin2G00314.1,Sin2G00315.1 |
| SiChr.2 | 27895001 | 27950000 | 590 | 0.70 | 0.64 | 3.93 | Sin2G02611.1,Sin2G02612.1,Sin2G02613.1,Sin2G02614.1,Sin2G02615.1,Sin2G02616.1,Sin2G02617.1 |
| SiChr.2 | 27360001 | 27430000 | 483 | 0.70 | 0.68 | 2.71 | Sin2G02540.1,Sin2G02541.1,Sin2G02542.1,Sin2G02543.1,Sin2G02544.1,Sin2G02545.1,Sin2G02546.1,Sin2G02547.1 |
| SiChr.12 | 13795001 | 13835000 | 848 | 0.69 | 0.58 | 4.59 | Sin12G01302.1,Sin12G01303.1,Sin12G01304.1,Sin12G01305.1,Sin12G01306.1 |
| SiChr.12 | 15140001 | 15185000 | 1026 | 0.69 | 0.62 | 1.95 | Sin12G01434.1,Sin12G01435.1,Sin12G01436.1,Sin12G01437.1 |
| SiChr.3 | 745001 | 905000 | 1690 | 0.68 | 0.59 | 3.04 | Sin3G00126.1,Sin3G00127.1,Sin3G00128.1,Sin3G00129.1,Sin3G00130.1,Sin3G00131.1,Sin3G00132.1,Sin3G00133.1,Sin3G00134.1,Sin3G00135.1,Sin3G00136.2,Sin3G00137.1,Sin3G00138.1,Sin3G00139.1,Sin3G00140.1,Sin3G00141.1,Sin3G00142.1,Sin3G00143.1,Sin3G00144.1,Sin3G00145.1,Sin3G00146.1,Sin3G00147.1,Sin3G00148.1,Sin3G00149.1,Sin3G00150.1 |
| SiChr.7 | 23290001 | 23365000 | 426 | 0.69 | 0.61 | 4.89 | Sin7G02119.1,Sin7G02120.1,Sin7G02121.1,Sin7G02122.1,Sin7G02123.1,Sin7G02124.1,Sin7G02125.1,Sin7G02126.1,Sin7G02127.1,Sin7G02128.2,Sin7G02129.1,Sin7G02130.1,Sin7G02131.1,Sin7G02132.3 |
| SiChr.1 | 2590001 | 2665000 | 912 | 0.66 | 0.56 | 2.69 | Sin1G00348.1,Sin1G00349.1,Sin1G00350.1,Sin1G00351.1,Sin1G00352.1,Sin1G00353.1,Sin1G00354.1,Sin1G00355.1,Sin1G00356.1,Sin1G00357.1 |
| SiChr.7 | 22840001 | 22870000 | 151 | 0.65 | 0.60 | 3.69 | Sin7G02025.1,Sin7G02026.1,Sin7G02027.1,Sin7G02028.1,Sin7G02029.1,Sin7G02030.1,Sin7G02031.1 |
| SiChr.8 | 14205001 | 14255000 | 671 | 0.65 | 0.60 | 5.54 | Sin8G01134.1,Sin8G01135.1,Sin8G01136.1,Sin8G01137.1,Sin8G01138.1 |
| SiChr.2 | 20305001 | 20355000 | 625 | 0.69 | 0.61 | 2.18 | Sin2G01862.1,Sin2G01863.1,Sin2G01864.1,Sin2G01865.1,Sin2G01866.1,Sin2G01867.1,Sin2G01868.2 |
| SiChr.1 | 23055001 | 23090000 | 403 | 0.67 | 0.48 | 2.96 | Sin1G01951.1,Sin1G01952.1,Sin1G01953.1,Sin1G01954.1,Sin1G01955.4 |
| SiChr.1 | 21510001 | 21560000 | 455 | 0.68 | 0.67 | 2.00 | Sin1G01769.1,Sin1G01770.2,Sin1G01771.1,Sin1G01772.1,Sin1G01773.1,Sin1G01774.1 |
| SiChr.13 | 18940001 | 19030000 | 1345 | 0.68 | 0.57 | 5.84 | Sin13G02232.1,Sin13G02233.1,Sin13G02234.1,Sin13G02235.1,Sin13G02236.1,Sin13G02237.1,Sin13G02238.1,Sin13G02239.1,Sin13G02240.1,Sin13G02241.1,Sin13G02242.1,Sin13G02243.1 |
| SiChr.3 | 3970001 | 4085000 | 1925 | 0.65 | 0.56 | 4.01 | Sin3G00576.1,Sin3G00577.1,Sin3G00578.1,Sin3G00579.1,Sin3G00580.1,Sin3G00581.1,Sin3G00582.1,Sin3G00583.1,Sin3G00584.1,Sin3G00585.1,Sin3G00586.1,Sin3G00587.1,Sin3G00588.1,Sin3G00589.1,Sin3G00590.1,Sin3G00591.1,Sin3G00592.2,Sin3G00593.1 |
| SiChr.6 | 5925001 | 6065000 | 3202 | 0.68 | 0.58 | 7.39 | Sin6G00779.1,Sin6G00780.1,Sin6G00781.1,Sin6G00782.1,Sin6G00783.1,Sin6G00784.1,Sin6G00785.1,Sin6G00786.1,Sin6G00787.1,Sin6G00788.1,Sin6G00789.1,Sin6G00790.1 |
| SiChr.7 | 23365001 | 23430000 | 384 | 0.70 | 0.63 | 4.79 | Sin7G02132.3,Sin7G02133.1,Sin7G02134.1,Sin7G02135.1,Sin7G02136.1,Sin7G02137.1,Sin7G02138.1,Sin7G02139.1,Sin7G02140.1,Sin7G02141.1,Sin7G02142.1 |
| SiChr.5 | 12550001 | 12650000 | 1296 | 0.68 | 0.60 | 4.55 | Sin5G00953.1,Sin5G00954.2,Sin5G00955.1,Sin5G00956.1,Sin5G00957.1,Sin5G00958.1,Sin5G00959.1,Sin5G00960.1,Sin5G00961.1,Sin5G00962.1,Sin5G00963.1,Sin5G00964.1 |
| SiChr.13 | 17325001 | 17390000 | 356 | 0.67 | 0.51 | 4.19 | Sin13G01975.1,Sin13G01976.1,Sin13G01977.1,Sin13G01978.1,Sin13G01979.1,Sin13G01980.1,Sin13G01981.1,Sin13G01982.1,Sin13G01983.1,Sin13G01984.1,Sin13G01985.1,Sin13G01986.2 |
| SiChr.12 | 12855001 | 12915000 | 451 | 0.70 | 0.67 | 1.98 | Sin12G01220.1,Sin12G01221.1,Sin12G01222.1,Sin12G01223.7 |
| SiChr.1 | 16700001 | 16735000 | 230 | 0.67 | 0.51 | 3.35 | Sin1G01111.1,Sin1G01112.4,Sin1G01113.1,Sin1G01114.1,Sin1G01115.1,Sin1G01116.1,Sin1G01117.1,Sin1G01118.1 |
| SiChr.6 | 16630001 | 16670000 | 267 | 0.68 | 0.59 | 3.09 | Sin6G01381.1,Sin6G01382.1,Sin6G01383.1 |
| SiChr.2 | 26740001 | 26790000 | 752 | 0.67 | 0.53 | 4.87 | Sin2G02481.1,Sin2G02482.1,Sin2G02483.1,Sin2G02484.1 |
| SiChr.1 | 19670001 | 19760000 | 1433 | 0.66 | 0.63 | 3.09 | Sin1G01557.1,Sin1G01558.2,Sin1G01559.1,Sin1G01560.1,Sin1G01561.1,Sin1G01562.1,Sin1G01563.1,Sin1G01564.1,Sin1G01565.1,Sin1G01566.1,Sin1G01567.3 |
| SiChr.2 | 27520001 | 27580000 | 664 | 0.65 | 0.56 | 2.76 | Sin2G02562.1,Sin2G02563.2,Sin2G02564.1,Sin2G02565.1,Sin2G02566.1,Sin2G02567.1,Sin2G02568.2 |
| SiChr.1 | 1165001 | 1225000 | 1295 | 0.67 | 0.45 | 2.27 | Sin1G00184.1,Sin1G00185.1,Sin1G00186.1 |
| SiChr.3 | 590001 | 620000 | 282 | 0.67 | 0.52 | 2.53 | Sin3G00101.1,Sin3G00102.1,Sin3G00103.1,Sin3G00104.1,Sin3G00105.1,Sin3G00106.1,Sin3G00107.1,Sin3G00108.1 |
| SiChr.1 | 1755001 | 1785000 | 694 | 0.67 | 0.64 | 2.04 | Sin1G00248.1,Sin1G00249.1,Sin1G00250.1,Sin1G00251.2 |
| SiChr.1 | 17775001 | 17830000 | 332 | 0.69 | 0.67 | 3.24 | Sin1G01292.2,Sin1G01293.1,Sin1G01294.1,Sin1G01295.1,Sin1G01296.1,Sin1G01297.1,Sin1G01298.1,Sin1G01299.1,Sin1G01300.1 |
| SiChr.6 | 5820001 | 5915000 | 1368 | 0.68 | 0.58 | 6.49 | Sin6G00766.1,Sin6G00767.1,Sin6G00768.1,Sin6G00769.1,Sin6G00770.1,Sin6G00771.1,Sin6G00772.2,Sin6G00773.1,Sin6G00774.1,Sin6G00775.1,Sin6G00776.1,Sin6G00777.1 |
| SiChr.6 | 6065001 | 6150000 | 1784 | 0.68 | 0.62 | 4.50 | Sin6G00791.1,Sin6G00792.1,Sin6G00793.1,Sin6G00794.1,Sin6G00795.2,Sin6G00796.1,Sin6G00797.1,Sin6G00798.1,Sin6G00799.1,Sin6G00800.1,Sin6G00801.1,Sin6G00802.1 |
| SiChr.1 | 18985001 | 19010000 | 157 | 0.66 | 0.57 | 2.77 | Sin1G01470.1,Sin1G01471.1,Sin1G01472.1,Sin1G01473.1 |
| SiChr.1 | 17835001 | 17860000 | 242 | 0.68 | 0.55 | 2.27 | Sin1G01301.1,Sin1G01302.1,Sin1G01303.1,Sin1G01304.1,Sin1G01305.1,Sin1G01306.1 |
| SiChr.1 | 16780001 | 16835000 | 472 | 0.66 | 0.64 | 2.25 | Sin1G01125.1,Sin1G01126.1,Sin1G01127.1,Sin1G01128.1,Sin1G01129.1,Sin1G01130.1,Sin1G01131.1,Sin1G01132.1,Sin1G01133.1,Sin1G01134.1,Sin1G01135.1,Sin1G01136.1 |
| SiChr.3 | 25450001 | 25505000 | 486 | 0.67 | 0.62 | 5.04 | Sin3G02611.1,Sin3G02612.1,Sin3G02613.1,Sin3G02614.1,Sin3G02615.1,Sin3G02616.1,Sin3G02617.1 |
| SiChr.6 | 6175001 | 6250000 | 1307 | 0.68 | 0.64 | 2.48 | Sin6G00808.1,Sin6G00809.2,Sin6G00810.1,Sin6G00811.1,Sin6G00812.1,Sin6G00813.1 |
| SiChr.2 | 2405001 | 2475000 | 1693 | 0.66 | 0.63 | 2.07 | Sin2G00297.1,Sin2G00298.1,Sin2G00299.1 |
| SiChr.3 | 20515001 | 20565000 | 437 | 0.65 | 0.48 | 1.88 | Sin3G01935.2,Sin3G01936.1,Sin3G01937.1,Sin3G01938.1,Sin3G01939.1,Sin3G01940.1 |
| SiChr.3 | 20815001 | 20870000 | 1176 | 0.66 | 0.51 | 2.69 | Sin3G01977.1,Sin3G01978.1,Sin3G01979.1,Sin3G01980.1,Sin3G01981.1,Sin3G01982.2 |
| SiChr.8 | 17775001 | 17810000 | 63 | 0.65 | 0.63 | 1.78 | Sin8G01529.1,Sin8G01530.1,Sin8G01531.1,Sin8G01532.1 |
| SiChr.6 | 15835001 | 15865000 | 288 | 0.66 | 0.61 | 6.35 | Sin6G01299.1,Sin6G01300.1,Sin6G01301.1,Sin6G01302.5,Sin6G01303.1 |
| SiChr.10 | 13925001 | 13995000 | 650 | 0.68 | 0.64 | 3.84 | Sin10G01280.1,Sin10G01281.1,Sin10G01282.1,Sin10G01283.1,Sin10G01284.1,Sin10G01285.1,Sin10G01286.1,Sin10G01287.1,Sin10G01288.1 |
| SiChr.1 | 20085001 | 20155000 | 377 | 0.66 | 0.59 | 2.34 | Sin1G01603.1,Sin1G01604.4,Sin1G01605.1,Sin1G01606.1,Sin1G01607.1,Sin1G01608.1 |
| SiChr.5 | 16045001 | 16145000 | 1089 | 0.66 | 0.56 | 3.40 | Sin5G01387.1,Sin5G01388.1,Sin5G01389.1,Sin5G01390.1,Sin5G01391.1,Sin5G01392.1,Sin5G01393.1,Sin5G01394.1,Sin5G01395.1,Sin5G01396.1,Sin5G01397.1,Sin5G01398.1,Sin5G01399.1,Sin5G01400.13,Sin5G01401.1,Sin5G01402.1 |
| SiChr.6 | 6255001 | 6305000 | 636 | 0.63 | 0.56 | 2.30 | Sin6G00814.1,Sin6G00815.1,Sin6G00816.1,Sin6G00817.1,Sin6G00818.1 |
| SiChr.5 | 12190001 | 12270000 | 1648 | 0.63 | 0.49 | 2.58 | Sin5G00923.1,Sin5G00924.1,Sin5G00925.5,Sin5G00926.1,Sin5G00927.1 |
| SiChr.6 | 18950001 | 19055000 | 1041 | 0.64 | 0.61 | 3.66 | Sin6G01611.2,Sin6G01612.1,Sin6G01613.1,Sin6G01614.4,Sin6G01615.1,Sin6G01616.1,Sin6G01617.4 |
| SiChr.3 | 20565001 | 20635000 | 961 | 0.63 | 0.47 | 3.39 | Sin3G01940.1,Sin3G01941.1,Sin3G01942.1,Sin3G01943.1,Sin3G01944.1,Sin3G01945.1,Sin3G01946.1,Sin3G01947.1,Sin3G01948.1 |
| SiChr.3 | 6425001 | 6465000 | 588 | 0.65 | 0.52 | 3.77 | Sin3G00889.1,Sin3G00890.1,Sin3G00891.1,Sin3G00892.1,Sin3G00893.1,Sin3G00894.1,Sin3G00895.1 |
| SiChr.10 | 13610001 | 13755000 | 1338 | 0.65 | 0.61 | 4.73 | Sin10G01228.3,Sin10G01229.1,Sin10G01230.1,Sin10G01231.1,Sin10G01232.2,Sin10G01233.1,Sin10G01234.1,Sin10G01235.1,Sin10G01236.1,Sin10G01237.1,Sin10G01238.1,Sin10G01239.1,Sin10G01240.1,Sin10G01241.1,Sin10G01242.1,Sin10G01243.1,Sin10G01244.1,Sin10G01245.1,Sin10G01246.1,Sin10G01247.1,Sin10G01248.1,Sin10G01249.1,Sin10G01250.1,Sin10G01251.1,Sin10G01252.1,Sin10G01253.1,Sin10G01254.1,Sin10G01255.1,Sin10G01256.1,Sin10G01257.1 |
| SiChr.13 | 19255001 | 19285000 | 206 | 0.65 | 0.48 | 3.24 | Sin13G02289.1,Sin13G02290.1,Sin13G02291.1,Sin13G02292.1,Sin13G02293.1,Sin13G02294.2 |
| SiChr.13 | 8335001 | 8375000 | 581 | 0.65 | 0.47 | 10.41 | Sin13G00711.1,Sin13G00712.2,Sin13G00713.1,Sin13G00714.1 |
| SiChr.5 | 11905001 | 11970000 | 1996 | 0.64 | 0.41 | 3.58 | Sin5G00898.1,Sin5G00901.1,Sin5G00902.1,Sin5G00903.1,Sin5G00904.1,Sin5G00905.1,Sin5G00906.1,Sin5G00907.1,Sin5G00908.1 |
| SiChr.2 | 26800001 | 26820000 | 347 | 0.67 | 0.54 | 2.03 | Sin2G02486.1,Sin2G02487.1 |
| SiChr.4 | 22440001 | 22490000 | 735 | 0.66 | 0.54 | 4.62 | Sin4G02273.1,Sin4G02274.1,Sin4G02275.1,Sin4G02276.1 |
| SiChr.5 | 19605001 | 19650000 | 517 | 0.65 | 0.50 | 4.39 | Sin5G01882.3,Sin5G01883.1,Sin5G01884.4,Sin5G01885.1,Sin5G01886.1,Sin5G01887.1,Sin5G01888.1 |
| SiChr.8 | 18610001 | 18645000 | 186 | 0.67 | 0.63 | 2.12 | Sin8G01655.3 |
| SiChr.5 | 12415001 | 12470000 | 2662 | 0.64 | 0.54 | 2.50 | Sin5G00943.1,Sin5G00944.1,Sin5G00945.1 |
| SiChr.8 | 18550001 | 18595000 | 471 | 0.65 | 0.63 | 3.07 | Sin8G01652.1,Sin8G01653.1,Sin8G01654.1 |
| SiChr.1 | 22540001 | 22565000 | 270 | 0.65 | 0.62 | 2.60 | Sin1G01879.1,Sin1G01880.1,Sin1G01881.1,Sin1G01882.1 |
| SiChr.8 | 1340001 | 1380000 | 965 | 0.64 | 0.59 | 6.35 | Sin8G00135.1,Sin8G00136.1,Sin8G00137.1,Sin8G00138.1 |
| SiChr.9 | 3645001 | 3715000 | 1672 | 0.63 | 0.51 | 5.05 | Sin9G00485.1,Sin9G00486.1,Sin9G00487.1,Sin9G00488.1,Sin9G00489.1 |
| SiChr.2 | 23320001 | 23425000 | 1540 | 0.64 | 0.41 | 3.08 | Sin2G02141.1,Sin2G02142.1,Sin2G02143.1,Sin2G02144.1,Sin2G02145.1,Sin2G02146.1,Sin2G02147.1,Sin2G02148.1,Sin2G02149.1,Sin2G02150.1,Sin2G02151.1,Sin2G02152.1 |
| SiChr.8 | 17690001 | 17750000 | 773 | 0.64 | 0.50 | 3.90 | Sin8G01515.1,Sin8G01516.1,Sin8G01517.1,Sin8G01518.1,Sin8G01519.1,Sin8G01520.1,Sin8G01521.1,Sin8G01522.1,Sin8G01523.1 |
| SiChr.9 | 17905001 | 17960000 | 410 | 0.66 | 0.65 | 2.10 | Sin9G01814.1,Sin9G01815.2,Sin9G01816.1,Sin9G01817.1,Sin9G01818.1,Sin9G01819.2,Sin9G01820.1 |
| SiChr.2 | 24555001 | 24595000 | 605 | 0.64 | 0.58 | 2.32 | Sin2G02222.1,Sin2G02223.1,Sin2G02224.1 |
| SiChr.3 | 1300001 | 1350000 | 376 | 0.62 | 0.51 | 2.60 | Sin3G00226.1,Sin3G00227.3,Sin3G00228.1,Sin3G00229.1,Sin3G00230.1,Sin3G00231.3,Sin3G00232.1 |
| SiChr.13 | 9460001 | 9500000 | 839 | 0.66 | 0.64 | 3.13 | Sin13G00812.1,Sin13G00813.1,Sin13G00814.1,Sin13G00815.1,Sin13G00816.1,Sin13G00817.1 |
| SiChr.8 | 18350001 | 18425000 | 839 | 0.64 | 0.62 | 3.51 | Sin8G01618.1,Sin8G01619.1,Sin8G01620.1,Sin8G01621.1,Sin8G01622.1,Sin8G01623.1,Sin8G01624.1,Sin8G01625.1,Sin8G01626.1,Sin8G01627.1,Sin8G01628.1,Sin8G01629.1 |
| SiChr.2 | 23270001 | 23305000 | 620 | 0.63 | 0.54 | 2.25 | Sin2G02137.1,Sin2G02138.1,Sin2G02139.1,Sin2G02140.1 |
| SiChr.6 | 18105001 | 18180000 | 823 | 0.64 | 0.61 | 3.75 | Sin6G01516.1,Sin6G01517.1,Sin6G01518.1,Sin6G01519.1,Sin6G01520.2,Sin6G01521.1,Sin6G01522.1,Sin6G01523.1 |
| SiChr.6 | 12695001 | 12725000 | 542 | 0.64 | 0.40 | 2.88 | Sin6G01002.1 |
| SiChr.2 | 26495001 | 26555000 | 1236 | 0.63 | 0.51 | 1.96 | Sin2G02460.1,Sin2G02461.1,Sin2G02462.1,Sin2G02463.1,Sin2G02464.1,Sin2G02465.1,Sin2G02466.5,Sin2G02467.1,Sin2G02468.1,Sin2G02469.1 |
| SiChr.7 | 22255001 | 22305000 | 350 | 0.65 | 0.63 | 5.02 | Sin7G01923.1,Sin7G01924.1,Sin7G01925.1,Sin7G01926.1,Sin7G01927.1,Sin7G01928.1,Sin7G01929.1,Sin7G01930.1 |
| SiChr.2 | 26345001 | 26365000 | 6 | 0.65 | 0.24 | 3.44 | Sin2G02435.1,Sin2G02436.1,Sin2G02437.1,Sin2G02438.1 |
| SiChr.2 | 2770001 | 2810000 | 975 | 0.63 | 0.50 | 2.47 | Sin2G00332.1,Sin2G00333.1,Sin2G00334.1,Sin2G00335.1,Sin2G00336.1,Sin2G00337.1 |
| SiChr.6 | 520001 | 570000 | 422 | 0.65 | 0.64 | 1.93 | Sin6G00091.1,Sin6G00092.1,Sin6G00093.1,Sin6G00094.1,Sin6G00095.1,Sin6G00096.1,Sin6G00097.1,Sin6G00098.1,Sin6G00099.1,Sin6G00100.1,Sin6G00101.1,Sin6G00102.1,Sin6G00103.1, |
| SiChr.5 | 24420001 | 24455000 | 297 | 0.64 | 0.63 | 2.14 | Sin5G02531.1,Sin5G02532.1,Sin5G02533.1,Sin5G02534.2,Sin5G02535.1 |
| SiChr.13 | 8875001 | 8910000 | 457 | 0.65 | 0.62 | 2.19 | Sin13G00765.1,Sin13G00766.1,Sin13G00767.8,Sin13G00768.1 |
| SiChr.4 | 7720001 | 7760000 | 649 | 0.65 | 0.56 | 6.02 | Sin4G01044.4,Sin4G01045.3 |
| SiChr.6 | 585001 | 645000 | 400 | 0.63 | 0.61 | 2.33 | Sin6G00107.1,Sin6G00108.1,Sin6G00109.1,Sin6G00110.1,Sin6G00111.4,Sin6G00112.1,Sin6G00113.1,Sin6G00114.1,Sin6G00115.1,Sin6G00116.1,Sin6G00117.1,Sin6G00118.1,Sin6G00119.1,Sin6G00120.1 |
| SiChr.9 | 15270001 | 15320000 | 1038 | 0.64 | 0.52 | 5.85 | Sin9G01611.2,Sin9G01612.1,Sin9G01613.1,Sin9G01614.1 |
| SiChr.1 | 785001 | 835000 | 690 | 0.64 | 0.48 | 3.86 | Sin1G00132.1,Sin1G00133.1,Sin1G00134.2,Sin1G00135.1,Sin1G00136.1,Sin1G00137.1,Sin1G00138.1,Sin1G00139.1,Sin1G00140.1,Sin1G00141.1 |
| SiChr.2 | 24940001 | 24980000 | 606 | 0.62 | 0.47 | 4.26 | Sin2G02267.6,Sin2G02268.1,Sin2G02269.1,Sin2G02270.1,Sin2G02271.4 |
| SiChr.5 | 14435001 | 14485000 | 508 | 0.63 | 0.55 | 2.56 | Sin5G01167.1,Sin5G01168.1,Sin5G01169.4,Sin5G01170.1,Sin5G01171.1,Sin5G01172.1,Sin5G01173.2,Sin5G01174.1 |
| SiChr.3 | 20455001 | 20515000 | 972 | 0.62 | 0.51 | 2.28 | Sin3G01925.1,Sin3G01926.1,Sin3G01927.1,Sin3G01928.1,Sin3G01929.1,Sin3G01930.1,Sin3G01931.1,Sin3G01932.1,Sin3G01933.4,Sin3G01934.1 |
| SiChr.3 | 20955001 | 21015000 | 1039 | 0.63 | 0.47 | 2.84 | Sin3G01996.6,Sin3G01997.1,Sin3G01998.1,Sin3G01999.2,Sin3G02000.2,Sin3G02001.1,Sin3G02002.1,Sin3G02003.1,Sin3G02004.1,Sin3G02005.1,Sin3G02006.1,Sin3G02007.1,Sin3G02008.1 |
| SiChr.13 | 9145001 | 9180000 | 535 | 0.63 | 0.55 | 2.45 | Sin13G00792.1,Sin13G00793.1,Sin13G00794.1 |
| SiChr.3 | 4760001 | 4815000 | 538 | 0.64 | 0.62 | 2.57 | Sin3G00697.1,Sin3G00698.1,Sin3G00699.1,Sin3G00700.1,Sin3G00701.1,Sin3G00702.1,Sin3G00703.1,Sin3G00704.1,Sin3G00705.1,Sin3G00706.1 |
| SiChr.4 | 8750001 | 8800000 | 841 | 0.63 | 0.48 | 2.55 | Sin4G01189.1,Sin4G01190.1,Sin4G01191.1,Sin4G01192.1 |
| SiChr.4 | 6245001 | 6305000 | 806 | 0.62 | 0.47 | 2.73 | Sin4G00813.1,Sin4G00814.1,Sin4G00815.1,Sin4G00816.1,Sin4G00817.1,Sin4G00818.1,Sin4G00819.1,Sin4G00820.1,Sin4G00821.1,Sin4G00822.2,Sin4G00823.1,Sin4G00824.1 |
| SiChr.1 | 19060001 | 19115000 | 768 | 0.62 | 0.60 | 4.46 | Sin1G01480.1,Sin1G01481.1,Sin1G01482.1,Sin1G01483.1,Sin1G01484.2,Sin1G01485.1 |
| SiChr.1 | 19855001 | 19895000 | 686 | 0.63 | 0.52 | 2.93 | Sin1G01575.1,Sin1G01576.1,Sin1G01577.2,Sin1G01578.1,Sin1G01579.1 |
| SiChr.7 | 20775001 | 20815000 | 547 | 0.63 | 0.61 | 3.86 | Sin7G01714.1,Sin7G01715.1,Sin7G01716.1,Sin7G01717.1 |
| SiChr.4 | 8700001 | 8750000 | 1412 | 0.63 | 0.47 | 2.36 | Sin4G01184.1,Sin4G01185.1,Sin4G01186.1,Sin4G01187.1,Sin4G01188.1 |
| SiChr.1 | 875001 | 915000 | 585 | 0.63 | 0.47 | 3.93 | Sin1G00149.1,Sin1G00150.1,Sin1G00151.1,Sin1G00152.1 |
| SiChr.13 | 8245001 | 8330000 | 1927 | 0.61 | 0.44 | 4.20 | Sin13G00705.1,Sin13G00706.1,Sin13G00707.4,Sin13G00708.1,Sin13G00709.1,Sin13G00710.1 |
| SiChr.3 | 23960001 | 24020000 | 915 | 0.62 | 0.45 | 5.45 | Sin3G02362.1,Sin3G02363.1,Sin3G02364.1,Sin3G02365.1,Sin3G02366.1,Sin3G02367.1,Sin3G02368.2,Sin3G02369.1 |
| SiChr.6 | 14590001 | 14690000 | 1812 | 0.62 | 0.56 | 4.45 | Sin6G01151.1,Sin6G01152.1,Sin6G01153.1,Sin6G01154.2,Sin6G01155.1,Sin6G01156.1,Sin6G01157.1,Sin6G01158.1,Sin6G01159.1,Sin6G01160.1,Sin6G01161.1 |
| SiChr.11 | 13985001 | 14025000 | 391 | 0.63 | 0.52 | 7.08 | Sin11G01306.1,Sin11G01307.1,Sin11G01308.1,Sin11G01309.1,Sin11G01310.1,Sin11G01311.1,Sin11G01312.1,Sin11G01313.1,Sin11G01314.2 |
| SiChr.6 | 5770001 | 5810000 | 752 | 0.63 | 0.55 | 7.00 | Sin6G00762.1,Sin6G00763.1,Sin6G00764.1,Sin6G00765.1 |
| SiChr.3 | 25715001 | 25745000 | 227 | 0.62 | 0.52 | 3.08 | Sin3G02647.1,Sin3G02648.1,Sin3G02649.1,Sin3G02650.1,Sin3G02651.1,Sin3G02652.1,Sin3G02653.1 |
| SiChr.2 | 22245001 | 22275000 | 426 | 0.64 | 0.63 | 2.61 | Sin2G02045.1,Sin2G02046.1,Sin2G02047.2,Sin2G02048.1,Sin2G02049.1 |
| SiChr.2 | 24475001 | 24520000 | 379 | 0.63 | 0.53 | 2.46 | Sin2G02216.1,Sin2G02217.1,Sin2G02218.1,Sin2G02219.1,Sin2G02220.1 |
| SiChr.13 | 11175001 | 11220000 | 928 | 0.63 | 0.58 | 2.19 | Sin13G00978.1,Sin13G00980.1,Sin13G00981.1,Sin13G00982.1,Sin13G00983.1 |
| SiChr.10 | 13895001 | 13920000 | 80 | 0.62 | 0.51 | 2.78 | Sin10G01277.1,Sin10G01278.1,Sin10G01279.1 |
| SiChr.4 | 8020001 | 8085000 | 463 | 0.63 | 0.61 | 2.27 | Sin4G01082.1,Sin4G01083.1,Sin4G01084.1,Sin4G01085.1,Sin4G01086.1,Sin4G01087.1,Sin4G01088.1,Sin4G01089.1,Sin4G01090.2,Sin4G01091.1,Sin4G01092.1,Sin4G01093.2,Sin4G01094.1,Sin4G01095.1,Sin4G01096.1,Sin4G01097.1,Sin4G01098.1,Sin4G01099.2 |
| SiChr.3 | 4085001 | 4120000 | 549 | 0.62 | 0.54 | 2.31 | Sin3G00593.1,Sin3G00594.1,Sin3G00595.1,Sin3G00596.3,Sin3G00597.1,Sin3G00598.1 |
| SiChr.4 | 21675001 | 21720000 | 607 | 0.63 | 0.62 | 3.60 | Sin4G02207.1,Sin4G02208.1,Sin4G02209.1,Sin4G02210.2 |
| SiChr.9 | 17695001 | 17735000 | 594 | 0.62 | 0.61 | 2.07 | Sin9G01797.1,Sin9G01798.3,Sin9G01799.1,Sin9G01800.1 |
| SiChr.8 | 14155001 | 14180000 | 485 | 0.63 | 0.57 | 5.55 | Sin8G01126.1,Sin8G01127.1,Sin8G01128.1,Sin8G01129.1,Sin8G01130.1 |
| SiChr.10 | 885001 | 925000 | 874 | 0.62 | 0.57 | 3.50 | Sin10G00114.1 |
| SiChr.10 | 15520001 | 15550000 | 216 | 0.62 | 0.60 | 5.10 | Sin10G01528.1,Sin10G01529.1,Sin10G01530.1,Sin10G01531.1,Sin10G01532.1 |
| SiChr.2 | 21810001 | 21840000 | 561 | 0.62 | 0.55 | 2.47 | Sin2G02010.1,Sin2G02011.1,Sin2G02012.1,Sin2G02013.1 |
| SiChr.1 | 1455001 | 1485000 | 833 | 0.62 | 0.41 | 3.32 | Sin1G00212.1,Sin1G00213.1 |
| SiChr.4 | 24885001 | 24920000 | 497 | 0.62 | 0.43 | 2.99 | Sin4G02543.1,Sin4G02544.1,Sin4G02545.1 |
| SiChr.2 | 2685001 | 2745000 | 1673 | 0.61 | 0.52 | 3.09 | Sin2G00325.1,Sin2G00326.1,Sin2G00327.1,Sin2G00328.1,Sin2G00329.1,Sin2G00330.1 |
| SiChr.1 | 16500001 | 16550000 | 440 | 0.62 | 0.61 | 2.47 | Sin1G01087.1,Sin1G01088.1,Sin1G01089.1,Sin1G01090.1 |
| SiChr.8 | 2375001 | 2420000 | 933 | 0.62 | 0.51 | 4.53 | Sin8G00278.1,Sin8G00279.1,Sin8G00280.1,Sin8G00281.1,Sin8G00282.1,Sin8G00283.1,Sin8G00284.1,Sin8G00285.1,Sin8G00286.2 |
| SiChr.5 | 21370001 | 21400000 | 182 | 0.63 | 0.60 | 1.69 | Sin5G02083.1,Sin5G02084.1,Sin5G02085.1,Sin5G02086.1,Sin5G02087.1 |
| SiChr.5 | 12150001 | 12170000 | 551 | 0.63 | 0.48 | 2.16 | Sin5G00920.1,Sin5G00921.1,Sin5G00922.1 |
| SiChr.5 | 11810001 | 11835000 | 760 | 0.63 | 0.36 | 2.60 | Sin5G00893.1 |
| SiChr.1 | 24970001 | 25045000 | 427 | 0.62 | 0.57 | 1.94 | Sin1G02145.1,Sin1G02146.1,Sin1G02147.1,Sin1G02148.1,Sin1G02149.1,Sin1G02150.1,Sin1G02151.1,Sin1G02152.1,Sin1G02153.1 |
| SiChr.7 | 20425001 | 20470000 | 719 | 0.62 | 0.46 | 2.44 | Sin7G01689.1,Sin7G01690.1,Sin7G01691.1,Sin7G01692.1,Sin7G01693.1,Sin7G01694.1,Sin7G01695.2,Sin7G01696.1 |
| SiChr.5 | 21455001 | 21525000 | 491 | 0.61 | 0.59 | 1.90 | Sin5G02098.1,Sin5G02099.1,Sin5G02100.1,Sin5G02101.1,Sin5G02102.1,Sin5G02103.1,Sin5G02104.1,Sin5G02105.1,Sin5G02106.1,Sin5G02107.1,Sin5G02108.3 |
| SiChr.10 | 8485001 | 8520000 | 417 | 0.61 | 0.53 | 7.92 | Sin10G00656.1,Sin10G00657.1,Sin10G00658.1 |
| SiChr.8 | 18650001 | 18680000 | 482 | 0.61 | 0.53 | 1.80 | Sin8G01656.2,Sin8G01657.3,Sin8G01658.2 |
| SiChr.4 | 8195001 | 8230000 | 192 | 0.61 | 0.58 | 2.02 | Sin4G01118.1,Sin4G01119.1,Sin4G01120.1,Sin4G01121.1,Sin4G01122.1,Sin4G01123.1,Sin4G01124.1 |
| SiChr.5 | 14345001 | 14400000 | 793 | 0.61 | 0.40 | 4.18 | Sin5G01155.1,Sin5G01156.1,Sin5G01157.1,Sin5G01158.1,Sin5G01159.1,Sin5G01160.1,Sin5G01161.1,Sin5G01162.3 |
| SiChr.2 | 23130001 | 23155000 | 180 | 0.62 | 0.51 | 2.45 | Sin2G02120.1 |
| SiChr.10 | 815001 | 850000 | 1110 | 0.61 | 0.52 | 3.10 | Sin10G00109.1,Sin10G00110.1,Sin10G00111.1,Sin10G00112.1 |
| SiChr.10 | 13765001 | 13800000 | 480 | 0.61 | 0.48 | 6.30 | Sin10G01259.1,Sin10G01260.1,Sin10G01261.1,Sin10G01262.1,Sin10G01263.1 |
| SiChr.2 | 23695001 | 23720000 | 501 | 0.62 | 0.51 | 2.95 | Sin2G02175.1,Sin2G02176.1 |
| SiChr.2 | 26710001 | 26735000 | 98 | 0.61 | 0.44 | 1.75 | Sin2G02480.1,Sin2G02481.1 |
| SiChr.10 | 13445001 | 13495000 | 670 | 0.62 | 0.60 | 4.70 | Sin10G01202.1,Sin10G01203.1,Sin10G01204.1,Sin10G01205.1,Sin10G01206.1,Sin10G01207.1,Sin10G01208.1,Sin10G01209.1,Sin10G01210.1 |
| SiChr.1 | 415001 | 440000 | 125 | 0.62 | 0.60 | 2.63 | Sin1G00061.1,Sin1G00062.4,Sin1G00063.2 |
| SiChr.2 | 24385001 | 24410000 | 247 | 0.61 | 0.55 | 2.67 | Sin2G02209.2,Sin2G02210.1 |
| SiChr.2 | 5750001 | 5800000 | 777 | 0.61 | 0.50 | 2.61 | Sin2G00672.1,Sin2G00673.1,Sin2G00674.2,Sin2G00675.1,Sin2G00676.1,Sin2G00677.1,Sin2G00678.1,Sin2G00679.1,Sin2G00680.1,Sin2G00681.1,Sin2G00682.1,Sin2G00683.1 |
| SiChr.13 | 15855001 | 15880000 | 245 | 0.61 | 0.49 | 6.32 | Sin13G01709.1,Sin13G01710.1,Sin13G01711.1 |
| SiChr.9 | 3445001 | 3465000 | 213 | 0.61 | 0.59 | 2.61 | Sin9G00464.1,Sin9G00465.2,Sin9G00466.1,Sin9G00467.1 |
| SiChr.3 | 20655001 | 20710000 | 886 | 0.60 | 0.55 | 2.33 | Sin3G01952.1,Sin3G01953.1,Sin3G01954.1,Sin3G01955.6,Sin3G01956.1,Sin3G01957.1,Sin3G01958.1,Sin3G01959.4,Sin3G01960.1,Sin3G01961.1 |
| SiChr.1 | 2190001 | 2240000 | 746 | 0.60 | 0.55 | 4.29 | Sin1G00303.1,Sin1G00304.1,Sin1G00305.1,Sin1G00306.1,Sin1G00307.1,Sin1G00308.1,Sin1G00309.1 |
| SiChr.8 | 12525001 | 12550000 | 513 | 0.61 | 0.58 | 5.17 | Sin8G00993.1 |
| SiChr.1 | 22500001 | 22520000 | 93 | 0.61 | 0.58 | 2.87 | Sin1G01872.1,Sin1G01873.1,Sin1G01874.4,Sin1G01875.1,Sin1G01876.1 |
| SiChr.13 | 15690001 | 15710000 | 284 | 0.61 | 0.47 | 3.75 | Sin13G01669.1,Sin13G01670.1,Sin13G01671.1,Sin13G01672.1 |
| SiChr.5 | 21410001 | 21445000 | 497 | 0.61 | 0.59 | 2.41 | Sin5G02090.1,Sin5G02091.1,Sin5G02092.1,Sin5G02093.1,Sin5G02094.1,Sin5G02095.1,Sin5G02096.2,Sin5G02097.1 |
| SiChr.3 | 2075001 | 2095000 | 262 | 0.61 | 0.54 | 3.23 | Sin3G00320.1,Sin3G00321.1,Sin3G00322.1 |
| SiChr.9 | 15335001 | 15365000 | 400 | 0.60 | 0.53 | 5.07 | Sin9G01617.1,Sin9G01618.1,Sin9G01619.2,Sin9G01620.1,Sin9G01621.1 |
| SiChr.2 | 24440001 | 24460000 | 404 | 0.60 | 0.50 | 2.12 | Sin2G02213.1,Sin2G02214.1,Sin2G02215.1 |
| SiChr.4 | 8670001 | 8690000 | 550 | 0.60 | 0.42 | 1.71 | Sin4G01181.1,Sin4G01182.1,Sin4G01183.3 |
| SiChr.8 | 12355001 | 12375000 | 303 | 0.60 | 0.59 | 2.20 | Sin8G00975.2,Sin8G00976.1 |
| SiChr.3 | 7615001 | 7635000 | 224 | 0.60 | 0.48 | 2.27 | Sin3G01068.1,Sin3G01069.1,Sin3G01070.1 |
| SiChr.12 | 13525001 | 13560000 | 393 | 0.60 | 0.57 | 1.68 | Sin12G01279.1,Sin12G01280.1,Sin12G01281.1 |
| SiChr.6 | 6450001 | 6470000 | 92 | 0.60 | 0.57 | 3.11 | Sin6G00832.1 |
| SiChr.13 | 11250001 | 11270000 | 251 | 0.60 | 0.59 | 2.66 | Sin13G00988.1,Sin13G00989.1,Sin13G00990.1 |
| SiChr.3 | 3395001 | 3415000 | 344 | 0.60 | 0.45 | 3.41 | Sin3G00491.1,Sin3G00492.1,Sin3G00493.1 |

| **Table S19 Statistics of flowering date related genes subject to selection.** | | | | | | | | | |
| --- | --- | --- | --- | --- | --- | --- | --- | --- | --- |
| Chrom | Start | End | Var_number | Mean_WEIGHTED_FST | Mean_FST | xpclr_norm | Gene ID | Homology ID | Homology annotation |
| SiChr.12 | 11265001 | 11770000 | 10505 | 0.80 | 0.75 | 5.53 | Sin12G01088.1 | AT1G78440.1 | Symbols: GA2OX1, ATGA2OX1 |
| SiChr.12 | 11265001 | 11770000 | 10505 | 0.80 | 0.75 | 5.53 | Sin12G01070.1 | AT1G71800.1 | Symbols: CSTF64 |
| SiChr.3 | 395001 | 565000 | 1536 | 0.83 | 0.76 | 6.76 | Sin3G00092.1 | AT5G48560.1 | Symbols: CIB2 |
| SiChr.3 | 5001 | 390000 | 1965 | 0.82 | 0.76 | 4.06 | Sin3G00044.1 | AT1G55250.5 | Symbols: HUB2 |
| SiChr.3 | 5001 | 390000 | 1965 | 0.82 | 0.76 | 4.06 | Sin3G00060.1 | AT2G30140.2 | Symbols: UGT87A2 |
| SiChr.4 | 7275001 | 7425000 | 1629 | 0.79 | 0.71 | 7.25 | Sin4G00982.1 | AT4G32980.1 | Symbols: ATH1 |
| SiChr.4 | 7160001 | 7265000 | 452 | 0.74 | 0.62 | 4.56 | Sin4G00967.2 | AT2G25930.1 | Symbols: ELF3, PYK20 |
| SiChr.1 | 22350001 | 22490000 | 1737 | 0.72 | 0.70 | 2.47 | Sin1G01868.1 | AT1G57820.1 | Symbols: VIM1, ORTH2 |
| SiChr.1 | 21595001 | 21790000 | 3599 | 0.71 | 0.61 | 4.24 | Sin1G01799.1 | AT5G10140.2 | Symbols: FLC, RSB6, AGL25, FLF |
| SiChr.5 | 12870001 | 13100000 | 3146 | 0.72 | 0.64 | 6.05 | Sin5G00995.1 | AT4G23100.1 | Symbols: CAD2, GSHA, RAX1, AtGSH1, PAD2, RML1, ATECS1, GSH1 |
| SiChr.1 | 2465001 | 2555000 | 990 | 0.71 | 0.60 | 2.70 | Sin1G00338.2 | AT2G32950.1 | Symbols: ATCOP1, COP1, DET340, EMB168, FUS1 |
| SiChr.9 | 675001 | 820000 | 1620 | 0.68 | 0.63 | 5.99 | Sin9G00103.1 | AT2G42280.1 | Symbols: FBH4, AKS3 |
| SiChr.2 | 27895001 | 27950000 | 590 | 0.70 | 0.64 | 3.93 | Sin2G02616.1 | AT4G37280.1 | Symbols: MRG1 |
| SiChr.3 | 745001 | 905000 | 1690 | 0.68 | 0.59 | 3.04 | Sin3G00132.1 | AT3G44110.1 | Symbols: ATJ3, ATJ, J3 |
| SiChr.7 | 23290001 | 23365000 | 426 | 0.69 | 0.61 | 4.89 | Sin7G02124.1 | AT4G16280.4 | Symbols: FCA |
| SiChr.7 | 23365001 | 23430000 | 384 | 0.70 | 0.63 | 4.79 | Sin7G02140.1 | AT1G47990.1 | Symbols: ATGA2OX4, GA2OX4 |
| SiChr.1 | 17775001 | 17830000 | 332 | 0.69 | 0.67 | 3.24 | Sin1G01299.1 | AT4G22140.2 | Symbols: EBS |
| SiChr.3 | 20815001 | 20870000 | 1176 | 0.66 | 0.51 | 2.69 | Sin3G01981.1 | AT1G54830.1 | Symbols: NF-YC3 |
| SiChr.3 | 6425001 | 6465000 | 588 | 0.65 | 0.52 | 3.77 | Sin3G00894.1 | AT3G44680.1 | Symbols: HDAC9, HDA09, HDA9, AtHDAC9, AtHDA9 |
| SiChr.10 | 13610001 | 13755000 | 1338 | 0.65 | 0.61 | 4.73 | Sin10G01230.1 | AT3G04030.1 | Symbols: MYR2 |
| SiChr.10 | 13610001 | 13755000 | 1338 | 0.65 | 0.61 | 4.73 | Sin10G01241.1 | AT1G65480.1 | Symbols: RSB8, FT |
| SiChr.10 | 13610001 | 13755000 | 1338 | 0.65 | 0.61 | 4.73 | Sin10G01248.1 | AT5G03840.1 | Symbols: TFL1, TFL-1 |
| SiChr.13 | 8335001 | 8375000 | 581 | 0.65 | 0.47 | 10.41 | Sin13G00712.2 | AT2G43010.2 | Symbols: SRL2, AtPIF4, PIF4 |
| SiChr.9 | 17905001 | 17960000 | 410 | 0.66 | 0.65 | 2.10 | Sin9G01814.1 | AT5G06600.1 | Symbols: UBP12, AtUBP12 |
| SiChr.2 | 26345001 | 26365000 | 6 | 0.65 | 0.24 | 3.44 | Sin2G02435.1 | AT4G08920.1 | Symbols: OOP2, BLU1, ATCRY1, CRY1, HY4 |
| SiChr.4 | 8700001 | 8750000 | 1412 | 0.63 | 0.47 | 2.36 | Sin4G01188.1 | AT5G06600.2 | Symbols: UBP12, AtUBP12 |
| SiChr.8 | 14155001 | 14180000 | 485 | 0.63 | 0.57 | 5.55 | Sin8G01130.1 | AT1G04870.2 | Symbols: PRMT10, ATPRMT10 |

| **Table S20 Candidate genes putatively associated with DF using GWAS method.** | | | | | | | | | | | | |
| --- | --- | --- | --- | --- | --- | --- | --- | --- | --- | --- | --- | --- |
| **Trait** | **SiChr.osome no.** | **Variant no.** | **Variant information** | **Reference genome** | **Position** | **P value** | **PVE (%)** | **Func.refGene** | **Related gene ID** | **Homology_ID** | **Homology_annotation** | **ExonicFunc.refGene** |
| FD_Yuanyang(2022) | SiChr.2 | 2_26544331 | A | G | 26544331 | 1.03E-09 | 7.637405245 | Exonic | Sin2G02467 | AT4G24560.1 | Symbols: UBP16 | synonymous SNV |
| FD_Yuanyang(2020) | SiChr.2 | 2_26522801 | A | T | 26522801 | 1.31E-13 | 1.942003091 | intronic | Sin2G02467 | AT4G24560.1 | Symbols: UBP16 | - |

| **Table S21 Gene flow intervals from 'South Asia' population into 'North China' population.** | | | |
| --- | --- | --- | --- |
| **SiChr.osome no.** | **Start ste (bp)** | **End site (bp)** | **Trinscripts_IDs** |
| SiChr.1 | 910001 | 930000 | Sin1G00153.1,Sin1G00154.1,Sin1G00155.1 |
| SiChr.1 | 1000001 | 1030000 | Sin1G00166.1,Sin1G00167.1,Sin1G00168.1,Sin1G00169.1 |
| SiChr.1 | 1100001 | 1140000 | Sin1G00176.1,Sin1G00177.1,Sin1G00178.1,Sin1G00179.1,Sin1G00180.1,Sin1G00181.1 |
| SiChr.1 | 6645001 | 6800000 | Sin1G00751.1,Sin1G00752.1,Sin1G00753.1,Sin1G00754.1,Sin1G00755.1,Sin1G00756.1,Sin1G00757.1,Sin1G00758.1,Sin1G00759.1,Sin1G00760.1,Sin1G00761.1,Sin1G00762.1,Sin1G00763.1,Sin1G00764.1,Sin1G00765.1,Sin1G00766.1 |
| SiChr.1 | 6815001 | 6835000 | Sin1G00770.1,Sin1G00771.1 |
| SiChr.1 | 6845001 | 6890000 | Sin1G00772.1,Sin1G00773.1,Sin1G00774.1,Sin1G00775.1 |
| SiChr.1 | 6895001 | 7005000 | Sin1G00776.1,Sin1G00777.1,Sin1G00778.1,Sin1G00779.1,Sin1G00780.1,Sin1G00781.1,Sin1G00782.1 |
| SiChr.1 | 16450001 | 16475000 | Sin1G01084.1,Sin1G01085.1,Sin1G01086.1 |
| SiChr.1 | 27245001 | 27270000 | Sin1G02428.1,Sin1G02429.1,Sin1G02430.1,Sin1G02431.1,Sin1G02432.1,Sin1G02433.1 |
| SiChr.1 | 28930001 | 28965000 | Sin1G02687.1,Sin1G02688.1,Sin1G02689.3,Sin1G02690.1,Sin1G02691.1,Sin1G02692.1 |
| SiChr.2 | 3810001 | 3845000 | Sin2G00451.1,Sin2G00452.1,Sin2G00453.1,Sin2G00454.1,Sin2G00455.1 |
| SiChr.2 | 3850001 | 3895000 | Sin2G00457.1,Sin2G00458.1,Sin2G00459.1,Sin2G00460.1,Sin2G00461.2 |
| SiChr.2 | 3905001 | 3935000 | Sin2G00463.1,Sin2G00464.2,Sin2G00465.1,Sin2G00466.1 |
| SiChr.2 | 5215001 | 5235000 | Sin2G00623.1 |
| SiChr.2 | 5510001 | 5610000 | Sin2G00647.1,Sin2G00648.3,Sin2G00649.1,Sin2G00650.1,Sin2G00651.1,Sin2G00652.1,Sin2G00653.1,Sin2G00654.1,Sin2G00655.1,Sin2G00656.1,Sin2G00657.1,Sin2G00658.1 |
| SiChr.2 | 5625001 | 5650000 | Sin2G00660.1,Sin2G00661.1 |
| SiChr.2 | 7075001 | 7100000 | Sin2G00806.1,Sin2G00807.1,Sin2G00808.1,Sin2G00809.1 |
| SiChr.2 | 7465001 | 7505000 | Sin2G00828.1,Sin2G00829.1 |
| SiChr.2 | 13340001 | 13375000 | Sin2G00985.2,Sin2G00986.1,Sin2G00987.1,Sin2G00988.1 |
| SiChr.2 | 13875001 | 13895000 | Sin2G01050.1,Sin2G01051.1,Sin2G01052.1,Sin2G01053.1,Sin2G01054.1 |
| SiChr.2 | 13930001 | 13970000 | Sin2G01067.1,Sin2G01068.1,Sin2G01069.1,Sin2G01070.1,Sin2G01071.1,Sin2G01072.1 |
| SiChr.2 | 14005001 | 14095000 | Sin2G01079.1,Sin2G01080.1,Sin2G01081.2,Sin2G01082.1,Sin2G01083.1,Sin2G01084.1 |
| SiChr.2 | 14095001 | 14145000 | Sin2G01085.1,Sin2G01086.1,Sin2G01087.1,Sin2G01088.1,Sin2G01089.1 |
| SiChr.2 | 14160001 | 14215000 | Sin2G01091.1,Sin2G01092.1,Sin2G01093.1,Sin2G01094.1,Sin2G01095.1,Sin2G01096.1 |
| SiChr.2 | 16225001 | 16260000 | Sin2G01419.1,Sin2G01420.1,Sin2G01421.1,Sin2G01422.1,Sin2G01423.1,Sin2G01424.1,Sin2G01425.1,Sin2G01426.1,Sin2G01427.1,Sin2G01428.1,Sin2G01429.2,Sin2G01430.1,Sin2G01431.2 |
| SiChr.2 | 17100001 | 17130000 | Sin2G01522.1,Sin2G01523.1 |
| SiChr.2 | 17180001 | 17200000 | Sin2G01529.1,Sin2G01530.1,Sin2G01531.1,Sin2G01532.1 |
| SiChr.2 | 17245001 | 17265000 | Sin2G01539.1,Sin2G01540.1,Sin2G01541.2,Sin2G01542.1 |
| SiChr.2 | 19430001 | 19470000 | Sin2G01757.1,Sin2G01758.1 |
| SiChr.2 | 21980001 | 22030000 | Sin2G02024.1 |
| SiChr.2 | 23665001 | 23705000 | Sin2G02170.1,Sin2G02171.1,Sin2G02172.1,Sin2G02173.1,Sin2G02174.1,Sin2G02175.1 |
| SiChr.2 | 26510001 | 26550000 | Sin2G02463.1,Sin2G02464.1,Sin2G02465.1,Sin2G02466.5,Sin2G02467.1,Sin2G02468.1 |
| SiChr.2 | 30105001 | 30125000 | Sin2G03005.1,Sin2G03006.1,Sin2G03007.1,Sin2G03008.1,Sin2G03009.1,Sin2G03010.1,Sin2G03011.3 |
| SiChr.2 | 30235001 | 30255000 | Sin2G03030.1,Sin2G03031.1 |
| SiChr.3 | 1875001 | 1935000 | Sin3G00296.2,Sin3G00297.1,Sin3G00298.2,Sin3G00299.1,Sin3G00300.1,Sin3G00301.1,Sin3G00302.3,Sin3G00303.1 |
| SiChr.3 | 1965001 | 1995000 | Sin3G00308.1,Sin3G00309.1,Sin3G00310.1 |
| SiChr.3 | 4540001 | 4580000 | Sin3G00668.1,Sin3G00669.1,Sin3G00670.1,Sin3G00671.1,Sin3G00672.1,Sin3G00673.1,Sin3G00674.1,Sin3G00675.2 |
| SiChr.3 | 6505001 | 6530000 | Sin3G00900.1,Sin3G00901.1,Sin3G00902.1 |
| SiChr.3 | 7770001 | 7835000 | Sin3G01082.1,Sin3G01083.1,Sin3G01084.1,Sin3G01085.1,Sin3G01086.1,Sin3G01087.1,Sin3G01088.1,Sin3G01089.1,Sin3G01090.1,Sin3G01091.1,Sin3G01092.1,Sin3G01093.1,Sin3G01094.1 |
| SiChr.3 | 17200001 | 17235000 | Sin3G01571.1,Sin3G01572.1,Sin3G01573.1,Sin3G01574.1 |
| SiChr.3 | 19800001 | 19825000 | Sin3G01842.1,Sin3G01843.1,Sin3G01844.1,Sin3G01845.1 |
| SiChr.3 | 19845001 | 19895000 | Sin3G01847.4,Sin3G01848.1,Sin3G01849.1,Sin3G01850.1,Sin3G01851.1,Sin3G01852.1,Sin3G01853.1,Sin3G01854.3 |
| SiChr.3 | 19910001 | 19945000 | Sin3G01856.1,Sin3G01857.1,Sin3G01858.3,Sin3G01859.1,Sin3G01860.1,Sin3G01861.1 |
| SiChr.3 | 20690001 | 20745000 | Sin3G01958.1,Sin3G01959.4,Sin3G01960.1,Sin3G01961.1,Sin3G01962.1,Sin3G01963.1,Sin3G01964.1,Sin3G01965.1,Sin3G01966.2 |
| SiChr.3 | 20745001 | 20790000 | Sin3G01966.2,Sin3G01967.1,Sin3G01968.1,Sin3G01969.1,Sin3G01970.3,Sin3G01971.1,Sin3G01972.1,Sin3G01973.1 |
| SiChr.3 | 20795001 | 20840000 | Sin3G01974.2,Sin3G01975.1,Sin3G01976.1,Sin3G01977.1,Sin3G01978.1,Sin3G01979.1,Sin3G01980.1,Sin3G01981.1 |
| SiChr.3 | 20855001 | 20875000 | Sin3G01982.2 |
| SiChr.3 | 20905001 | 20995000 | Sin3G01987.1,Sin3G01988.2,Sin3G01989.1,Sin3G01990.1,Sin3G01991.2,Sin3G01992.1,Sin3G01993.1,Sin3G01994.1,Sin3G01995.1,Sin3G01996.6,Sin3G01997.1,Sin3G01998.1,Sin3G01999.2,Sin3G02000.2,Sin3G02001.1,Sin3G02002.1,Sin3G02003.1,Sin3G02004.1,Sin3G02005.1,Sin3G02006.1 |
| SiChr.3 | 21000001 | 21055000 | Sin3G02006.1,Sin3G02007.1,Sin3G02008.1,Sin3G02009.1,Sin3G02010.1,Sin3G02011.1,Sin3G02012.1,Sin3G02013.1 |
| SiChr.4 | 645001 | 715000 | Sin4G00075.1,Sin4G00076.1,Sin4G00077.1,Sin4G00078.1,Sin4G00079.1,Sin4G00080.1,Sin4G00081.1,Sin4G00082.1 |
| SiChr.4 | 750001 | 790000 | Sin4G00086.1,Sin4G00087.1,Sin4G00088.1,Sin4G00089.1,Sin4G00090.1,Sin4G00091.1,Sin4G00092.6,Sin4G00093.1,Sin4G00094.1 |
| SiChr.4 | 810001 | 835000 | Sin4G00097.1,Sin4G00098.1,Sin4G00099.1,Sin4G00100.1,Sin4G00101.4 |
| SiChr.4 | 2050001 | 2085000 | Sin4G00255.1,Sin4G00256.1,Sin4G00257.1,Sin4G00258.1,Sin4G00259.1 |
| SiChr.4 | 7135001 | 7165000 | Sin4G00950.1,Sin4G00951.2,Sin4G00952.1,Sin4G00953.1,Sin4G00954.1,Sin4G00955.1,Sin4G00956.1 |
| SiChr.4 | 18895001 | 18920000 | Sin4G01915.1,Sin4G01916.1,Sin4G01917.1,Sin4G01918.1 |
| SiChr.4 | 19760001 | 19785000 | Sin4G02021.1,Sin4G02022.1,Sin4G02023.1 |
| SiChr.4 | 21560001 | 21600000 | Sin4G02198.1,Sin4G02199.1,Sin4G02200.1,Sin4G02201.1,Sin4G02202.1 |
| SiChr.4 | 21865001 | 21890000 | Sin4G02221.2,Sin4G02222.1,Sin4G02223.1 |
| SiChr.4 | 21900001 | 21920000 | Sin4G02224.1,Sin4G02225.1 |
| SiChr.4 | 21995001 | 22020000 | Sin4G02233.1,Sin4G02234.1,Sin4G02235.1,Sin4G02236.1,Sin4G02237.1,Sin4G02238.1 |
| SiChr.4 | 22040001 | 22075000 | Sin4G02239.1,Sin4G02240.2,Sin4G02241.1 |
| SiChr.4 | 22090001 | 22135000 | Sin4G02241.1,Sin4G02242.1,Sin4G02243.1,Sin4G02244.1,Sin4G02245.1,Sin4G02246.1,Sin4G02247.1 |
| SiChr.4 | 22155001 | 22180000 | Sin4G02249.1,Sin4G02250.1,Sin4G02251.1 |
| SiChr.4 | 25115001 | 25135000 | Sin4G02576.1,Sin4G02577.1,Sin4G02578.1,Sin4G02579.1 |
| SiChr.4 | 25140001 | 25175000 | Sin4G02580.2,Sin4G02581.3,Sin4G02582.1,Sin4G02583.1 |
| SiChr.5 | 3660001 | 3780000 | Sin5G00457.1,Sin5G00458.1,Sin5G00459.1,Sin5G00460.1,Sin5G00461.1,Sin5G00462.1,Sin5G00463.1,Sin5G00464.1,Sin5G00465.1,Sin5G00466.1,Sin5G00467.1,Sin5G00468.1 |
| SiChr.5 | 3795001 | 3820000 | Sin5G00469.1 |
| SiChr.5 | 4445001 | 4490000 | Sin5G00527.1,Sin5G00528.1 |
| SiChr.5 | 4600001 | 4850000 | Sin5G00537.1,Sin5G00538.1,Sin5G00539.1,Sin5G00540.1,Sin5G00541.1,Sin5G00542.2,Sin5G00543.1,Sin5G00544.1,Sin5G00545.1,Sin5G00546.1,Sin5G00547.1,Sin5G00548.1,Sin5G00549.1,Sin5G00550.1,Sin5G00551.1,Sin5G00552.1,Sin5G00553.1,Sin5G00554.1,Sin5G00555.1,Sin5G00556.1,Sin5G00557.1 |
| SiChr.5 | 5750001 | 5770000 | Sin5G00646.1,Sin5G00647.1,Sin5G00648.1,Sin5G00649.1 |
| SiChr.5 | 5780001 | 5805000 | Sin5G00651.1,Sin5G00652.1,Sin5G00653.1 |
| SiChr.5 | 6015001 | 6045000 | Sin5G00672.1 |
| SiChr.5 | 11900001 | 11920000 | Sin5G00898.1,Sin5G00899.1,Sin5G00900.1,Sin5G00901.1,Sin5G00902.1 |
| SiChr.5 | 11930001 | 11950000 | Sin5G00904.1,Sin5G00905.1 |
| SiChr.6 | 1205001 | 1240000 | Sin6G00224.1,Sin6G00225.1,Sin6G00226.1,Sin6G00227.1,Sin6G00228.1 |
| SiChr.6 | 1350001 | 1485000 | Sin6G00244.1,Sin6G00246.1,Sin6G00247.1,Sin6G00248.1,Sin6G00249.1,Sin6G00250.1,Sin6G00251.1,Sin6G00252.1,Sin6G00253.1,Sin6G00254.1,Sin6G00255.1,Sin6G00256.1,Sin6G00257.1,Sin6G00258.1,Sin6G00259.1 |
| SiChr.6 | 1510001 | 1550000 | Sin6G00263.1,Sin6G00264.4,Sin6G00265.1,Sin6G00266.1,Sin6G00267.1 |
| SiChr.6 | 4625001 | 4645000 | Sin6G00581.8,Sin6G00582.1,Sin6G00583.1 |
| SiChr.6 | 23070001 | 23105000 | Sin6G02143.1,Sin6G02144.1,Sin6G02145.1,Sin6G02146.1,Sin6G02147.1 |
| SiChr.7 | 5710001 | 5740000 | Sin7G00709.1,Sin7G00710.1,Sin7G00711.1 |
| SiChr.7 | 5740001 | 5850000 | Sin7G00711.1,Sin7G00712.1,Sin7G00713.1,Sin7G00714.1,Sin7G00715.1,Sin7G00716.2,Sin7G00717.1,Sin7G00718.1,Sin7G00719.1,Sin7G00720.1,Sin7G00721.1 |
| SiChr.7 | 5870001 | 5930000 | Sin7G00723.1,Sin7G00724.1,Sin7G00725.1,Sin7G00726.1,Sin7G00727.1,Sin7G00728.1,Sin7G00729.1 |
| SiChr.7 | 5960001 | 5995000 | Sin7G00731.1,Sin7G00732.1,Sin7G00733.1,Sin7G00734.1 |
| SiChr.7 | 7250001 | 7335000 | Sin7G00752.1,Sin7G00753.1,Sin7G00754.1,Sin7G00755.1,Sin7G00756.1,Sin7G00757.1,Sin7G00758.1 |
| SiChr.7 | 7340001 | 7410000 | Sin7G00758.1,Sin7G00759.1,Sin7G00760.1,Sin7G00761.1,Sin7G00762.1,Sin7G00763.1,Sin7G00764.1,Sin7G00765.1,Sin7G00766.1 |
| SiChr.7 | 16280001 | 16305000 | Sin7G01220.1,Sin7G01221.1,Sin7G01222.1,Sin7G01223.1 |
| SiChr.7 | 16320001 | 16345000 | Sin7G01225.1,Sin7G01226.1 |
| SiChr.7 | 16380001 | 16410000 | Sin7G01230.5,Sin7G01231.1,Sin7G01232.1,Sin7G01233.1 |
| SiChr.7 | 17120001 | 17140000 | Sin7G01304.1,Sin7G01305.1 |
| SiChr.7 | 17160001 | 17190000 | Sin7G01307.2,Sin7G01308.1,Sin7G01309.1 |
| SiChr.8 | 12480001 | 12500000 | Sin8G00985.1,Sin8G00986.1,Sin8G00987.1,Sin8G00988.1,Sin8G00989.1,Sin8G00990.1 |
| SiChr.8 | 13235001 | 13270000 | Sin8G01052.1,Sin8G01053.1 |
| SiChr.8 | 13520001 | 13565000 | Sin8G01072.1,Sin8G01073.1,Sin8G01074.1,Sin8G01075.1,Sin8G01076.1,Sin8G01077.1 |
| SiChr.8 | 13640001 | 13675000 | Sin8G01083.1,Sin8G01084.1,Sin8G01085.1,Sin8G01086.1,Sin8G01087.1 |
| SiChr.8 | 15135001 | 15180000 | Sin8G01221.1,Sin8G01222.1,Sin8G01223.1,Sin8G01224.1,Sin8G01225.1,Sin8G01226.1,Sin8G01227.1,Sin8G01228.1 |
| SiChr.9 | 55001 | 85000 | Sin9G00002.1,Sin9G00003.1,Sin9G00004.1,Sin9G00005.1 |
| SiChr.9 | 85001 | 425000 | Sin9G00005.1,Sin9G00006.1,Sin9G00007.1,Sin9G00008.1,Sin9G00009.1,Sin9G00010.1,Sin9G00011.2,Sin9G00012.2,Sin9G00013.1,Sin9G00014.2,Sin9G00015.1,Sin9G00016.1,Sin9G00017.1,Sin9G00018.1,Sin9G00019.1,Sin9G00020.1,Sin9G00021.1,Sin9G00022.1,Sin9G00023.3,Sin9G00024.1,Sin9G00025.1,Sin9G00026.1,Sin9G00027.1,Sin9G00028.1,Sin9G00029.1,Sin9G00030.1,Sin9G00031.1,Sin9G00032.1,Sin9G00033.1,Sin9G00034.1,Sin9G00035.1,Sin9G00036.1,Sin9G00037.1,Sin9G00038.1,Sin9G00039.1,Sin9G00040.1,Sin9G00041.2,Sin9G00042.1,Sin9G00043.5,Sin9G00044.8,Sin9G00045.1,Sin9G00046.1,Sin9G00047.1,Sin9G00048.1,Sin9G00049.1,Sin9G00050.1,Sin9G00051.1,Sin9G00052.1,Sin9G00053.1,Sin9G00054.1,Sin9G00055.1,Sin9G00056.1,Sin9G00057.1,Sin9G00058.1 |
| SiChr.9 | 615001 | 635000 | Sin9G00089.1,Sin9G00090.1,Sin9G00091.1,Sin9G00092.1 |
| SiChr.9 | 640001 | 670000 | Sin9G00094.1,Sin9G00095.1,Sin9G00096.1 |
| SiChr.9 | 3495001 | 3555000 | Sin9G00471.1,Sin9G00472.1,Sin9G00473.1,Sin9G00474.1,Sin9G00475.1,Sin9G00476.1,Sin9G00477.1,Sin9G00478.2 |
| SiChr.9 | 13760001 | 13835000 | Sin9G01432.1,Sin9G01433.1,Sin9G01434.1,Sin9G01435.1,Sin9G01436.1,Sin9G01437.1,Sin9G01438.1,Sin9G01439.1,Sin9G01440.1,Sin9G01441.1,Sin9G01442.1,Sin9G01443.5 |
| SiChr.9 | 15450001 | 15560000 | Sin9G01630.1,Sin9G01631.1,Sin9G01632.1,Sin9G01633.1,Sin9G01634.1,Sin9G01635.1,Sin9G01636.1,Sin9G01637.1,Sin9G01638.1,Sin9G01639.1,Sin9G01640.1 |
| SiChr.9 | 15660001 | 15680000 | Sin9G01647.1,Sin9G01648.1 |
| SiChr.9 | 20560001 | 20585000 | Sin9G02069.1,Sin9G02070.1,Sin9G02071.1 |
| SiChr.10 | 1665001 | 1765000 | Sin10G00203.2,Sin10G00204.1,Sin10G00205.1,Sin10G00206.1,Sin10G00207.1,Sin10G00208.1,Sin10G00209.1 |
| SiChr.10 | 9240001 | 9295000 | Sin10G00750.1,Sin10G00751.1,Sin10G00752.1 |
| SiChr.10 | 9330001 | 9365000 | Sin10G00758.1,Sin10G00759.1,Sin10G00760.2,Sin10G00761.1 |
| SiChr.10 | 11115001 | 11290000 | Sin10G00943.1,Sin10G00944.1,Sin10G00945.1,Sin10G00946.1,Sin10G00947.1,Sin10G00948.1,Sin10G00949.1,Sin10G00950.1,Sin10G00951.1,Sin10G00952.1,Sin10G00953.1,Sin10G00954.1,Sin10G00955.1,Sin10G00956.1,Sin10G00957.1,Sin10G00958.1,Sin10G00959.1,Sin10G00960.1,Sin10G00961.1,Sin10G00962.1,Sin10G00963.1,Sin10G00964.1,Sin10G00965.1,Sin10G00966.1,Sin10G00967.1,Sin10G00968.2,Sin10G00969.1,Sin10G00970.1,Sin10G00971.2,Sin10G00972.1 |
| SiChr.10 | 15755001 | 15780000 | Sin10G01573.1,Sin10G01574.1,Sin10G01575.1,Sin10G01576.1 |
| SiChr.12 | 1760001 | 1800000 | Sin12G00256.1,Sin12G00257.1,Sin12G00258.1 |
| SiChr.12 | 1880001 | 1915000 |  |
| SiChr.12 | 8210001 | 8235000 | Sin12G00625.1,Sin12G00626.1,Sin12G00627.1,Sin12G00628.1,Sin12G00629.1 |
| SiChr.12 | 8350001 | 8375000 | Sin12G00640.1,Sin12G00641.1,Sin12G00642.1,Sin12G00643.1,Sin12G00644.1 |
| SiChr.12 | 12630001 | 12655000 | Sin12G01199.1 |
| SiChr.12 | 18005001 | 18030000 | Sin12G01748.1,Sin12G01749.1,Sin12G01750.1,Sin12G01751.1,Sin12G01752.1,Sin12G01753.1,Sin12G01754.1 |
| SiChr.13 | 1890001 | 1915000 |  |
| SiChr.13 | 1920001 | 1940000 | Sin13G00313.2,Sin13G00314.1 |
| SiChr.13 | 1945001 | 1980000 | Sin13G00315.1,Sin13G00316.1,Sin13G00317.1,Sin13G00318.1,Sin13G00319.1,Sin13G00320.1,Sin13G00321.1,Sin13G00322.1 |
| SiChr.13 | 1990001 | 2040000 | Sin13G00324.1,Sin13G00325.1,Sin13G00326.1 |
| SiChr.13 | 2105001 | 2145000 | Sin13G00337.1,Sin13G00338.1,Sin13G00339.1,Sin13G00340.1 |
| SiChr.13 | 2150001 | 2175000 | Sin13G00341.1,Sin13G00342.1 |
| SiChr.13 | 2190001 | 2245000 | Sin13G00345.1,Sin13G00346.1,Sin13G00347.1,Sin13G00348.1,Sin13G00349.1,Sin13G00350.1,Sin13G00351.1 |
| SiChr.13 | 2260001 | 2285000 | Sin13G00356.1,Sin13G00357.1,Sin13G00358.1,Sin13G00359.1 |
| SiChr.13 | 2725001 | 2755000 | Sin13G00388.1,Sin13G00389.1,Sin13G00390.1 |
| SiChr.13 | 2855001 | 2905000 | Sin13G00399.1,Sin13G00400.1,Sin13G00401.1,Sin13G00402.1,Sin13G00403.1 |
| SiChr.13 | 2905001 | 2965000 | Sin13G00403.1,Sin13G00404.1,Sin13G00405.1 |
| SiChr.13 | 2970001 | 3000000 | Sin13G00406.1,Sin13G00407.1,Sin13G00408.1,Sin13G00409.1 |
| SiChr.13 | 3040001 | 3100000 | Sin13G00414.1,Sin13G00415.1,Sin13G00416.1,Sin13G00417.1,Sin13G00418.1,Sin13G00419.1,Sin13G00420.1 |
| SiChr.13 | 9455001 | 9495000 | Sin13G00812.1,Sin13G00813.1,Sin13G00814.1,Sin13G00815.1,Sin13G00816.1 |
| SiChr.13 | 10560001 | 10585000 | Sin13G00917.1,Sin13G00918.1,Sin13G00919.1,Sin13G00920.2 |
| SiChr.13 | 13290001 | 13335000 | Sin13G01270.1,Sin13G01271.2,Sin13G01272.1,Sin13G01273.1,Sin13G01274.1,Sin13G01275.1,Sin13G01276.1,Sin13G01277.2 |
| SiChr.13 | 14515001 | 14535000 | Sin13G01480.1,Sin13G01481.1,Sin13G01482.1,Sin13G01483.1 |

| **Table S22 Number of genes keeping ZnF UBP domain identified in 27 species.** | | | |
| --- | --- | --- | --- |
| **Species** | **UCH(PF00443.32)** | **zf-MYND(PF01753.21)** | **Combined** |
| Arabidopsis thaliana | 44 | 11 | 6 |
| Brassica rapa | 51 | 12 | 5 |
| Cicer arietinum | 43 | 18 | 7 |
| Crambe hispanica | 56 | 16 | 8 |
| Eruca vesicaria | 103 | 24 | 12 |
| Hirschfeldia incana | 59 | 20 | 11 |
| Linum usitatissimum | 51 | 15 | 4 |
| Zea mays | 112 | 28 | 10 |
| Mimulus hybridus Wettst | 32 | 11 | 4 |
| Nicotiana tabacum | 64 | 12 | 6 |
| Arachis hypogaea | 95 | 44 | 11 |
| Gossypium raimondii | 131 | 41 | 17 |
| Oryza sativa | 40 | 13 | 3 |
| Ricinus communis | 66 | 20 | 13 |
| Carthamus tinctorius | 36 | 10 | 3 |
| Sinapis alba | 92 | 27 | 14 |
| Glycine max | 156 | 53 | 34 |
| Helianthus annuus | 50 | 17 | 6 |
| Gossypium arboreum | 72 | 23 | 12 |
| Perilla frutescens | 58 | 13 | 5 |
| Sesamum indicum.var Yuzhi11 | 37 | 11 | 6 |
| Sesamum calycinum | 39 | 7 | 3 |
| Sesamum latifolium | 34 | 9 | 4 |
| Sesamum angolense | 40 | 9 | 5 |
| Sesamum radiatum | 74 | 13 | 5 |
| Sesamum angolense | 37 | 7 | 3 |
| Sesamum alatum | 31 | 10 | 5 |

| **Table S23 Prediction for PXLXP motif of homologous sesame genes to *Arabidopsis thaliana* regulating flowering time.** | | | | | | | | |
| --- | --- | --- | --- | --- | --- | --- | --- | --- |
| **Sequence name** | **Strand** | **Start** | **End** | **p-value** | **q-value** | **Matched Motif Sequence** | **Homology_ID** | **Homology_annotation** |
| Sin11G00862.1 | + | 370 | 374 | 1.23E-06 | 0.137 | PHLHP | AT3G33520.1 | Symbols: SUF3, ARP6, ESD1, ATARP6 |
| Sin12G00486.1 | + | 36 | 40 | 6.48E-05 | 0.42 | PNLDP | AT5G06600.2 | Symbols: UBP12, AtUBP12 |
| Sin12G01088.1 | + | 19 | 23 | 5.59E-05 | 0.42 | PILFP | AT1G78440.1 | Symbols: GA2OX1, ATGA2OX1 |
| Sin12G01452.1 | + | 158 | 162 | 5.92E-05 | 0.42 | PKLFP | AT2G37630.1 | Symbols: ATMYB91, LL2, AS1, ATPHAN, MYB91 |
| Sin12G01654.1 | + | 391 | 395 | 8.19E-06 | 0.25 | PHLFP | AT5G14170.1 | Symbols: BAF60, SWP73B, CHC1 |
| Sin13G00883.1 | + | 345 | 349 | 3.74E-05 | 0.42 | PQLQP | AT2G20570.1 | Symbols: ATGLK1, GLK1, GPRI1 |
| Sin13G01889.1 | + | 1166 | 1170 | 6.14E-06 | 0.25 | PTLWP | AT4G04920.3 | Symbols: IEN1, AtSFR6, YID1, MED16, GLH2, SFR6 |
| Sin13G02102.1 | + | 629 | 633 | 2.31E-05 | 0.376 | PKLHP | AT1G78580.1 | Symbols: TPS1, ATTPS1 |
| Sin13G02336.1 | + | 200 | 204 | 8.40E-05 | 0.454 | PGLQP | AT1G30970.1 | Symbols: SUF4 |
| Sin1G00501.1 | + | 228 | 232 | 9.78E-05 | 0.478 | PVLNP | AT5G39660.1 | Symbols: CDF2 |
| Sin1G01984.1 | + | 1015 | 1019 | 7.50E-05 | 0.426 | PVLQP | AT4G24680.3 | Symbols: MOS1 |
| Sin2G01341.1 | + | 1143 | 1147 | 2.56E-05 | 0.379 | PKLMP | AT1G72390.1 | Symbols: PHL |
| Sin2G02360.1 | + | 241 | 245 | 1.13E-05 | 0.25 | PQLMP | AT1G22770.1 | Symbols: FB, GI |
| Sin3G00903.1 | + | 119 | 123 | 2.37E-05 | 0.376 | PTLHP | AT5G12840.2 | Symbols: NF-YA1, EMB2220, HAP2A, NFYA1, AtNFYA1, ATHAP2A |
| Sin3G00920.1 | + | 94 | 98 | 5.81E-05 | 0.42 | PNLPP | AT1G51450.1 | Symbols: ASH2R, TRAUCO, TRO |
| Sin3G02316.1 | + | 312 | 316 | 1.56E-07 | 0.0346 | PWLCP | AT2G23380.1 | Symbols: CLF, ICU1, SET1, SDG1 |
| Sin3G02452.4 | + | 311 | 315 | 2.83E-05 | 0.392 | PELMP | AT5G39660.1 | Symbols: CDF2 |
| Sin3G02719.1 | + | 393 | 397 | 8.19E-06 | 0.25 | PHLFP | AT5G14170.1 | Symbols: BAF60, SWP73B, CHC1 |
|  | + | 91 | 95 | 8.19E-06 | 0.25 | PHLFP | AT3G26744.1 | Symbols: SCRM, ICE1, SCREAM, ATICE1 |
| Sin3G02779.1 | + | 104 | 108 | 9.36E-05 | 0.478 | PSLFP | AT3G26744.1 | Symbols: SCRM, ICE1, SCREAM, ATICE1 |
| Sin4G00982.1 | + | 123 | 127 | 8.19E-06 | 0.25 | PFLHP | AT4G32980.1 | Symbols: ATH1 |
| Sin4G02486.1 | + | 118 | 122 | 2.37E-05 | 0.376 | PTLHP | AT5G12840.1 | Symbols: NF-YA1, EMB2220, HAP2A, NFYA1, AtNFYA1, ATHAP2A |
| Sin4G02822.2 | + | 1147 | 1151 | 7.01E-05 | 0.42 | PPLPP | AT2G48160.1 | Symbols: HULK2, SL3 |
| Sin5G00183.5 | + | 1562 | 1566 | 6.95E-05 | 0.42 | PELQP | AT3G43920.2 | Symbols: ATDCL3, DCL3 |
| Sin5G01591.1 | + | 477 | 481 | 3.74E-05 | 0.42 | PQLQP | AT1G79730.1 | Symbols: ELF7 |
|  | + | 148 | 152 | 4.83E-05 | 0.42 | PPLQP | AT1G79730.1 | Symbols: ELF7 |
| Sin5G01677.1 | + | 2219 | 2223 | 9.92E-05 | 0.478 | PQLSP | AT1G80070.1 | Symbols: SUS2, EMB177, EMB33, PRP8, EMB14 |
| Sin5G01846.1 | + | 386 | 390 | 2.37E-05 | 0.376 | PHLTP | AT5G14170.1 | Symbols: BAF60, SWP73B, CHC1 |
| Sin5G02245.1 | + | 550 | 554 | 7.01E-05 | 0.42 | PPLPP | AT5G13480.2 | Symbols: FY, WDR33 |
| Sin5G02245.1 | + | 756 | 760 | 7.01E-05 | 0.42 | PPLPP | AT5G13480.2 | Symbols: FY, WDR33 |
| Sin5G02513.1 | + | 234 | 238 | 3.12E-05 | 0.407 | PMLGP | AT2G46020.2 | Symbols: CHA2, BRM, ATBRM, FFO3, CHR2 |
| Sin6G00421.1 | + | 238 | 242 | 1.13E-05 | 0.25 | PQLMP | AT1G22770.1 | Symbols: FB, GI |
| Sin6G01560.1 | + | 1063 | 1067 | 7.01E-05 | 0.42 | PPLPP | AT5G23150.1 | Symbols: ART1, HUA2 |
|  | + | 1100 | 1104 | 7.01E-05 | 0.42 | PPLPP | AT5G23150.1 | Symbols: ART1, HUA2 |
| Sin6G01698.1 | + | 163 | 167 | 4.83E-05 | 0.42 | PPLQP | AT2G01570.1 | Symbols: RGA, RGA1, RGA24 |
| Sin6G01915.1 | + | 199 | 203 | 4.83E-05 | 0.42 | PPLQP | AT1G69120.1 | Symbols: AP1, AtAP1, AGL7 |
|  | + | 196 | 200 | 7.01E-05 | 0.42 | PPLPP | AT1G69120.1 | Symbols: AP1, AtAP1, AGL7 |
| Sin7G01286.1 | + | 789 | 793 | 9.78E-05 | 0.478 | PKLPP | AT2G43410.2 | Symbols: FPA |
| Sin8G00401.1 | + | 1207 | 1211 | 7.50E-05 | 0.426 | PVLQP | AT3G57300.1 | Symbols: INO80, ATINO80 |
|  | + | 175 | 179 | 8.67E-05 | 0.458 | PNLEP | AT3G57300.1 | Symbols: INO80, ATINO80 |
| Sin8G00824.1 | + | 1255 | 1259 | 8.00E-05 | 0.444 | PNLKP | AT5G20320.1 | Symbols: DCL4, ATDCL4 |
| Sin9G00732.1 | + | 1146 | 1150 | 7.01E-05 | 0.42 | PPLPP | AT5G23150.1 | Symbols: ART1, HUA2 |
|  | + | 1168 | 1172 | 7.01E-05 | 0.42 | PPLPP | AT5G23150.1 | Symbols: ART1, HUA2 |
| Sin9G01086.1 | + | 540 | 544 | 6.60E-05 | 0.42 | PRLNP | AT5G24470.1 | Symbols: APRR5, PRR5 |
| Sin9G01828.1 | + | 40 | 44 | 5.38E-05 | 0.42 | PNLNP | AT3G10390.1 | Symbols: FLD, RSI1 |
| Sin9G01834.1 | + | 342 | 346 | 1.04E-05 | 0.25 | PRLCP | AT4G25420.1 | Symbols: ATGA20OX1, AT2301, GA5, GA20OX1 |
